# Estimating resource acquisition and at-sea body condition of a marine predator

**DOI:** 10.1111/1365-2656.12102

**Published:** 2013-07-19

**Authors:** Robert S Schick, Leslie F New, Len Thomas, Daniel P Costa, Mark A Hindell, Clive R McMahon, Patrick W Robinson, Samantha E Simmons, Michele Thums, John Harwood, James S Clark

**Affiliations:** 1 Nicholas School of the Environment, Duke UniversityDurham, NC, 27708, USA; 2 Centre for Research into Ecological and Environmental Modelling, The Observatory, University of St AndrewsSt Andrews, KY16 9LZ, UK; 3 Department of Ecology and Evolutionary Biology, University of California Santa CruzSanta Cruz, California, 95064, USA; 4 Marine Predator Unit, Institute for Marine and Antarctic Studies, University of TasmaniaPrivate Bag 129, Hobart, Tasmania, 7001, Australia; 5 Research Institute for the Environment and Livelihoods, Charles Darwin UniversityDarwin, Northern Territory, 0909, Australia; 6 Marine Mammal Commission4340 East-West Highway, Suite 700, Bethesda, MD, 20814, USA; 7 School of Environmental Systems Engineering and UWA Oceans Institute, The University of Western AustraliaM470, 35 Stirling Highway, Crawley, WA, 6009, Australia; 8 Australian Institute of Marine Science, UWA Oceans Institute, The University of Western AustraliaM096, 35 Stirling Highway, Crawley, WA, 6009, Australia

**Keywords:** resource acquisition, Bayesian, elephant seals, Markov chain Monte Carlo, satellite telemetry, state-space model, body condition, Año Nuevo, Macquarie Island

## Abstract

1. Body condition plays a fundamental role in many ecological and evolutionary processes at a variety of scales and across a broad range of animal taxa. An understanding of how body condition changes at fine spatial and temporal scales as a result of interaction with the environment provides necessary information about how animals acquire resources.
2. However, comparatively little is known about intra- and interindividual variation of condition in marine systems. Where condition has been studied, changes typically are recorded at relatively coarse time-scales. By quantifying how fine-scale interaction with the environment influences condition, we can broaden our understanding of how animals acquire resources and allocate them to body stores.
3. Here we used a hierarchical Bayesian state-space model to estimate the body condition as measured by the size of an animal's lipid store in two closely related species of marine predator that occupy different hemispheres: northern elephant seals (*Mirounga angustirostris*) and southern elephant seals (*Mirounga leonina*). The observation model linked drift dives to lipid stores. The process model quantified daily changes in lipid stores as a function of the physiological condition of the seal (lipid:lean tissue ratio, departure lipid and departure mass), its foraging location, two measures of behaviour and environmental covariates.
4. We found that physiological condition significantly impacted lipid gain at two time-scales – daily and at departure from the colony – that foraging location was significantly associated with lipid gain in both species of elephant seals and that long-term behavioural phase was associated with positive lipid gain in northern and southern elephant seals. In northern elephant seals, the occurrence of short-term behavioural states assumed to represent foraging were correlated with lipid gain. Lipid gain was a function of covariates in both species. Southern elephant seals performed fewer drift dives than northern elephant seals and gained lipids at a lower rate.
5. We have demonstrated a new way to obtain time series of body condition estimates for a marine predator at fine spatial and temporal scales. This modelling approach accounts for uncertainty at many levels and has the potential to integrate physiological and movement ecology of top predators. The observation model we used was specific to elephant seals, but the process model can readily be applied to other species, providing an opportunity to understand how animals respond to their environment at a fine spatial scale.

## Introduction

Ecologists have long studied the role of body condition. Studies on a broad and diverse set of taxonomic groups, including ungulates (Festa-Bianchet 1998; Gaillard, Festa-Bianchet & Yoccoz 1998), fishes (Bestley *et al*. 2010), songbirds (Schmaljohann & Naef-Daenzer 2011), seabirds (Weimerskirch 1992) and pinnipeds (McMahon & Burton 2005), have shown how individual phenotypic variation can influence several important aspects of ecology: foraging strategies; individual survival; reproduction; offspring survival; and the impacts of density dependence (Clutton-Brock & Sheldon 2010). Therefore, a better understanding of how individual condition varies over multiple spatial and temporal scales should help us quantify population dynamics in wild populations.

Body condition typically varies as a function of many variables including resource intake, movement, parental care, stressors, predation and environmental conditions (Clutton-Brock, Guinness & Albon 1982). Researchers typically define and refer to body condition as the relative energy stores scaled in some fashion by the structural components of the animal (Green 2001; Peig & Green 2009). In some systems, condition can be measured. For example, in many long-term studies of different ungulate systems (Clutton-Brock, Guinness & Albon 1982), animals can be caught or harvested, measured and weighed to obtain direct measures of condition. Similarly individual birds can be caught and weighed upon return to the nest (Weimerskirch 1998). And in some species of tuna, tagging or harvest efforts provide opportunities to study condition (Goldstein *et al*. 2007; Golet *et al*. 2007; Willis & Hobday 2008). However, in many systems, daily (or similar small time-scales) changes in condition as a function of resource acquisition are difficult or impossible to observe. Therefore, two central questions in these systems include (i) how individuals obtain resources and (ii) how changes in their condition ultimately influence the dynamics of the population. Here we address the first of these questions by developing a model of daily change in condition of a marine predator, the elephant seal (*Mirounga* spp.).

Elephant seals, a colonially breeding marine predator, represent an ideal system to quantify how individual foraging efforts lead to changes in condition. There are two species in this genus: northern elephant seals (NES, *Mirounga angustirostris*) and southern elephant seals (SES, *Mirounga leonina*). Multiple long-term research efforts on each species (Le Boeuf *et al*. 2000; Hindell *et al*. 2003) allow us to explore within- and between-species–level differences in two hemispheres. Because these two species exhibit similar behaviour in markedly different ecosystems, interspecies comparison allows for greater potential inference in how top predators gain condition. In addition, the population trajectories at each of the colonies analysed herein differ. Notably, NES at Año Nuevo appear to have a stable population (Le Boeuf *et al*. 2011), while SES at Macquarie Island are a population in decline (McMahon *et al*. 2005). By comparing the physiological underpinnings of foraging and changes of condition in these two species, we can broaden our understanding of how condition may influence population dynamics.

Elephant seals are long lived with a relatively simple and repeated life-history pattern (Le Boeuf & Laws 1994). Adult female elephant seals alternate two extended trips to sea with two on-land periods for (i) pupping and breeding and (ii) moulting. Following an approximately 1-month-long haul-out to give birth and breed, females make an approximately 3-month-long trip to sea (Le Boeuf & Laws 1994). After this trip, they return to land for approximately 1 month to moult (Le Boeuf & Laws 1994) and then make an extended trip to sea, typically 8 months long (Le Boeuf & Laws 1994). During this trip, the foetus implants, and the animals gain large amount of fat reserves, which they will bring ashore to nourish the pup. Maternal condition is an important factor for juvenile survival (McMahon, Burton & Bester 2000); estimating how it changes at sea could influence understanding of vital rates.

The life-history patterns of elephant seals allow for repeat mark–recaptures and thus direct measures of body condition (Le Boeuf *et al*. 2000). In addition, elephant seals conduct drift dives (Le Boeuf *et al*. 1992, 1996; Crocker, Le Boeuf & Costa 1997; Mitani *et al*. 2009) that provide an at-sea proxy for condition (Biuw *et al*. 2003). These proxies can then be used to identify locations where animals successfully gain lipids, a measure of body condition (Biuw *et al*. 2007; Thums, Bradshaw & Hindell 2008a, 2011; Robinson *et al*. 2010). It is important to note that condition in elephant seals is driven not only by resource acquisition, but also by physiological decisions by the animal to preferentially store lean or lipid tissue (Condit & Ortiz 1987). This is an important behaviour as it can influence the observed drift rates, for example, a seal repairing lean tissue will become denser. In contrast, a seal simply losing lipids will also become denser, although this seal would clearly be in a different condition status than the previous seal.

Beyond identifying areas of change in condition, researchers working on NES and SES have explored how environmental covariates influence change (Biuw *et al*. 2007; Robinson *et al*. 2010). By examining changes in drift rates in both environmental (Biuw *et al*. 2007) and geographical space (Robinson *et al*. 2010; Thums, Bradshaw & Hindell 2011), the first links between condition and the environment have been uncovered. Biuw *et al*. (2007) showed how e-seals gained lipids in water masses with different characteristics, and Robinson *et al*. (2010) showed how covariates like mean daily transit influence observed changes in buoyancy. Thums, Bradshaw & Hindell (2011) explored lipid gain in SES and found that animals foraging in different locations had significantly different lipid gain patterns. Differential lipid gain as a function of foraging location has also been shown for NES (Simmons *et al*. 2007). While there has been much recent research on the influence of behaviour on movement patterns (Nathan *et al*. 2008; Schick *et al*. 2008), there has been less work linking behaviourally specific movement patterns with changes in condition. (Though see Weimerskirch *et al*. (1997) for an example in the seabird literature; see Bailleul *et al*. (2007a) and Dragon *et al*. (2012) for recent examples in SES.) Therefore, for many top marine predators, there are many unanswered questions relating the influence of the environment, behaviour and foraging location on the acquisition of resources.

Here we build upon efforts to understand the ecological processes by which animals gain resources in their environment and allocate them to body stores. The approach proposed herein builds on previous attempts to model condition in the following four ways: (i) we account for uncertainty in both the observations and the process in a coherent framework; (ii) we account for the role that dynamic environmental covariates play in influencing body condition; (iii) we explicitly account for behaviourally specific gains in condition; and (iv) we account for interindividual physiological and behavioural differences. We use the elephant seals as a model system to understand changes in body condition. In the process of modelling condition in elephant seals, we explore three specific factors that may influence change in condition: (i) the effect of individual foraging location (Weimerskirch *et al*. 1997; Bradshaw *et al*. 2004; Simmons *et al*. 2007; Thums, Bradshaw & Hindell 2011); (ii) the effect of individual behaviour (Morales *et al*. 2004); and (iii) the effect of covariates (Hanks *et al*. 2011; Bestley *et al*. 2012; Breed *et al*. 2012). We describe within- and between-species differences in these processes, and we note how the temporal patterns of body condition change differ across individuals and species.

## Materials and methods

### Data

Female elephant seals have a relatively simple life-history pattern, which makes them a good system for studying changes in body condition. Adult females alternate two periods of time ashore at a breeding colony, with two extended trips to sea (Le Boeuf & Laws 1994). Here we focus on the longer of the two trips – the approximately 8-month trip taken following the annual moult. SES are on land in January for the moult, then at sea until from February through September, on land for pupping and breeding and then at sea again from November to early January (Hindell & Burton 1988). The cycle is similar for NES, but shifted owing to the different hemisphere (Le Boeuf & Laws 1994). NES are typically ashore for pupping and breeding in January and February, then at sea for the post-breeding trip, on land for the moult around April and May and back to sea for the remainder of the year (Le Boeuf & Laws 1994). Because of this repeated pattern, it is relatively easy to catch the animals before and after the extended trips to sea. At each capture, the animals can be weighed, measured and have their body fat recorded (Le Boeuf *et al*. 2000; Thums, Bradshaw & Hindell 2008a; Robinson *et al*. 2010).

**Figure 1:**
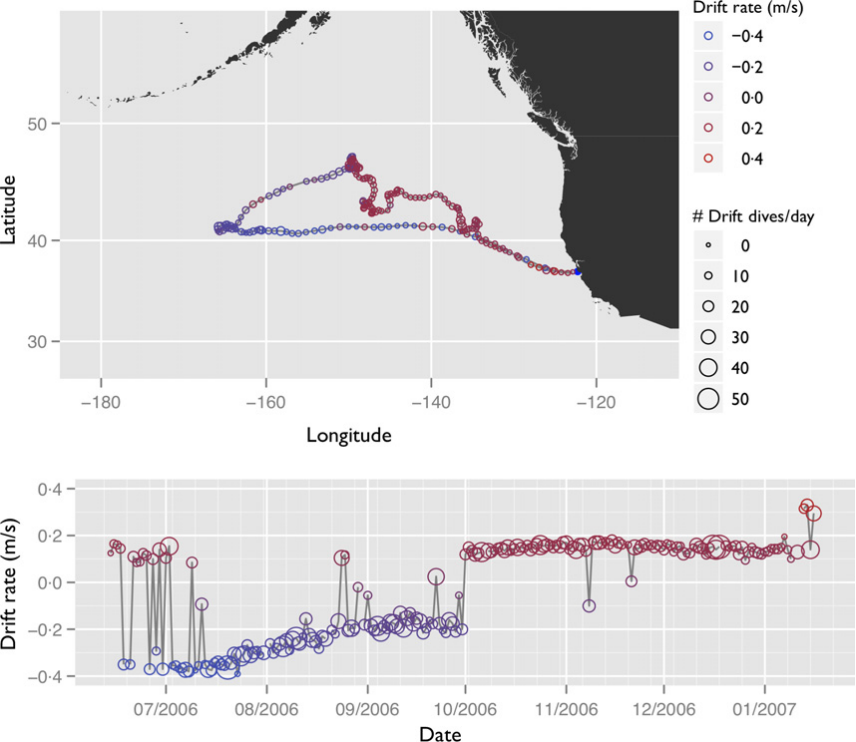
Foraging trip map and drift rate time series for one northern elephant seal (M583, Table 1) tagged at Ano Nuevo in 2006. The large shift to positive buoyancy occurs in early October 2006.

**Figure 2:**
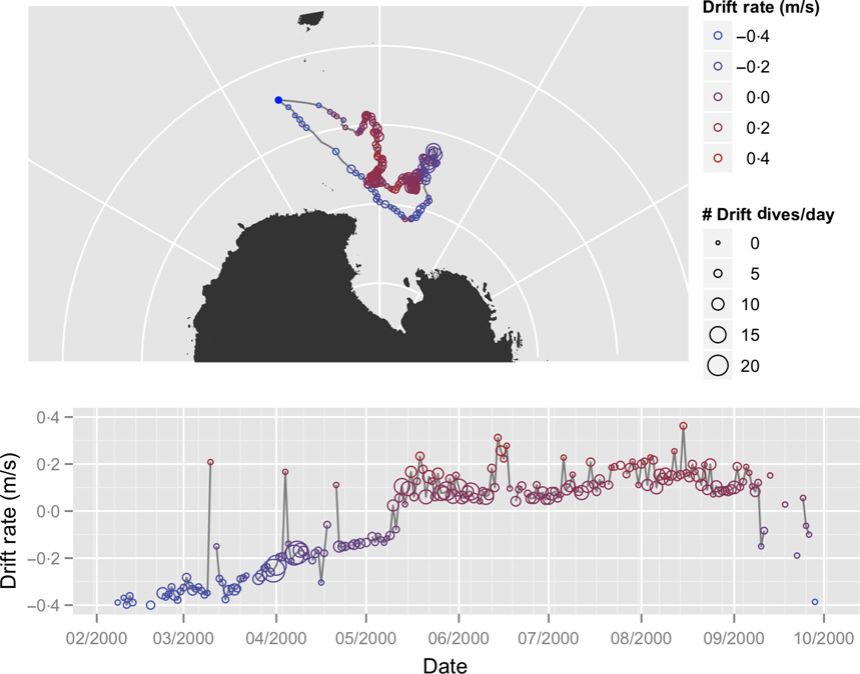
Sample track of one southern elephant seal tagged in 2000 (b889_pm). This animal forages in the ice edge or Ross Sea. She became positively buoyant in early to mid-May, remaining that way until the very end of her foraging trip. For display purposes, the data are projected into an Azimuthal Equidistant projection. Concentric lines of latitude are, from the pole outward, −80, −70, −60 and −50 S. Radial lines of longitude are, from left to right, 120 E, 150 E, 180 E, 150 W, 120 W. Colours and symbols are as in Fig. 1.

Twenty-nine NES were tagged at Año Nuevo, California, USA, from 2004 through 2007 (Figs 1 and S1.1 in Appendix S1, Table S2.1 in Appendix S2, Supporting information), and 30 SES were tagged at Macquarie Island in 2000, 2001, 2002, 2004 and 2005 (Figs 2 and S1.2 in Appendix S1, Table S2.2 in Appendix S2, Supporting information). At Año Nuevo, NES were equipped with ARGOS satellite transmitters (Wildlife Computers, Redmond, WA, USA or Sea Mammal Research Unit, St. Andrews, UK) and time-depth recorders (TDRs, Wildlife Computers MK9). Information on the dives was recorded every 8 s; information on the *x*, *y* position of the animal was linearly interpolated from filtered ARGOS data to provide locations at 8-hour intervals (Robinson *et al*. 2010). Dive information was extracted from the tags using a custom analysis programme (Robinson *et al*. 2010). Mass of the females at departure and arrival was measured by weighing the animals in a canvas sling suspended from a tripod, and their lipid stores were measured with portable ultrasound units (Robinson *et al*. 2010). In most cases, the mother is not weighed until 5 days after she gives birth; hence, the recorded mass at this time is likely lower than the true arrival mass. To get the arrival mass, we back-calculated the weight data with a linear regression (Thums, Bradshaw & Hindell 2008a; Robinson *et al*. 2010; Simmons *et al*. 2010).

At Macquarie, SES were equipped with TDRs (Wildlife Computers MK8). These provided data on time, depth, light level and revolutions of a flow-driven turbine every 30 s (Thums, Bradshaw & Hindell 2008a). Daily at-sea positions were calculated using geolocation software (Wildlife Computers, WC-GPE; Thums, Bradshaw & Hindell 2008a). Dive information was extracted from the tags and analysed using a custom dive analysis programme (‘DIVE,’ Stuart Greenhill, Murdoch University; Thums, Bradshaw & Hindell 2008a). These tags did not contain velocity measurements, and we acknowledge that using time-depth profiles in the absence of velocity data will inevitably result in some drift dives being missed or incorrectly identified. However, a validation of this classification technique found that misclassification of drift dives was only 2–4% (Thums, Bradshaw & Hindell 2008b). From the extracted and analysed dive data, individual dives were classified. For dives classified as drift dives, the speed through the water column was retained. For further details of the tagging process, and the use of the dive information to enumerate individual drift dives, see Robinson *et al*. (2010), and Thums, Bradshaw & Hindell (2008a,b).

Individual drift dives occur from 0 to *n* times per day with each drift dive having a drift rate. Since we modelled the lipid change process at a daily time step, we created a median daily drift rate (m sec^−1^) for each day for each animal. We also summed the number of daily drift dives and used the *x*, *y* locations of the animals to calculate daily transit (km) and a 5-day running average transit value (km).

**Figure 3:**
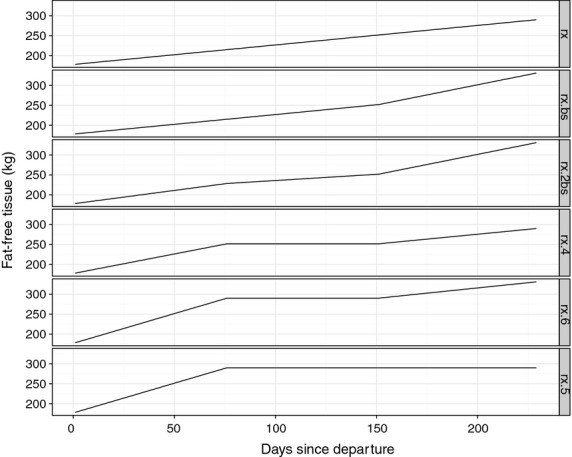
Six different assumptions we tested for the fixed fat-free tissue time series between the known initial measurement and the known final measurement. Top panel assumes a constantly linear increase between measurements. Second panel has a higher rate of increase in the final third of the trip to account for the weight of the pup. Third panel has higher initial gain, then slower, then higher again. Fourth panel is higher, flat and higher but unlike panels 2 and 3, does not account for the weight of the pup. Panel 5 is like panel 4, but does account for the weight of the pup. Finally, panel 6 – the assumption we used following model selection – assumes all gain in the first third of the trip.

Seal density is the product of four body components: bone (ash), body water, lipid and protein (Biuw *et al*. 2003), and we lack information on the relative at-sea proportions of each of these four components. Accordingly, we have fixed the non-lipid tissue (bone, body water and protein) and modelled just the lipids. Although we fixed the non-lipid tissue time series, and therefore did not estimate it, we did explore several different functional forms for the time series of non-lipid tissue (Fig. 3). Using model selection, we chose a functional form that places most of the gain of non-lipid tissue in the first third of the trip and flat thereafter (Fig. 3). We used the departure and arrival mass and lipid measurements of each female in each year in two ways. First we used these measurements to provide known start/end points to the non-lipid tissue time series (Fig. 3). Second, we used these measurements as fixed known data points in the lipid estimation process (see Model section below for more details).

### Covariates

We tested the influence of each of the three ecological factors (foraging location, behaviour and covariates) on body condition by placing covariates into the model. For factor 1, these included discrete covariates that indicated the macroscale foraging location of the animal. For factor 2, this included discrete covariates that indicated the behavioural state the animal was in at time *t*. Lastly, for factor 3, we included continuous covariates for environmental variables, as well as self-referential covariates for the animals' swimming and diving behaviour and physiological measurements.

#### Factor 1: foraging location

**Figure 4:**
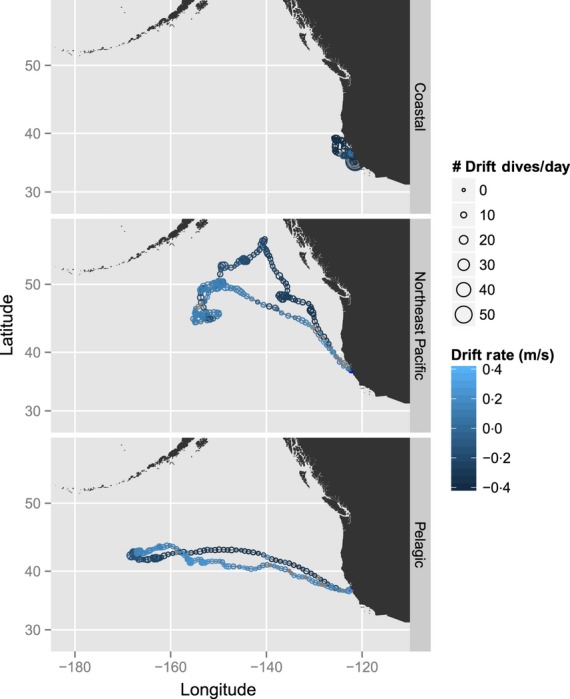
Three-panel plot depicting examples of the three different foraging locations in which northern elephant seals forage.

**Figure 5:**
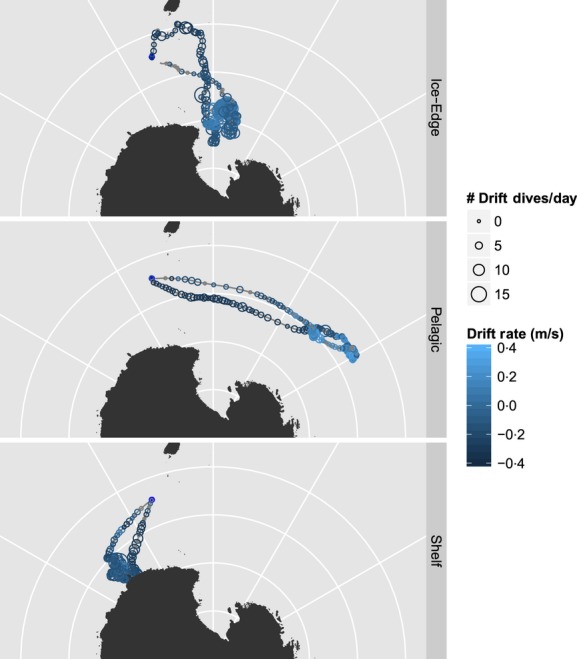
Three-panel plot depicting examples of the three different foraging locations in which southern elephant seals forage.

Each species had three different macroscale foraging locations: coastal, NE Pacific and transition zone for NES (Fig. 4); shelf, ice edge and pelagic for SES (Fig. 5). Our initial assumption was that shelf-associated NES and SES would put on lipids faster than the other strategies.

#### Factor 2: behavioural state

**Figure 6:**
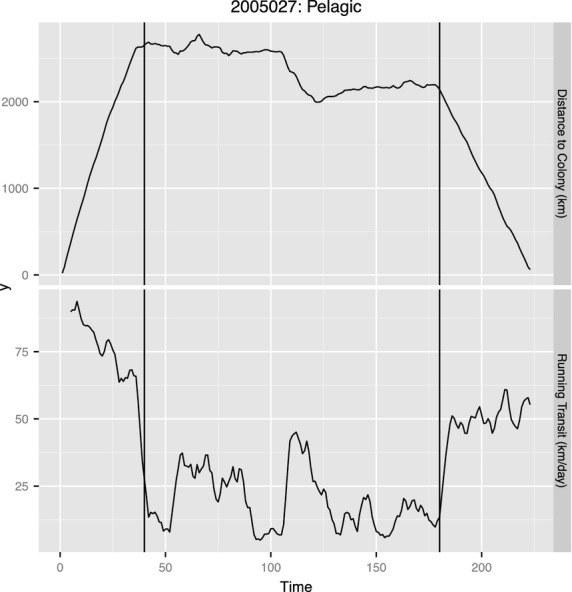
Two panels show the data used to delineate the tracks into the three long-term behavioural phases for one northern elephant seal (2005027). Top plot shows daily distance to colony (km); bottom plot shows 5-day running transit values (km). Vertical bars denote the transitions between the three states, which are defined as: transit away, foraging and return.

We included and tested measures of behavioural state that corresponded to behaviours at two different temporal scales. (Hereafter we use ‘phase’ to refer to the long time-scale behavioural state and ‘state’ to refer to the short time-scale behavioural state.) To create and assign the long time-scale measure of behavioural phase, we used a combination of daily distance to colony values and 5-day running transit values to split the track into three macroscale phases: transiting away from the colony, foraging and transiting back (Fig. 6). The transition points for each were assessed and assigned for each individual, that is, we did not use one threshold value for all seals. The range for transit values when animals shifted into the foraging phase was 25–40 km per day. The delineations between phases correspond approximately to each third of the track, although the last phase is typically the shortest. These delineations are done for each strategy and placed accordingly into the design matrix. Hence, ‘coastal foraging’ is a factor covariate that indicates the foraging phase for animals foraging in the coastal location. The second measure of behaviour was an estimate of behavioural state at a finer temporal scale. This measure partitioned daily locations into one of two categories, travelling or foraging (Jonsen, Flemming & Myers 2005). Each of these measures was placed as factor covariates in the design matrix (see Model Details section). Using either state or phase, our a priori assumption was that animals classified as being in a ‘foraging’ mode would put on lipids at a higher rate.

#### Factor 3: environmental and self-referential covariates

We measured environmental covariates for each mean daily position of the animal using ArcGIS 9·3® (ESRI, Redlands, CA, USA). For each species, we measured a variety of environmental covariates that we assumed had an impact on the rate at which individuals gain and lose lipids (Table S3.1 in Appendix S3, Supporting information). Initial exploratory data analysis with several different explicitly environmental covariates revealed few correlations between lipid gain and remotely sensed covariates. Accordingly we created several self-referential covariates: (i) # of drift dives per day; (ii) surface transit (km day^−1^); and (iii) three covariates governing physiological status of the female: daily ratio of lipid:lean tissue, departure lipid percentage and departure mass (kg).

### Model Details

We used a state-space model framework to describe the process of lipid change during a foraging trip and to quantify the relationship between observed drift rate and underlying lipid content. The two fundamental components of our model addressed (i) the link between the observations and the hidden process and (ii) the underlying process of lipid gain/loss. We constructed a hierarchical model, comprised of a data model for drift rate, a process model for lipid content and parameter models that incorporate prior knowledge about lipid gain.

### Data Model

Drift rate observations are linked to lipid status as: 

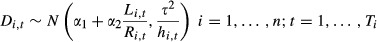
eqn 1where *D*_*i,t*_ is the median daily drift rate (m sec^−1^) of individual *i* on day *t*, *L*_*i,t*_ is the estimated daily lipid content (kg), *R*_*i,t*_ is the non-lipid tissue (kg), τ^2^ is the observation variance, which is scaled by *h*_*i,t*_ is the number of drift dives during the interval (*t*, *t *+* *1). This scaling indicates that the variance decreases with an increase in the number of drift dives observed each day. The *α* parameters in the observation model are for the intercept and the slope of linear relationship between lipid:lean ratio and the observed drift rates. Lipid content at the time when the animal leaves the colony *L*_*i,*0_ and upon return to the colony 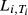 is known (Fig. S1.3 in Appendix S1, Supporting information); all other values for *L*_*i,t*_ are estimated together with model parameters. Non-lipid tissue *R*_*i,t*_ is also known at departure and upon return. The known birth mass of the pup provides information on the fraction of the returning mother's lean mass represented by the developing foetus (Fig. 3). We acknowledge that our assumption that drift rates are a linear function of the ratio of lipid:non-lipid tissue is a simplification of the many factors that likely influence observed drift rates. These factors include the depth at which the animal is diving, the salinity of the surrounding water, the volume and surface area of the animal and finally the drag coefficient of the animal (Biuw *et al*. 2003). We chose this functional form for several reasons. The first is parsimony in that we should make the model as simple as possible, especially since we lack information about at-sea volume, surface area and the drag coefficient. Second, from a computational standpoint, this formulation is much more efficient and allows for direct sampling from the conditional distribution. We explored a functional form that was similar to equation 9 from Biuw *et al*. (2003), but the model was unstable, especially when the sign of the difference between seal density and sea water density changed, that is when the data were fluctuating above and below 0. Third, effective simulation from the posterior indicates that this functional form captures the approximate behaviour of the system. Work is ongoing to extend this observation model.

### Process Model

Lipid change over time depends on the environment, individual differences and model error 

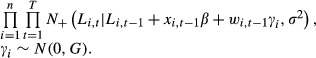
eqn 2

Lipid content, *L*_*t*+1_ conditioned upon lipid content at time *t* and covariates. The truncated normal density *N*_+_( · ) has non-negative values for positive *L*_*i,t*_ and zero otherwise. Environmental covariates are contained in the 1 by *p* design vector *x*_*i,t*_ a subset of which are included as *q *< *p* random effects *w*_*i,t*_ (Clark 2007). Population-level parameters *β* and random individual effects γ relate covariates to lipid gain. If lipid gain is influenced by an environmental covariate, then we would expect the credible interval for *β* to exclude 0. In the manuscript, when the 95% credible intervals for the parameter exclude 0, we will refer to this association as significant. The σ^2^ parameter represents the process error.

### Parameter Models and Priors

Prior distributions were specified to incorporate prior knowledge and to make efficient posterior simulation. The α parameters in the observation model have the prior: 

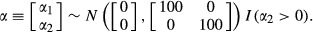
eqn 3

The indicator function *I*_+_( · ) means that the bivariate normal is truncated at zero for the slope parameter. This prior expresses the knowledge that drift rates increase, that is, become more positive, as lipid content increases.

Observation and model errors have informative inverse gamma prior distributions: 

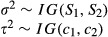
eqn 4with prior parameter values centred on 1 for the observation model and 4 for the process model. This means that after the variation in the observations has been accounted for in the observation model, there can be at most 0·1 m s^−1^ of unexplained error in the drift rates. Similarly, for the process error, this means that after the growth in lipids is accounted for, there can be at most 2 kg day^−1^ of unexplained error in the lipids. These priors are weighted proportional to sample sizes: 

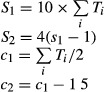
eqn 5

These mean values were chosen to match the scale of variation expected for uncertainty in drift rates (0·1 m s^−1^) and for the residual variation expected from the process model of lipid gain or loss (2 kg day^−1^). We lacked measurements on mass gain at sea, so we used on-land weight loss values to establish these mean values (Crocker *et al*. 2001). Although mass loss rates (kg day^−1^) may differ on land and at sea due to the differing energetic demands of these two phases of the seal's annual cycle, using an informed prior for τ^2^ is still more useful than a flat, uninformed prior. Prior distributions for fixed effects were flat, but in certain cases were truncated at zero to reflect prior knowledge of the sign of covariate effects *β* ∼ *N*(*b*,*B*)*I*(*b*_min_< *β *< *b*_max_). For example, we assumed that animals with higher numbers of drift dives will put on lipids at a higher rate, hence *b*_min_ = 0. The prior distribution on the random effects covariance matrix was inverse Wishart *G* ∼ *IW* (*R*, *r*) with prior covariance matrix *R = *diag(1,*q*) and non-informative degrees of freedom *r *= *q *+* *1.

### Computation, Model Selection and Diagnostics

We fit the model to data for both species using a Markov chain Monte Carlo (MCMC) technique involving a Metropolis step within a Gibbs sampler (Clark 2007). We conditioned the estimates of the lipids on known departure and arrival values of *L*_*i,t*_ as noted above. Each MCMC step involved Gibbs sampling of fixed effects, random effects and variances, and a Metropolis update of latent states *L*_*i,t*_. We used model selection to determine the importance of input variables, random effects and prior distributions. Model selection was based on the marginal likelihood, approximated using the approach of Chib (1995). Following model fitting and selection, we arrived at a ‘final model’ that included significant parameters governing the lipid gain process. (See Appendix S3 (Supporting information) for a full listing of the different covariates we explored in the modelling process.)

To evaluate the model and algorithm, we determined the capacity to recover known values from input data. Because the model provides estimates of the missing data, we compared estimates to true data by artificially creating missing values in the observed drift data and in the environmental covariates. Estimates of the missing data were very good for the drift dive data, that is, the BCI covered the true value (Fig. S3.2 in Appendix S3, Supporting information), and for the missing covariate data (Fig. S3.3 in Appendix S3, Supporting information). Appendix S3 (Supporting information) contains a full exposition of the Gibbs sampler, details on model selection and a description of the model fit.

## Results

**Figure 7:**
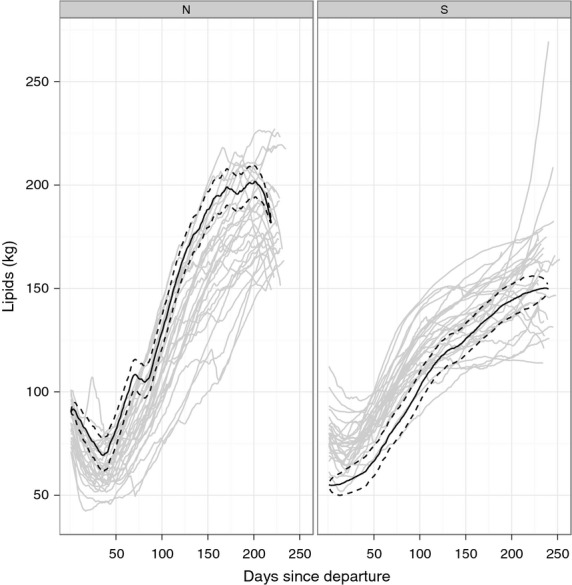
Posterior estimates of lipid gain (grey lines) in all northern elephant seals (left panel) and all southern elephant seals (right panel). Black lines depict one northern elephant seal (M583, as in Fig. 1) and one southern elephant seal (b889_pm, as in Fig. 2). Solid line represents the posterior daily mean lipid content in the animal; dashed lines represent ±1 standard deviation away from the mean.

**Figure 8:**
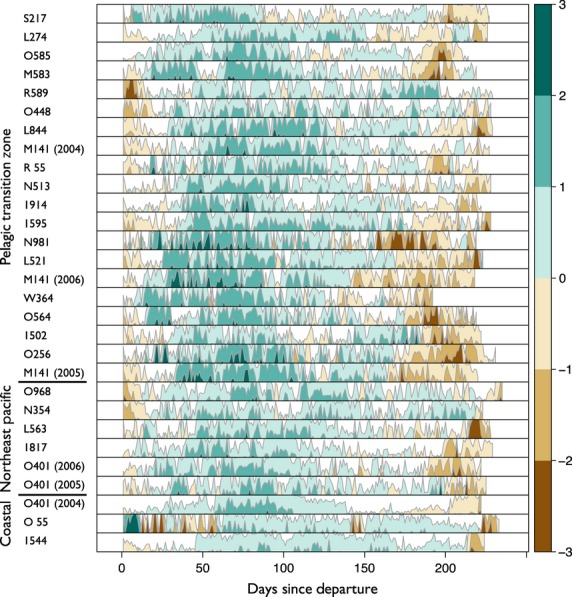
A horizon plot depicting daily lipid gain (blue) and loss (red) over the post-moult foraging trip for 29 northern elephant seals (*Mirounga angustirostris*) from the Año Nuevo colony. This plot shows gain and loss as filled areas on the same positive ordinate, with colour depicting the direction of the change. The filled areas are sliced into three equal levels (the colour bar) with the highest and lowest values of gain and loss shown in the most saturated colours. The magnitude of lipid gain/loss is shown with increasingly saturated colours and is scaled equivalently across individuals. The three horizontal bars on the left denote which animals used which foraging location: (i) transition zone; (ii) NE Pacific; and (iii) coastal. Within each foraging location, the animals are ordered based on departure lipid percentage, with leanest animals at the top and fattest animals at the bottom. Animals that foraged in the coastal waters put on less lipids than animals foraging in either transition zone or NE Pacific. Animals with a higher departure lipid percentage upon departure put on lipids faster.

**Figure 9:**
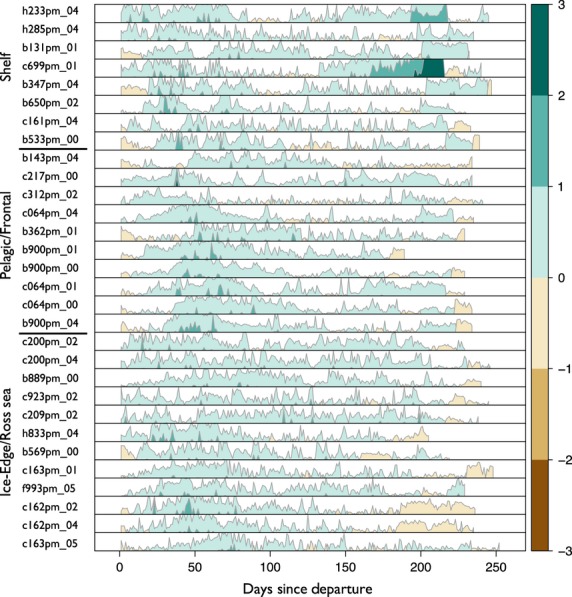
A horizon plot as in Fig. 7 depicting daily lipid gain (blue) and loss (red) over the post-moult foraging trip for 30 southern elephant seals (*Mirounga leonina*) from the Macquarie Island colony. The three horizontal bars on the left denote which animals used which foraging location: (i) animals that went to the Antarctic shelf; (ii) animals that foraged in the pelagic zone to the south and east of Macquarie; and (iii) animals that foraged at the ice edge of the Ross Sea. Within each foraging location, the animals are ordered based on departure lipid percentage, with leanest animals at the top and fattest animals at the bottom. Lean shelf-associated animals put on lipids for a longer duration than fatter animals foraging in the same location. Pelagic animals ranged the farthest from the colony and gained lipids for a sustained period. Ice edge animals gained throughout their trip, but the gain was much more varied across animals.

The pattern of lipid gain in NES consisted of an initial period of decline in lipids, rapid lipid gain during the foraging phase and then subsequent loss during the return trip (Fig. 7). With the exception of one coastal animal (0 55), all NES followed this pattern (Figs 7 and 8). In contrast, SES lost relatively little lipids early in the post-moult trip; instead animals gained throughout the course of their trip (Fig. 7). NES lost more lipids initially (Figs 7 and 8), but gained more absolute lipid than SES (Figs 7 and 9). Detailed graphical results for each individual can be found in Appendix S4 (NES) and Appendix S5 (SES) (Supporting information).

At the species level, the final model retained after model selection for NES and SES was identical save for one covariate (Table 1). The final model for each species included parameters for the intercept term, mean daily transit, number of drift dives, lipid:lean ratio, foraging location, behavioural phase and departure lipid percentage. The final model for NES also included the short-term behavioural state (Table 1). Parameter estimates for the two species were similar for the observation model (Table 1).

**Table 1:** Posterior estimates and 95% Bayesian credible intervals for *α* and *β* parameters for each species of elephant seals. For the interaction terms between foraging location and behavioural phase, there are nine possible interactions. Here eight are listed, and the nineth is the reference, that is, the reference for pelagic: phase 1, equals *β*_reference_ + *β*_*P*:*P*1_. For NES, the reference corresponds to the animals foraging in the coastal zone during the foraging phase. For SES, the reference corresponds to animals foraging in the pelagic zone during the foraging phase. Intercept is the *β*_0_ term in the process model for lipid change

Species	Parameter	Mean	0·025%	0·975%
Northern	*α*_1_	−0·578	−0·697	−0·461
	*α*_2_	1·214	0·964	1·469
	Intercept	−2·32	−4·644	.0124
	Transit	−0·035	−0·06	−0·01
	# Drift dives	0·067	0·0	0·159
	Lipid: lean ratio	−2·197	−3·155	−1·365
	Pelagic: phase 1	0·902	−0·237	2·013
	Pelagic: phase 2	1·074	0·366	1·782
	Pelagic: phase 3	0·521	−0·489	1·508
	NE Pacific: phase 1	0·146	−1·446	1·846
	NE Pacific: phase 2	0·561	−0·323	1·429
	NE Pacific: phase 3	1·149	−0·559	2·816
	Coastal: phase 1	−0·925	−2·117	0·206
	Coastal: phase 3	−0·047	−1·283	1·181
	Departure lipid (%)	0·114	0·055	0·172
	State Index	0·433	−0·108	0·978
Southern	*α*_1_	−0·561	−0·695	−0·436
	*α*_2_	1·332	1·003	1·681
	Intercept	1·981	0. 753	3·215
	Transit	−0·374	−0·695	−0·081
	# Drift dives	0·071	0·012	0·133
	Lipid: lean ratio	−2·240	−2·826	−1·585
	Ice edge: phase 1	0·029	−0·413	0·491
	Ice edge: phase 2	−0·039	−0·346	0·271
	Ice edge: phase 3	−0·234	−0·802	0·309
	Shelf: phase 1	−0·115	−0·663	0·443
	Shelf: phase 2	−0·165	−0·487	0·159
	Shelf: phase 3	0·591	−0·028	1·237
	Pelagic: phase 1	0·079	−0·337	0·583
	Pelagic: phase 3	0·020	−0·580	0·583
	Departure lipid (%)	0·011	−0·018	0·039

NES, northern elephant seals; SES, southern elephant seals.

Northern elephant seals foraging in either the pelagic transition zone or the NE Pacific had similar lipid gain patterns, with pelagic animals putting on lipids at a higher rate (Table 1, Fig. 8). In particular, pelagic animals during the foraging phase put on lipids at the highest rate, *β *= 1·074 (0·37, 1·78) (Table 1, Fig. 8), followed by NE Pacific animals *β* = 0·561(−0·32, 1·43) (Table 1, Fig. 8). Animals foraging near the coast put on lipids more slowly (Table 1, Fig. 8). During the transit-away phase, pelagic animals put on lipids at the highest rate, followed by NE Pacific animals and coastal animals (Table 1, Fig. 8), suggesting perhaps that pelagic animals are encountering more prey on their route away from the colony. For SES, the return phase for animals foraging on the shelf is the state of highest lipid gain, followed by the foraging phase for pelagic animals (Table 1). The return phase for both the ice edge and pelagic strategies was costly; animals in this phase tended to lose lipids (Table 1).

Finally, for NES only, the parameter for the shorter-term behavioural state was significant and positive (Table 1), suggesting that the locations where animals are estimated to be in a foraging state (Jonsen, Flemming & Myers 2005) are locations where animals consistently put on more lipids. We included this model parameter in previous versions of the SES model, but the estimates were always near 0.

The relationship between the number of drift dives and lipid gain was positive and similar in magnitude for each species; however, the relationship between transit and lipid gain was stronger for SES (Table 1). This means that increased daily transit rates resulted in a comparably smaller lipid gain in SES (Table 1). None of the relationships between environmental covariates and lipid gain were different from 0 for NES or SES.

For the self-referential covariates, lipid gain in NES and SES depended on physiological status, that is, departure lipid percentage and lipid:lean ratio (Table 1). The relationship between departure lipid percentage and lipid gain for NES was positive, significant and fourfold higher than SES (Table 1). This suggests that animals that were fatter prior to the migration gained more lipids than leaner ones and is consistent with, although certainly does not prove, the hypothesis that leaner animals would initially focus on structural repair of lean tissue (Fedak, Arnbom & Boyd 1996; Crocker *et al*. 2001).

### Random Effects

As modelled here, no strong individual response was noted for the intercept, lipid:lean ratio or transit in either species (Table S2.3, Table S2.4 in Appendix S2, Supporting information). The fact that these random effects were close to 0 means either that individuals are similar in their responses or that much larger data sets would be required to infer individual differences.

## Discussion

We have estimated the daily lipid gain process in individual elephant seals across two different species. We have taken advantage of the unique drift diving behaviour in elephant seals together with a modelling approach that incorporates uncertainty in both the observations and the process to provide insight into how individuals are gaining and losing condition in their environment. By doing so, we have been able to quantitatively determine how foraging location affects body condition. This allows us to see profitable areas in time and space and understand how exactly lipid gain differs among animals employing these different locations.

### Factor #1: Influence of Foraging Location on Condition

Each model supported the inclusion of the factor covariates indicating three different foraging locations. For NES, non-coastal animals put on lipids at a higher rate than those who forage near the coasts (Fig. 8, Table 1). This contrasts with previous findings (Simmons *et al*. 2007), but could simply be an artefact of small sample size. Compared to coastal seals, animals that forage in the pelagic transition zone have to travel farther to reach destinations with presumably higher prey concentration; yet despite this cost, these animals consistently put on lipids at a higher rate (Fig. 8, Table 1).

The three foraging locations seen in SES are more geographically distinct than NES (Fig. 5); however, the pattern of lipid gain is not dramatically different in the three foraging locations (Fig. 9). In contrast to NES shelf females, shelf females in the return phase put on lipids at the highest rate (Table 1, Fig. 8). Although our analysis and classification methods were different from Thums, Bradshaw & Hindell (2011), the results were consistent – namely that shelf foraging habitats are favourable areas for lipid gain. Of particular note is that the foraging phase for pelagic animals appears to be better than for shelf animals, yet the return phase for shelf animals is the highest (Table 1). Part of this may be due to the fact that the return phase for these animals differs markedly (Appendix S5, Supporting information). Pelagic animals have on average 2240 km to cover to return to Macquarie, while shelf animals have on average 1360 km. Pelagic animals average 86 km per day in the return phase, while shelf animals average 60 km per day. Thus, shelf animals may have more foraging opportunities on the return trip as they are not forced to go as far or as fast. Another plausible hypothesis is that because the energetic demands are lower, the animals simply do not have to burn as much lipid. The coastal environment that each species forages in is very different. The inclusion of more coastal NES would help infer whether the apparent differences in the effect of foraging location in NES, that is, coastal vs. the other two locations, and the interspecies difference, that is, coastal SES putting on lipids at a relatively higher rate than coastal NES, are real. Finally, it has been recently suggested that for SES at îles Kerguelen, there exists a trade-off between favourable habitat and predation risk, that is, females may do better on the shelf but have a higher predation risk there (Authier *et al*. 2012). For SES at Macquarie, it is not clear how predation risk factors into commitment to a long-term foraging strategy, although Thums, Bradshaw & Hindell (2011) also suggested predation risk may temper how long females forage in the shelf region. Our results for lipid gain in shelf animals are consistent with this hypothesis. Of the three foraging locations, shelf animals are the leanest upon departure from Macquarie (20·77% lipid). Several SES females that forage on the shelf initially put on lipids while they are close to Antarctica and then depart and put on lipids in shelf habitat closer to Macquarie. These females include h233pm_04, b131pm_01, c699pm_01 and b347pm_04 (Fig. 9, Appendix S5, Supporting information). We propose that shelf animals go to the shelf near Antarctica first because it is profitable and presents less predation risk than foraging near Macquarie. As the ice encroaches and females are excluded from these foraging grounds (Bailleul *et al*. 2007b), they return to shelf waters near Macquarie to continue foraging (Appendix S5, Supporting information). However, since they are now bigger, they may face less predation risk than if they were to forage near Macquarie immediately after departing.

### Factor #2: Behavioural State

We included two proxies for behaviour state, one shorter-term behavioural state (Jonsen, Flemming & Myers 2005) and one longer-term behavioural phase. Results offered support for inclusion of the shorter-term covariate for NES (Table 1), but not for SES. This suggests that, at least in NES, it is possible to use proxies of surface movement to assess areas of positive gain in condition. For SES, it appears that some additional measure of behavioural activity at depth (Bailleul *et al*. 2008; McClintock *et al*. 2013) may be necessary to characterize short-term state differences. Additionally, the differences in data collection, that is, the poorer spatial resolution of the geolocation estimates used for SES, may mask some of the shorter-term behavioural patterns seen in NES.

The longer-term behavioural phase was significant for SES and NES (Table 1). That rates of gain and loss differ as a function of behaviour is not in itself surprising. However, there are three aspects of these results that are of note. First, the relative differences between the coastal animals across the species are interesting because the foraging ground for SES is influenced by ice cover (Bailleul *et al*. 2007b) and is projected to change as a function of global climate change (Ainley *et al*. 2010). This indicates that while coastal SES do well at present, they may be vulnerable to future change that alters the dynamics of this foraging location. Second, the fact that pelagic animals put on lipids at a high rate has potential role in our understanding of disturbance. We know seals stay out to sea longer in El Niño years (Crocker *et al*. 2006). Because these animals have farther to go (Figs 4 and 5), and because they lose lipids at the highest rate in the return phase (Figs 8 and 9), these animals may in fact be most influenced by a lack of prey in their preferred foraging grounds. These animals have travelled far to reach a putative foraging ground, presumably based on past experience (McConnell *et al*. 2002; Bradshaw *et al*. 2004), and if because of disturbance they are unable to put on lipids as quickly as in normal years, their condition may suffer most. Third, there is evidence that elephant seals can preferentially allocate resources to different tissue types (Condit & Ortiz 1987; Crocker *et al*. 2001). Seals that go to different foraging locations may be allocating resources at different times. Seals in the transit-away phase (Figs 8 and 9) may be burning fat reserves to reach their foraging location, but in addition, they may also be allocating newly acquired resources to structural repair of catabolized lean tissue.

### Factor #3: Environmental and Self-Referential Covariates

None of the environmental covariates were significantly linked to lipid gain in individual elephant seals. While this was initially surprising, it is consistent with organism–environment interactions in other systems (e.g. Hanks *et al*. (2011)). There are several likely explanations for the lack of a significant relationship. First, it is possible that we have chosen the wrong set of explanatory covariates. We know elephant seals are feeding on fish and squid (Bradshaw *et al*. 2003; Hindell *et al*. 2003), yet we lack data on the distribution of their prey. While we tested proxies for ocean productivity, it is plausible that the proxies are too distant in space, time and trophic status to explain the lipid gain process. Work in bluefin tuna has shown that lagging these variables can significantly improve correlation between covariates and fish abundance (Walli 2007); though computationally difficult, we presume that similar approaches in elephant seals would be informative. Second, it is possible that the temporal scale of the analysis could be refined with further work. Elephant seals are foraging on the order of metres, while the covariates used here vary on the scale of kilometres. Hence, there is an inherent mismatch between the two. In addition, it is possible that the change in lipids in response to the environment could manifest on different time-scales. For example, we have modelled lipids at a daily time step, but it is possible that the state evolves instead over weekly time steps. Finally, for SES at least, it is possible that the coarser spatial resolution of the location estimates hinders precise inference on the relationship between lipid gain and environmental covariates. These are areas for future research.

While the environmental covariates were not significant, it is possible that animals have spatial memory of past successful foraging areas. In this case, their distance and bearing to past successful areas might prove to be the important covariate, rather than *in situ* covariates like sea surface temperature. Although we did not test this hypothesis, we were able to track several individuals across multiple years (4 SES, 2 NES tagged in multiple years): in five of the six cases, the animals visit the same foraging location in multiple years; in most cases, the tracks of the animals in subsequent years can be plotted almost directly upon one another (Figs S1.5–S1.11 in Appendix S1, Supporting information). This is an area of current research.

Southern elephant seals are both bigger and leaner, than NES (Fig. S1.3 in Appendix S1, Supporting information); this explains why the β parameter for the lipid:lean ratio was stronger and more negative for SES (Table 1). Because SES are larger, they may have higher energetic costs to travel and to dive; these costs may be reflected in lower rates of gain (Fig. 9). Recent work in both NES and SES has shown that leaner animals have to work harder as they dive (Aoki *et al*. 2011; Miller *et al*. 2012). Thus, the relative leanness of individuals may be more important than absolute size. Altering leanness in SES with buoyancy experiments sensu, Aoki *et al*. (2011) may help explain the observed differences in species and highlight, from a bioenergetics standpoint, whether SES are indeed working harder throughout their dives. It is possible that, rather than mere physiological differences, SES are simply faring worse in gaining body condition. As mentioned in the Introduction, this has implications for understanding the trajectories of each species. McMahon *et al*. (2005) noted that large-scale environmental change over the second half of last century may explain the decline in some populations of SES. Our results placed in the context of the recent work on cost of swimming suggest areas of future research and comparison in these two species. At a minimum, long-scale monitoring is needed to see how females of each species fare in different oceanic conditions in each hemisphere.

While departure condition – as expressed by departure lipid percentage – did have a significant positive correlation with lipid gain process in elephant seals (Table 1), it is unclear whether departure status in elephant seals impacts the choice of foraging location. Body condition at departure in other systems has been shown to have a profound effect on foraging location (Chastel, Weimerskirch & Jouventin 1995; Weimerskirch *et al*. 1997; Weimerskirch 1998; Schmaljohann & Naef-Daenzer 2011). For example, in different species of pelagic seabirds where the adults provision a chick, individual body condition triggers the switch between two different types of foraging excursions (Weimerskirch *et al*. 1997; Weimerskirch 1998). While elephant seals do not employ such switching behaviour, it has been shown that condition can impact both the length and the nature of the post-moult foraging trip (Crocker *et al*. 2006). In these El Niño years, the animals are in poorer condition and remain away from the colony for longer periods of time in search of prey (Crocker *et al*. 2006).

## Conclusion

While the observation model employed here took advantage of the drift diving behaviour unique to elephant seals, there exist other proxies for buoyancy in many other systems. For example, many marine animals employ a stroke-and-glide swimming pattern. This pattern can be successfully extracted from portions of dive records in sperm whales and in elephant seals (Miller *et al*. 2004; Thums, Bradshaw & Hindell 2008a; Aoki *et al*. 2011). The use of tag systems with appropriate sensor suites in different species could provide us with additional opportunities to harvest these proxies for buoyancy and hence condition (Aoki *et al*. 2011). Although this would require changes to the observation model used here, it would provide many additional opportunities to examine how condition changes over time and space.

Although quantifying how individuals make movement choices in response to their landscape can lead to an understanding of landscape perception and habitat suitability, there have been few attempts to quantify how this interaction leads to changes in an individual's condition at fine temporal scales [though, see examples in Weimerskirch *et al*. (1997); Bailleul *et al*. (2007a); and Dragon *et al*. (2012)]. We have shown a way to estimate body condition in individual elephant seals at fine time-scales; results from this effort have allowed us to infer the areas and times in which these top predators profitably exploit their environment and choose to allocate resources to lipid stores. This has shown us the effect of foraging location, behavioural states and physiological status on the lipid gain process within and between species. This effort takes into account multiple sources of uncertainty and offers insight into a hidden process that reveals much about how marine predators successfully acquire resources. Although the results presented herein are specific to elephant seals, the model can be readily extended to other species – both marine and terrestrial – and represents a new trajectory in the study of body condition.

## Supporting Information

Appendix S1 **Figure S1.1.** Three example tracks from the northern elephant seal dataset illustrating examples of the three different foraging strategies: coastal, northeast Pacific, pelagic transition zone. **Figure S1.2.** Three example tracks from the southern elephant seal dataset illustrating examples of the three different foraging strategies: frontal (pelagic), ice-edge (Ross Sea), and shelf. **Figure S1.3.** Summary physiological information for northern elephant seals (top row) and southern elephant seals (bottom row). **Figure S1.4.** Daily drift dive by time aggregated across individuals for southerns (left panels) and northerns (right panels). **Figure S1.5.** Foraging trip by animal M141 in three separate years – 2004, 2005, and 2006. **Figure S1.6.** Foraging trip by animal O401 in three separate years – 2004, 2005, and 2006. **Figure S1.7.** Foraging trip by animal c200 in two separate years – 2002, and 2004. **Figure S1.8.** Foraging trip by animal c163 in two separate years – 2001, and 2005. **Figure S1.9.** Foraging trip by animal c162 in two separate years – 2002, and 2004. **Figure S1.10.** Foraging trip by animal b900 in three separate years – 2000, 2001, and 2004. **Figure S1.11.** Foraging trip by animal c064 in three separate years – 2000, 2001, and 2004.

Appendix S2 **Table S1.** Summary information for 29 northern elephant seals. **Table S2.** Sunmary information for 30 southern elephant seals. **Table S3.** Mean posterior estimate of the covariance matrix for random effects (intercept, lipid to lean ratio, and transit) for Northern elephant seals. **Table S4.** Mean posterior estimate of the covariance matrix for random effects (intercept, lipid to lean ratio, and transit) for Southern elephant seals.

**Appendix S3.** Further details on: (1) how the Gibbs sampler was constructed to fit the model to the data; (2) the fit of the model to simulated data; and (3) details on the different covariates included in the model. **Appendix S4.** Complete plots for each individual Northern elephant seal used in the analyses. Four plots per individual show: the overview map, the start and stop lipid percentage, the drift rate time series, and the time series of lipid estimates.

Appendix S5 Complete plots for each individual Southern elephant seal used in the analyses. Four plots per individual show: the overview map, the start and stop lipid percentage, the drift rate time series, and the time series of lipid estimates.
