# Supplementary material for: Estimating resource acquisition and at-sea body condition of a marine predator
Source: J Anim Ecol. 2013 Jul 19;82(6):1300–15. doi: 10.1111/1365-2656.12102 (PMC4028992; doi:10.1111/1365-2656.12102)
Supplement: Appendix S1 — Figure S1.1. Three example tracks from the northern elephant seal dataset illustrating examples of the three different foraging strategies: coastal, northeast Pacific, pelagic transition zone. Figure S1.2. Three example tracks from the southern elephant seal dataset illustrating examples of the three different foraging strategies: frontal (pelagic), ice-edge (Ross Sea), and shelf. Figure S1.3. Summary physiological information for northern elephant seals (top row) and southern elephant seals (bottom row). Figure S1.4. Daily drift dive by time aggregated across individuals for southerns (left panels) and northerns (right panels). Figure S1.5. Foraging trip by animal M141 in three separate years – 2004, 2005, and 2006. Figure S1.6. Foraging trip by animal O401 in three separate years – 2004, 2005, and 2006. Figure S1.7. Foraging trip by animal c200 in two separate years – 2002, and 2004. Figure S1.8. Foraging trip by animal c163 in two separate years – 2001, and 2005. Figure S1.9. Foraging trip by animal c162 in two separate years – 2002, and 2004. Figure S1.10. Foraging trip by animal b900 in three separate years – 2000, 2001, and 2004. Figure S1.11. Foraging trip by animal c064 in three separate years – 2000, 2001, and 2004. [file jane0082-1300-sd1.doc]

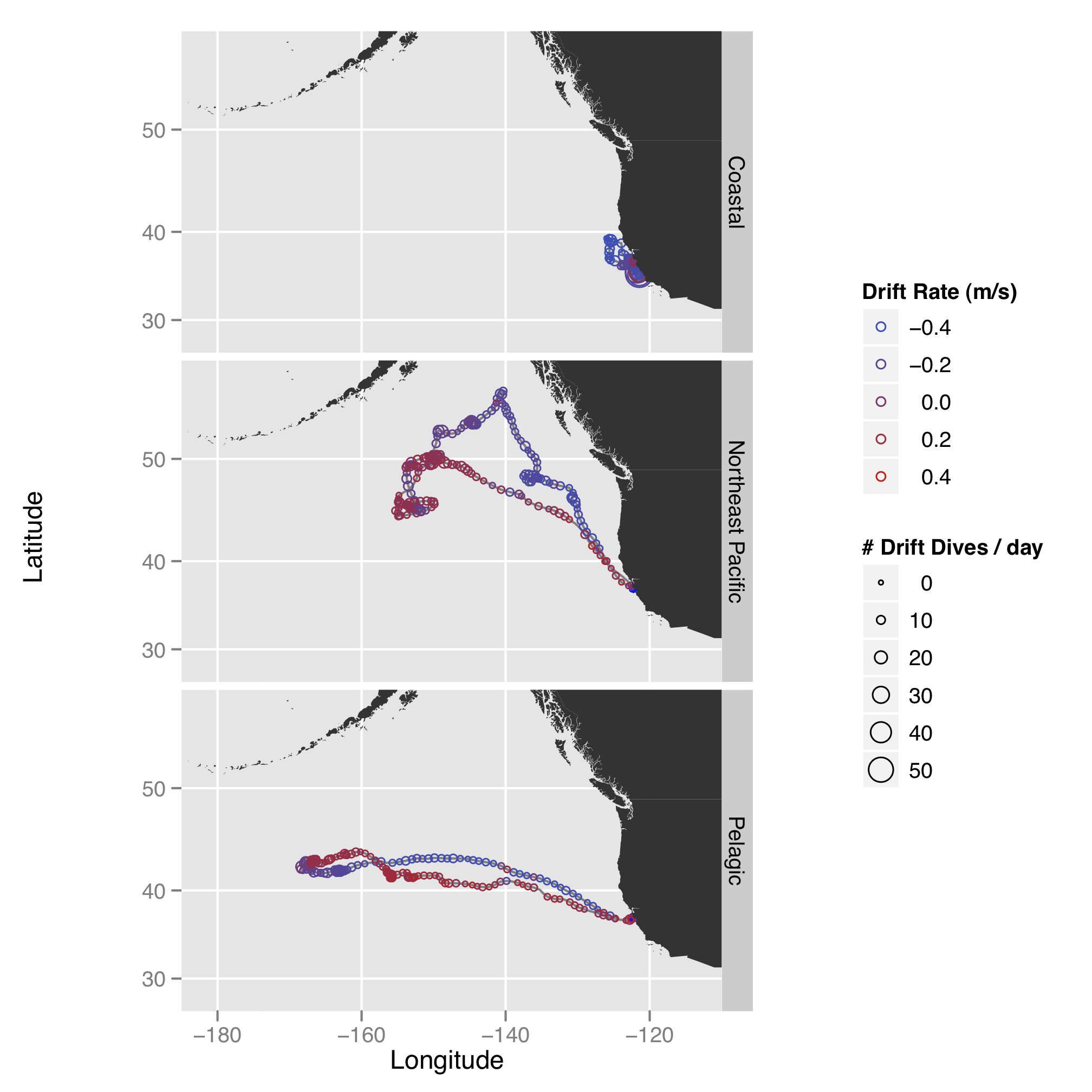


Figure S1.1. Three example tracks from the northern elephant seal dataset illustrating examples of the three different foraging strategies: coastal, northeast Pacific, pelagic transition zone. Note that the Coastal strategy can often include animals that range farther north along the California, Oregon, Washington, and British Columbia coasts.


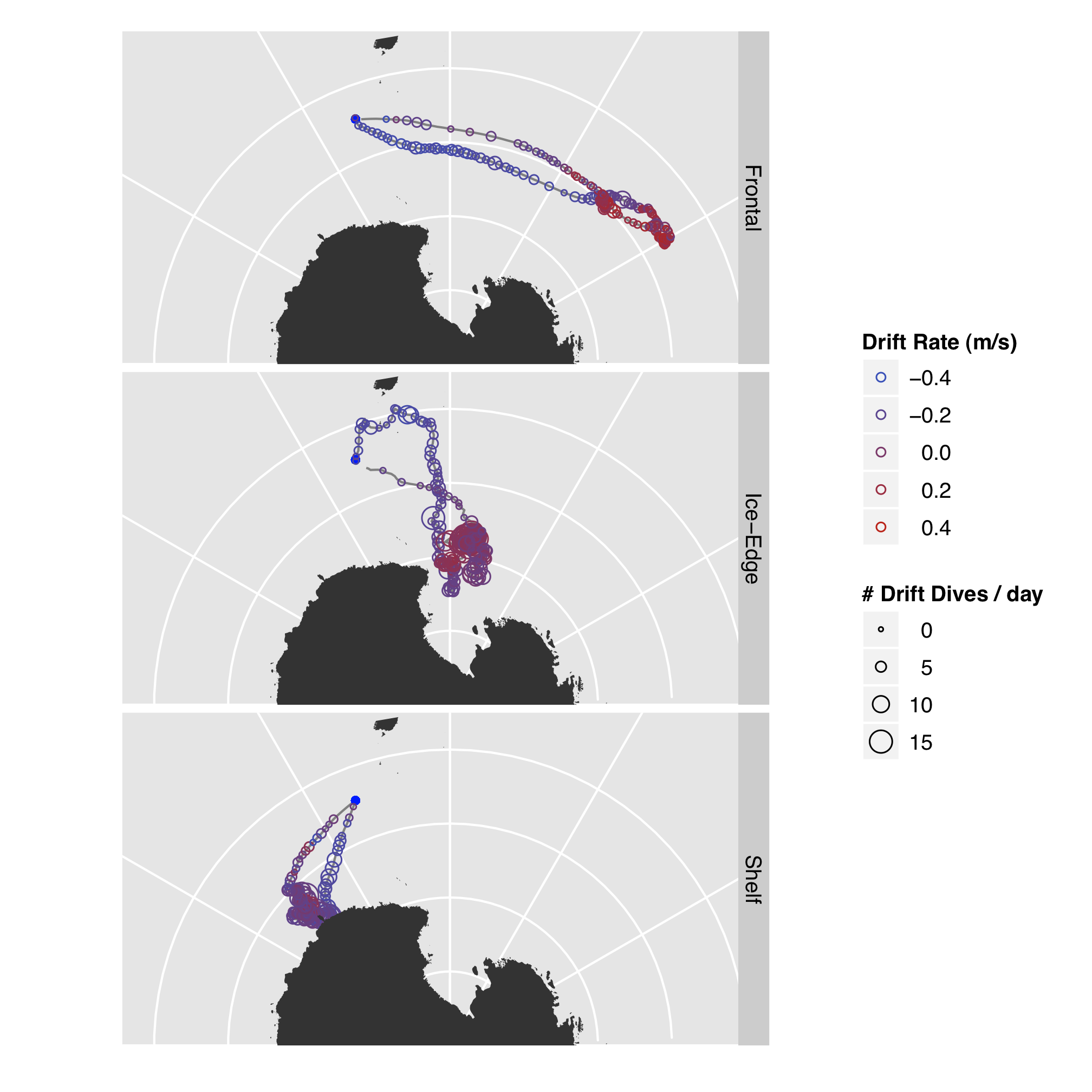


Figure S1.2. Three example tracks from the southern elephant seal dataset illustrating examples of the three different foraging strategies: frontal (pelagic), ice-edge (Ross Sea), and shelf.


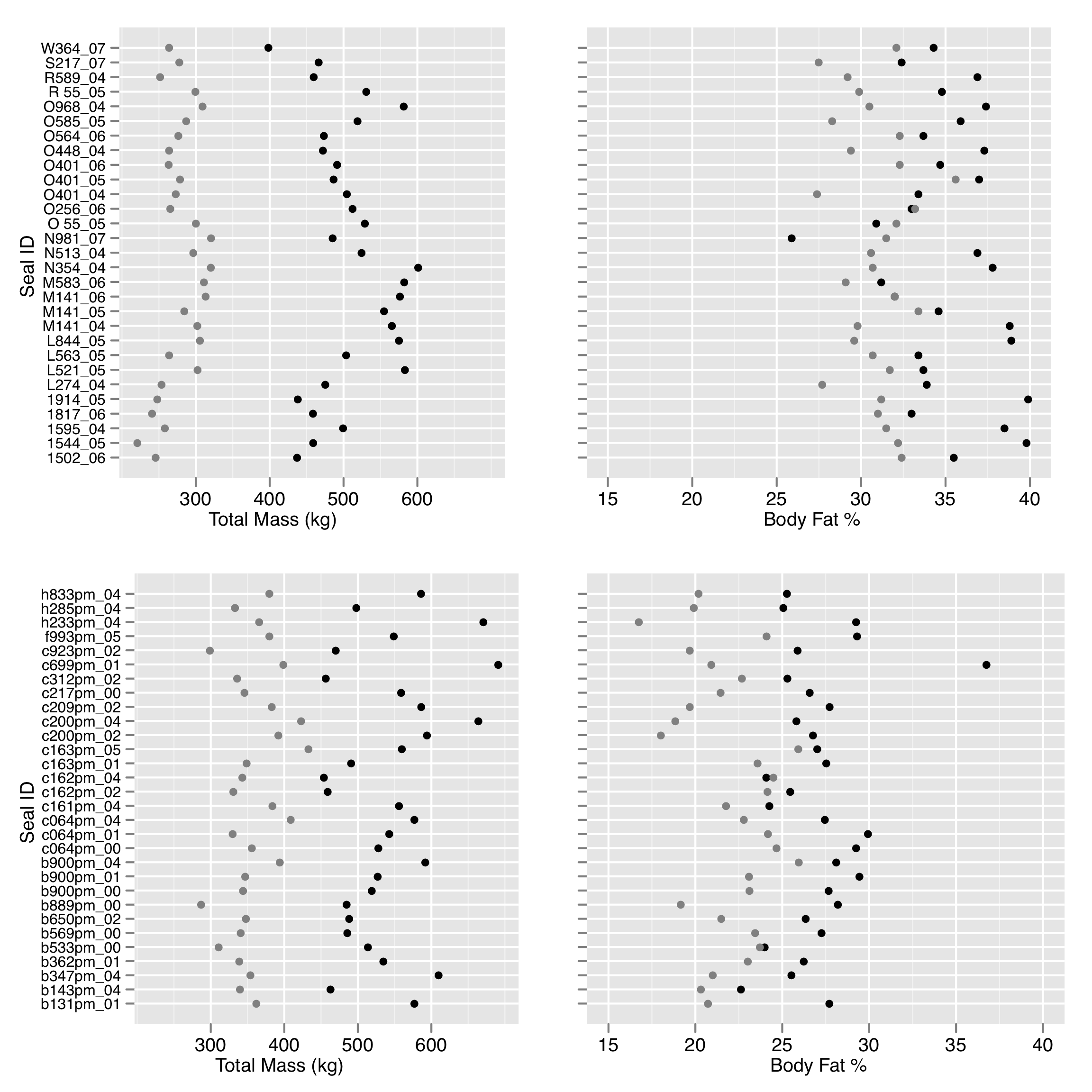


Figure S1.3. Summary physiological information for northern elephant seals (top row) and southern elephant seals (bottom row). Left panels depict departure and arrival mass (kg) in grey and black, respectively. Right panels depicts departure and arrival lipid % in the same fashion. Southern elephant seals are considerably larger but leaner as compared to northern elephant seals.


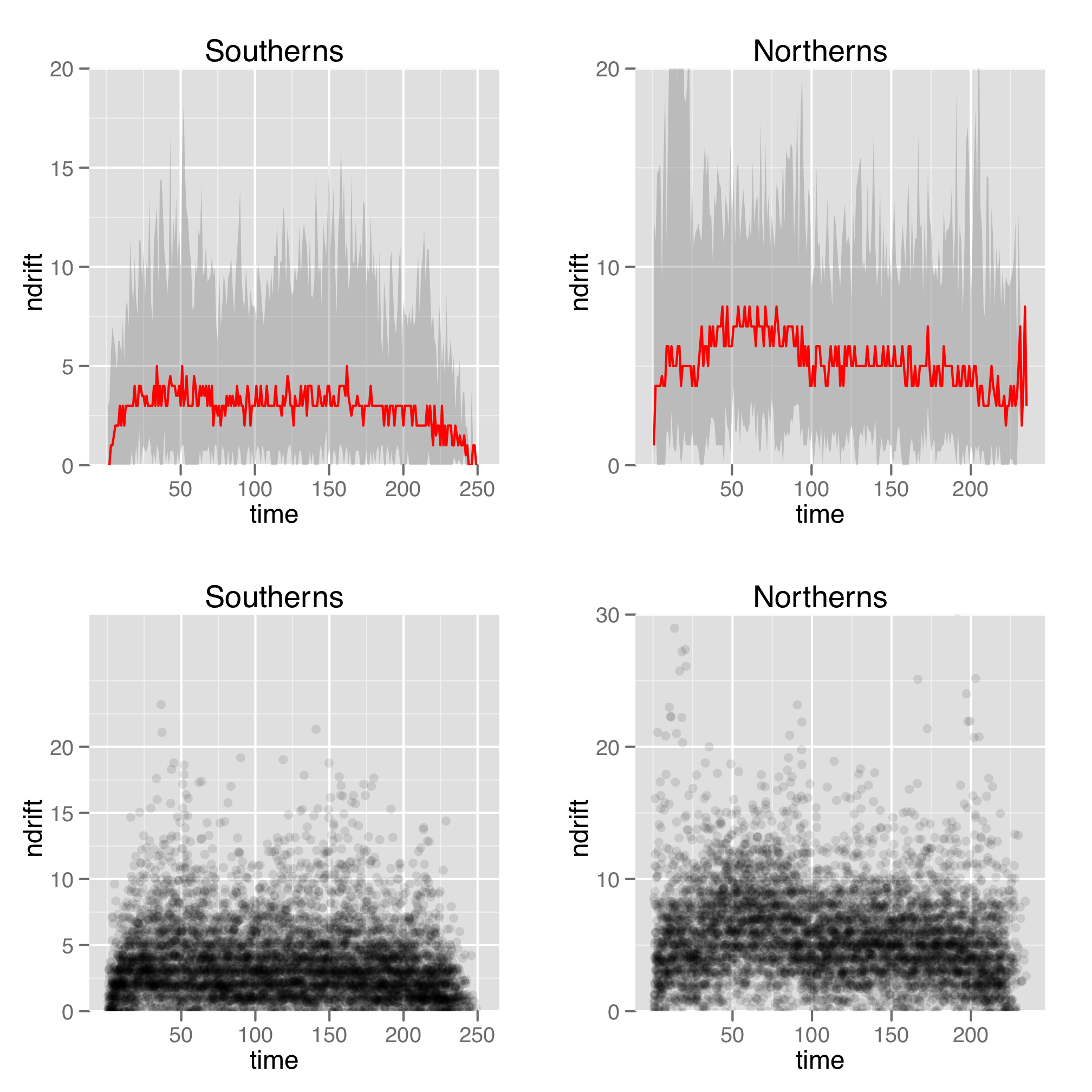


Figure S1.4. Daily drift dive by time aggregated across individuals for southerns (left panels) and northerns (right panels). As compared to northern elephant seals, southern elephant seals exhibit drift dives earlier, and at a lower rate. Northerns have an initial peak around 60 days out with an average (during the peak) in the 7-8 dives/day range. In contrast, southerns have an initial peak closer to 50 days out, with a daily average of approximately 4-5 dives/day.


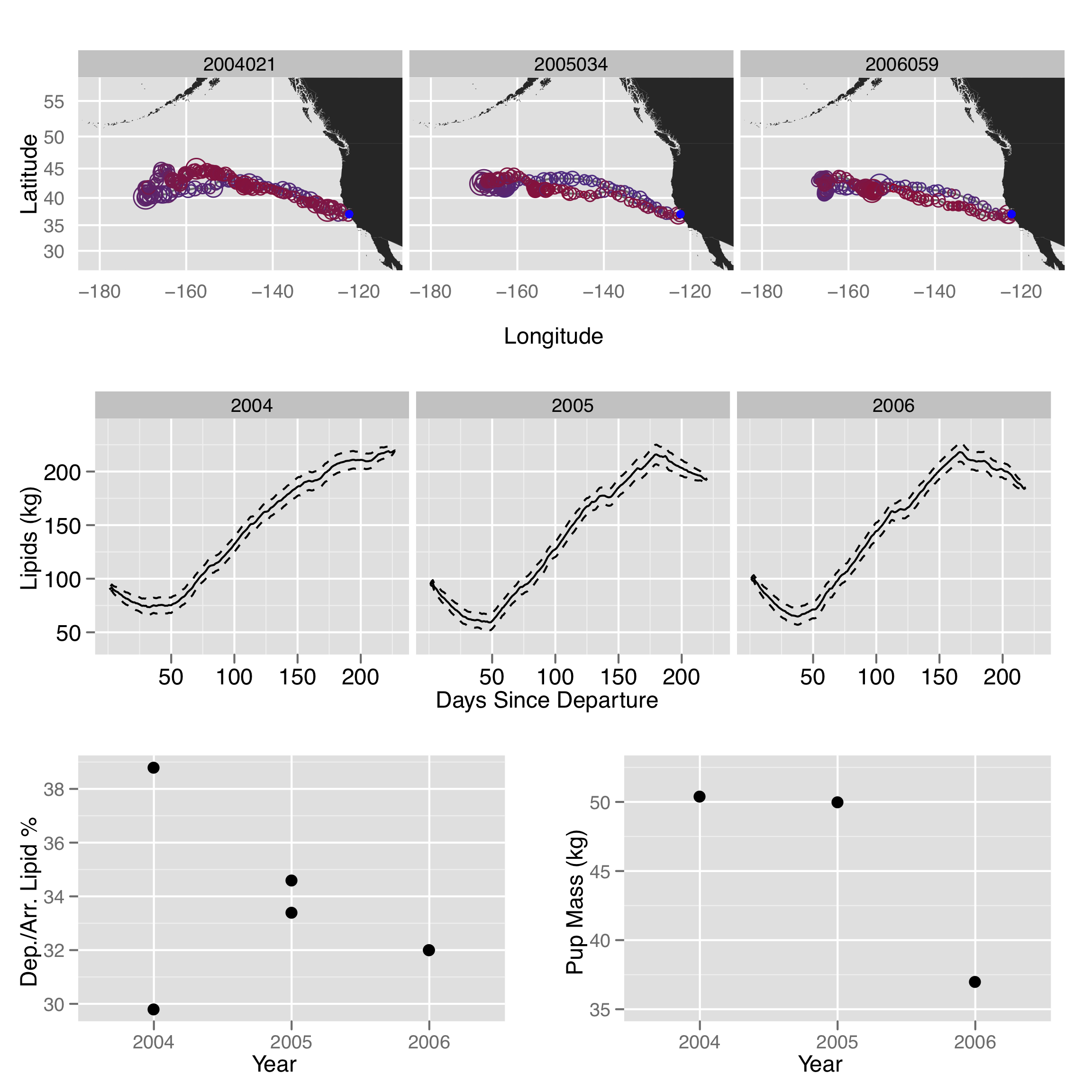


Figure S1.5. Foraging trip by animal M141 in three separate years – 2004, 2005, and 2006. Top panel depicts the map color and symbol coded by drift rate and # of drift dives. The animal employs a Pelagic strategy and in each year forages almost identically.. Middle panel shows estimates of daily lipid gain across the track. Bottom left panel notes the departure (lower #) and arrival lipid percentage (upper number). Bottom right panel shows the weight of the pup. Note that departure and arrival percentage (bottom left panel) in 2006 was the same at departure and arrival. In this year, she puts on fewer lipids and returns with a smaller pup.


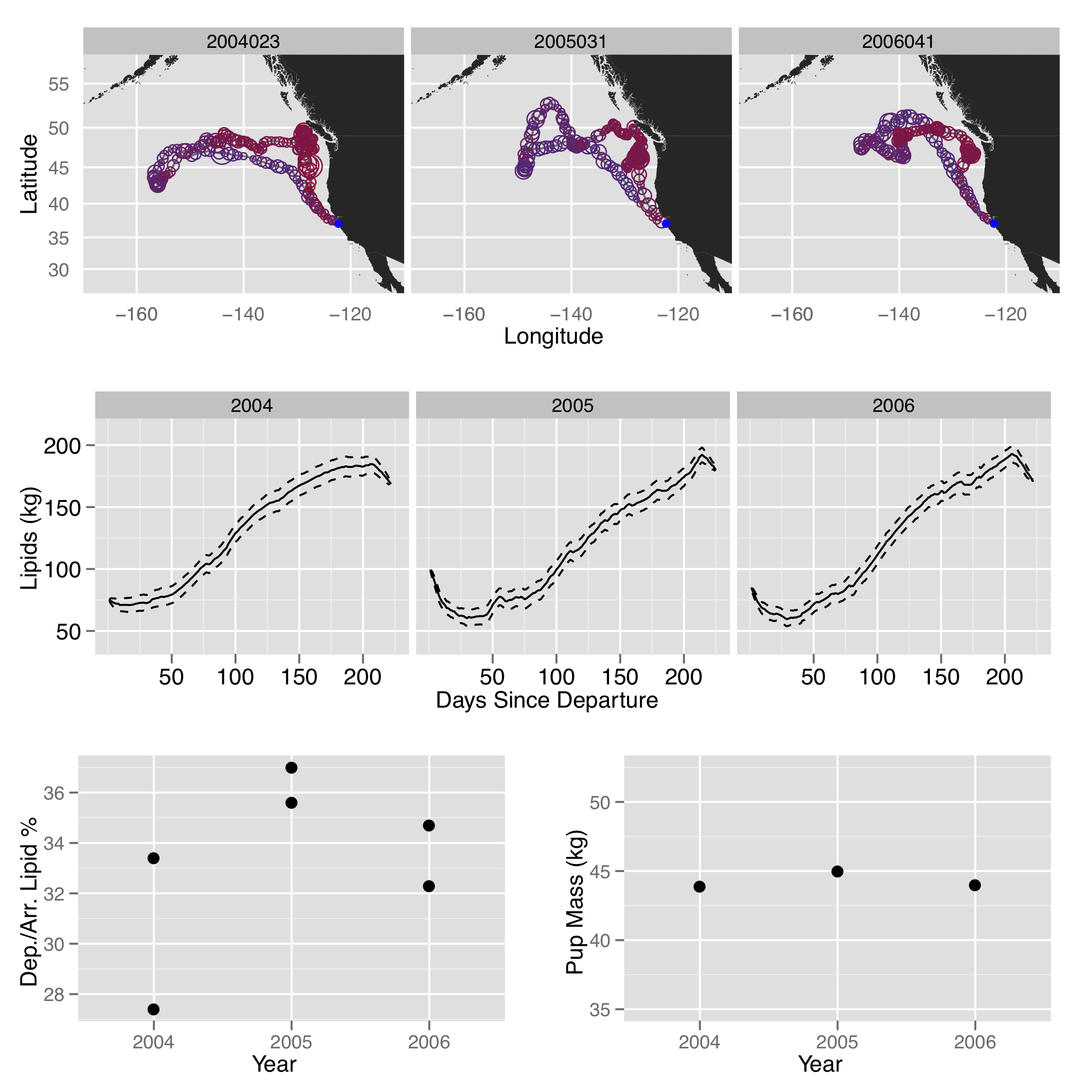


Figure S1.6. Foraging trip by animal O401 in three separate years – 2004, 2005, and 2006. Top panel depicts the map color and symbol coded by drift rate and # of drift dives. The animal employs a mix of foraging strategies with the first year being a mix of pelagic and Northeast Pacific. In 2005 and 2006, she forages almost exclusively in the Northeast Pacific zone. Note the large increase in lipids around the 90^th^ day of her trip in 2004. Middle panel shows estimates of daily lipid gain across the track. Bottom left panel notes the departure (lower #) and arrival lipid percentage (upper number). Bottom right panel shows the weight of the pup.


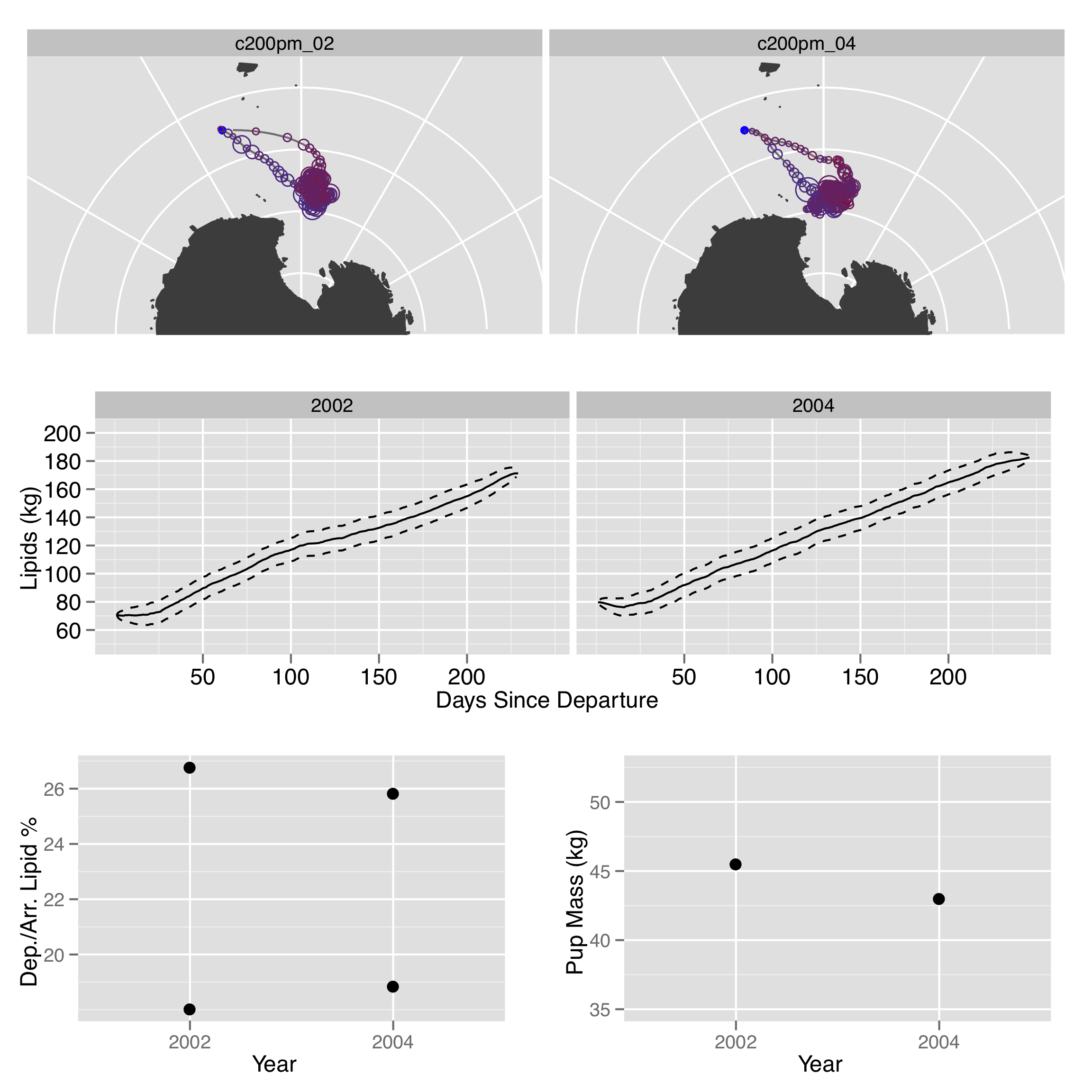


Figure S1.7. Foraging trip by animal c200 in two separate years – 2002, and 2004. Top panel depicts the map color and symbol coded by drift rate and # of drift dives. The animal employs a Ross Sea strategy and in 2004 returns to the same foraging area visited previously in 2002. Middle panel shows estimates of daily lipid gain across the track. Bottom left panel notes the departure (lower #) and arrival lipid percentage (upper number). Bottom right panel shows the weight of the pup.


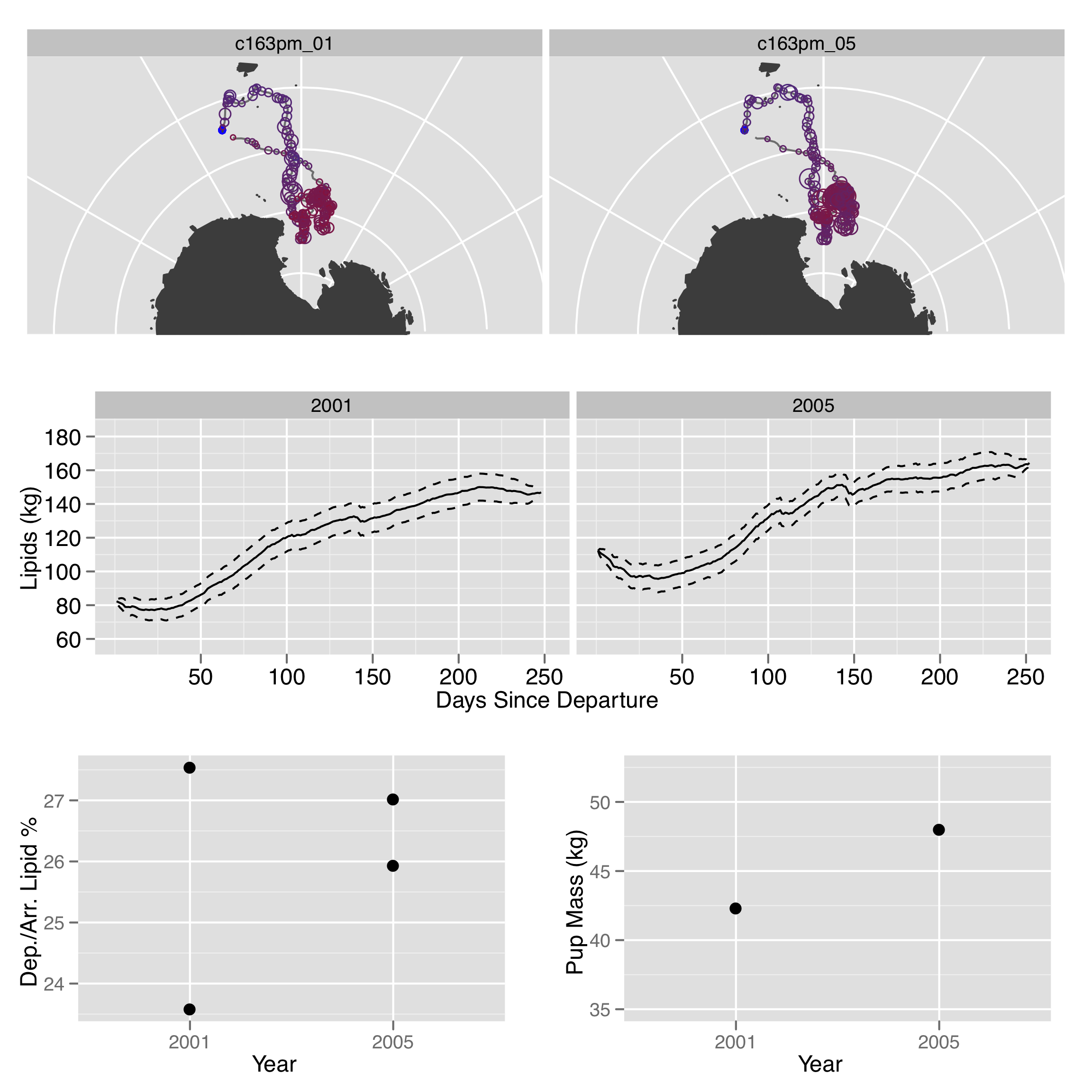


Figure S1.8. Foraging trip by animal c163 in two separate years – 2001, and 2005. Top panel depicts the map color and symbol coded by drift rate and # of drift dives. The animal employs a Ross Sea strategy and in 2005 returns to the same foraging area visited previously in 2001. Middle panel shows estimates of daily lipid gain across the track. Bottom left panel notes the departure (lower #) and arrival lipid percentage (upper number). Bottom right panel shows the weight of the pup.


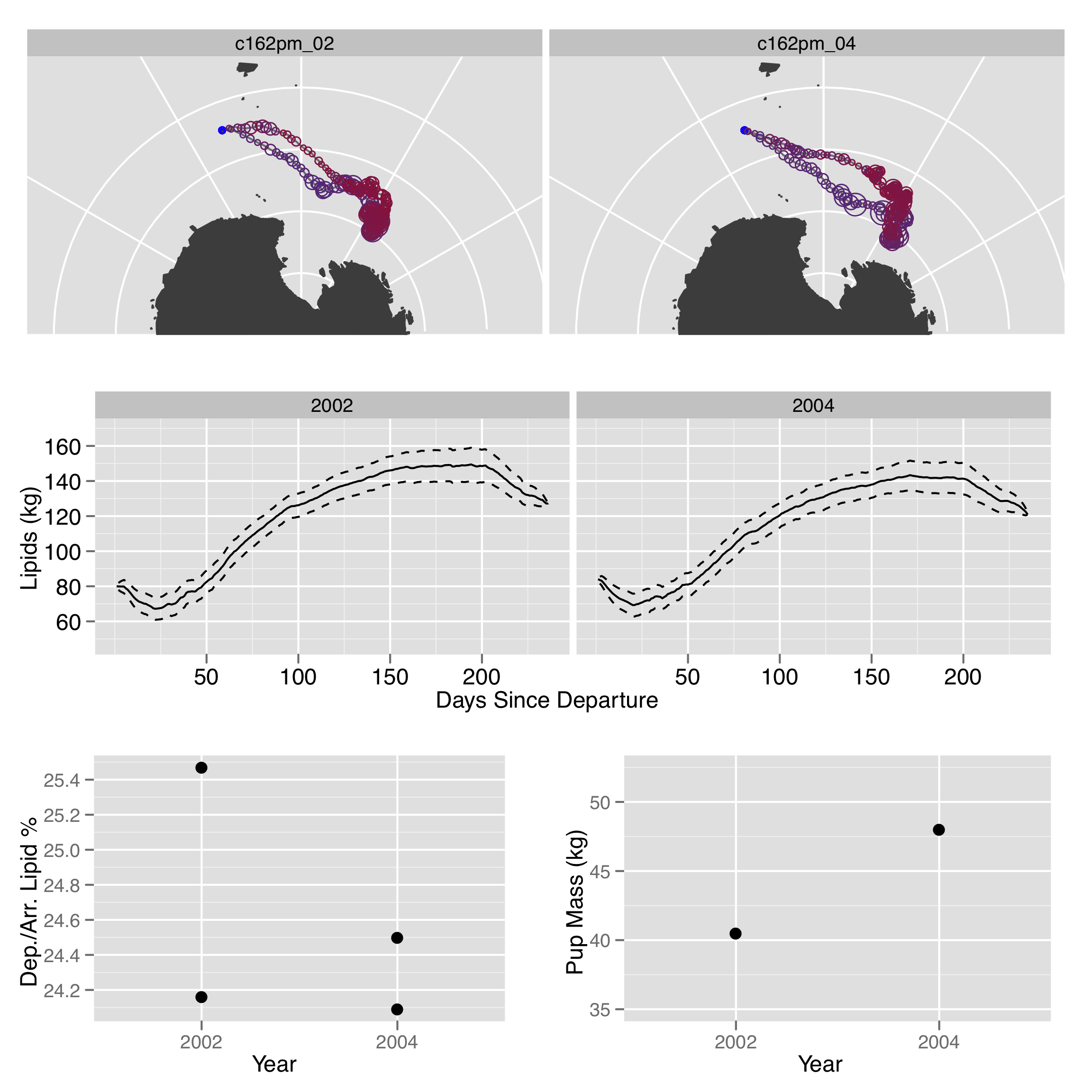


Figure S1.9. Foraging trip by animal c162 in two separate years – 2002, and 2004. Top panel depicts the map color and symbol coded by drift rate and # of drift dives. The animal employs a Ross Sea strategy and in 2004 returns to the same foraging area visited previously in 2002, though her track shows more overlap in 2002. Middle panel shows estimates of daily lipid gain across the track. Bottom left panel notes the departure (lower #) and arrival lipid percentage (upper number). Bottom right panel shows the weight of the pup. Note that this animal puts on less lipid in 2004, but produces a larger pup.


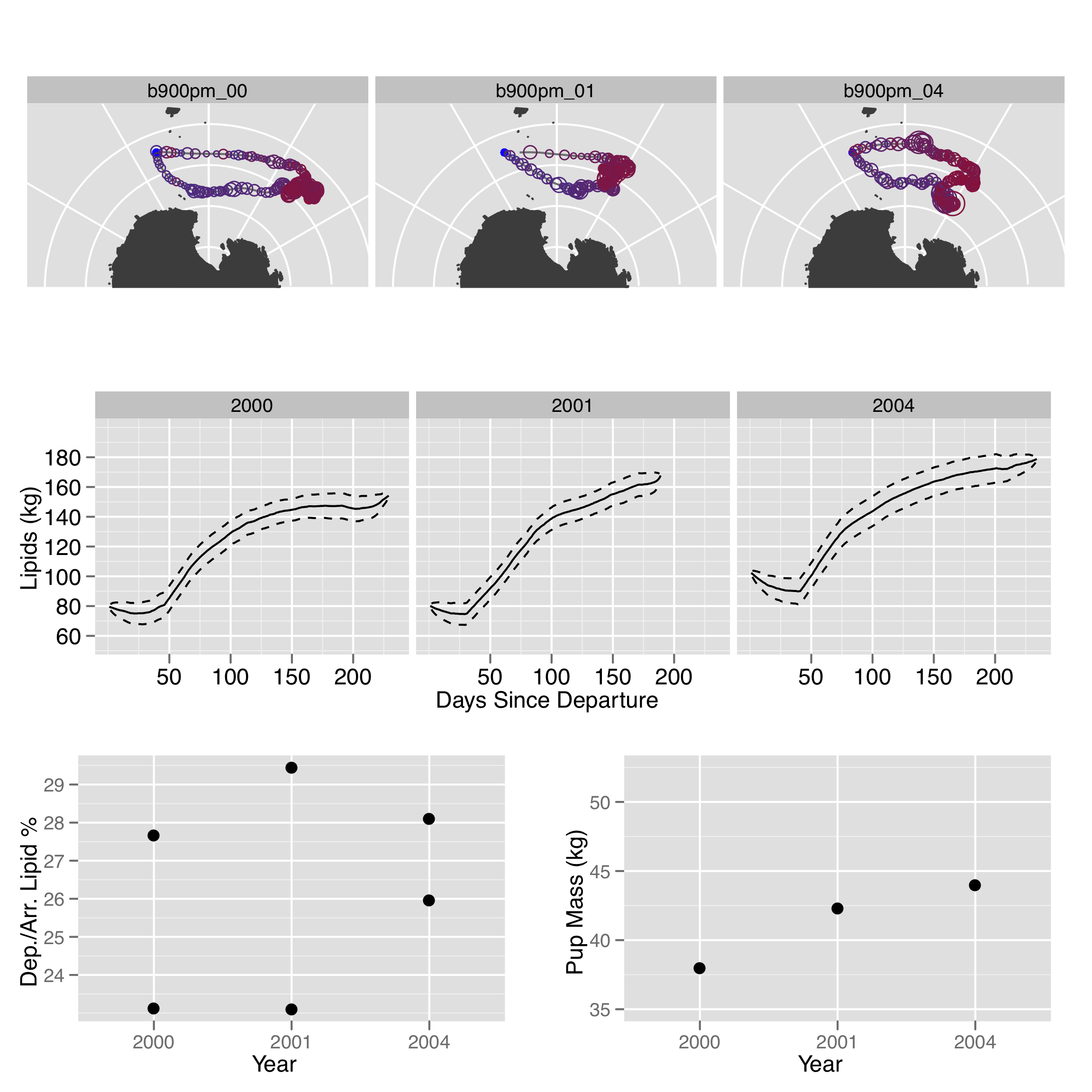


Figure S1.10. Foraging trip by animal b900 in three separate years – 2000, 2001, and 2004. Top panel depicts the map color and symbol coded by drift rate and # of drift dives. The animal employs a Pelagic strategy and in 2004. Note that in 2000 she ventures much farther than in subsequent years. Middle panel shows estimates of daily lipid gain across the track. Bottom left panel notes the departure (lower #) and arrival lipid percentage (upper number). Bottom right panel shows the weight of the pup. The mom gains mass across the three years, i.e. she starts each trip heavier than the previous year(s), and each year she produces a larger pup.


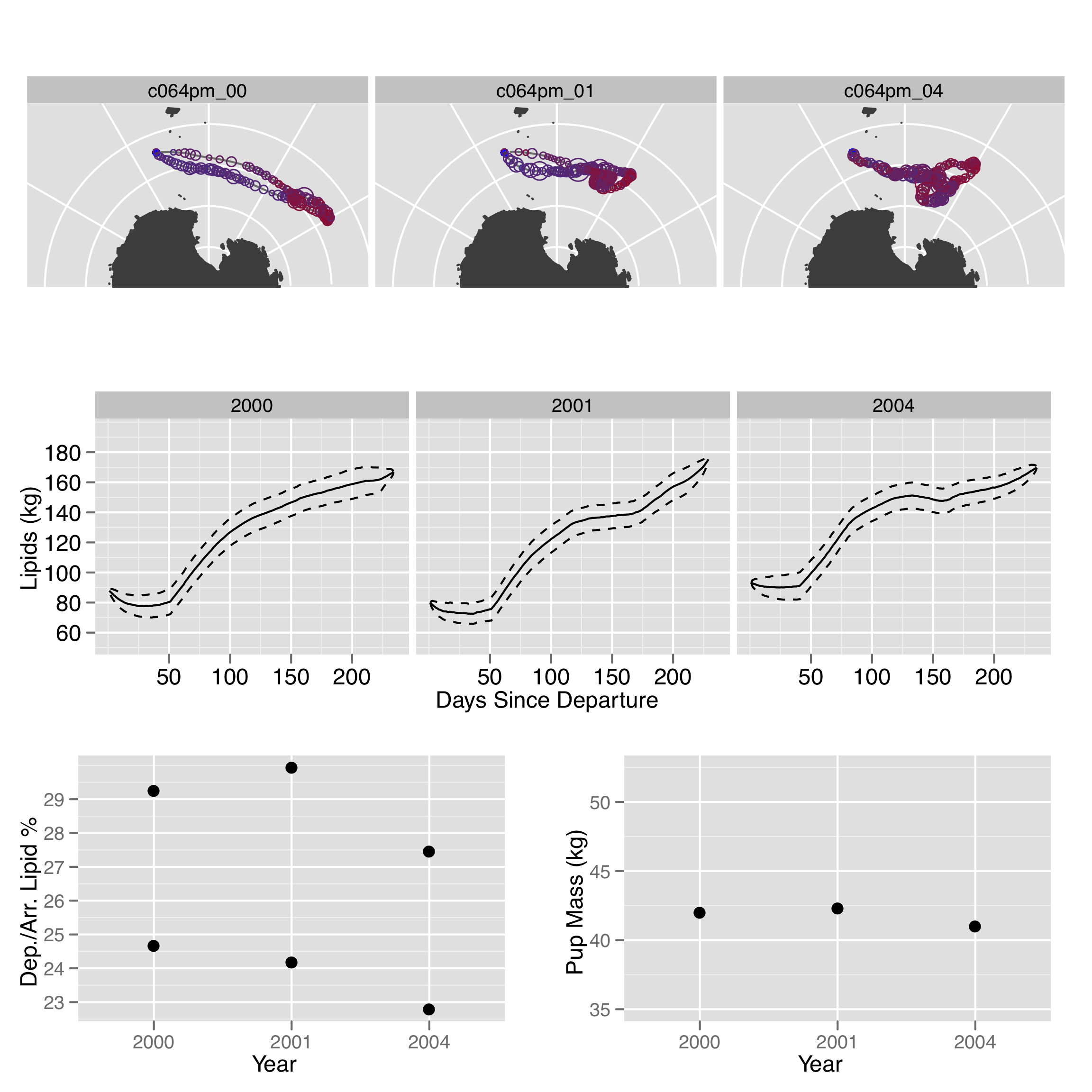


Figure S1.11. Foraging trip by animal c064 in three separate years – 2000, 2001, and 2004. Top panel depicts the map color and symbol coded by drift rate and # of drift dives. The animal employs a Pelagic strategy and in 2004. Middle panel shows estimates of daily lipid gain across the track. Bottom left panel notes the departure (lower #) and arrival lipid percentage (upper number). Bottom right panel shows the weight of the pup. While she seems to fare worse each year, i.e. starting leaner, and especially in 2004, returning leaner, she produces pups of almost identical weights each year.
