# Supplementary material for: Estimating resource acquisition and at-sea body condition of a marine predator
Source: J Anim Ecol. 2013 Jul 19;82(6):1300–15. doi: 10.1111/1365-2656.12102 (PMC4028992; doi:10.1111/1365-2656.12102)
Supplement: Appendix S2 — Table S1. Summary information for 29 northern elephant seals. Table S2. Sunmary information for 30 southern elephant seals. Table S3. Mean posterior estimate of the covariance matrix for random effects (intercept, lipid to lean ratio, and transit) for Northern elephant seals. Table S4. Mean posterior estimate of the covariance matrix for random effects (intercept, lipid to lean ratio, and transit) for Southern elephant seals. [file jane0082-1300-sd2.pdf]

Table S1. Summary information for 29 northern elephant seals. * indicates the same animal tagged in different years.

Seal ID	Start Date (Y-M-D)	End Date (Y-M-D)	Length (Days)	Departure Lipid %	Arrival Lipid %
R589	2004-06-06	2005-01-20	229	29.2	36.9
O448	2004-05-27	2005-01-09	228	29.4	37.3
M141*	2004-05-29	2005-01-10	227	29.8	38.8
O401*	2004-06-01	2005-01-08	222	27.4	33.4
O968	2004-05-27	2005-01-16	235	30.5	37.4
1595	2004-06-18	2005-01-31	228	31.5	38.5
N354	2004-06-19	2005-01-27	223	30.7	37.8
N513	2004-06-10	2005-01-23	228	30.6	36.9
L274	2004-06-18	2005-01-29	226	27.7	33.9
L844	2005-05-24	2006-01-07	229	29.6	38.9
1544	2005-05-23	2006-01-01	224	32.2	39.8
L521	2005-05-30	2006-01-07	223	31.7	33.7
O 55	2005-05-16	2006-01-03	233	32.1	30.9
L563	2005-06-01	2006-01-14	228	30.7	33.4
O401*	2005-05-31	2006-01-10	225	35.6	37.0
M141*	2005-06-10	2006-01-15	220	33.4	34.6
1914	2005-06-15	2006-01-22	222	31.2	39.9
R 55	2005-06-15	2006-01-20	220	29.9	34.8
O585	2005-06-25	2006-01-24	214	28.3	35.9
O401*	2006-06-03	2007-01-10	222	32.3	34.7
O256	2006-06-05	2007-01-21	231	33.2	33.0
1817	2006-06-06	2007-01-23	229	31.0	33.0
M583	2006-06-14	2007-01-22	219	29.1	31.2

1502	2006-06-14	2007-01-17	222	32.4	35.5
M141*	2006-06-20	2007-01-23	218	32.0	32.0
O564	2006-06-18	2007-01-22	219	32.3	33.7
N981	2007-06-13	2008-01-17	219	31.5	25.9
S217	2007-06-13	2008-01-25	227	27.5	32.4
W364	2007-06-10	2008-16-16	221	32.1	34.3

Table S2. Sunmary information for 30 southern elephant seals. Note that several seals were tagged multiple times in different years.

Seal ID	Start Date (Y-M-D)	End Date (Y-M-D)	Length (Days)	Departure Lipid %	Arrival Lipid %
B131	2001-02-10	2001-09-29	232	20.7	29.7
B143	2004-02-17	2004-10-07	234	20.3	24.6
B347	2004-02-02	2004-10-05	247	21.0	27.2
B362	2001-02-21	2001-10-07	229	23.0	28.3
B533	2000-02-02	2000-09-27	239	23.7	26.2
B569	2000-03-02	2000-10-06	219	23.5	29.3
B650	2002-02-01	2002-09-21	230	21.5	28.6
B889	2000-02-05	2000-10-01	240	19.2	30.9
B900	2000-02-24	2000-10-09	229	23.1	29.7
B900*	2001-02-20	2001-08-27	189	23.1	31.8
B900*	2004-02-14	2004-10-04	234	26.0	30.2
C064	2000-02-20	2000-10-10	234	24.7	31.6
C064*	2001-02-17	2001-10-03	229	24.2	32.3
C064*	2004-02-25	2004-10-16	235	22.8	29.4
C161	2004-02-15	2004-10-04	233	21.8	26.1
C162	2002-02-14	2002-10-10	236	24.2	27.7
C162*	2004-02-15	2004-10-06	235	24.5	26.6
C163	2001-02-05	2001-10-10	248	23.6	29.9
C163*	2005-02-01	2005-10-10	252	25.9	29.3
C200	2002-02-06	2002-09-22	229	18.0	28.8
C200*	2004-02-11	2004-10-13	246	18.8	27.5
C209	2002-01-28	2002-09-22	238	19.7	29.8

C217	200-02-13	2000-10-03	234	21.5	29.0
C312	2002-01-29	2002-09-29	241	22.7	27.6
C699	2001-01-28	2001-09-24	240	20.9	39.0
C923	2002-01-29	2002-09-30	245	19.7	27.9
F993	2005-02-14	2005-09-30	229	24.1	31.5
H233	2004-01-29	2004-09-29	245	16.8	31.1
H285	2004-02-18	2004-10-09	235	19.9	27.0
H833	2004-12-17	2001-10-08	235	20.2	27.1

Table S3. Mean posterior estimate of the covariance matrix for random effects (intercept, lipid to lean ratio, and transit) for Northern elephant seals. Variance estimates are along the diagonal; covariances on the off diagonal.

Intercept	Lipid/Lean Ratio	Transit
0.0064	0.0	0.0
0.0	0.0065	0.0
0.0	0.0	0.0067

Table S4. Mean posterior estimate of the covariance matrix for random effects (intercept, lipid to lean ratio, and transit) for Southern elephant seals. Variance estimates are along the diagonal; covariances on the off diagonal.

Intercept	Lipid/Lean Ratio	Transit
0.0062	0.0	0.0
0.0	0.0062	0.0
0.0	0.0	0.0064
