# Supplementary material for: Estimating resource acquisition and at-sea body condition of a marine predator
Source: J Anim Ecol. 2013 Jul 19;82(6):1300–15. doi: 10.1111/1365-2656.12102 (PMC4028992; doi:10.1111/1365-2656.12102)
Supplement: Supplementary file 3 — Appendix S3. Further details on: (1) how the Gibbs sampler was constructed to fit the model to the data; (2) the fit of the model to simulated data; and (3) details on the different covariates included in the model. Appendix S4. Complete plots for each individual Northern elephant seal used in the analyses. Four plots per individual show: the overview map, the start and stop lipid percentage, the drift rate time series, and the time series of lipid estimates. [file jane0082-1300-sd3.doc]

# Appendix 3

This appendix provides further details on: 1) how the Gibbs sampler was constructed to fit the model to the data; 2) the fit of the model to simulated data; and 3) details on the different covariates included in the model.

## Gibbs Sampler

Posterior simulation was accomplished with Markov chain Monte Carlo (MCMC) using Metropolis-within-Gibbs. Code was written in R by J.S. Clark. The MCMC includes submodels to impute missing covariates, sample parameters for fixed effects, sample random effects, sample parameters for the data model, sample process error, and predict rate of lipid gain. Here we summarize algorithms.

Based on eqns 1 and 3 from the main text, drift rate parameters for the observation model are sampled from

where

*h_i,t_* is the number of drift dives of individual *i* at time *t*. The observation error variance is sampled from

Fixed effects for the process model are sampled from

First stack the design vectors for each individual and day, excluding days *T_i_* to create a matrix *X*. A vector *Y* consists of the stacked vector of first differences minus random effects for individual *i*, , which are then concatenated as ; each element of *Y* is paired with the corresponding row of *X*. Then the conditional posterior is sampled from

where

The process error variance is sampled from

To sample random effects we created vectors of first differences minus fixed effects,, and random effects design matrix for individual *i*, . Random effects are sampled from

where

The random effects covariance is sampled from

where is the stacked *n* by *q* matrix of random effects parameters.

Missing drift rate values were sampled from

where *A_i,t_* is row *i,t* of *A*, and *h_i,t_* is the number of drift dives taken by individual *i* at time *t*.

Conditional posteriors for missing input variables depend on whether or not those variables enter only as fixed effects or as both fixed and random effects and include a conditional autoregressive (CAR) prior. Consider a vector of missing input values for individual *i* at time *t*. If there is a single missing value this is a scalar quantity. Let

and

where superscripts (*m*) and (*n*) indicate vector elements that are missing and not missing, respectively. For elements that are not included as random effects. The missing vector is sampled from

where

where and are contributed by the CAR prior.

## Simulation Experiments

To test the performance of the model and the algorithm, we performed a cross-validation experiment with the drift data and with the environmental covariates. Because the model was built to estimate missing data, we used known datums to compare model performance.

To do this, we randomly sampled 30 known data points, that were not in either the start or end time of an individual’s track. We then used a converged model as a starting point, and ran the model forward for 5,000 iterations through the Gibbs sampler. We did this for each of the 30 points, and recorded summary statistics from this MCMC chain, i.e. median, and 95% BCI.

We then plotted the true value versus the estimated and included the BCI. We did this separately for drift dive data and for the environmental covariates. Fit was very good for both the drift data (Figure S3.3), and for the environmental data (Figure S3.4). For the drift data, where the BCI does not cover the true value, these are instances where there is only 1 drift dive for the whole day. Because the drift dive measurements can vary substantially within one day, and because the observation error is tied to the number of drift dives, we were confident that the model is performing well.

Similarly for the environmental covariates the model estimates true values very well (Figure S3.5). The covariate that was estimated least well was transit. There are likely two reasons for this: 1) the satellite measurements for southerns (which is what we used for this cross-validation exercise) did not include error estimates; and 2) there is a CAR prior on the mean for missing covariate data that includes the previous time, the current time, and the future time. Because of this if the true value is very different from the other two, the prior can pull the estimate towards the mean of the three timesteps. Both the transit and distance to colony covariates were log transformed in this analysis.

## Input Data

As noted in the manuscript, we focus estimation on the lipids, not on the lean tissue. We need to establish a biologically realistic fixed estimate of the lipids. The pattern we assumed was as follows. We assume that a foraging female gains lean mass in a linear fashion throughout the course of the post-molt trip, as does the developing fetus during the final third of the trip (Fig. S3.1).

This linear model was selected on the basis of captive and field experiments (Condit and Ortiz, 1987, Field et al., 2005) and parsimony. We explored two other functional forms: 1) no accommodation for the additional weight gain of the pup; and 2) a gain process that favorably added lean tissue early in the trip, i.e. a tri-phase lean tissue gain process of high rates of gain, medium rates of gain, and then high rates again at the last stage of the pregnancy. Though the systems are obviously quite different, this linear gain process is consistent with measured gains in ruminants (Owens et al., 1993). Regardless, the choice of the model did not have a substantial effect on the estimates of lipid gain.

## Model Formulation: priors and covariates

We conducted an extensive analysis of different model combinations on each of the two elephant seal datasets. The covariates we tried are described in Table S3.1. Covariates in the table can either be shared across species, e.g. transit rate, or unique to each species, e.g. Ice Concentration for southern elephant seals (Table S3.1). We include a description of each covariate as well as the assumption for the sign of the response of the lipid gain process to each covariate; three possibilities are noted: 1) response could be positive or negative (0), 2) response could be positive (+); and 3) the response could be negative (-).

For example, we assumed that an increased number of drift dives each day means that the animal is processing more food, and thus is more likely to gain weight (Table S3.1). Similarly the fatter an animal gets, that is as the lipid to lean ratio (lxrx) increases, we expect it is harder to put on weight (Webb et al. 1998) (Table S3.1). Similarly based on past research, we expected animals who transited more would forage less and therefore gain less (Kuhn et al. 2008, Robinson et al. 2010).Where a sign is indicated, we constrained the sampler to be positive or negative for that covariate, that is in the draw for the $\beta$ parameters from the multivariate normal upper or lower bounds were set to 0 to ensure proposed values were negative or positive, respectively.

Where we assumed the response to be negative or positive, we similarly constrained the prior for the$\beta$ parameter. Priors for the$\beta$ parameters were otherwise uninformative. We did include informative priors for the means of the process variance and the observation variance. To test the effect of prior strength, we ran the same model and varied the mean values for the error variances, parameters *c_2_* and *s_2_* above. The strength of the prior reflected the amount of variance left unexplained by the model, i.e. the larger the mean value the larger the variance. After reviewing the results, and comparing them with known weight loss values on land (Costa et al. 1986, Crocker et al. 1988, Crocker et al. 2001), we settled on *c_2_* = 1 and *s_2_* = 4. This translates to a maximum observed drift rate of 1 m/s unexplained by the model, and a maximum of 4 kilograms loss/gain per day unexplained by the model. We used on-land values because there are no known at-sea gain/loss measurements.

We experimented with a variety of covariates in the model for northern elephant seals and for southern elephant seals. These covariates included “internal” covariates, e.g. surface transit rate, as well as “environmental” covariates, e.g. ice concentration. Here we provide necessary background information on how the individual covariates were determined, sampled, and included in the model. Where relevant, we indicate the prior assumption we had about the nature of the response of lipid gain to each individual covariate.

For southern elephant seals, covariates included mean daily transit (km), distance to colony, number of drift dives per day (linear and quadratic response), time (days since departure, both linear and quadratic), a behavioral state index conditioned upon distance to colony (Figure S3.2), ice concentration, distance to ice, depth of the sea floor, lipid to lean mass ratio, sea surface temperature, and distance to continental shelf (upper and lower). The assumption for the effect of lipid to lean ratio on lipid gain was that it would act as a penalty; as animals gained lipids a) they would find it harder to dive, and b) have a diminished physiological need to gain lipids. Ice concentration was sampled from the National Snow and Ice Data Center’s 25 km daily ice concentration rasters (<http://nsidc.org/data/nsidc-0081.html>, last accessed 2/16/2011). We converted these values to a binary 0/1 value representing No-Ice/Ice with a threshold cutoff of 15% coverage within the 25 km cell (Thums et al. 2011).

For northern elephant seals we also included a modeled zooplankton density covariate (Chai et al. 2002, Dugdale et al. 2002, Chai et al. 2007). We included three different formulations of this covariate: 1) values at the ocean surface; 2) values at the depth of the foraging dive; and 3) values at the depth of the drift dive. We chose these levels on first principles; presumably foraging animals are more concerned with prey, and therefore we assumed that prey values encountered by the animal at the depth of the foraging dive would be higher then prey values sampled at the depth of the drift dive. Sampling of these environmental covariates was performed using the Marine Geospatial Ecology Toolbox (Roberts et al. 2010).

For both species, we derived the behavioral state indexed upon distance to colony as follows. For each animal for each day’s mean daily position, we calculated the great circle distance to the colony. We then plotted these time series, which almost all had a trapezoidal shape, i.e. there was a transit away from the colony phase, followed by a foraging phase, which was followed by a return to the colony phase. The transition points were estimated through visual inspection of the plots, after which a binary state was assigned to each behavior (Figure S3.2). To test hypothesis number two, i.e. foraging strategy, we included foraging strategy state as a covariate, giving a binary state to each species’ three foraging states. Specifically we had a binary state for each of the three foraging strategies for each species.

Finally, to test hypothesis number three we included departure lipid percentage as a covariate, as well as an interaction term between departure lipid percentage and the first transiting away state.

Table S3.2 details all of the different model formulations that were attempted as part of the analysis. “Final” models are highlighted in grey.

### Model Selection

Model selection is based on the marginal likelihood, approximated using the approach of Chib (1995). Inverting Bayes Theorem allows evaluation of

where *β'* is a specific *β* vector having posterior density simulated by Gibbs sampling, *D* is the set of all drift observations, and *m* is model, consisting of a set of input variables. Results are stable and essentially independent of which particular parameter vector *β'* is chosen to evaluate it. Models are evaluated based on the posterior model probability, derived from the model prior *p*(*m*) and marginal likelihood *p*(*D*|*X,m*),

(Zellner 1978, Clyde and Ghosh In Press), where *m* is a model with *m* number of input variables *X*. This is the basis for many model choice criteria, including posterior odds and Bayes factors (Clyde and George 2004) and model averaging (Hoeting et al. 1999). Following Scott and Berger (2010) we apply the model prior

(George and McCullough 1997), where M is total number of variables considered (we considered a maximum of 16), and *p* is treated as an unknown, thus providing a multiplicity correction (Scott and Berger 2010). The marginal likelihood is

With prior *beta*(*p*|1, 1), the posterior model probability is

8

i.e., a mixture (George and McCullough 1993, Clyde and George 2004).

## References

Hoeting, J. A., D. Madigan, A. E. Raftery, and C. T. Volinsky. 1999. Bayesian model averaging: A tutorial. Statistical Science **14**:382-417.

Kuhn, C. E., D. E. Crocker, Y. Tremblay, and D. P. Costa. 2008. Time to eat: measurements of feeding behaviour in a large marine predator, the northern elephant seal Mirounga angustirostris. Journal of Animal Ecology **78**:513-523.

Webb, P., D. Crocker, S. Blackwell, D. Costa, and B. Boeuf. 1998. Effects of buoyancy on the diving behavior of northern elephant seals. Journal of Experimental Biology **201**:2349.

Costa, D. P., B. J. Le Boeuf, C. L. Ortiz, and A. C. Huntley. 1986. The energetics of lactation in the northern elephant seal. Journal of Zoology, London **209**:21-33.

Chai, F., R. C. Dugdale, T. H. Peng, F. P. Wilkerson, and R. T. Barber. 2002. One-dimensional ecosystem model of the equatorial Pacific upwelling system. Part I: model development and silicon and nitrogen cycle. Deep Sea Research Part II: Topical Studies in Oceanography **49**:2713-2745.

Chai, F., M.-S. Jiang, Y. Chao, R. C. Dugdale, F. Chavez, and R. T. Barber. 2007. Modeling responses of diatom productivity and biogenic silica export to iron enrichment in the equatorial Pacific Ocean. Global Biogeochemical Cycles **21**:GB3S90.

Chib, S. 1995. Marginal likelihood from the Gibbs output. Journal of American Statistical Association **90**:1313-1321.

Clyde, M. and E. I. George. 2004. Model uncertainty. Statistical Science **19**:81-94.

Clyde, M. and J. Ghosh. In Press. A note on the bias in estimating posterior probabilities in variable selection. Biometrika.

Crocker, D. E., P. M. Webb, D. P. Costa, and B. J. Le Boeuf. 1988. Protein catabolism and renal function in lactating northern elephant seals. Physiological Zoology **71**:485-491.

Crocker, D. E., J. D. Williams, D. P. Costa, and B. J. Le Boeuf. 2001. Maternal traits and reproductive effort in northern elephant seals. Ecology **82**:3541-3555.

Dugdale, R. C., R. T. Barber, F. Chai, T. H. Peng, and F. P. Wilkerson. 2002. One-dimensional ecosystem model of the equatorial Pacific upwelling system. Part II: sensitivity analysis and comparison with JGOFS EqPac data. Deep Sea Research Part II: Topical Studies in Oceanography **49**:2747-2768.

George, E. I. and R. E. McCullough. 1993. Variable selection via Gibbs sampling. American Statistical Association **88**:881-889.

George, E. I. and R. E. McCullough. 1997. Approaches for Bayesian variable selection. Statistica Sinica **7**:339-373.

Roberts, J. J., B. D. Best, D. C. Dunn, E. A. Treml, and P. N. Halpin. 2010. Marine Geospatial Ecology Tools: An integrated framework for ecological geoprocessing with ArcGIS, Python, R, MATLAB, and C++. Environmental Modelling & Software **25**:1197-1207.

Robinson, P. W., S. E. Simmons, D. E. Crocker, and D. P. Costa. 2010. Measurements of foraging success in a highly pelagic marine predator, the northern elephant seal. Journal of Animal Ecology **79**:1146-1156.

Scott, J. G. and J. O. Berger. 2010. Bayes and empirical-Bayes multiplicity adjustment in the variable-selection problem. The Annals of Statistics **38**:2587-2619.

Thums, M., C. Bradshaw, and M. A. Hindell. 2011. In situ measures of foraging success and prey encounter reveal marine habitat-dependent search strategies. Ecology **92**:1258-1270.

Zellner, A. 1978. Jeffreys-Bayes posterior odds ratio and the Akaike information criterion for discriminating between models. . Economics Letters **1**:337-342.

### Table S3.1. Summary of the different covariates tested in the model for both species

| Species | Covariate | Description | Prior Direction |
| --- | --- | --- | --- |
| Both | Intercept | Model intercept | 0 |
|  | Transit | Mean daily surface transit (km) | - |
|  | ndrift | Number drift dives recorded per day | + |
|  | ndrift^2^ | Number drift dives recorded per day squared | - |
|  | lxrx | Daily ration of lipid tissue to lean tissue | - |
|  | time | Days since departure from colony | + |
|  | time^2^ | Days since departure from colony | - |
|  | DepLipPct | Percent of departure body mass that is lipids | 0 |
|  | X | Interaction term, i.e. s1XDepLipPct is the interaction between the transit away state and departure lipid percentage | 0 |
|  | s1 | Distance to colony based states. We used plots of distance to colony for each seal to visualize three phases: transit away (s1); foraging (s2); and transit back (s3). | 0 |
|  | s2 | Foraging phase | 0 |
|  | s3 | Transit back to the colony phase | 0 |
| Northern | ROMS_zz2_F | Mean zooplankton abundance from the ROMS-CoSiNE model, at the mean daily depth of the foraging dive | + |
|  | ROMS_zz2_D | Mean zooplankton abundance from the ROMS-CoSiNE model, at the mean daily depth of the drift dive | - |
|  | stratPelagic | Animals employing the pelagic transition zone foraging strategy (Figure S1.1) | 0 |
|  | stratNEPacific | Animals employing the Northeast Pacific foraging strategy (Figure S1.1) | 0 |
|  | stratCoastal | Animals employing the coastal (US/Canada) foraging strategy (Figure S1.1) | 0 |
|  | stratPelagicS1, stratPelagicS2, etc. | Transit away phase for animals employing the pelagic transition zone foraging strategy, foraging phase for the same strategy, etc. | 0 |
| Southern | IceConc | Daily Ice concentration for the 25 km^2^ cell the seal is in. Water/Ice threshold set at 15%. | + |
|  | d2colony | Distance from mean daily position to colony (km) | + |
|  | stratRoss | Animals employing the “Ice-Edge” or Ross Sea foraging strategy (Figure S1.2) | 0 |
|  | stratShelf | Animals employing the “shelf-edge” foraging strategy (Figure S1.2) | 0 |
|  | stratPelagic | Animals employing the “frontal” or pelagic foraging strategy (Figure S1.2) | 0 |
|  | stratRossS1, stratRossS2, etc. | Transit away phase for animals employing the Ross Sea/Ice edge foraging strategy, foraging phase for the same strategy, etc. | 0 |

### Table S3.2. Tally of the different model formulations we attempted for each species. Covariates are labeled as in Table S3.2. The final models used are shaded in gray.

| Species | Covariates | Random Effects |
| --- | --- | --- |
| Northern | int, transit, ndrift, ndrift^2^, lxrx, time, time^2^, lxrxXndrift | int, transit |
|  | int, transit, ndrift, lxrx, time, time^2^, ROMS_zz2, lxrxXndrift | int, transit |
|  | int, transit, ndrift, lxrx, time, time^2^, ROMS_zz2_D, lxrxXndrift | int, transit |
|  | int, transit, ndrift, lxrx, time, time^2^, ROMS_zz2_F, lxrxXndrift | int, transit |
|  | int, transit, ndrift, lxrx, time, ROMS_zz2_D | int, lxrx, ROMS_zz2_D |
|  | int, transit, ndrift, lxrx, time, ROMS_zz2_D | int, lxrx, ROMS_zz2_D |
|  | int, transit, ndrift, lxrx, time, ROMS_zz2_D | int, lxrx |
|  | int, transit, ndrift, lxrx, time, ROMS_zz2_D, stratPelagic, stratNEPacific, stratCoastal, DepLipPct | int, lxrx, transit |
|  | int, transit, ndrift, lxrx, time, ROMS_zz2_D, stratPelagic, stratNEPacific, stratCoastal, DepLipPct | int, lxrx, transit |
|  | int, transit, ndrift, lxrx, time, ROMS_zz2_D, stratPelagic, stratNEPacific, stratCoastal, DepLipPct | int, lxrx, transit |
|  | int, transit, ndrift, lxrx, time | int, lxrx, transit |
|  | int, transit, ndrift, lxrx, time, stratPelagic, stratNEPacific, stratCoastal | int, lxrx, transit |
|  | int, transit, ndrift, lxrx, time, ROMS_zz2_D | int, lxrx |
|  | int, transit, ndrift, lxrx, time, ROMS_zz2_F | int, lxrx |
|  | int, transit, ndrift, lxrx, time, ROMS_zz2_D | int, lxrx, ROMS_zz2_D |
|  | int, transit, ndrift, lxrx, time, ROMS_zz2_F | int, lxrx, ROMS_zz2_F |
|  | int, transit, ndrift, lxrx, s1, s3, DepLipPct, s1XDepLipPct | int, lxrx |
|  | int, transit, ndrift, lxrx, s1, s3, DepLipPct, s1XDepLipPct | int, lxrx |
|  | int, transit, ndrift, lxrx, time, DepLipPct | int, lxrx, transit |
|  | int, transit, ndrift, lxrx, s1, s3, DepLipPct, s1XDepLipPct | int, lxrx |
|  | int, transit, ndrift, lxrx, time, DepLipPct | int, lxrx |
|  | int, transit, ndrift, lxrx, time | int, lxrx |
|  | int, transit, ndrift, lxrx, s1, s3, DepLipPct, s1XDepLipPct | int, lxrx, transit |
|  | int, transit, ndrift, lxrx, strat1, strat3, DepLipPct | int, lxrx, transit |
|  | int, transit, ndrift, lxrx, s1, s3, DepLipPct, s1XDepLipPct | int, lxrx, transit |
|  | int, transit, ndrift, lxrx, stratPelagic, stratNEPacific, stratCoastal, DepLipPct | int, lxrx, transit |
|  | int, transit, ndrift, lxrx, s1, s3, DepLipPct, s1XDepLipPct | int, lxrx, transit |
|  | int, transit, ndrift, lxrx, stratPelagic, stratNEPacific, stratCoastal, DepLipPct | int, lxrx, transit |
|  | int, transit, ndrift, lxrx, stratPelagicS1, stratPelagicS2, stratPelagicS3, stratNEPacificS1, stratNEPacificS2, stratNEPacificS3, stratCoastalS1, stratCoastalS3, DepLipPct, stratPelagicS1XDepLipPct, stratNEPacificS1X DepLipPct, stratCoastalS1XDepLipPct | int, lxrx, transit |
|  | int, transit, ndrift, lxrx, stratPelagicS1, stratPelagicS2, stratPelagicS3, stratNEPacificS1, stratNEPacificS2, stratNEPacificS3, stratCoastalS1, stratCoastalS3, DepLipPct | int, lxrx, transit |
| Southern | int, transit, ndrift, lxrx, IceConc, d2colony, lxrxXndrift | int, transit |
|  | int, transit, ndrift, lxrx, d2colony, lxrxXndrift | int, transit |
|  | int, transit, ndrift, ndrift^2^, time, time^2^, lxrx, lxrxXndrift | int, transit |
|  | int, transit, ndrift, lxrx | int, lxrx |
|  | int, transit, ndrift, lxrx | int, lxrx, transit |
|  | int, transit, ndrift, lxrx, stratRoss, stratShelf, stratPelagic, DepLipPct | int, transit |
|  | int, transit, ndrift, lxrx, stratRoss, stratShelf, stratPelagic, DepLipPct | int, transit |
|  | int, transit, ndrift, lxrx, stratShelf, stratPelagic, DepLipPct | int, transit |
|  | int, transit, ndrift, lxrx, stratShelf, stratPelagic, DepLipPct, stratShelfXDepLipPct | int, transit |
|  | int, transit, ndrift, lxrx, s1, DepLipPct, s1XDepLipPct | int, lxrx |
|  | int, transit, ndrift, lxrx | int, lxrx |
|  | int, transit, ndrift, lxrx, s1, s3, DepLipPct, s1XDepLipPct | int, lxrx |
|  | int, transit, ndrift, IceConc, lxrx | int, lxrx, IceConc |
|  | int, transit, ndrift, lxrx | int, lxrx |
|  | int, transit, ndrift, lxrx, s1, s3, DepLipPct, s1XDepLipPct | int, lxrx |
|  | int, transit, ndrift, lxrx, DepLipPct | int, lxrx |
|  | int, transit, ndrift, lxrx | int, lxrx, transit |
|  | int, transit, ndrift, lxrx, IceConc | int, lxrx, IceConc |
|  | int, transit, ndrift, lxrx | int, lxrx |
|  | int, transit, ndrift, lxrx, s1, s3, s1XDepLipPct, DepLipPct | int, lxrx, transit |
|  | int, transit, ndrift, lxrx, stratShelf, StratPelagic, DepLipPct, stratShelfXDepLipPct | int, lxrx, transit |
|  | int, transit, ndrift, lxrx, DepLipPct | int, lxrx, transit |
|  | int, transit, ndrift, lxrx, s1, s3, s1XDepLipPct, DepLipPct | int, lxrx, transit |
|  | int, transit, ndrift, lxrx, stratShelf, StratPelagic, DepLipPct, stratShelfXDepLipPct | int, lxrx, transit |
|  | int, transit, ndrift, lxrx, s1, s3, DepLipPct, s1XDepLipPct | int, lxrx, transit |
|  | int, transit, ndrift, lxrx, stratShelf, stratPelagic, DepLipPct, stratShelfXDepLipPct | int, lxrx, transit |
|  | int, transit, ndrift, lxrx, stratRossS1, stratRossS2, stratRossS3, stratShelfS1, stratShelfS2, stratShelfS3, stratPelagicS1, stratPelagicS3, DepLipPct, stratPelagicS1XDepLipPct, stratRossS1XDepLipPct, stratShelfS1XDepLipPct | int, lxrx, transit |
|  | int, transit, ndrift, lxrx, stratRossS1, stratRossS2, stratRossS3, stratShelfS1, stratShelfS2, stratShelfS3, stratPelagicS1, stratPelagicS3, DepLipPct | int, lxrx, transit |


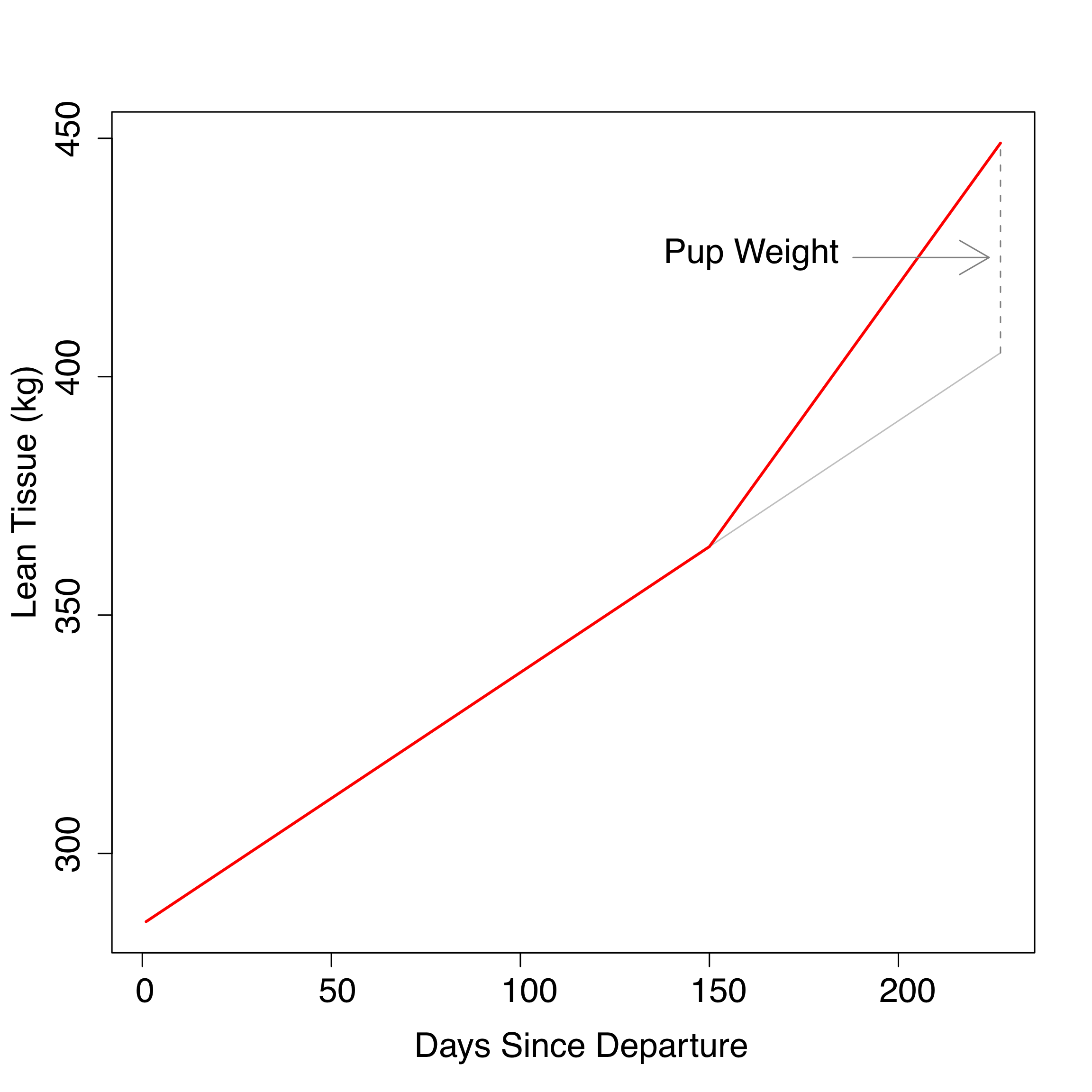


Figure S3.1. Graphical representation of the assumption we used for lean tissue gain. Lean tissue is linearly increasing throughout the first 2/3’s of the trip. During the last third, the lean tissue increases linearly but at an increased rate to account for the weight of the pup.


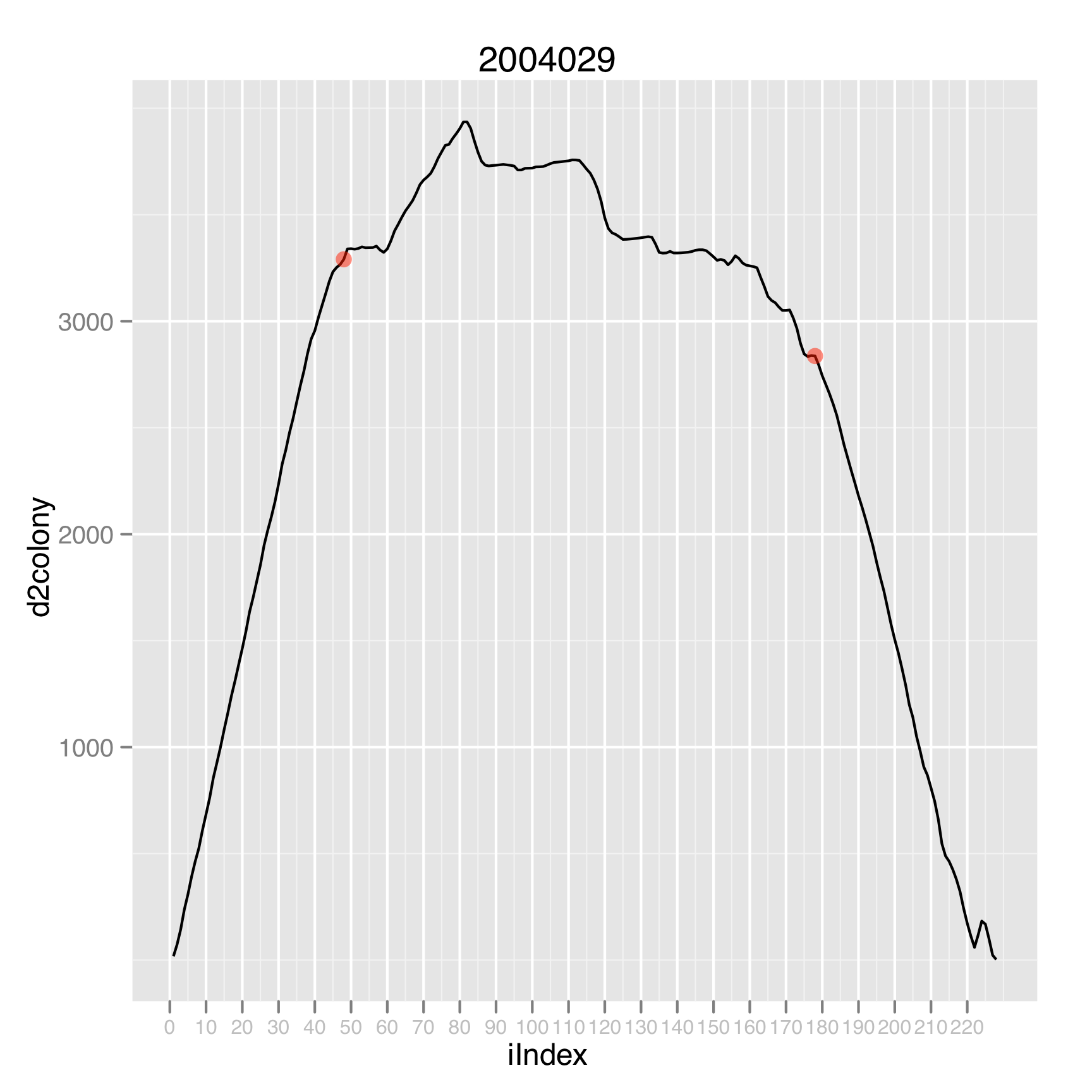


Figure S3.2. Time series of daily distance to colony values (km) for one individual northern elephant seal. Values on the x-axis are days since departure. Red circles indicate where we denoted the switch between each of the three behavioral states.


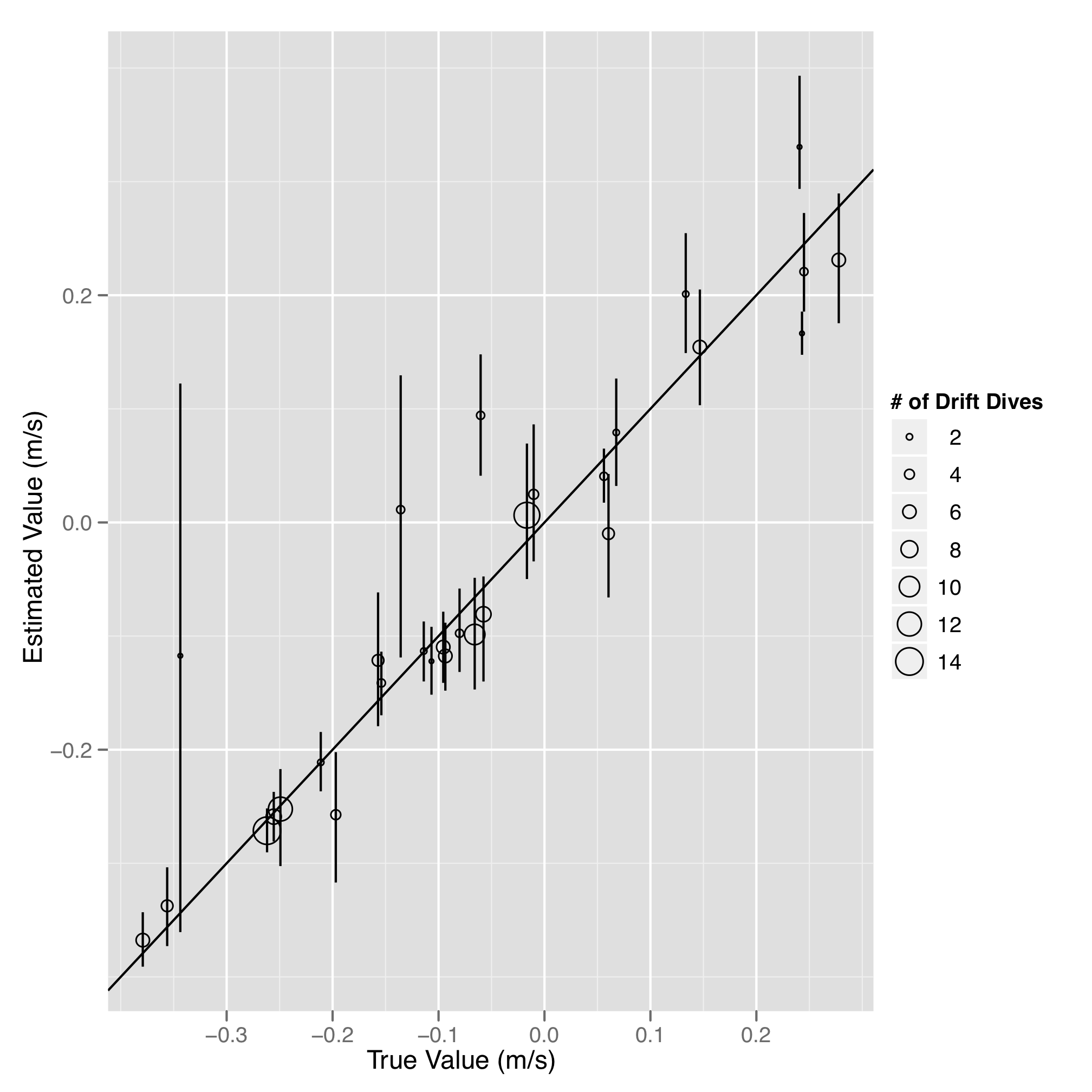


Figure S3.3. Results from the cross-validation exercise for the drift dive data indicate very good agreement between known values and estimates from the model. The plot shows true values on the x-axis that were removed and treated as missing in the model. Results on the y-axis show estimates of that value from model output. Vertical lines cover the 95% Bayesian Credible Interval. Where the BCI does not cover the 1:1 line, it is typically in cases where there were very few drift dives.


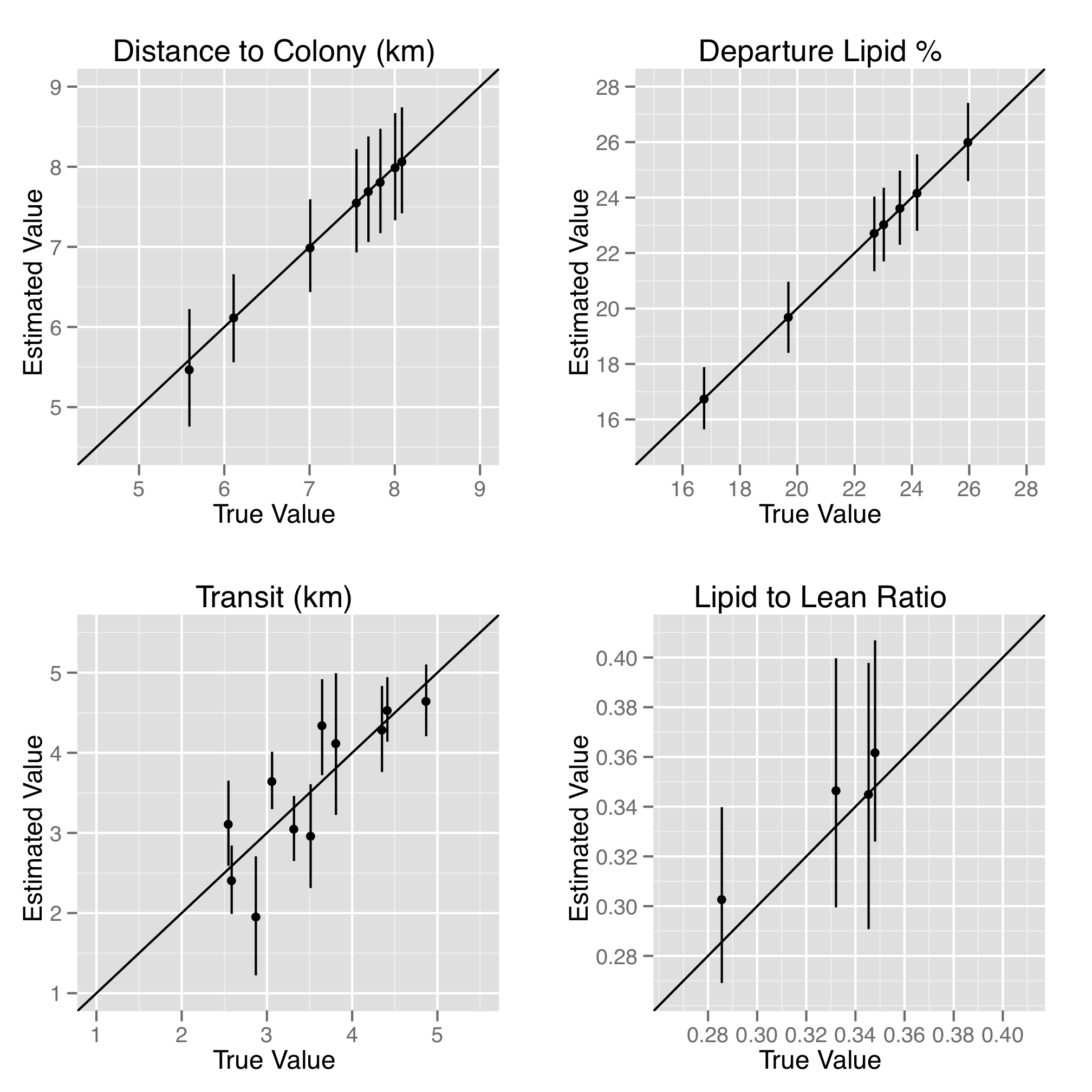


Figure S3.4. Results from the cross-validation exercise for missing environmental covariates also indicate very good performance of the model.
