# Supplementary figures and images for: Estimating resource acquisition and at-sea body condition of a marine predator
Source: J Anim Ecol. 2013 Jul 19;82(6):1300–15. doi: 10.1111/1365-2656.12102 (PMC4028992; doi:10.1111/1365-2656.12102)

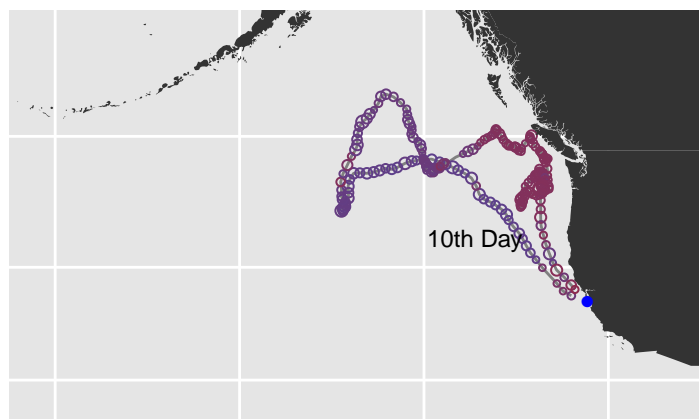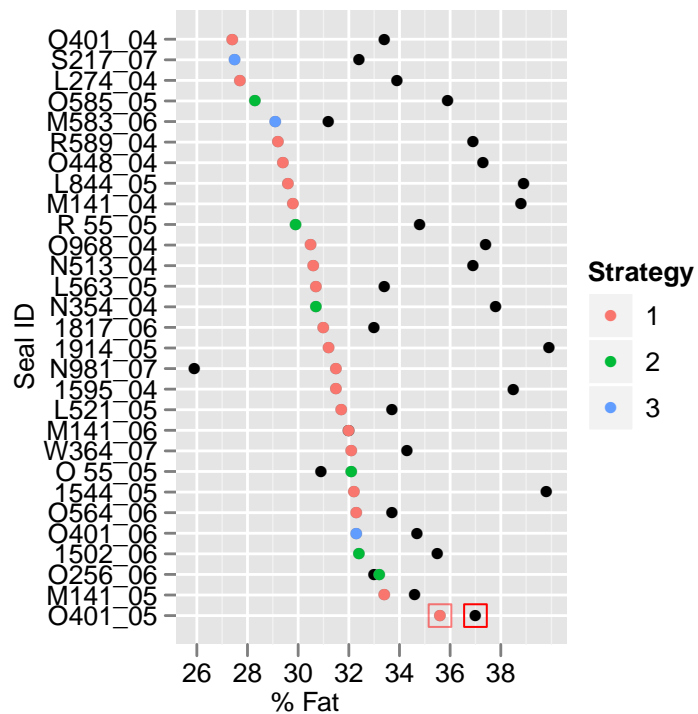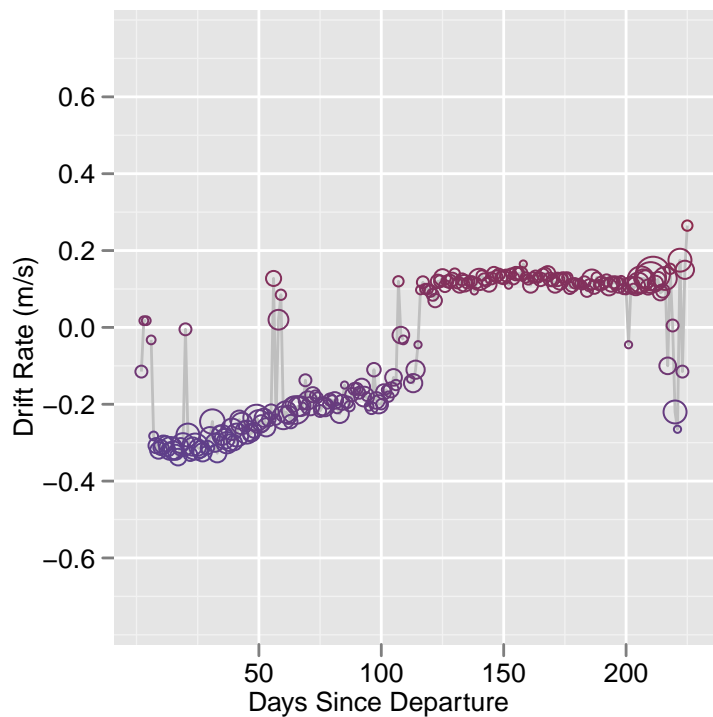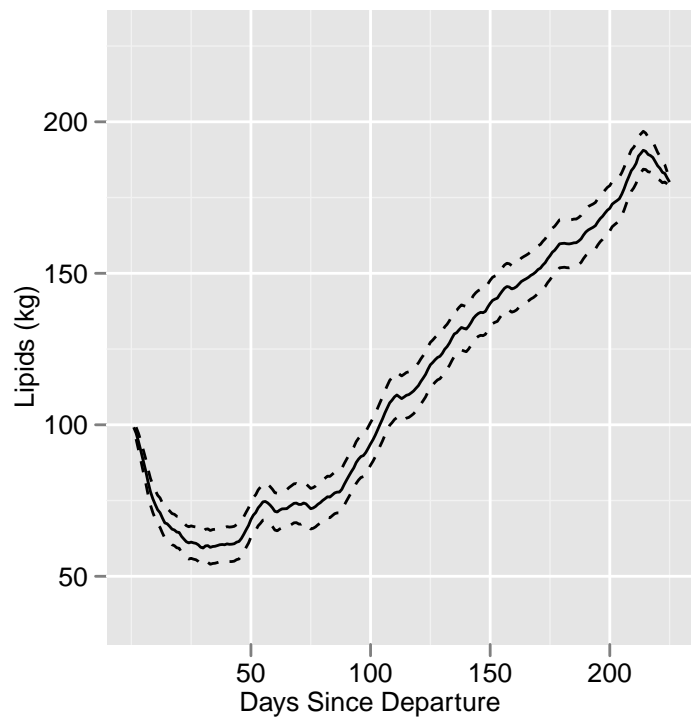

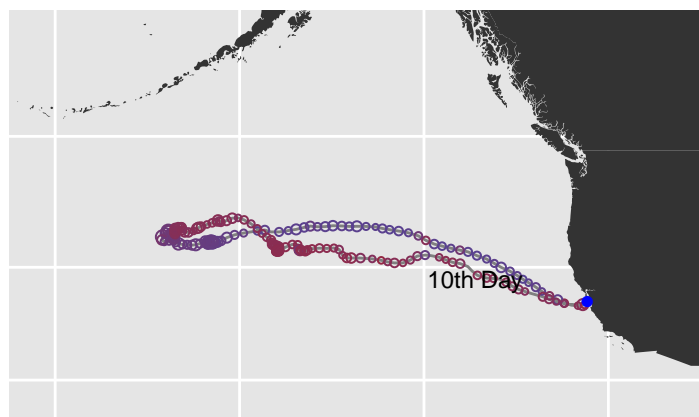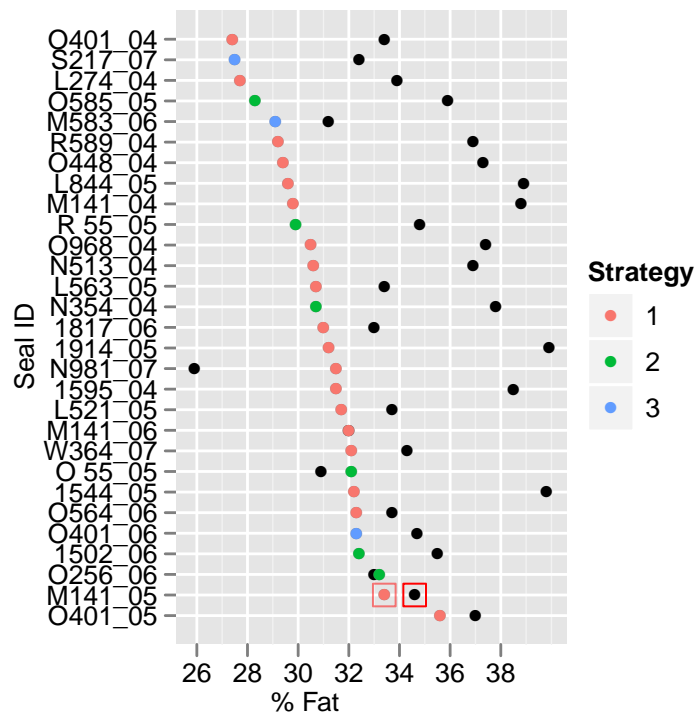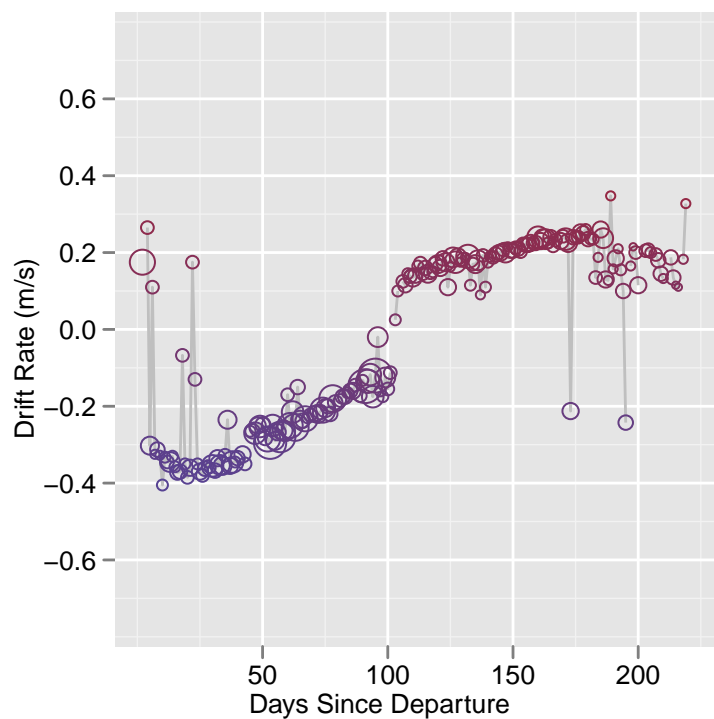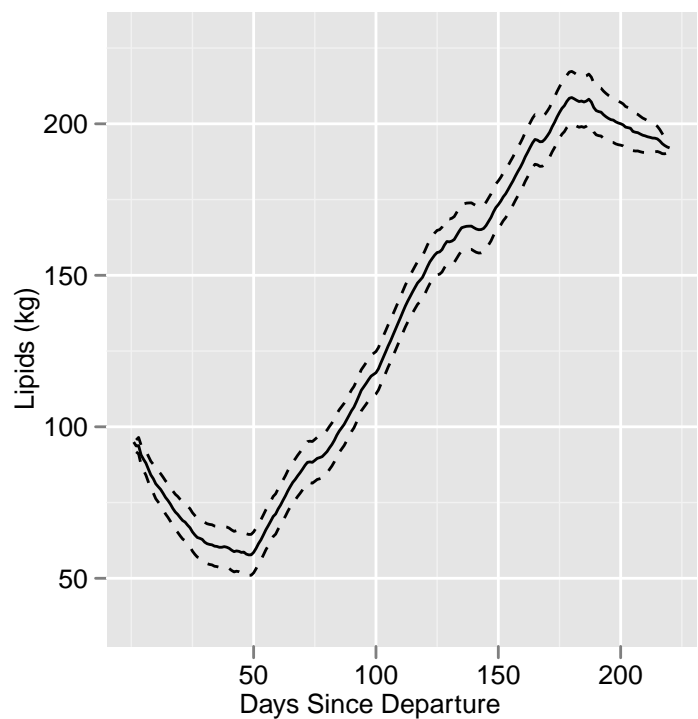

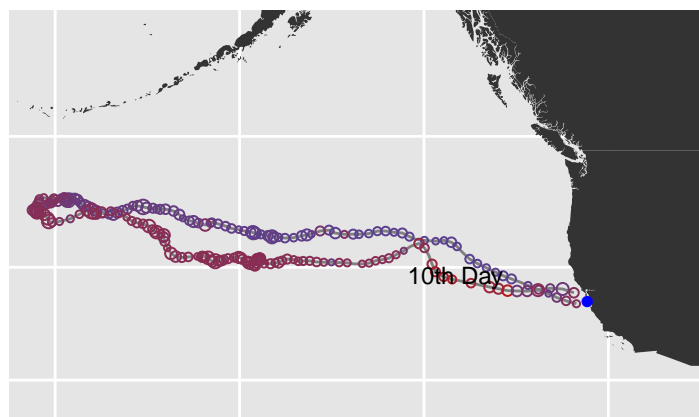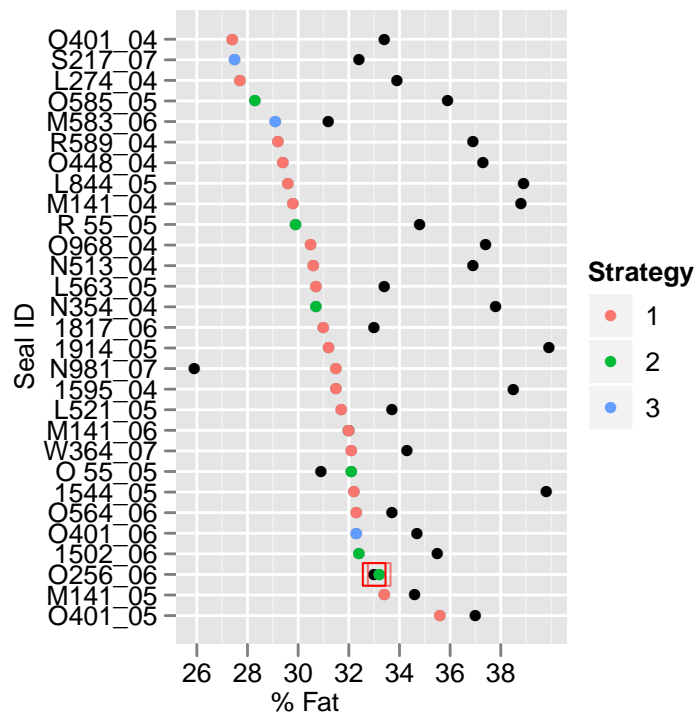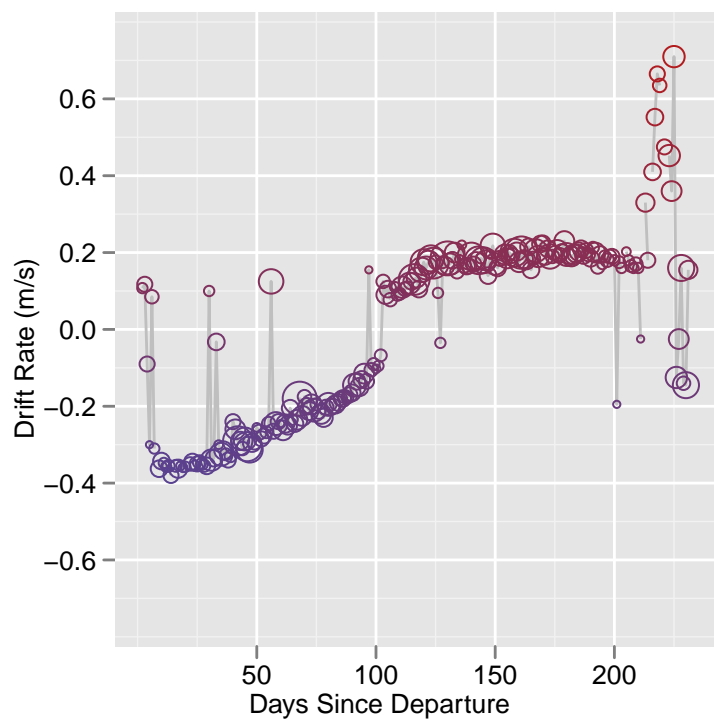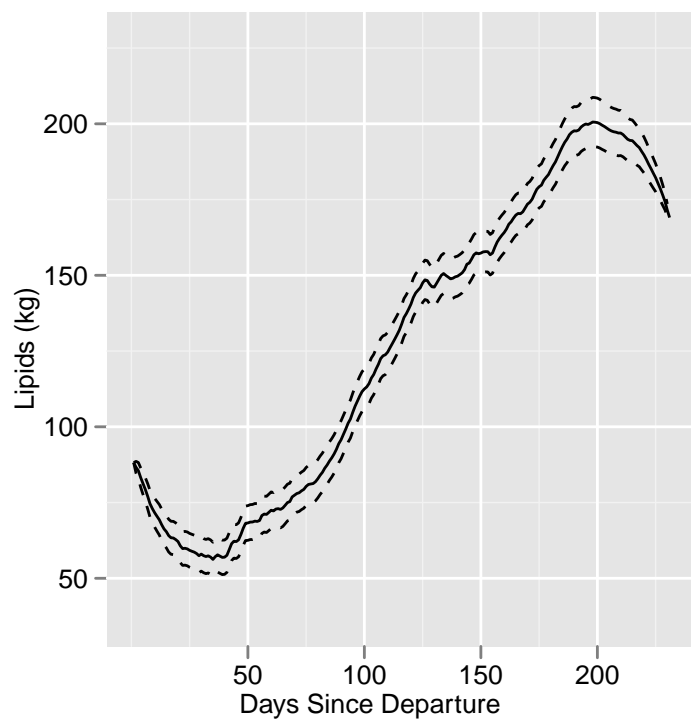

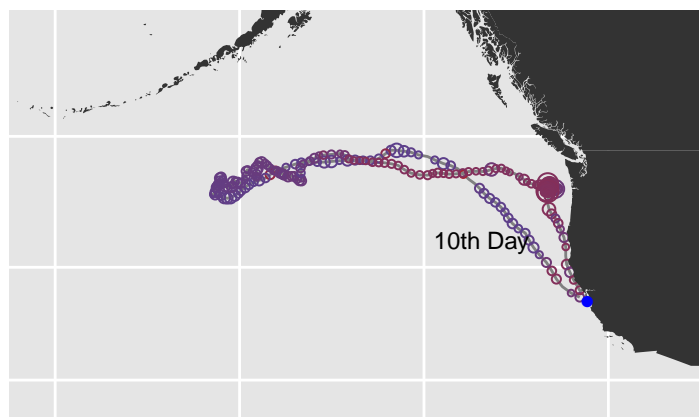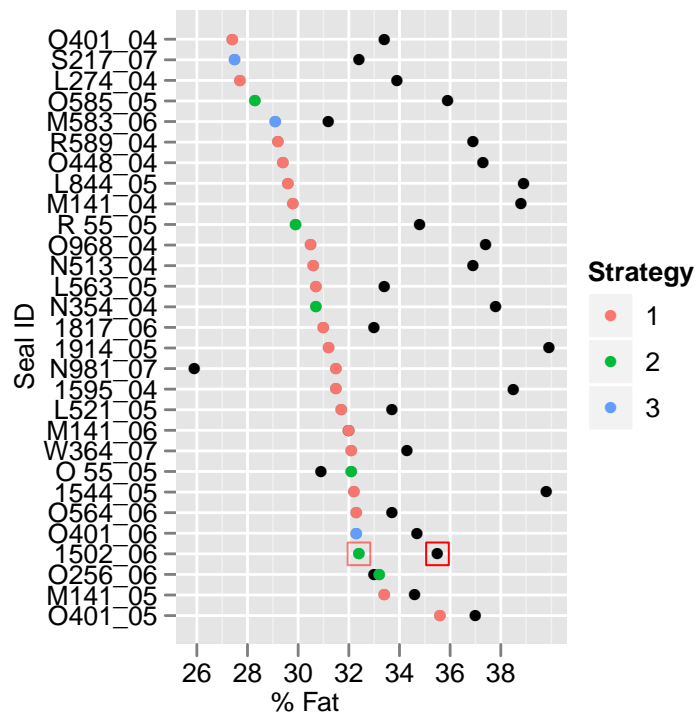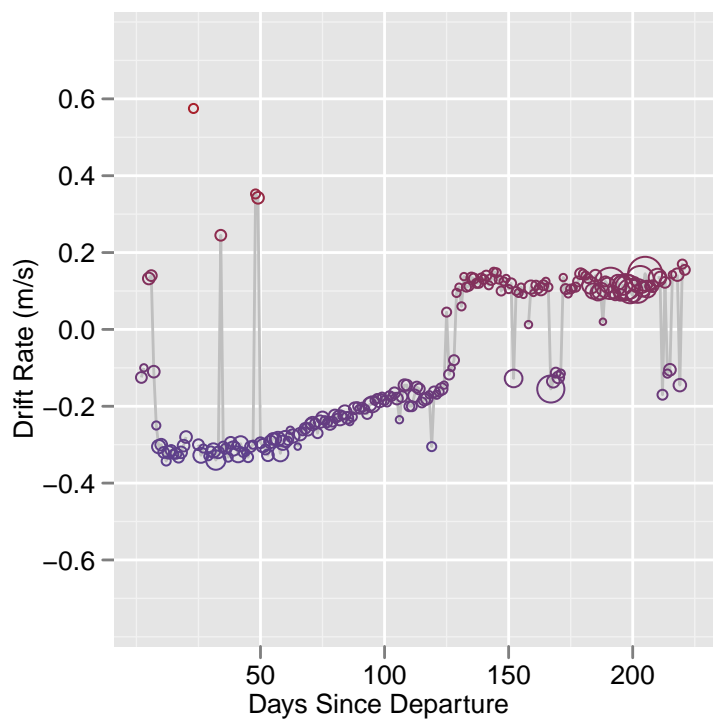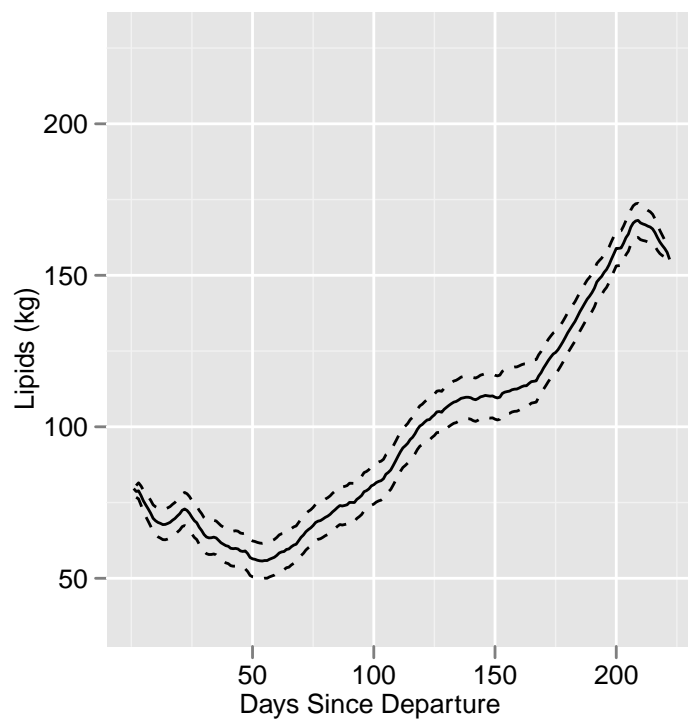

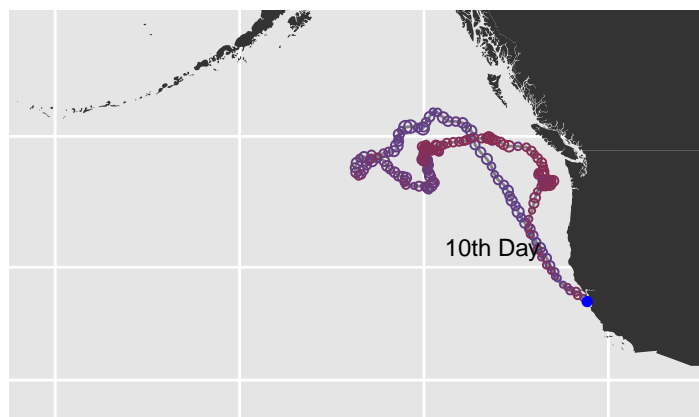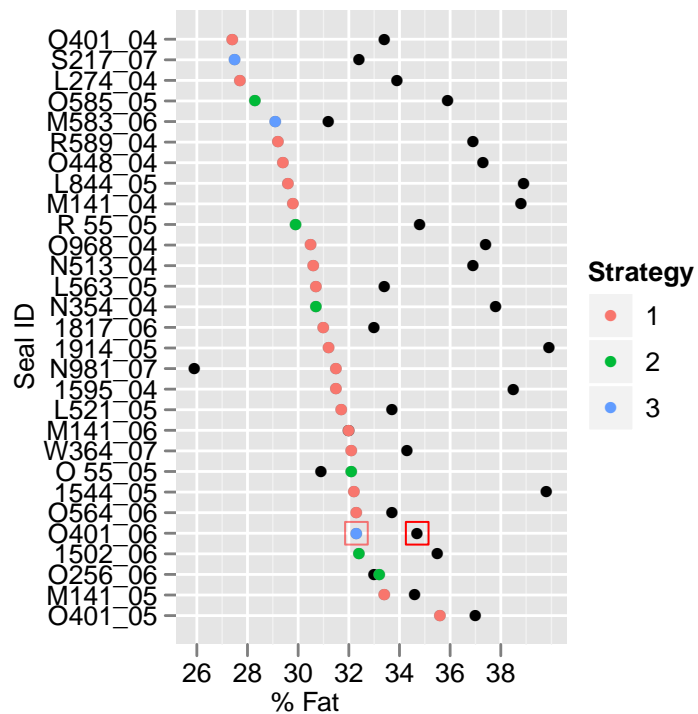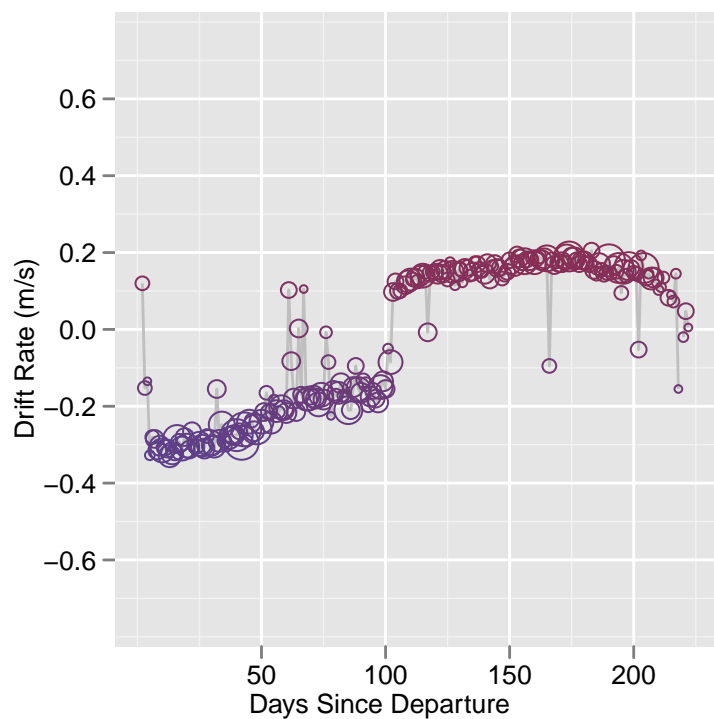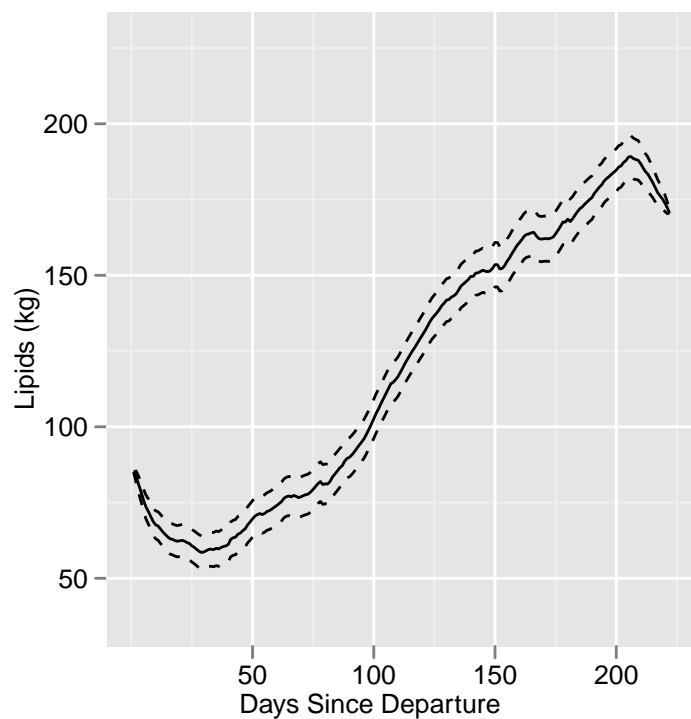

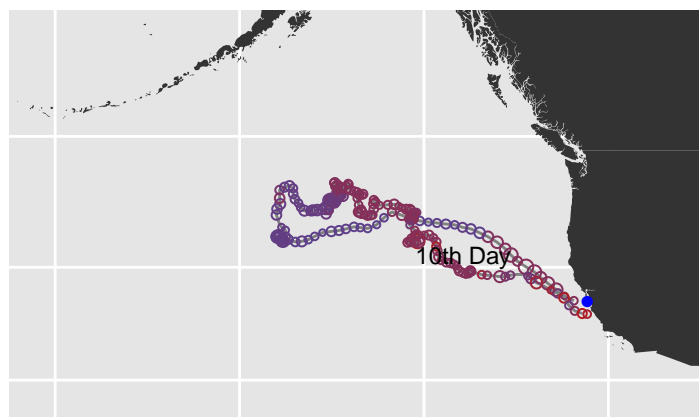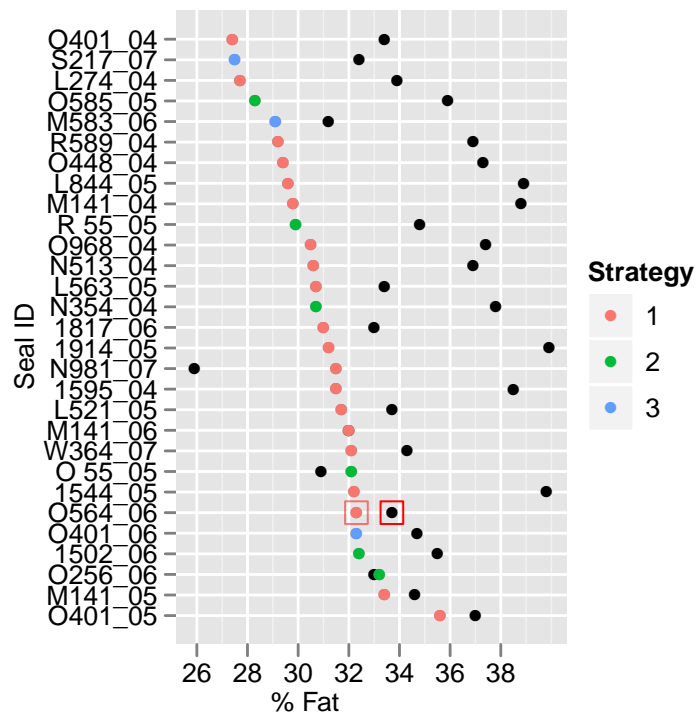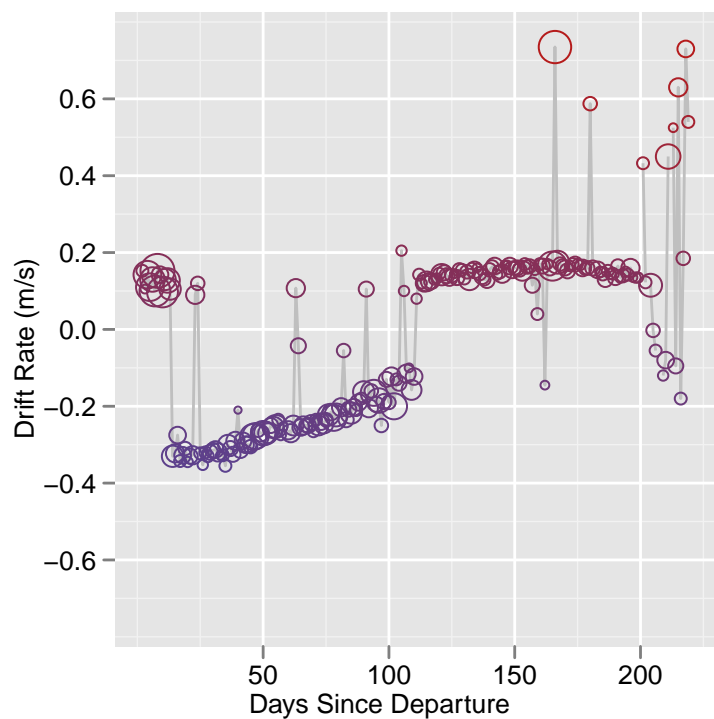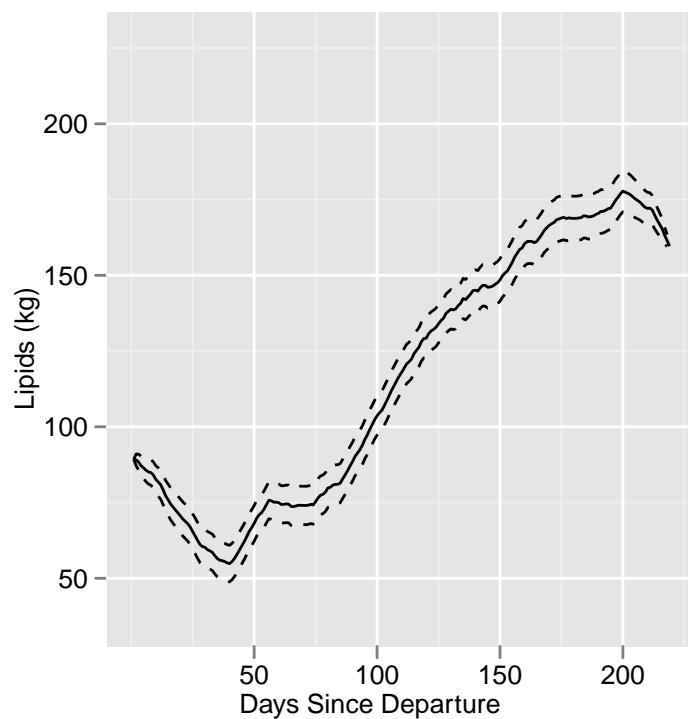

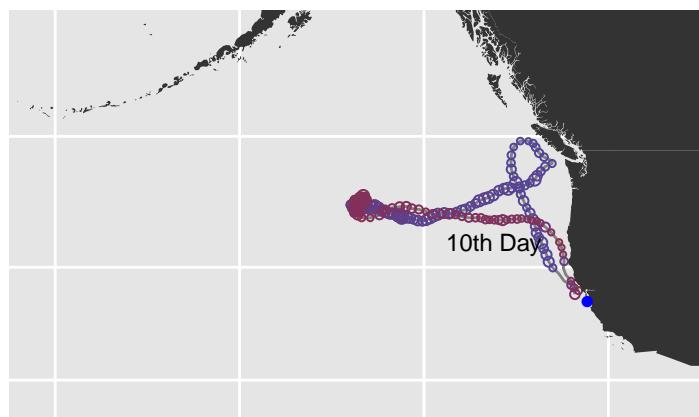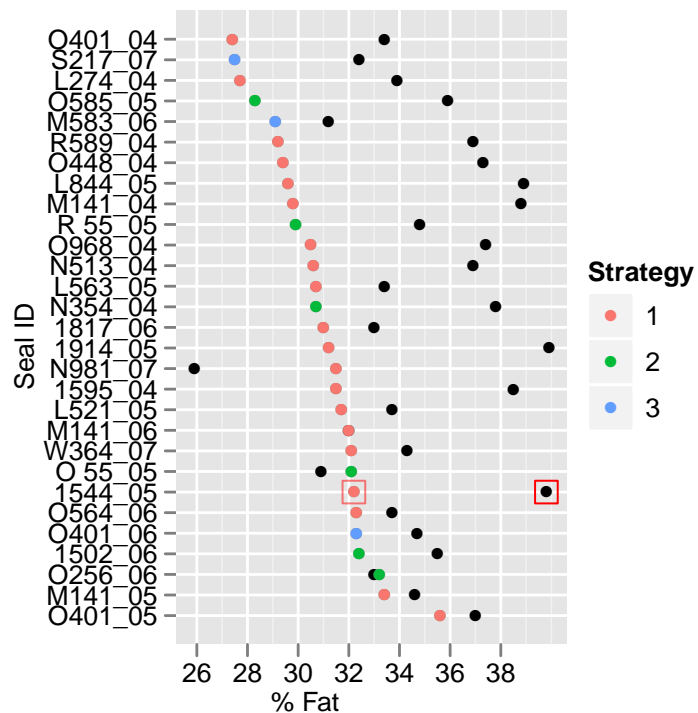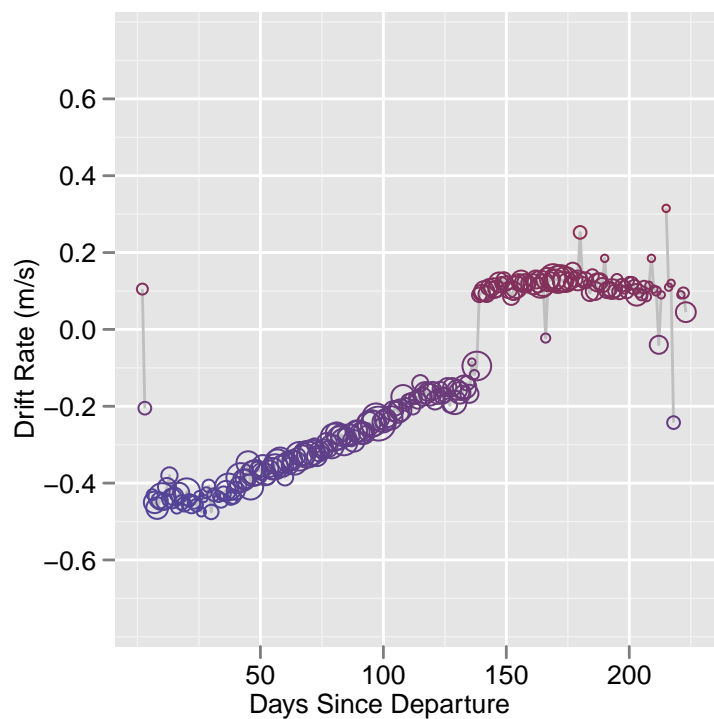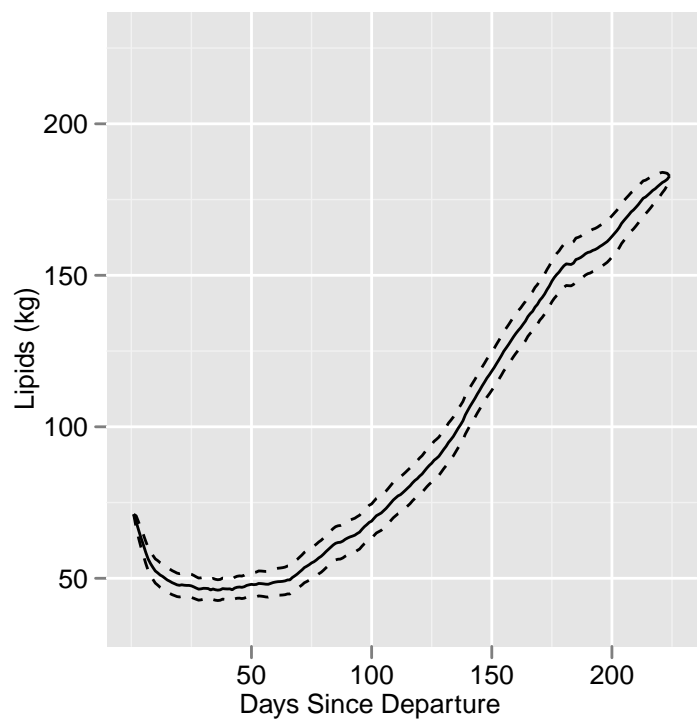

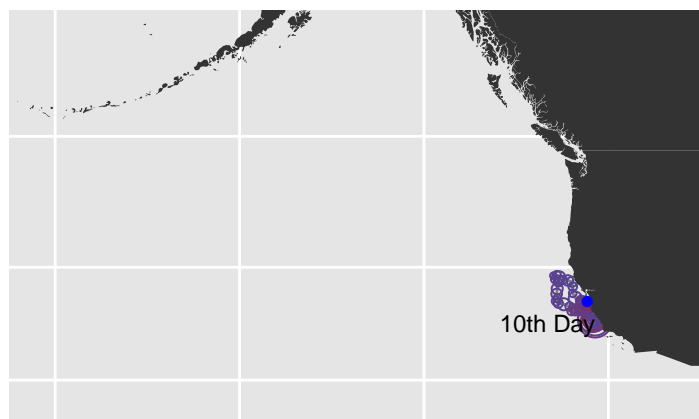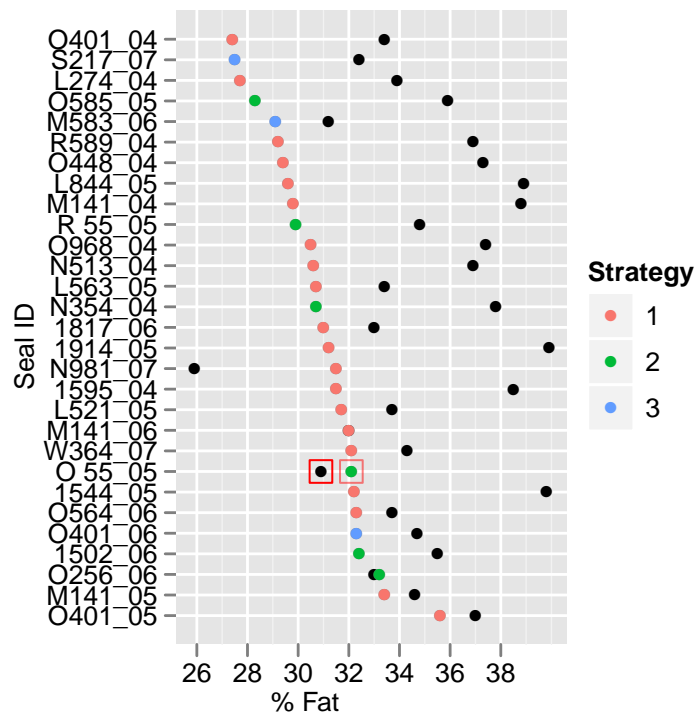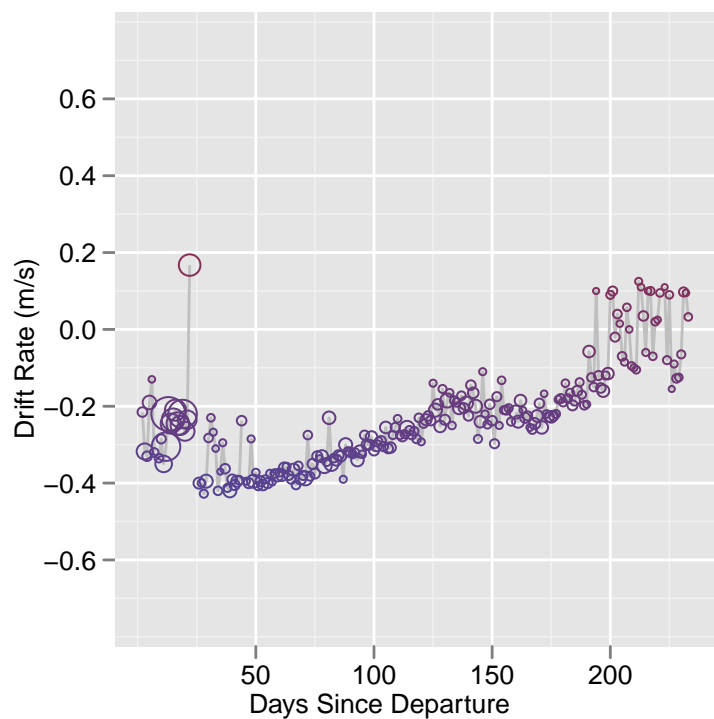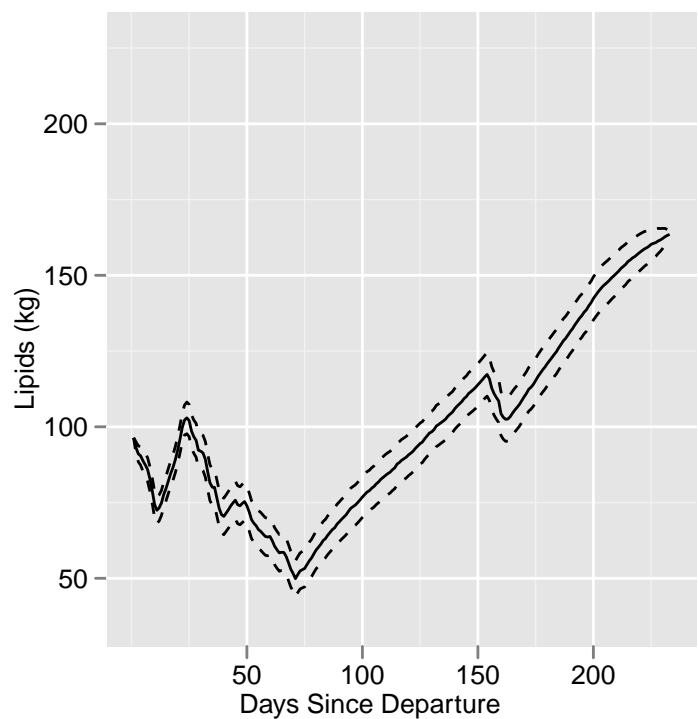

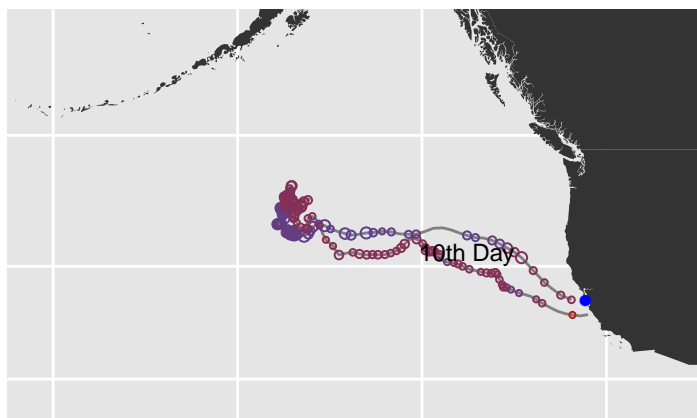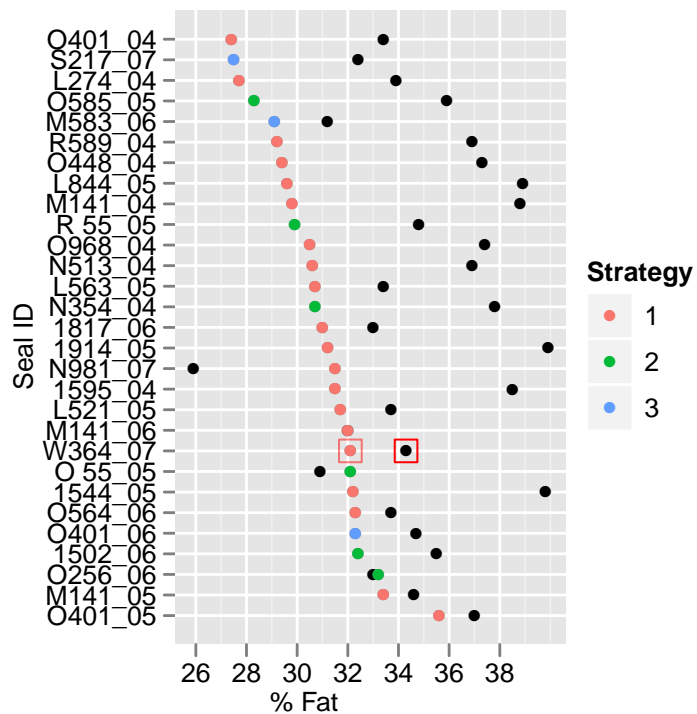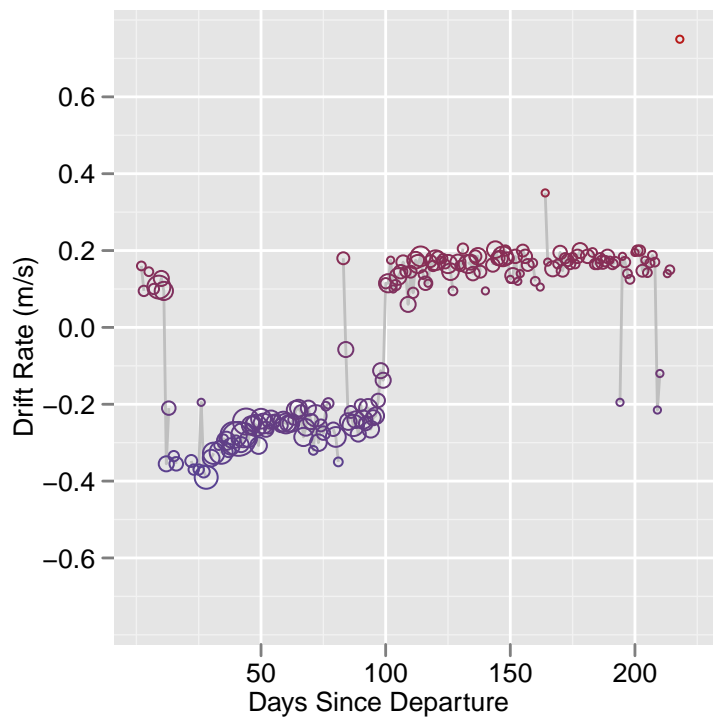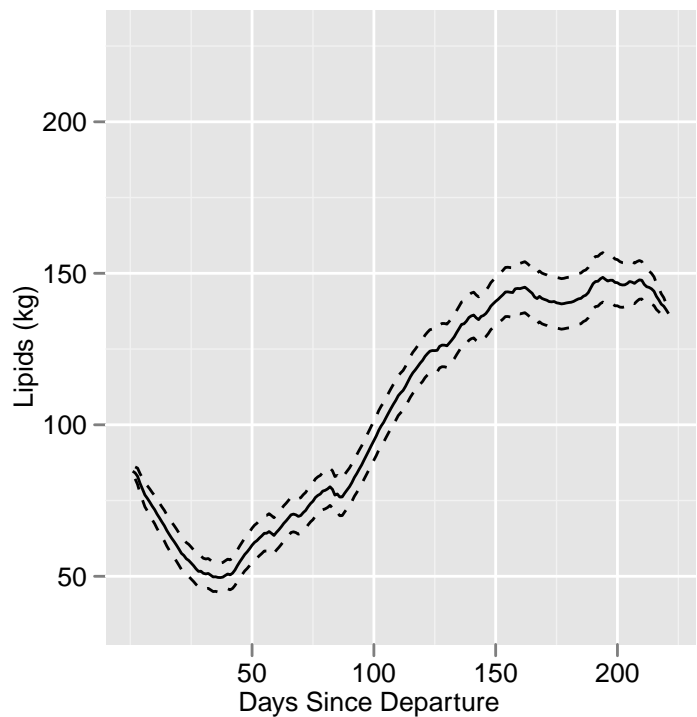

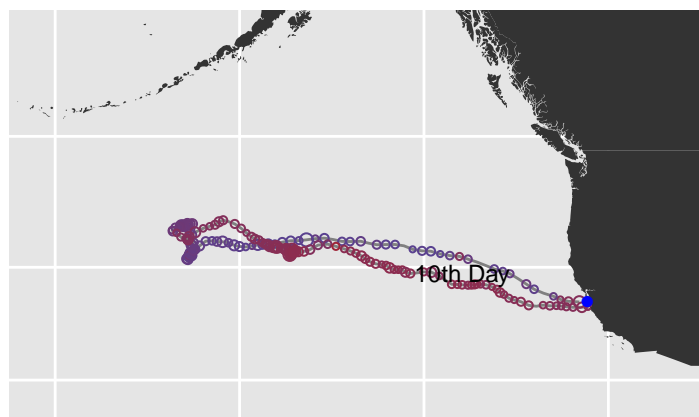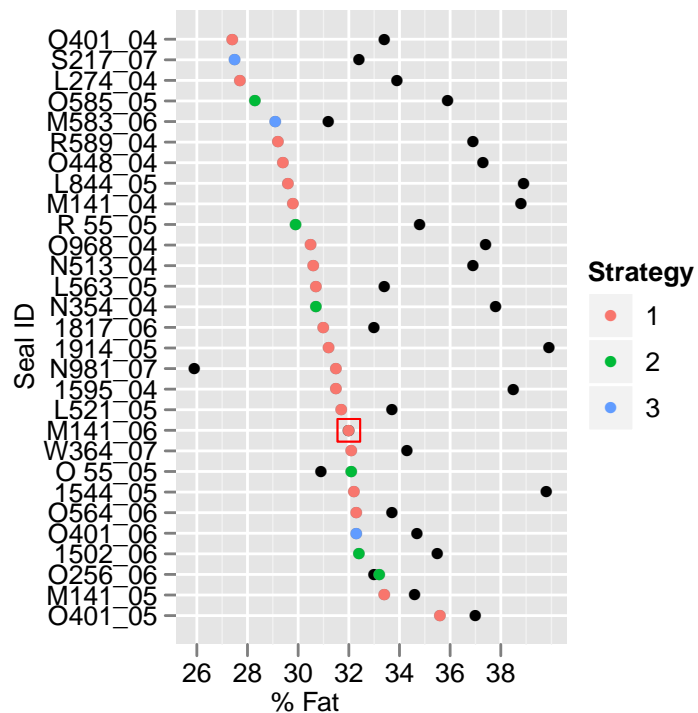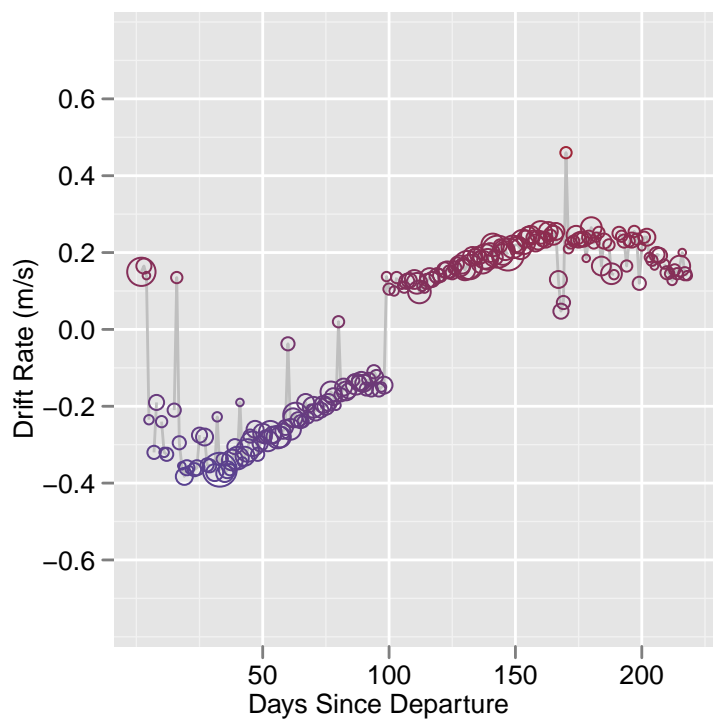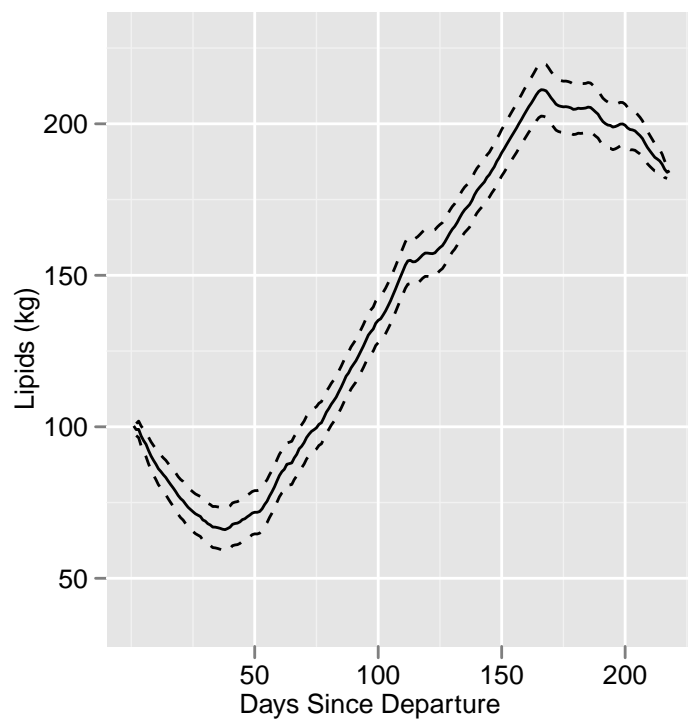

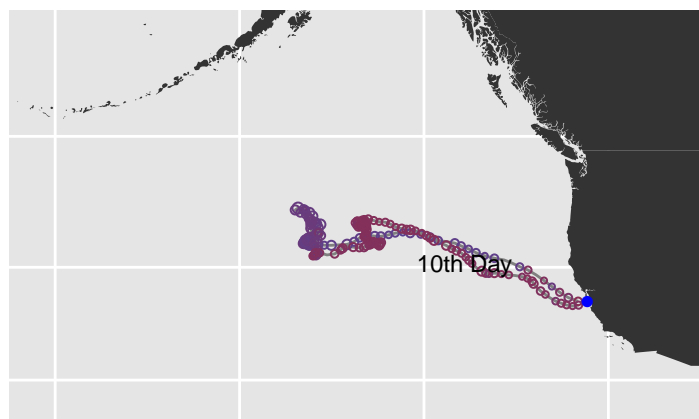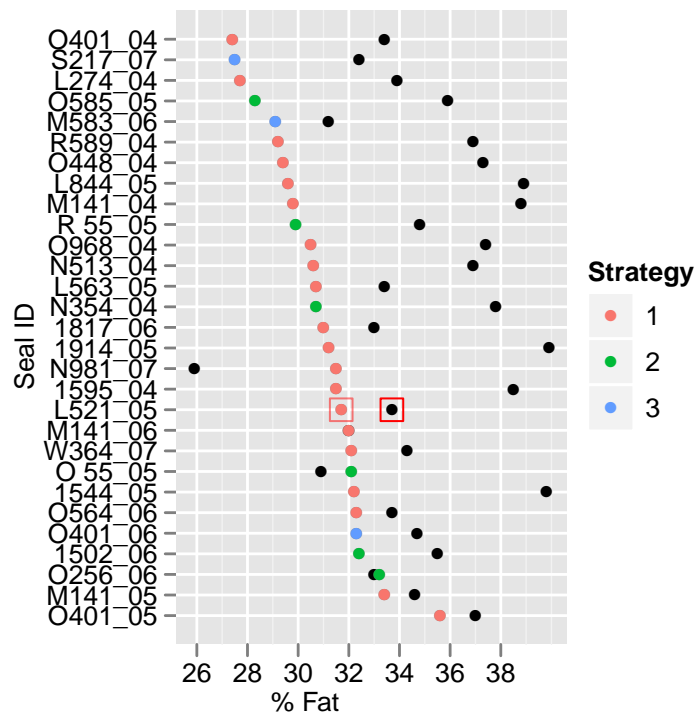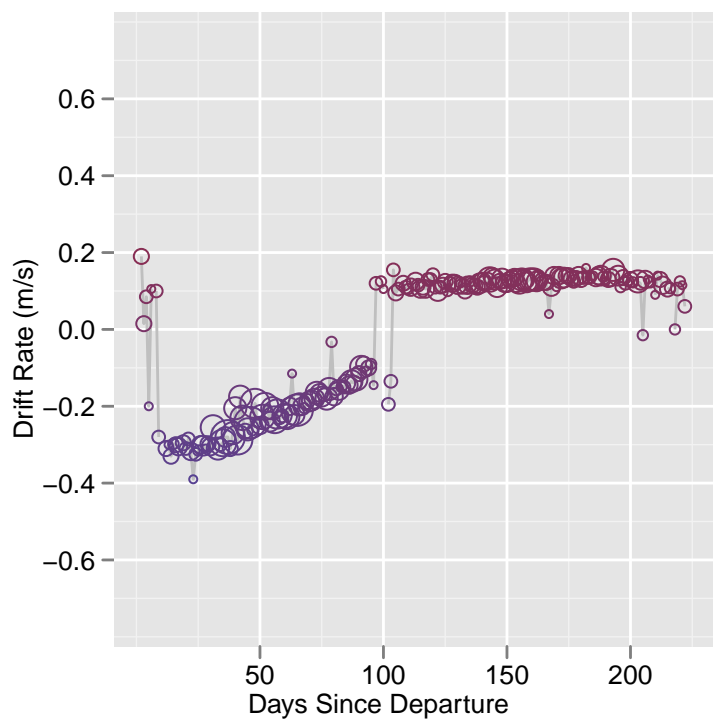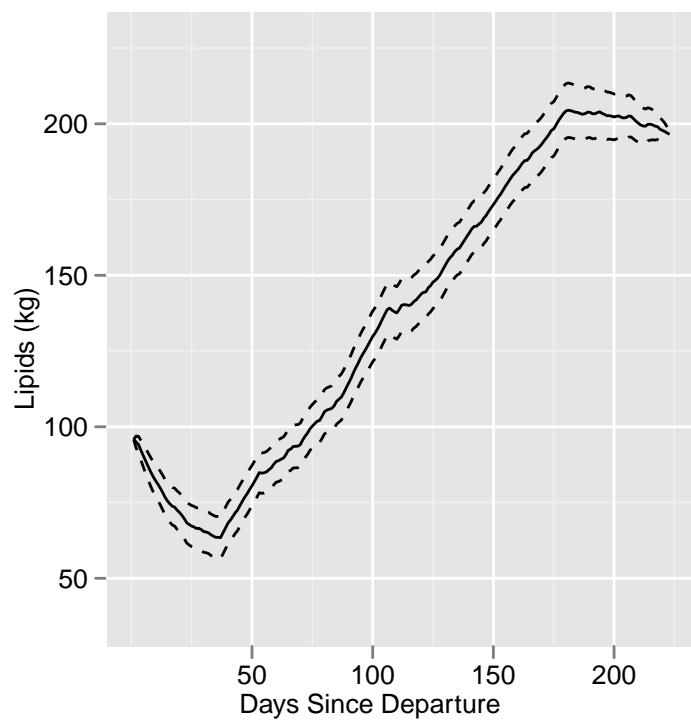

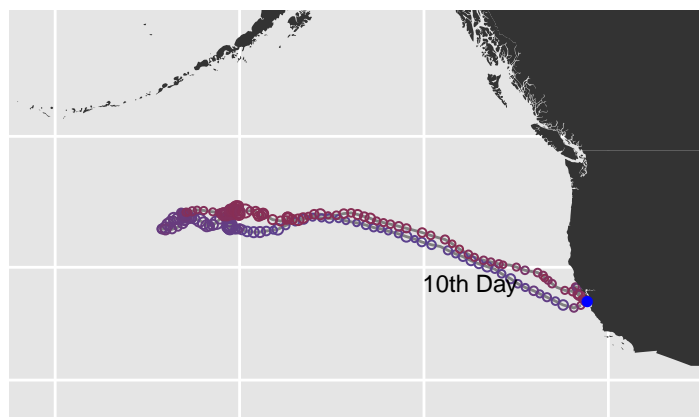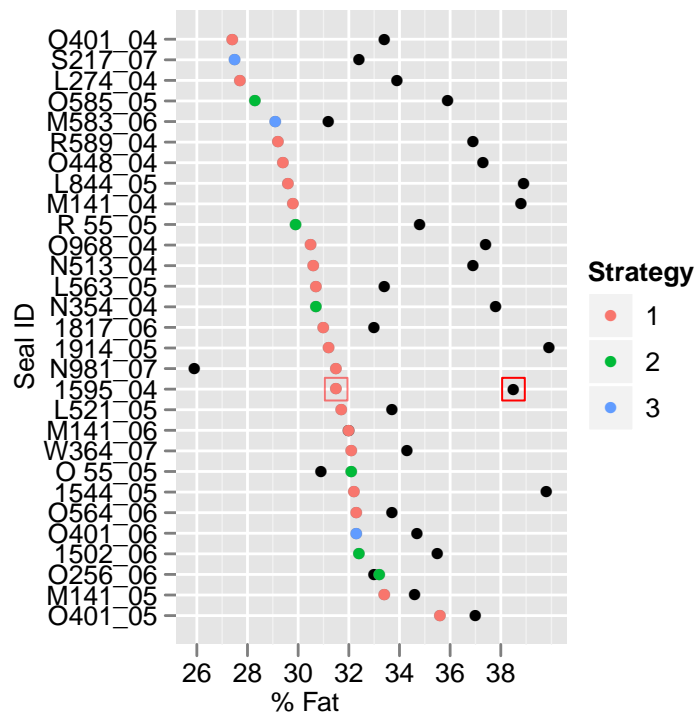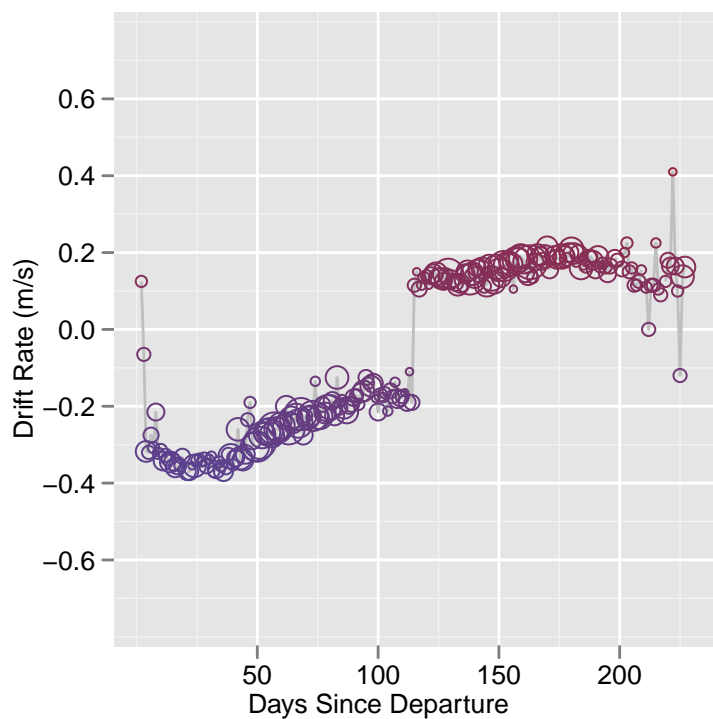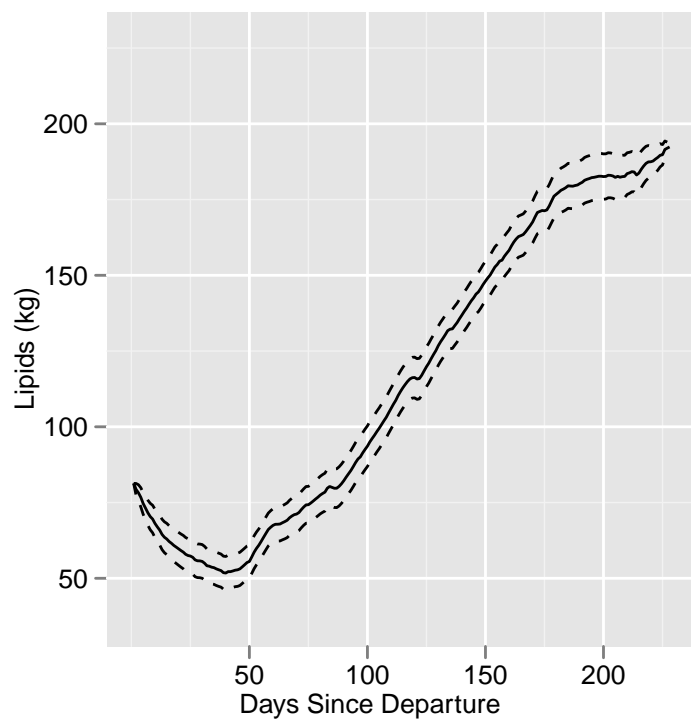

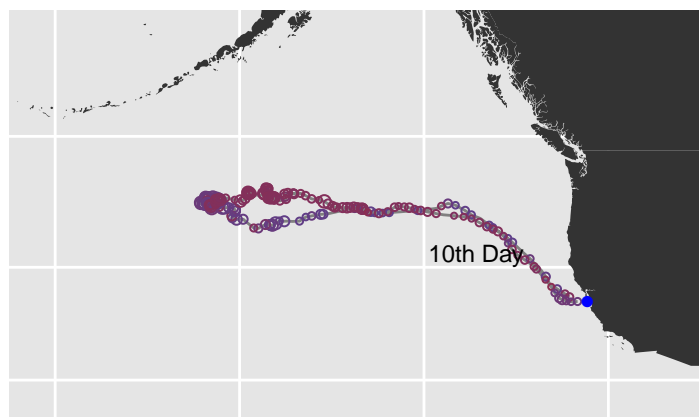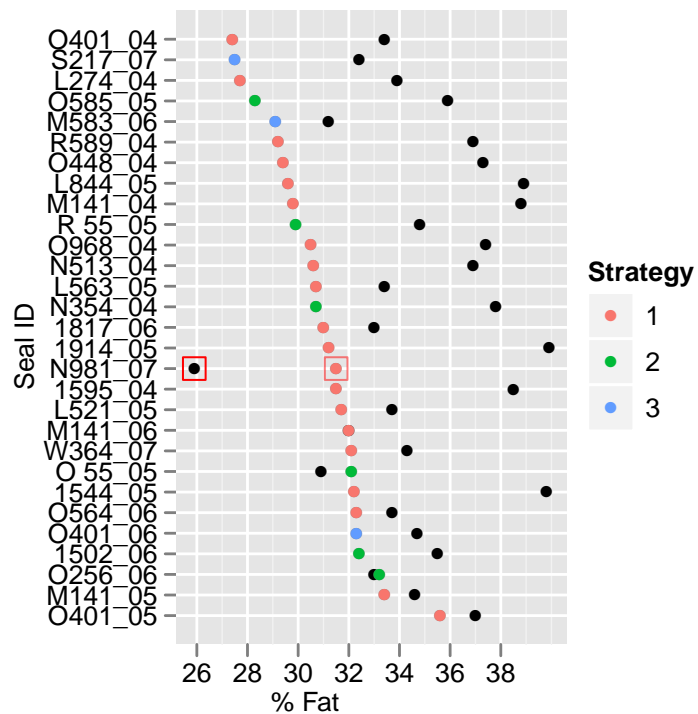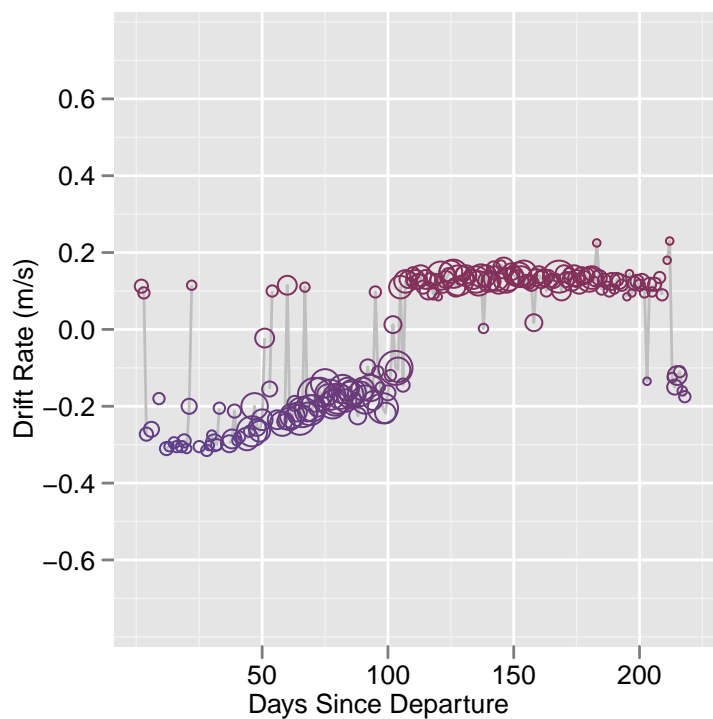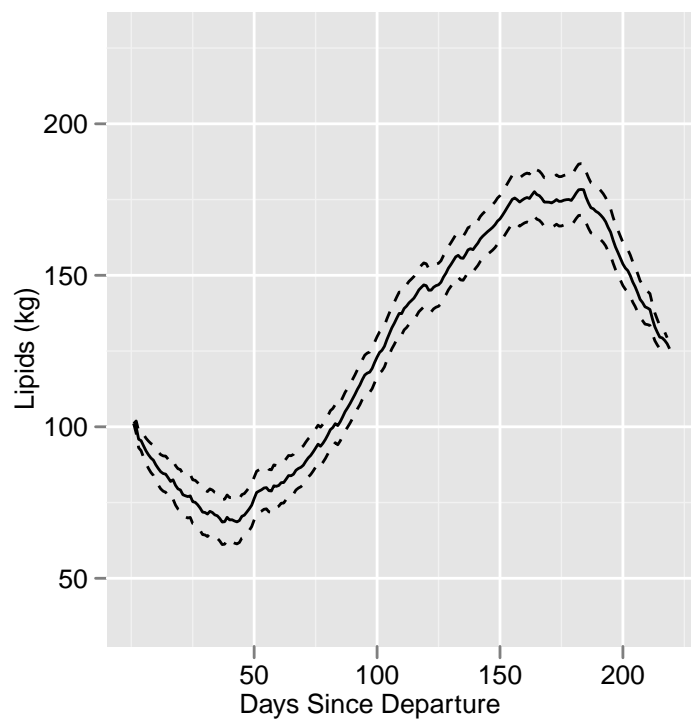

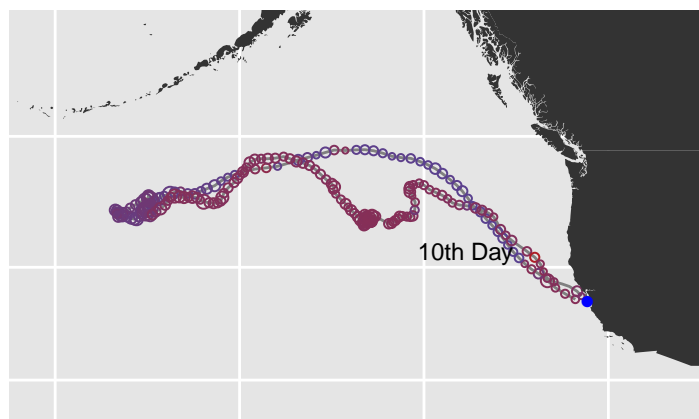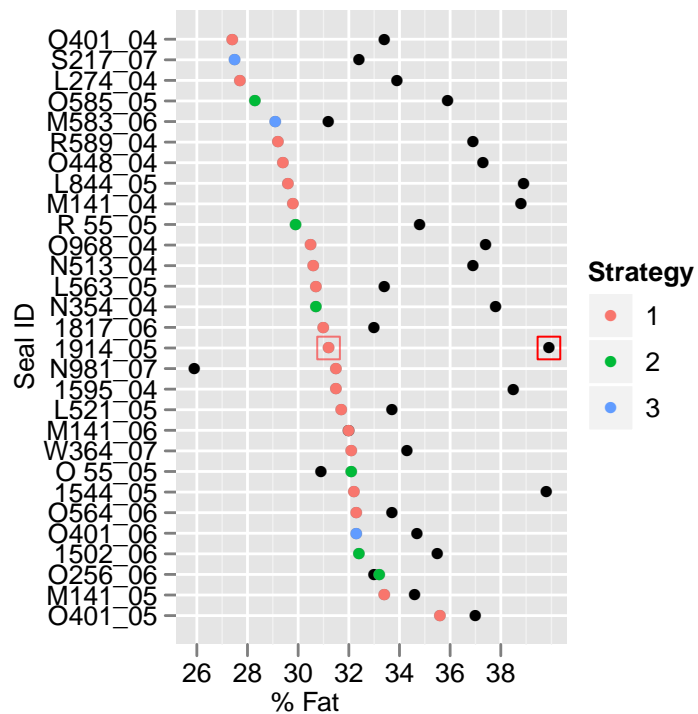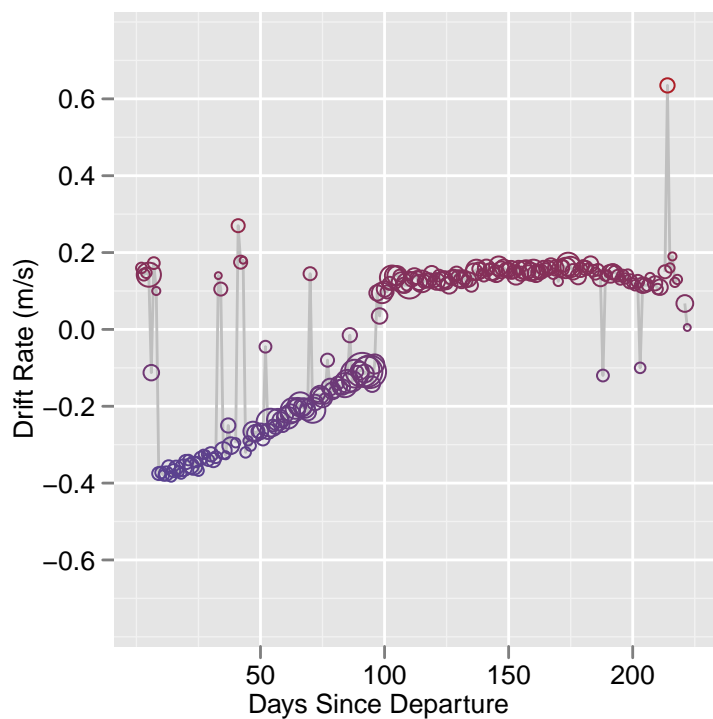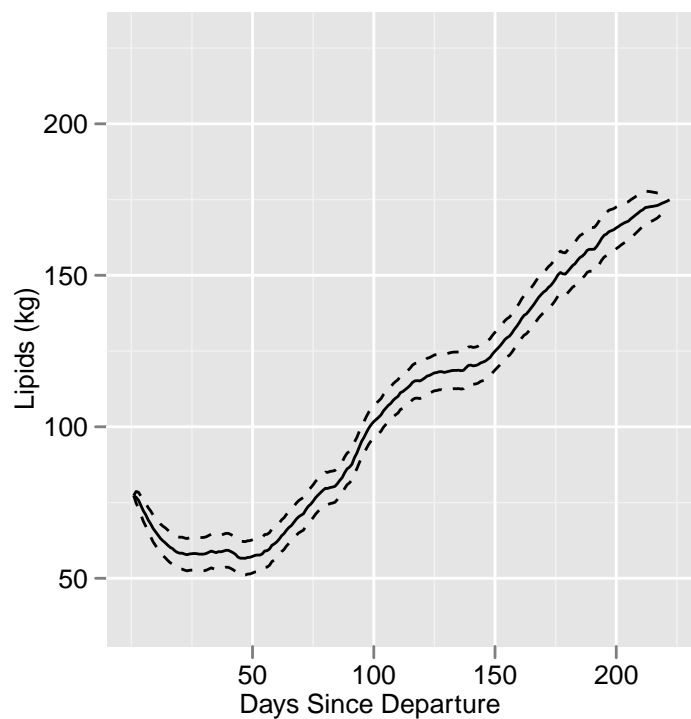

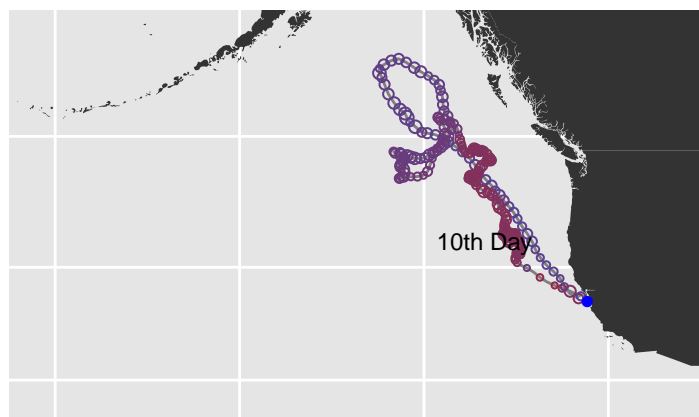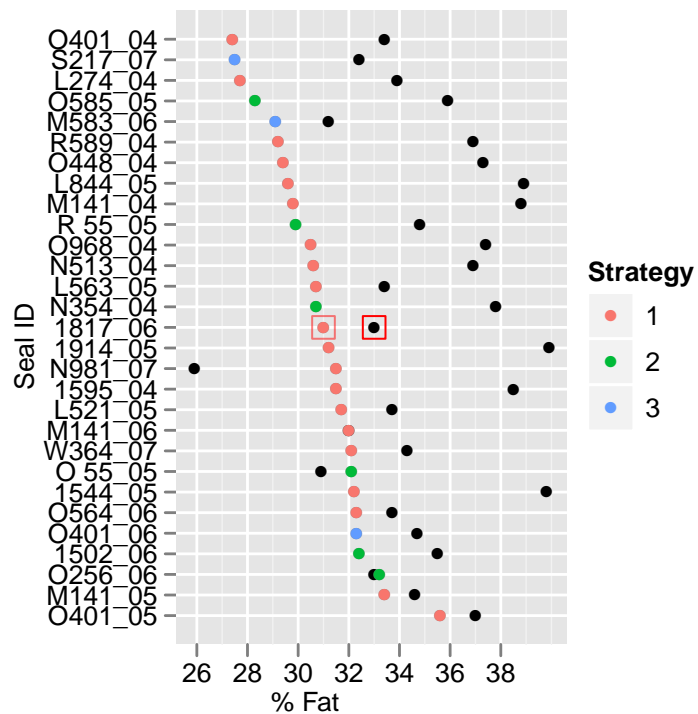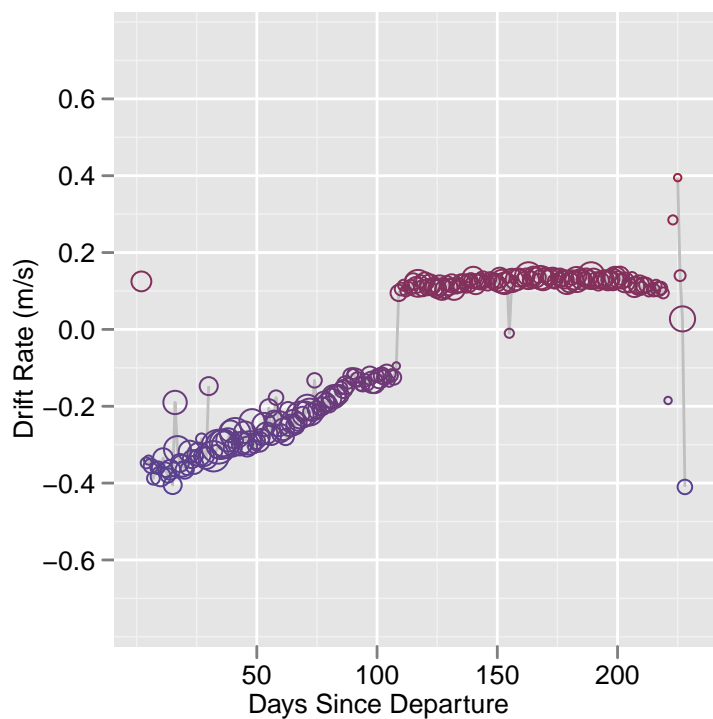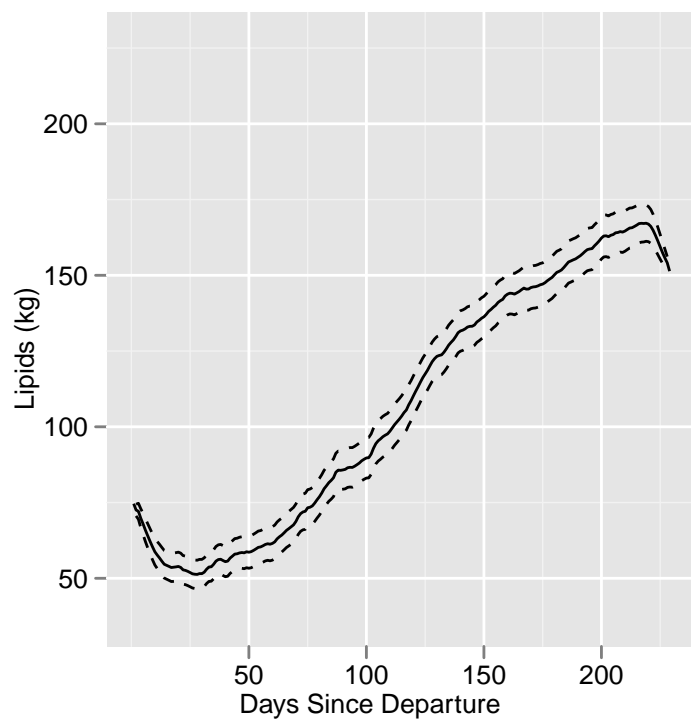

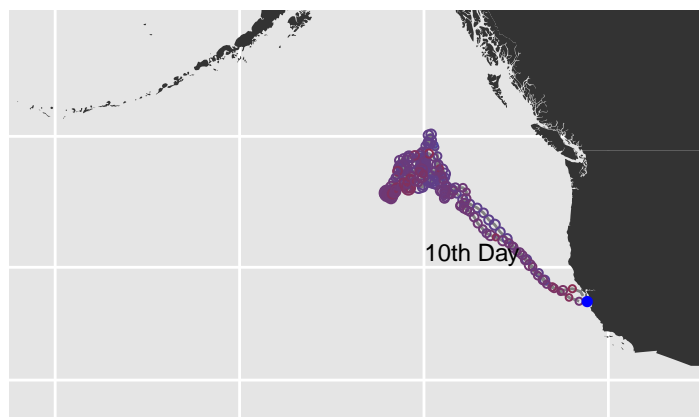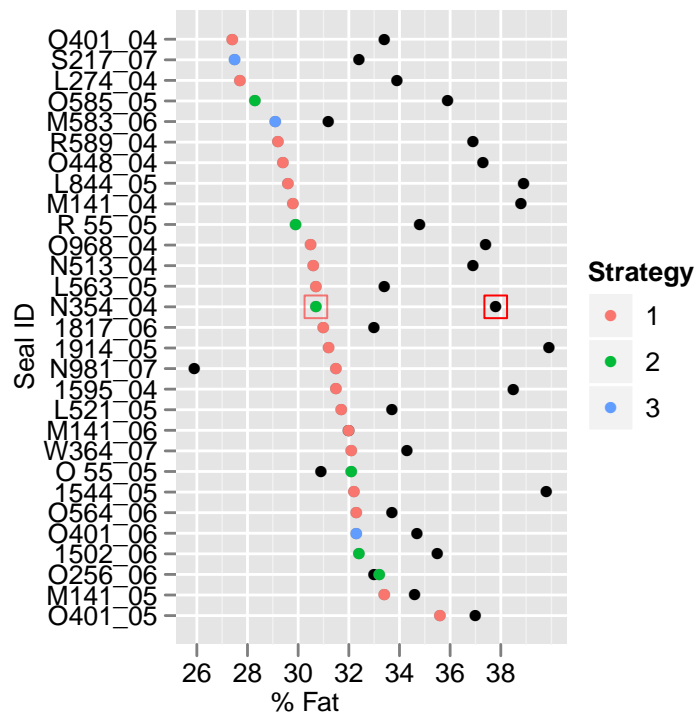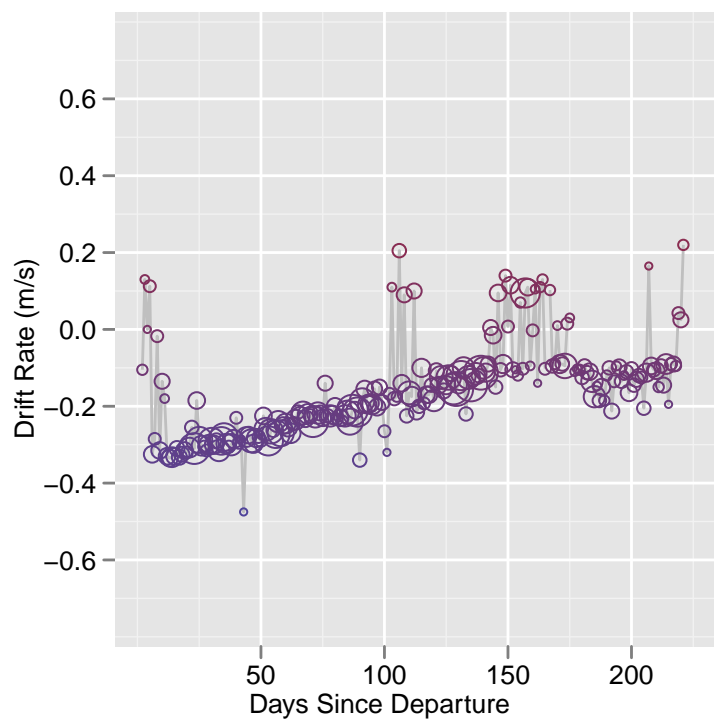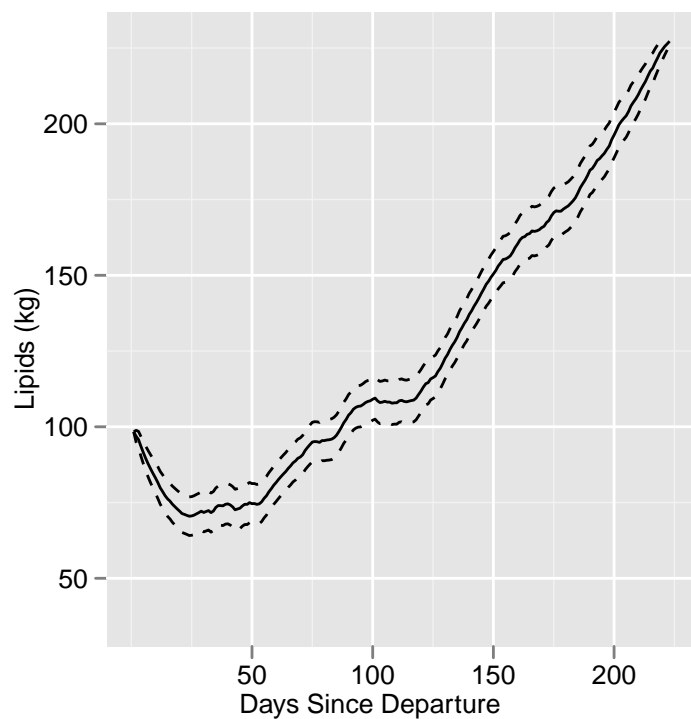

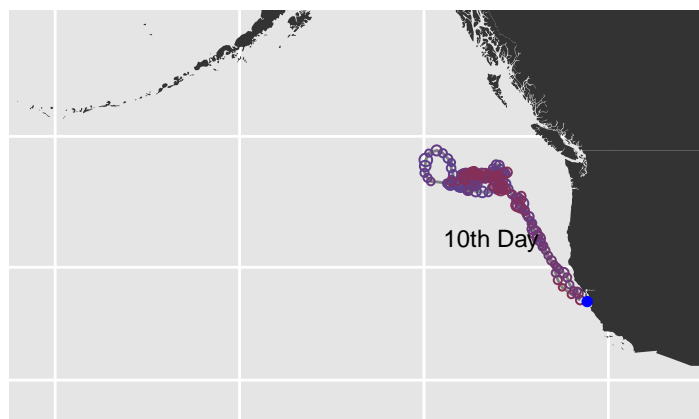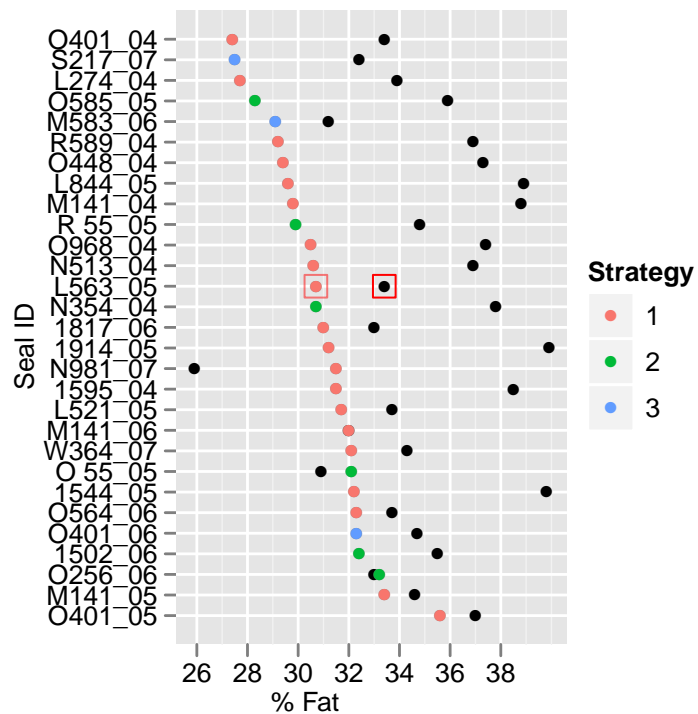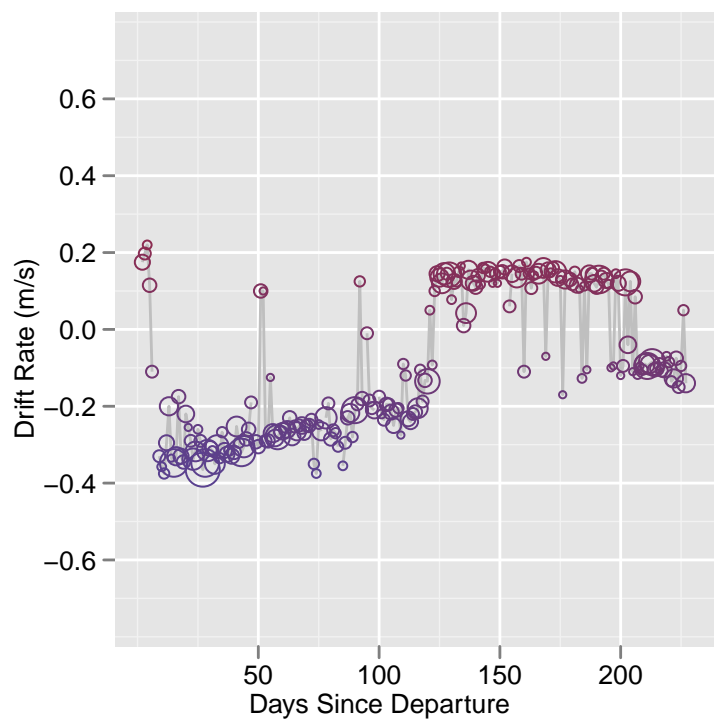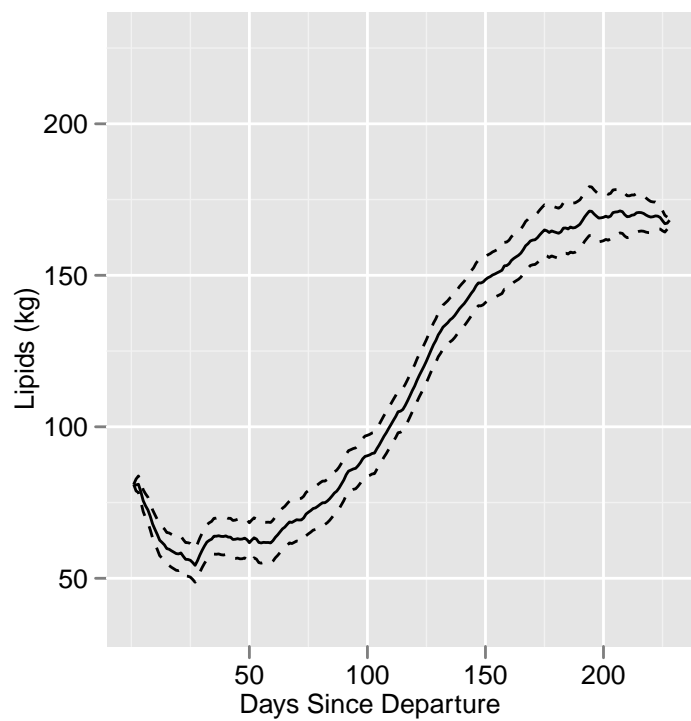

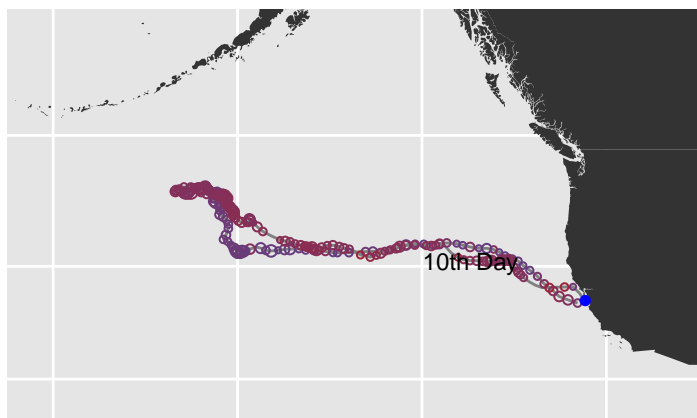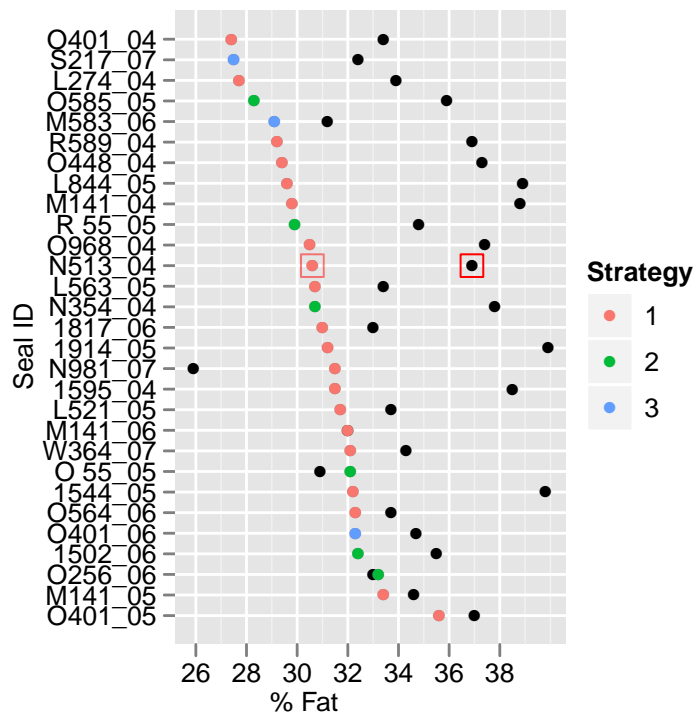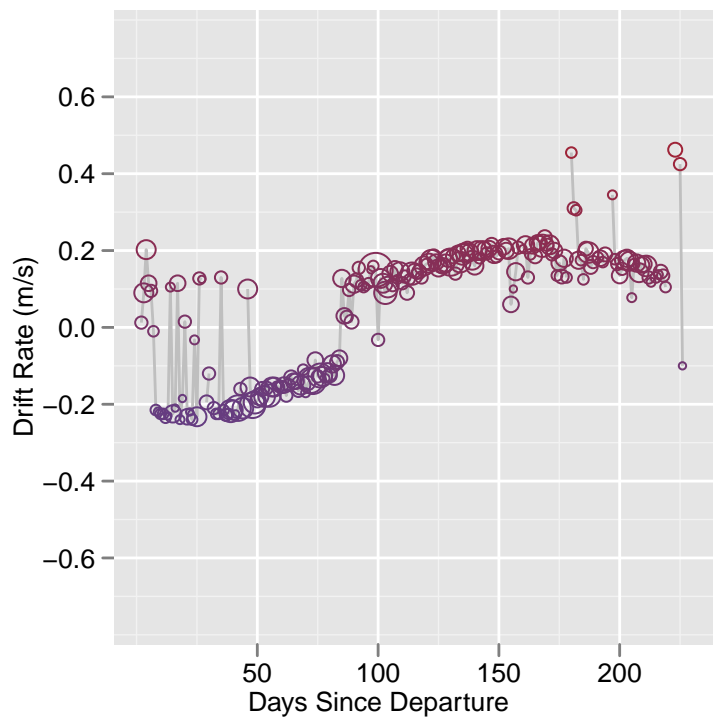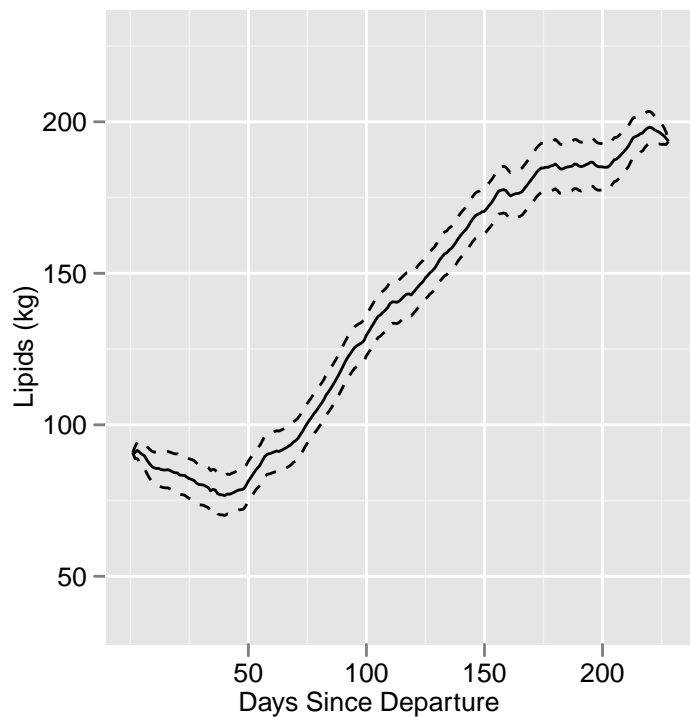

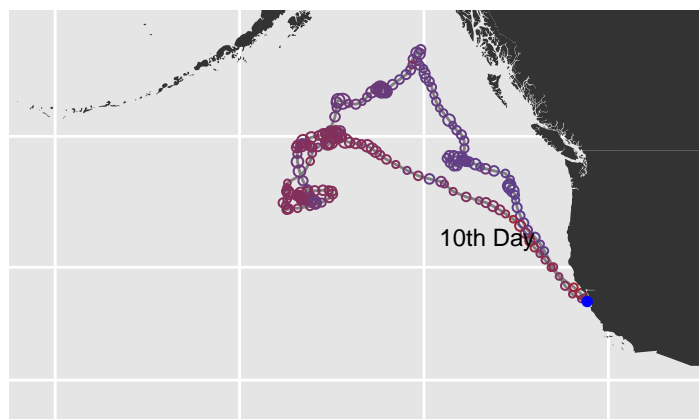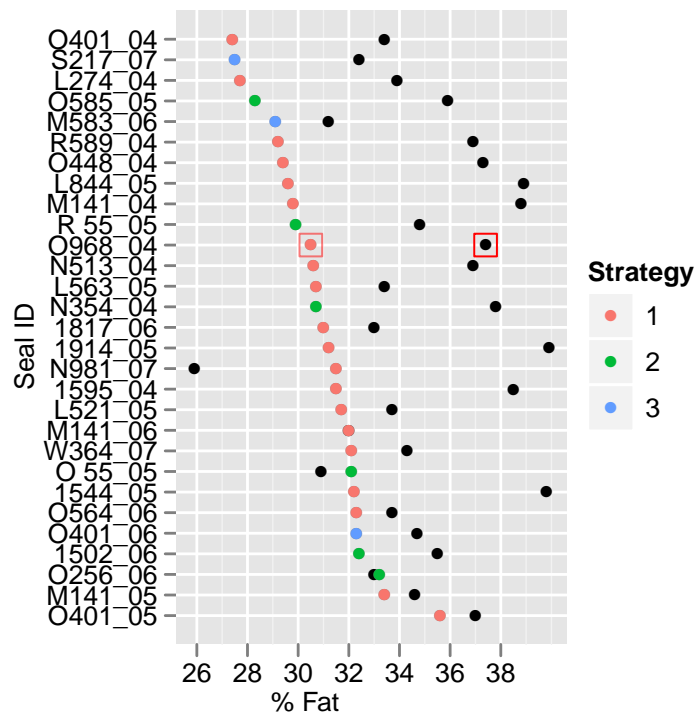

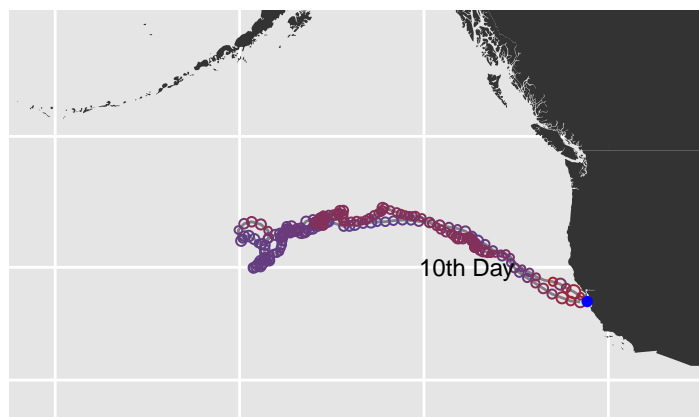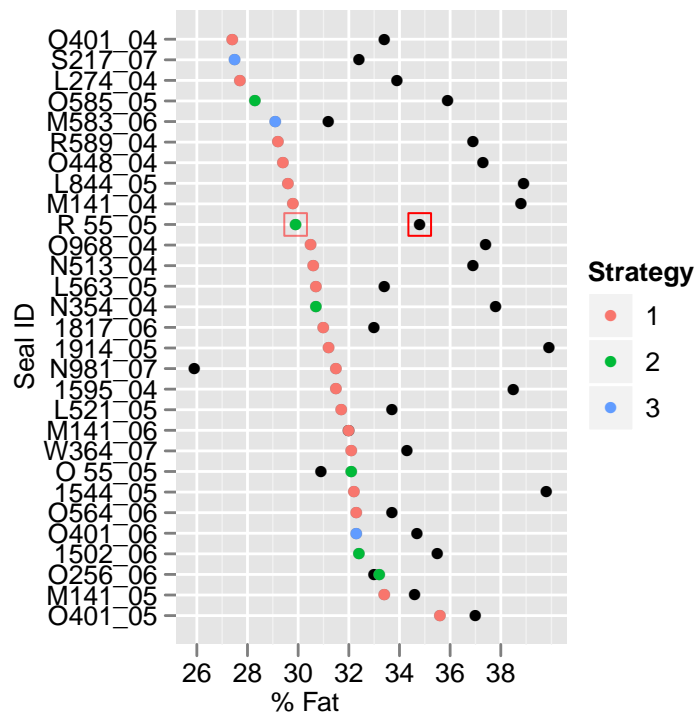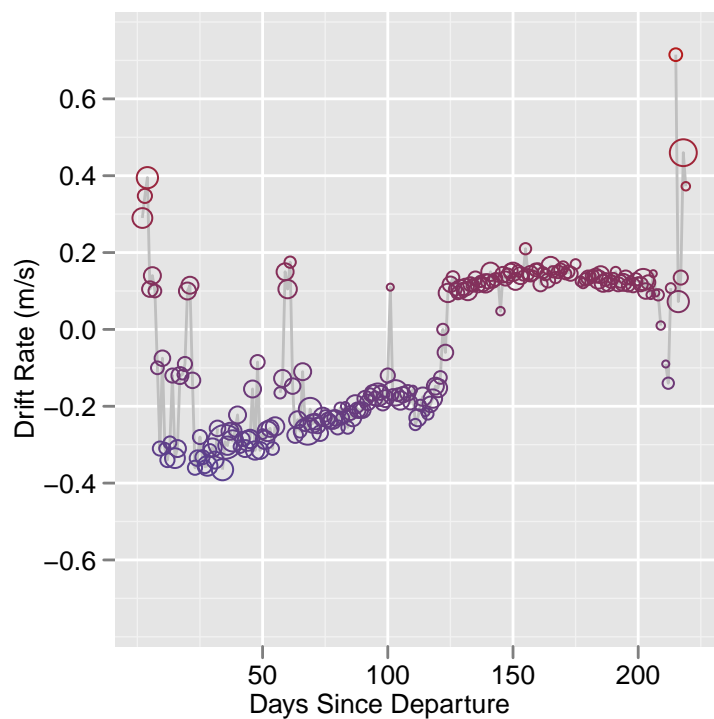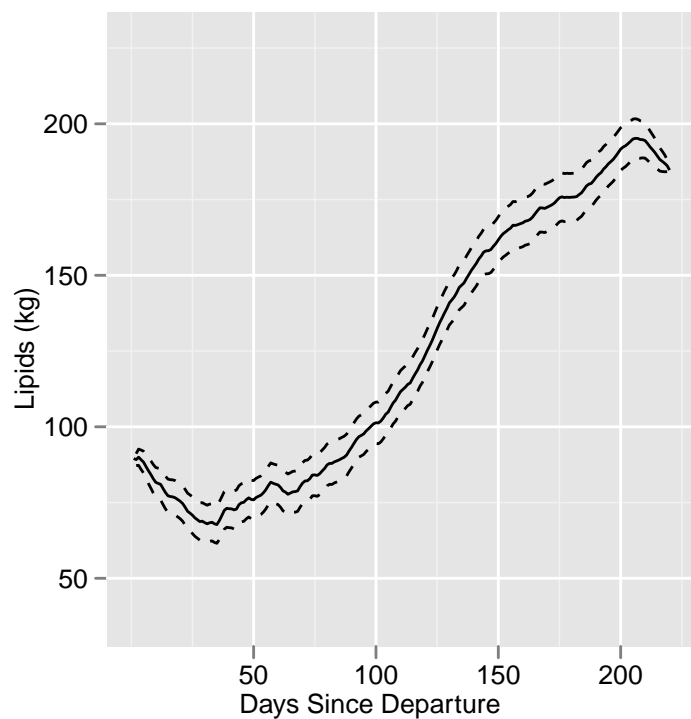

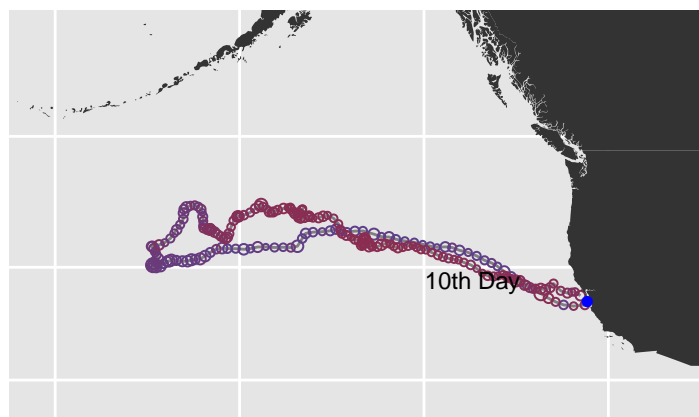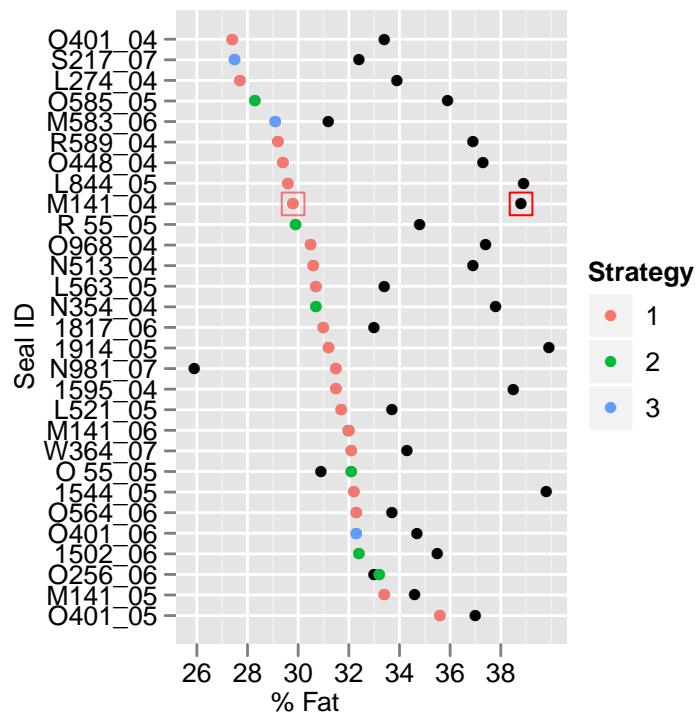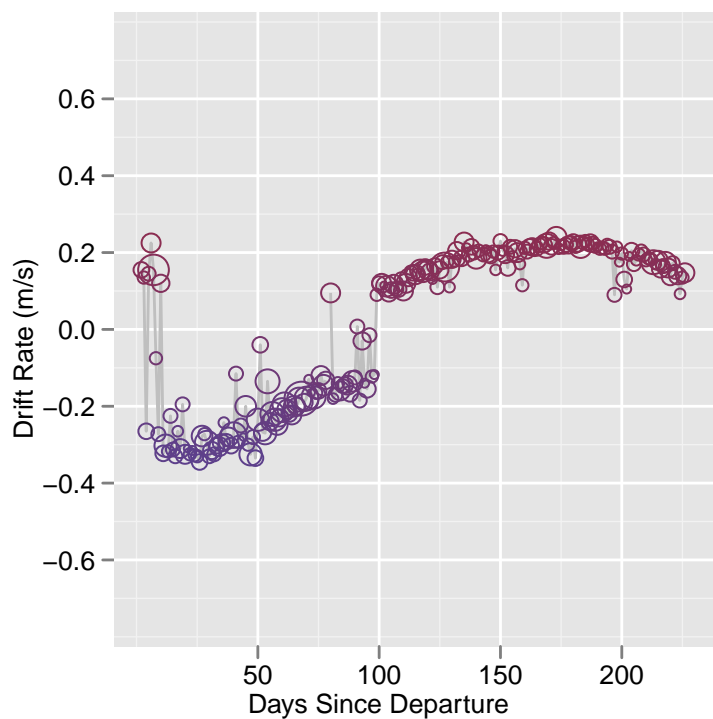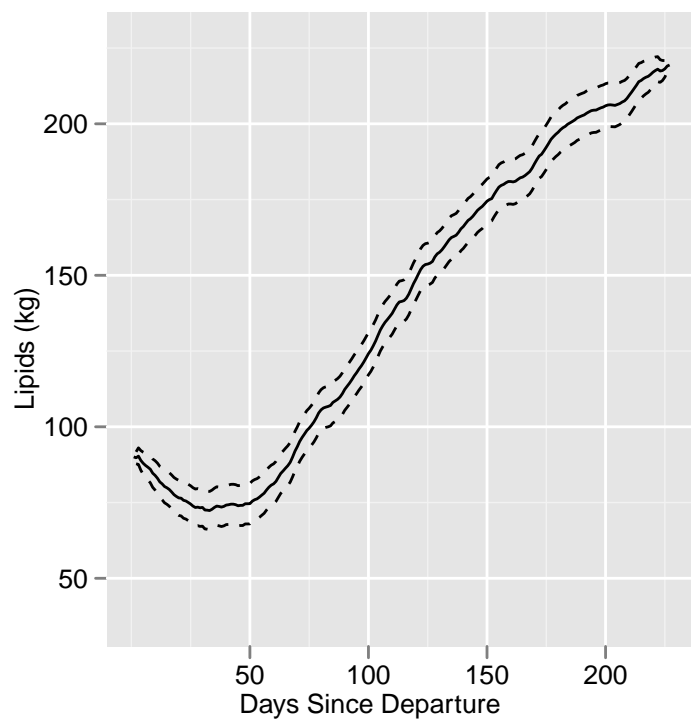

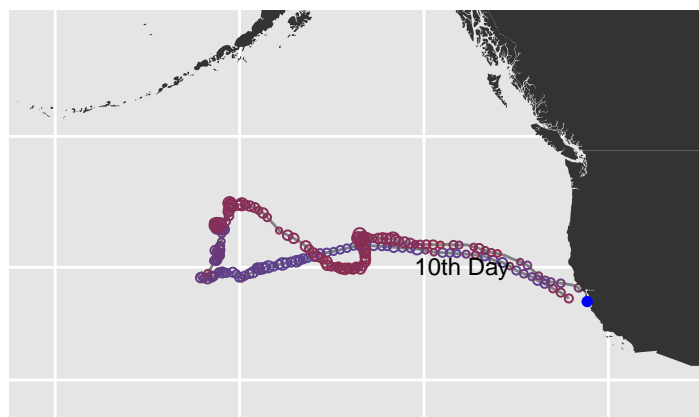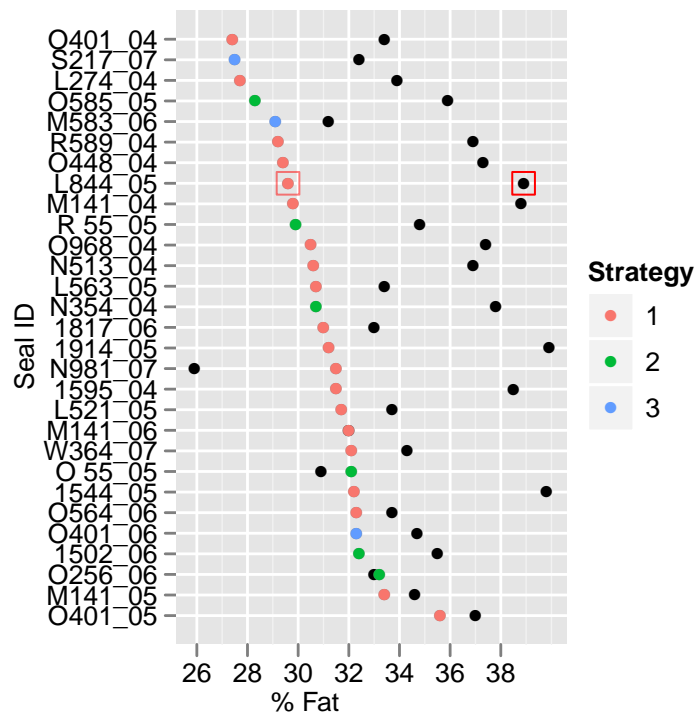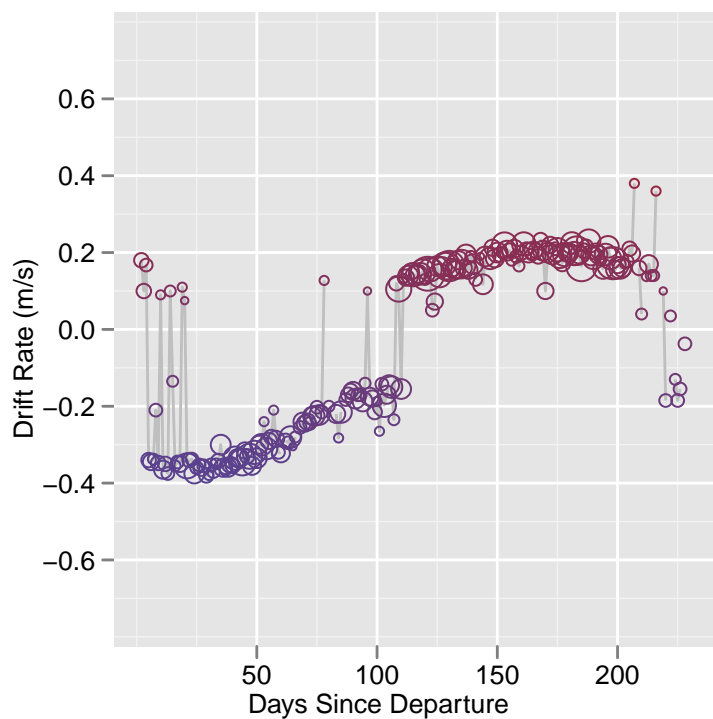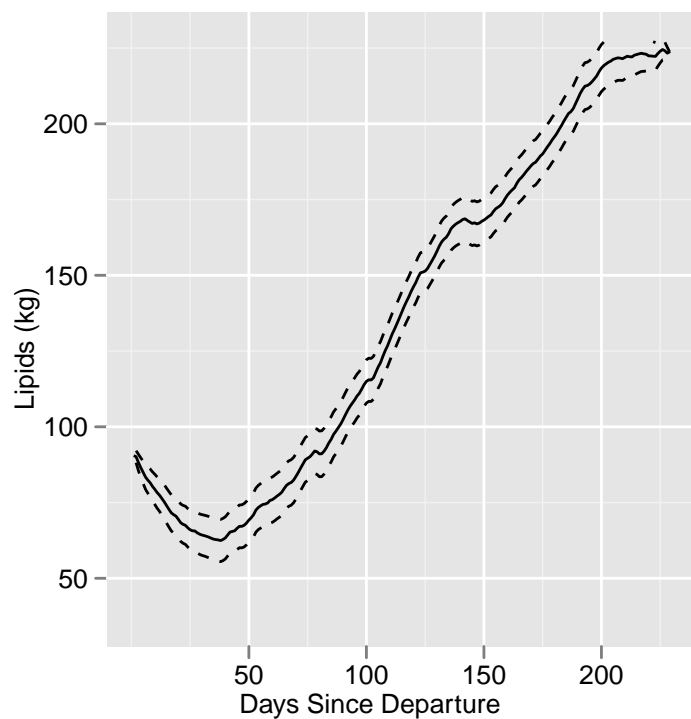

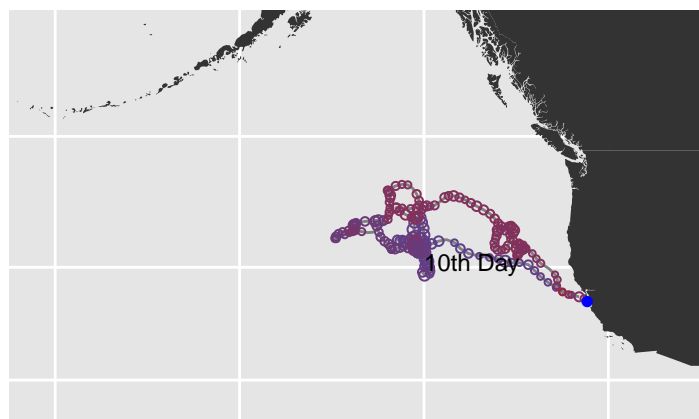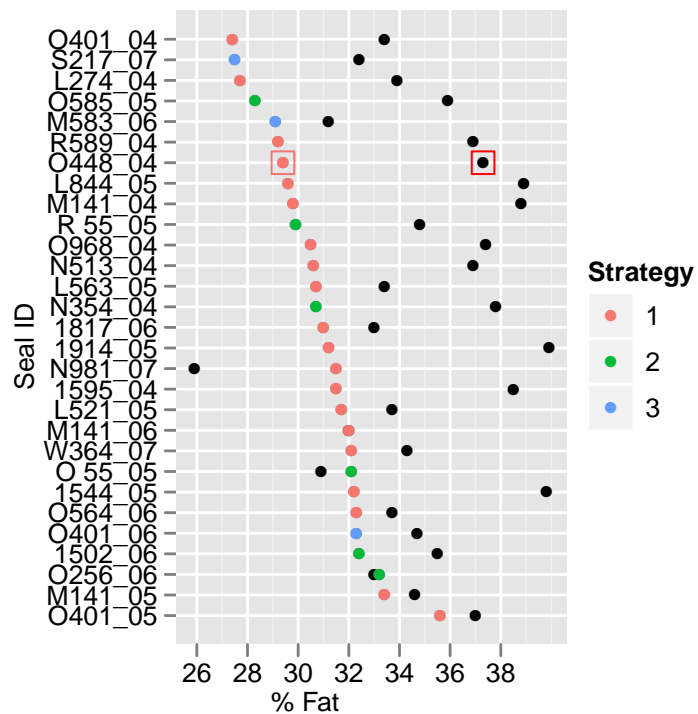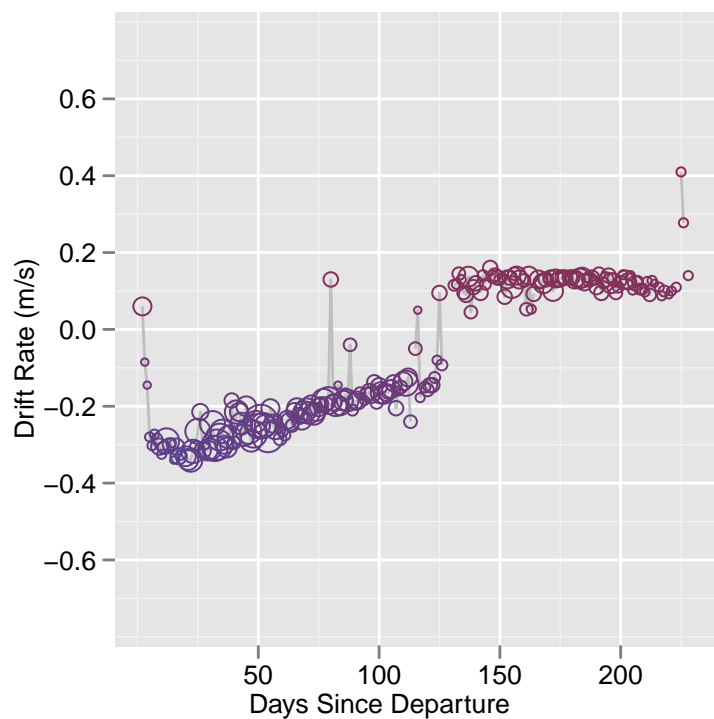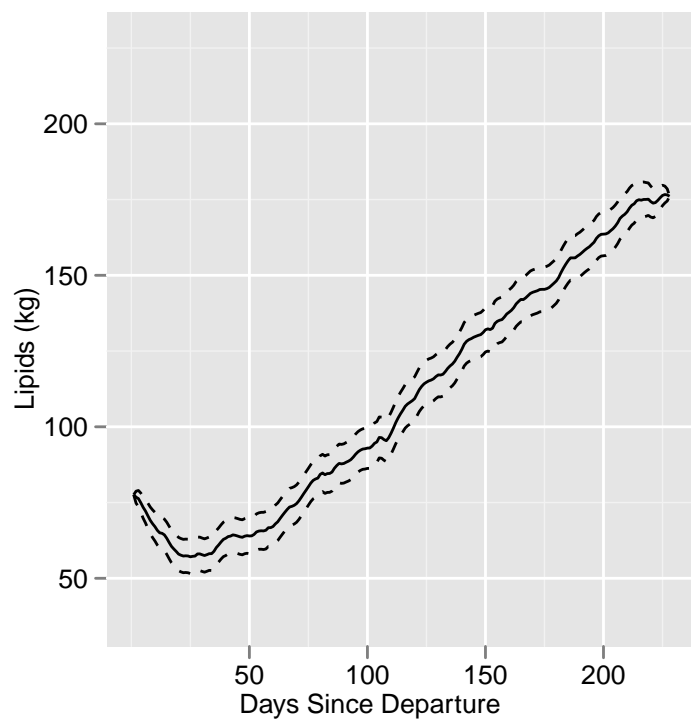

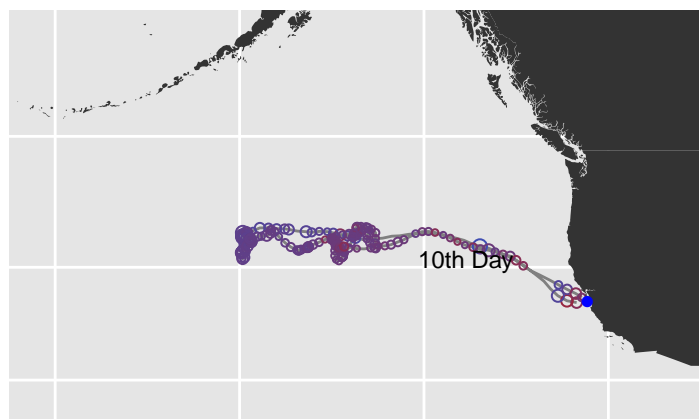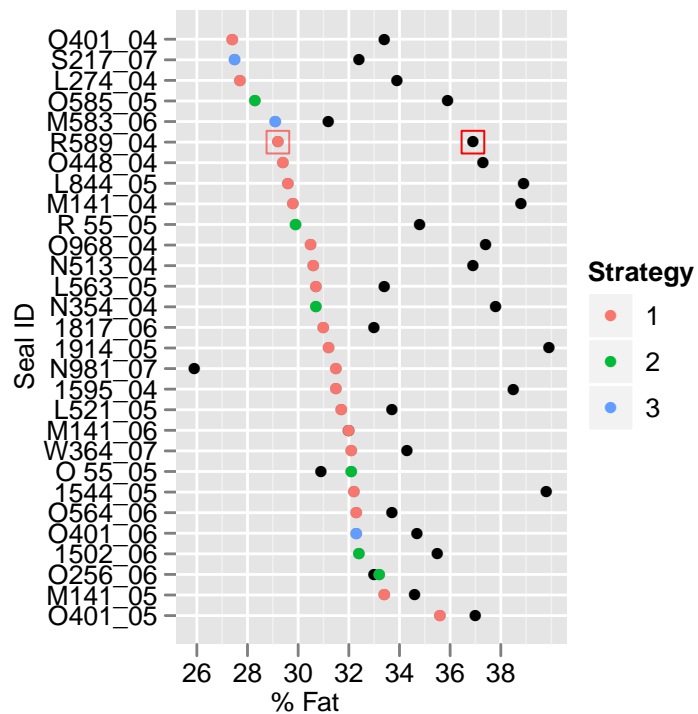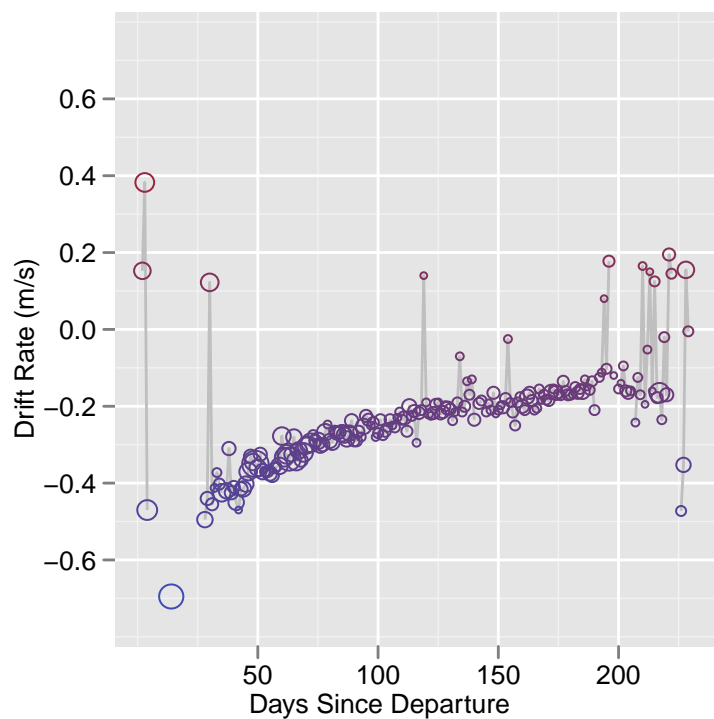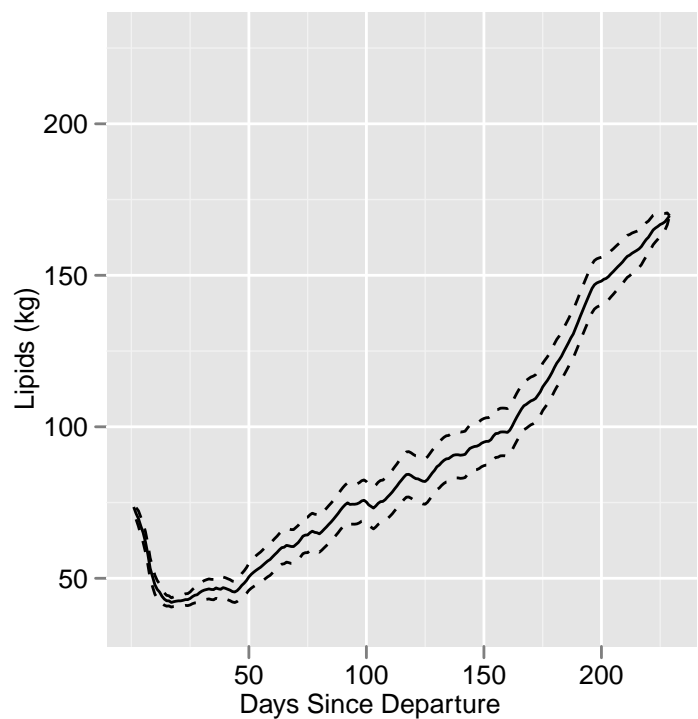

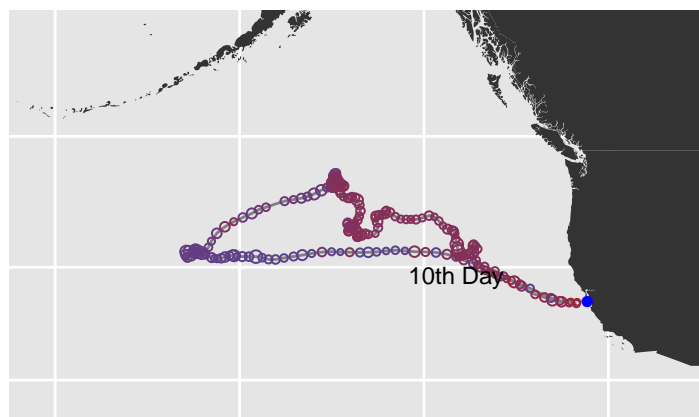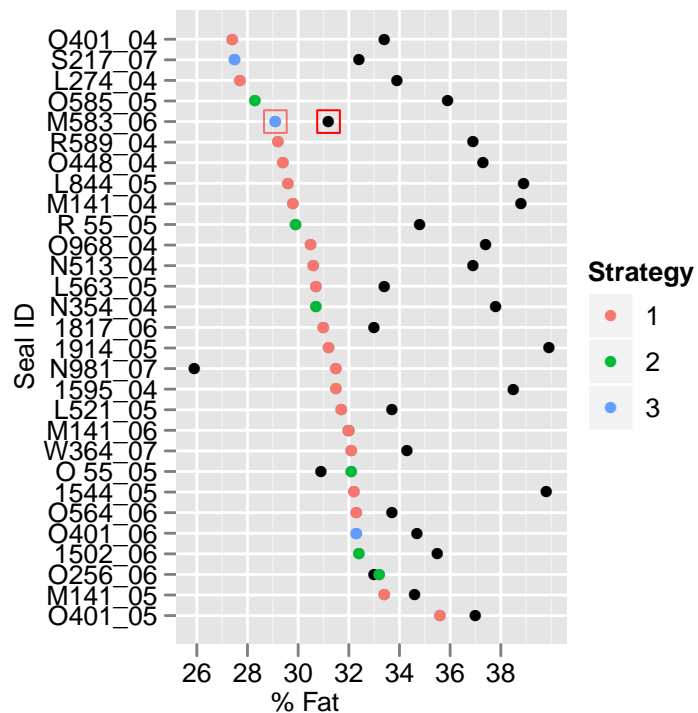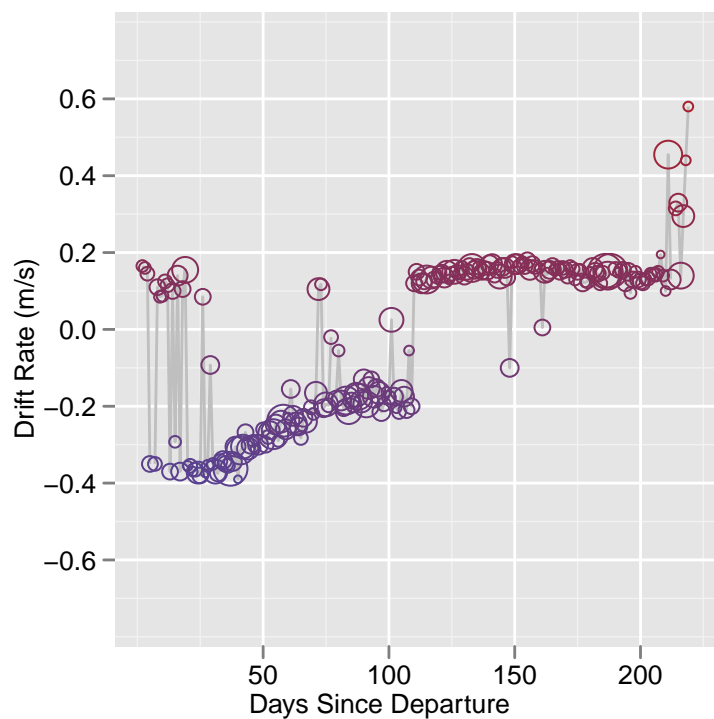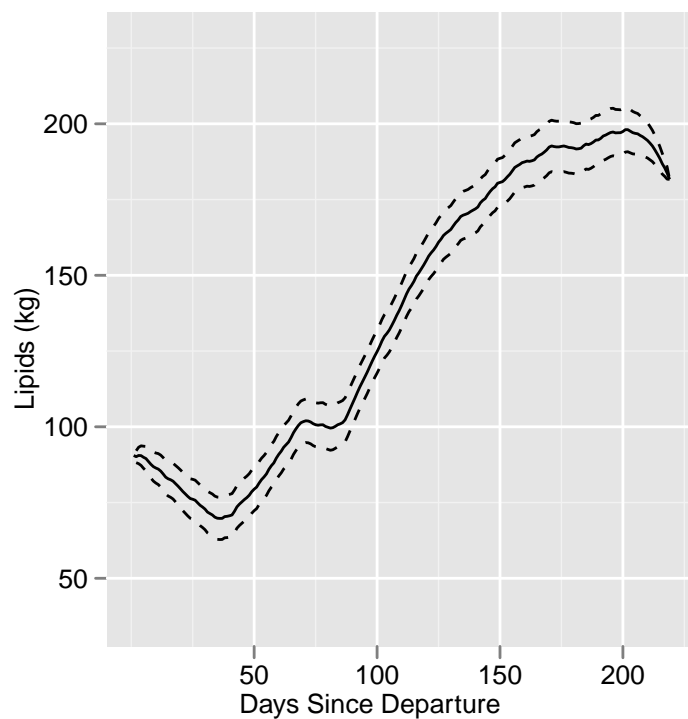

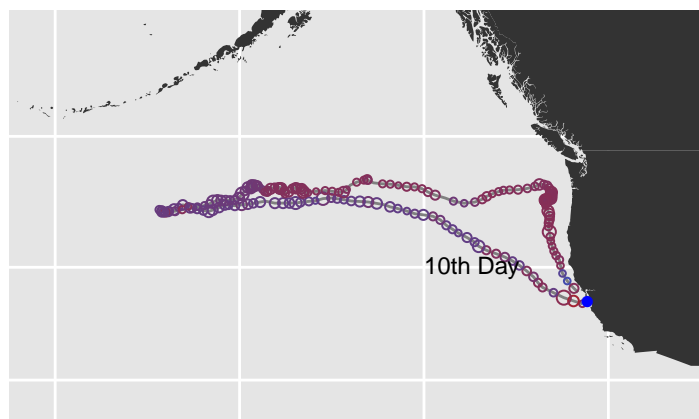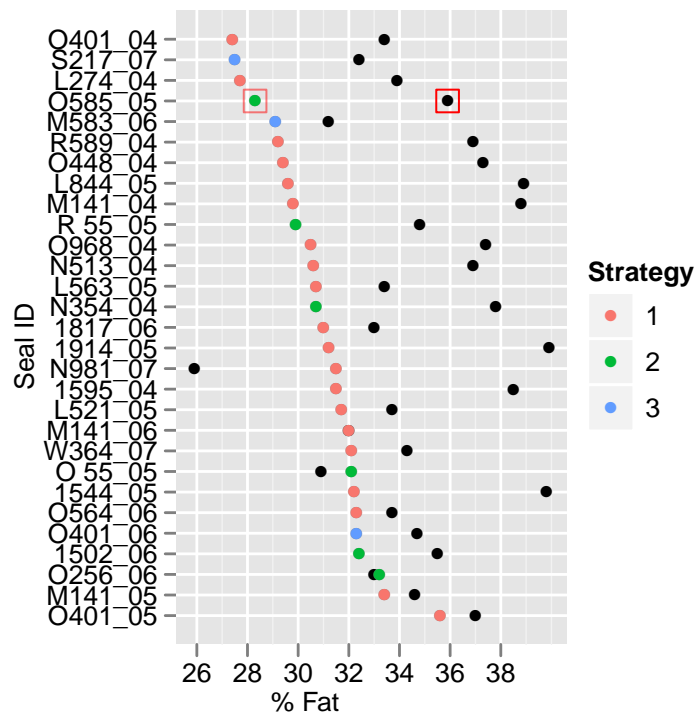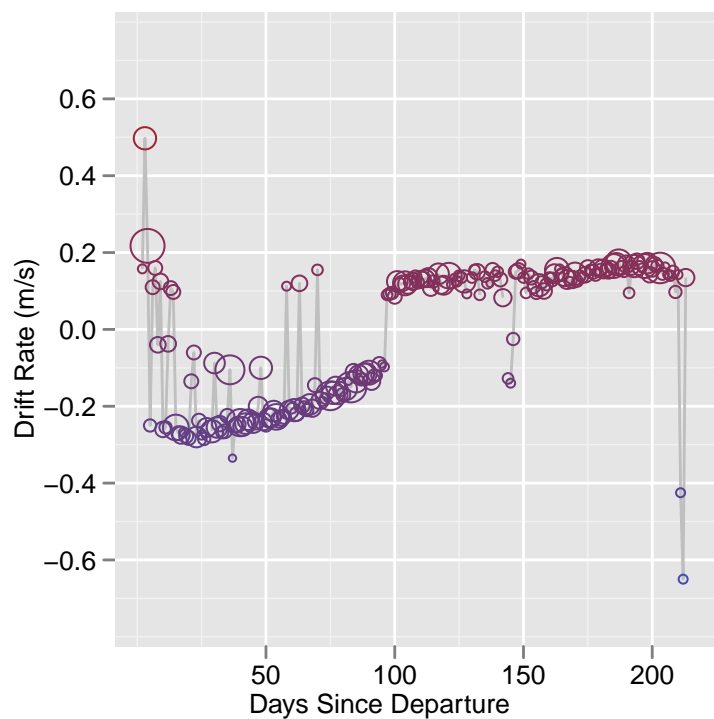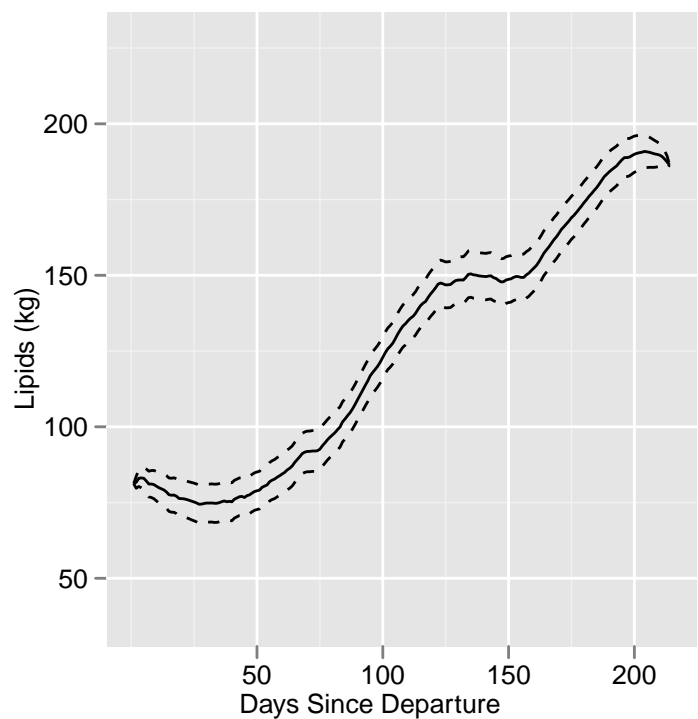

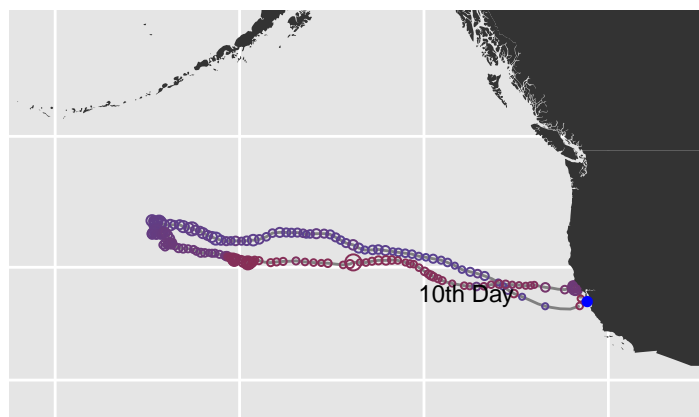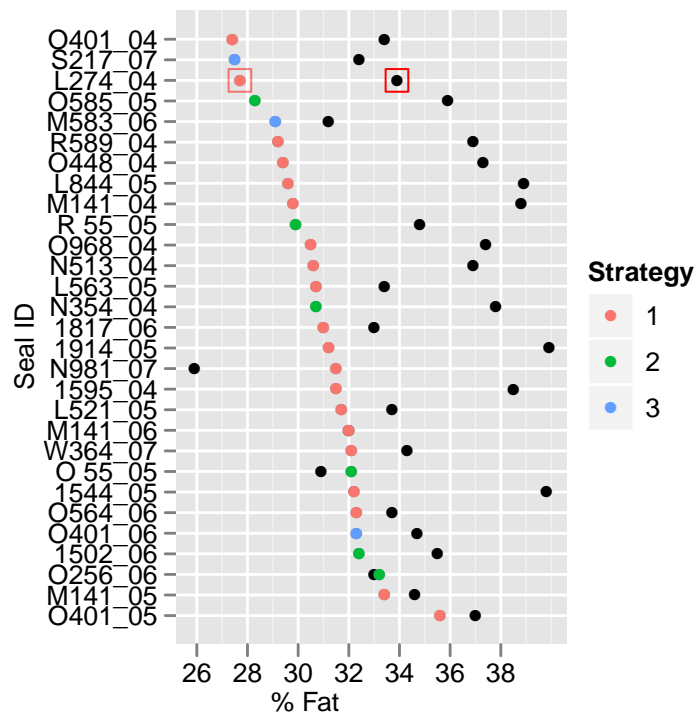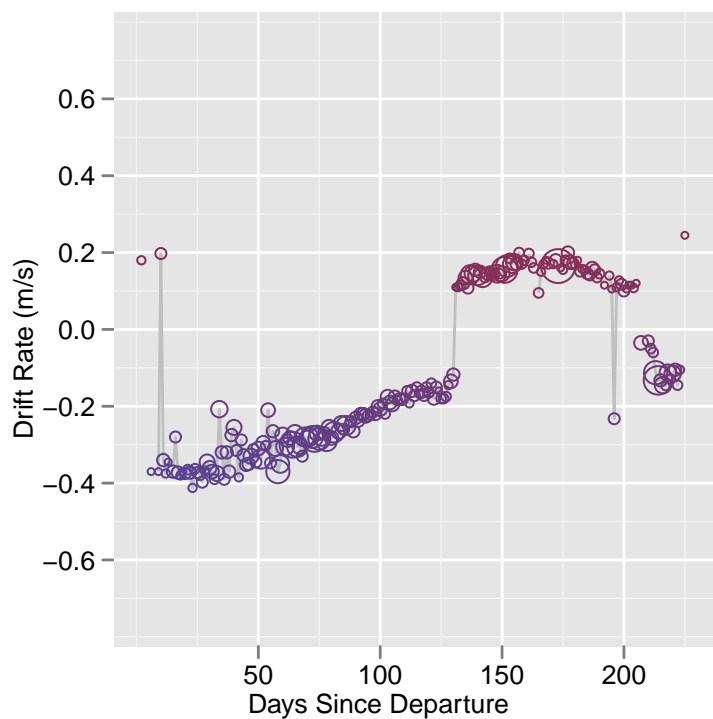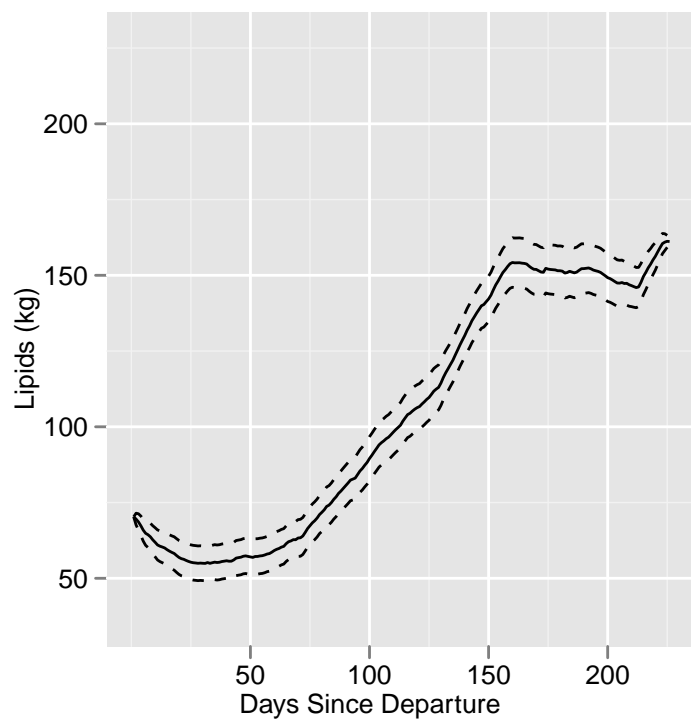

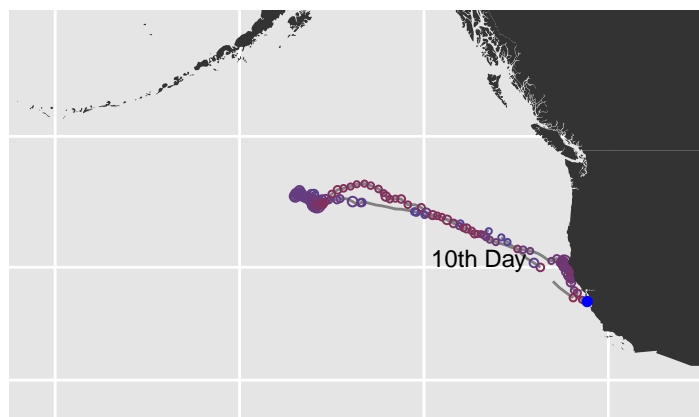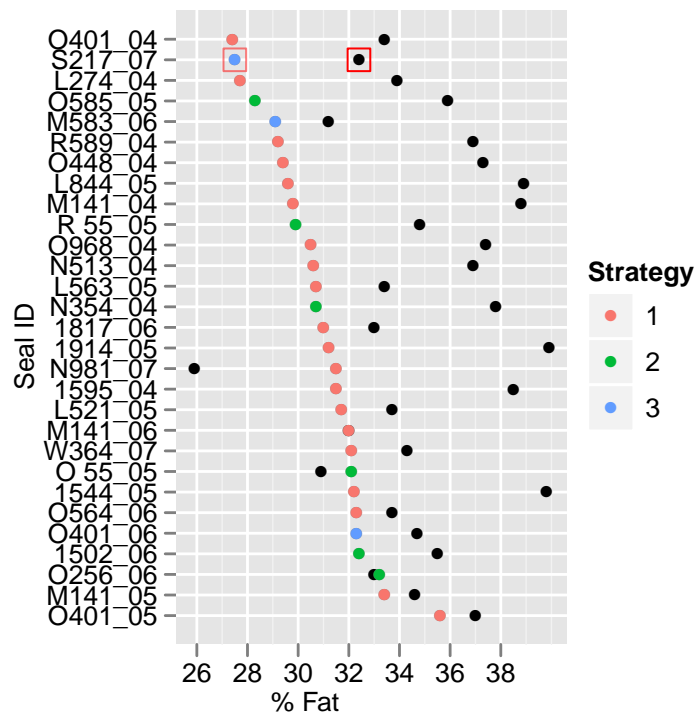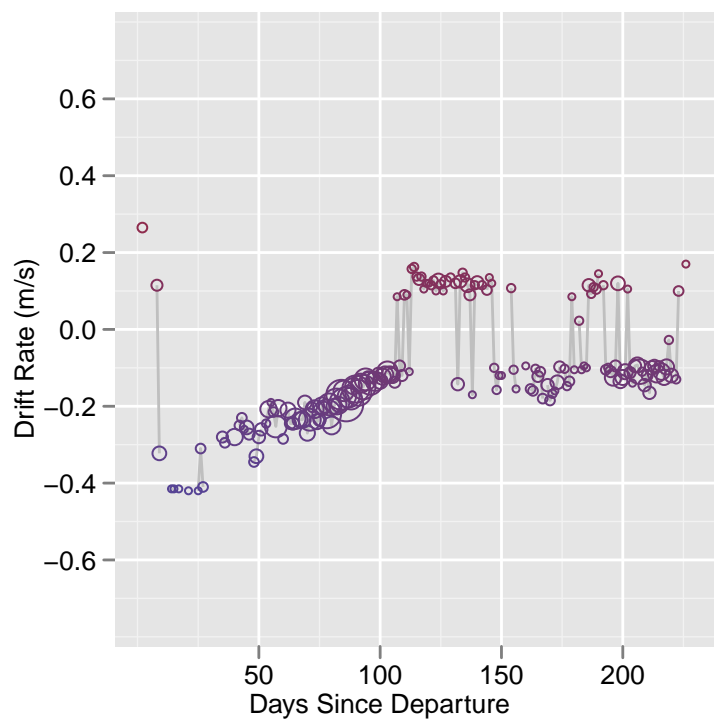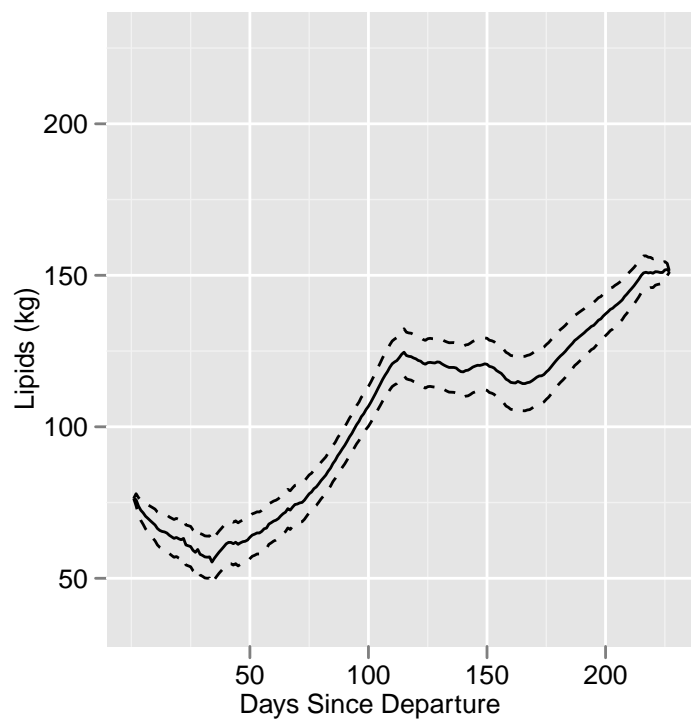

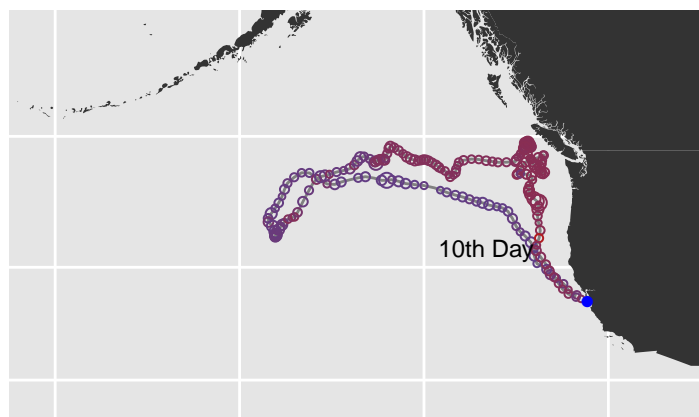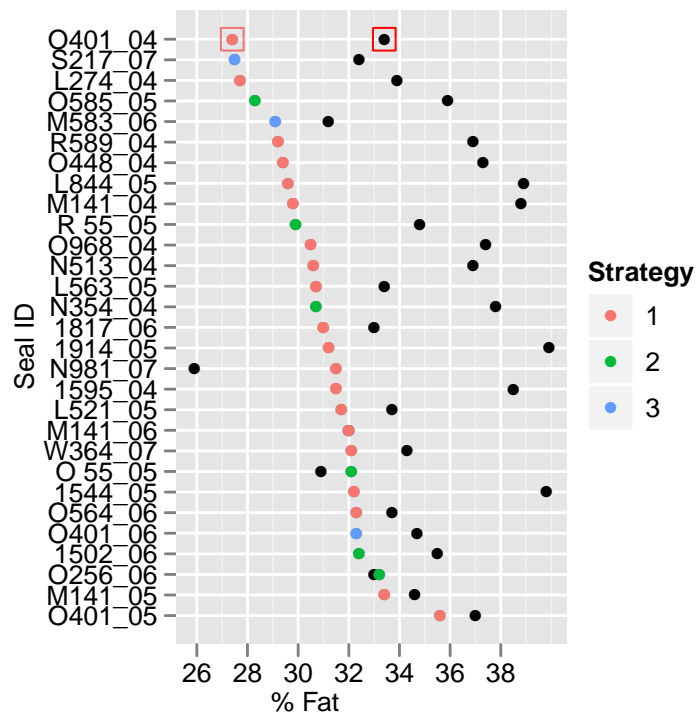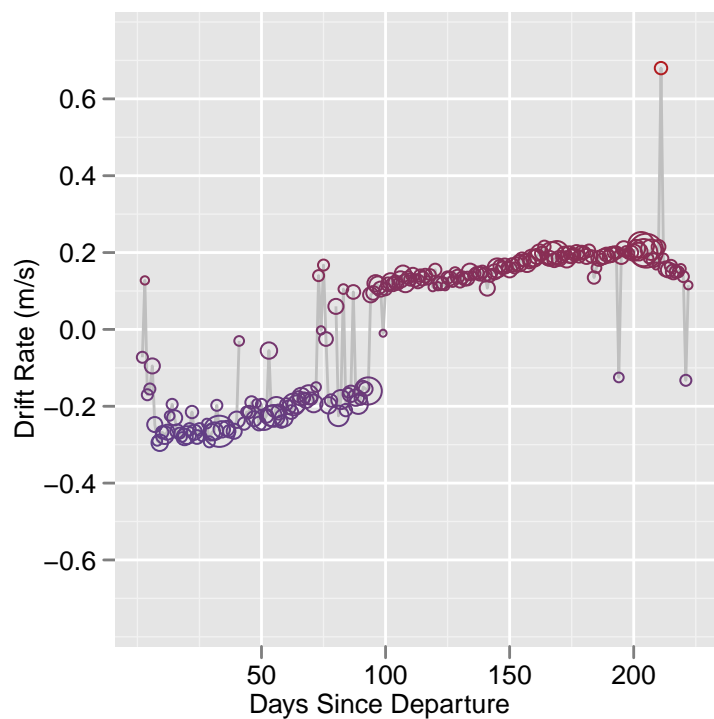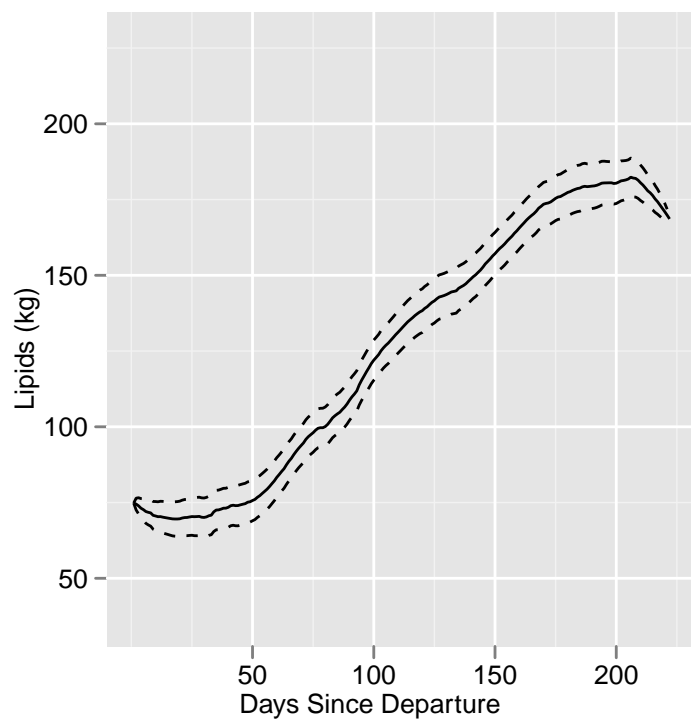

Supplement: Supplementary file 4 [file jane0082-1300-sd4.pdf]

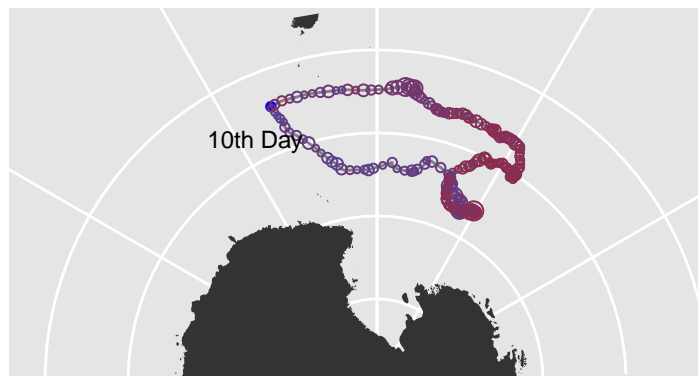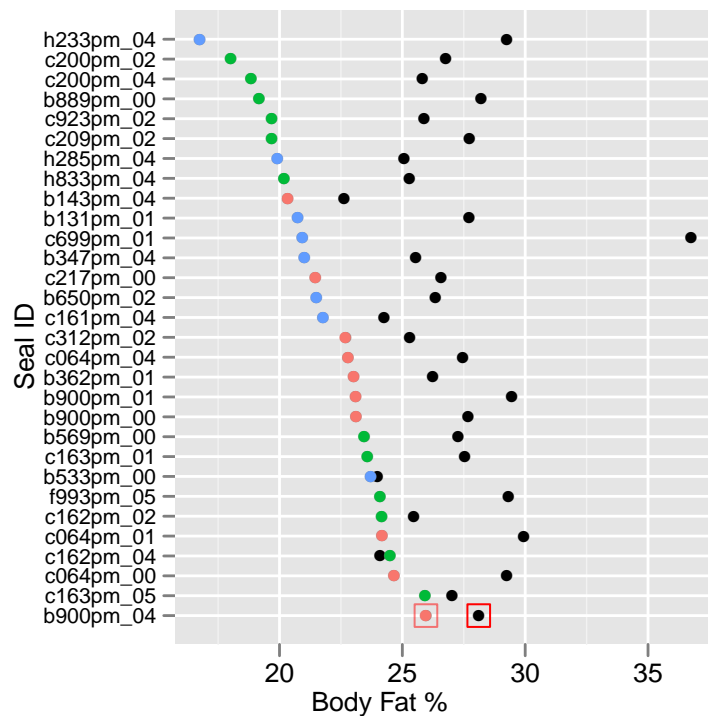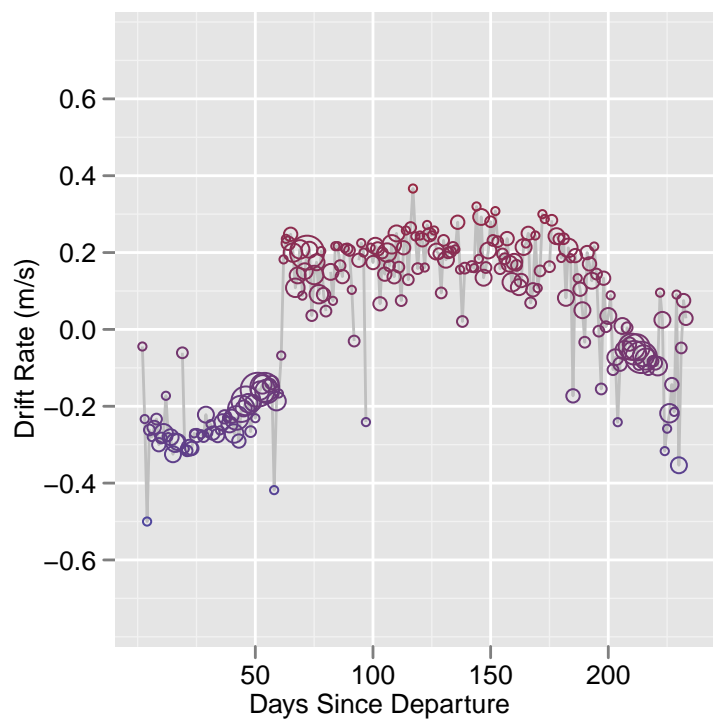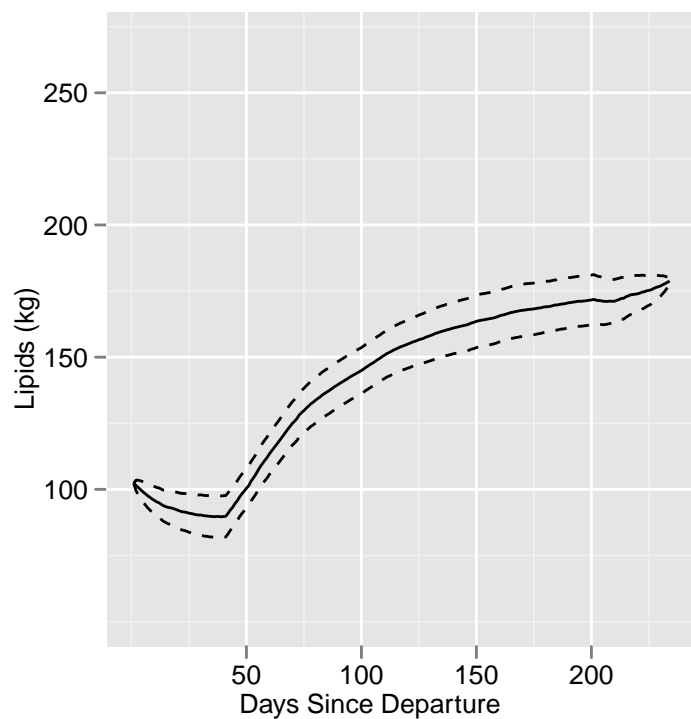

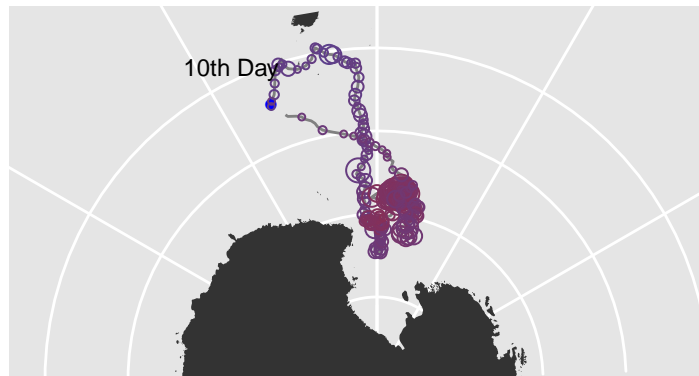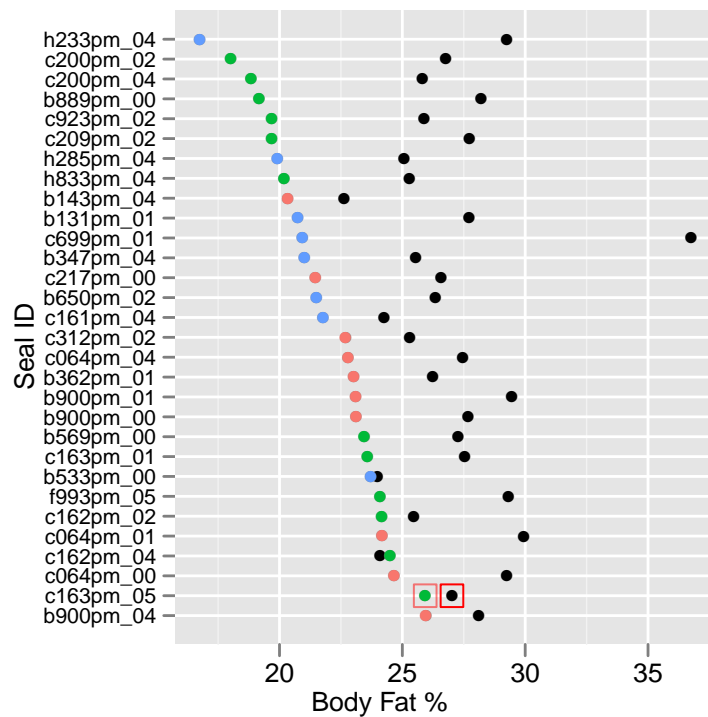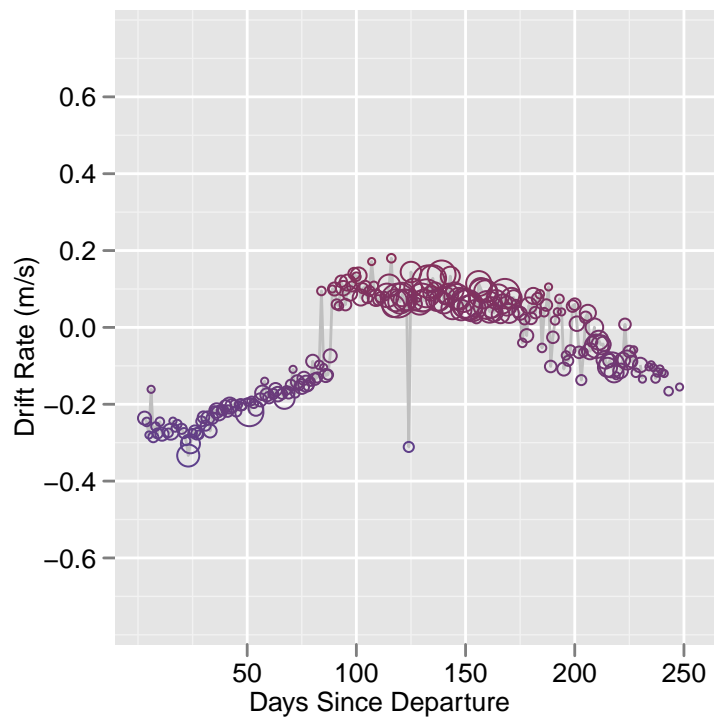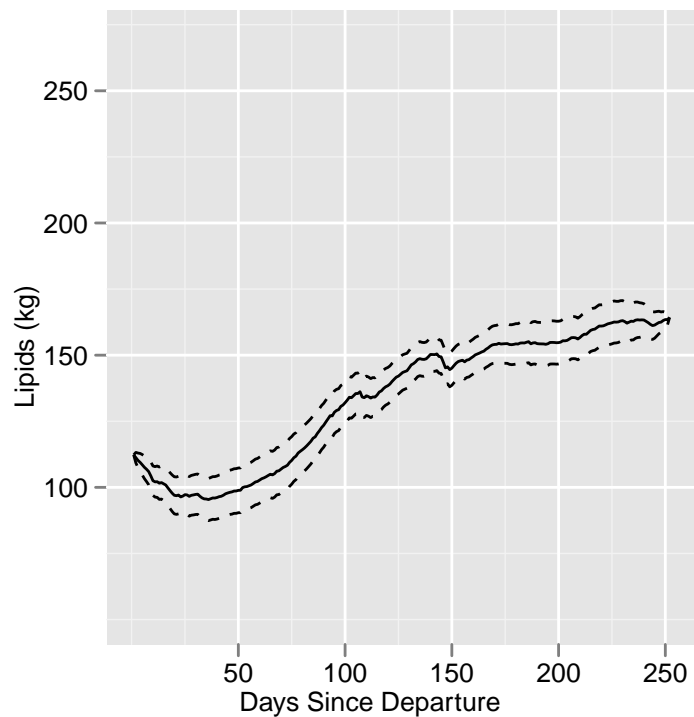

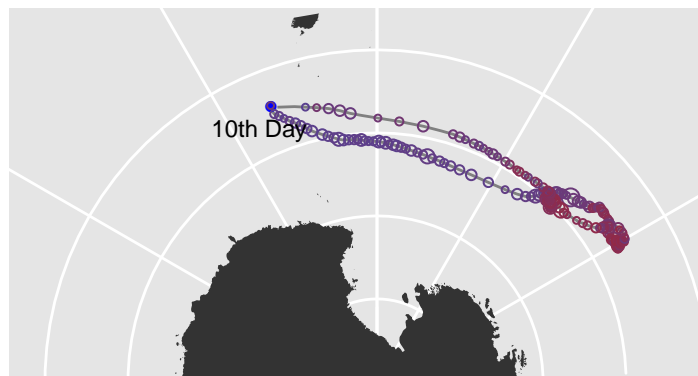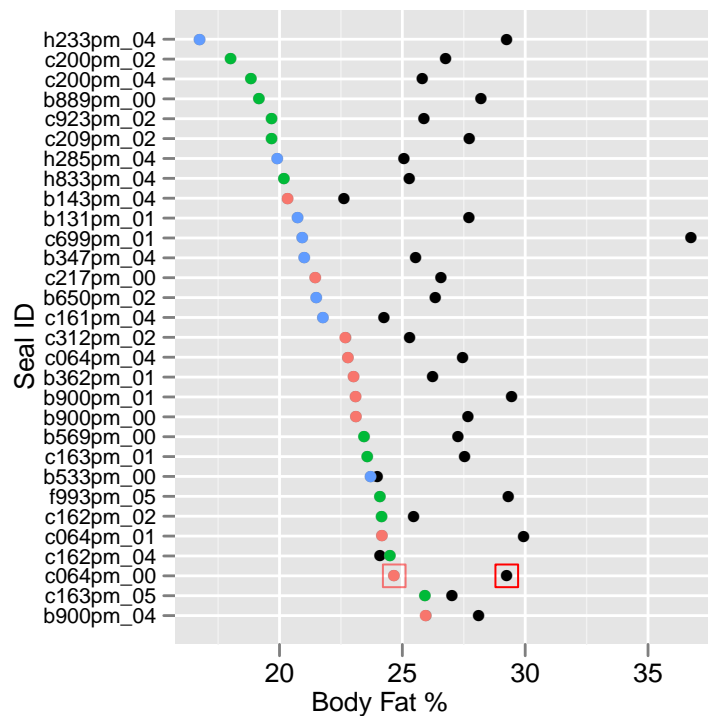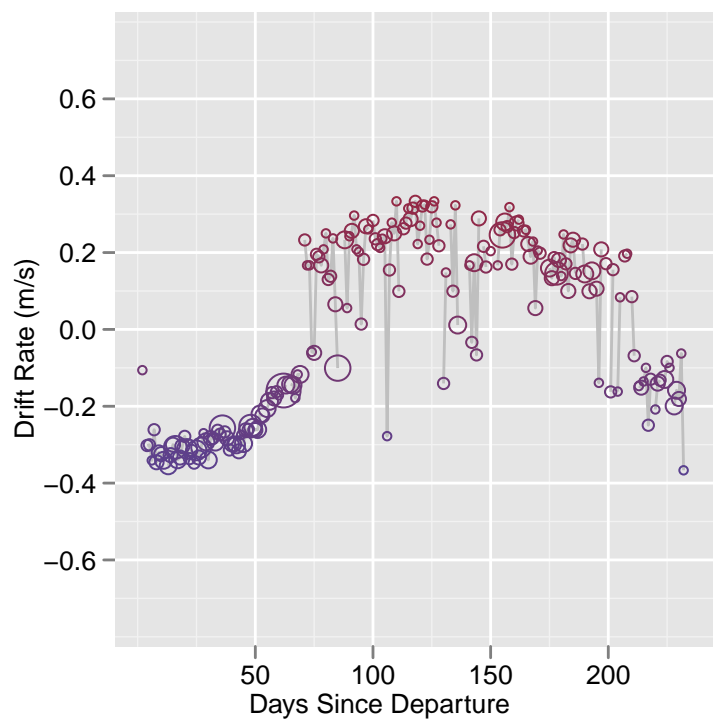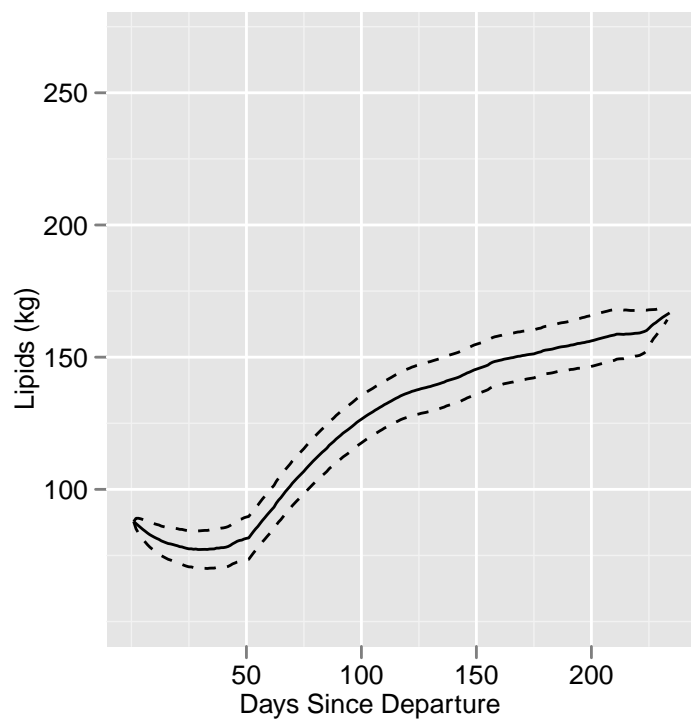

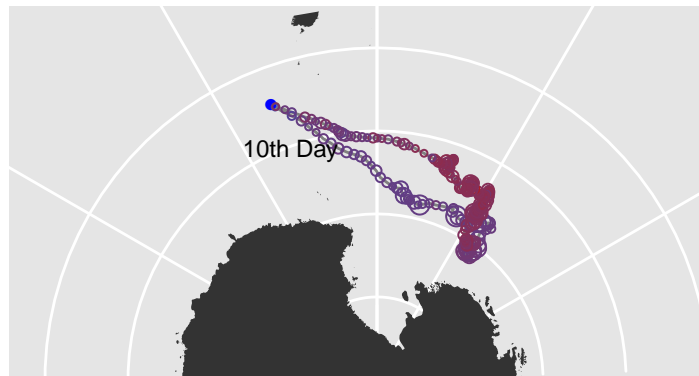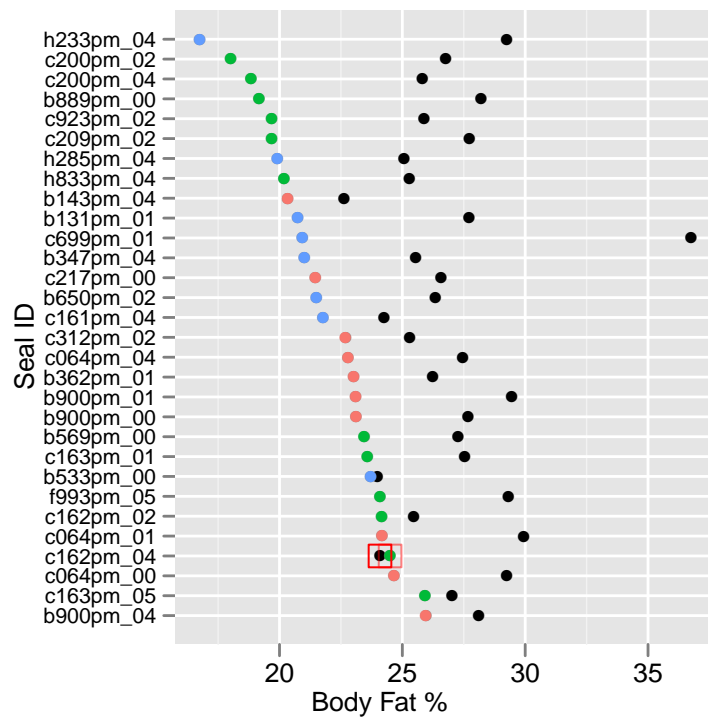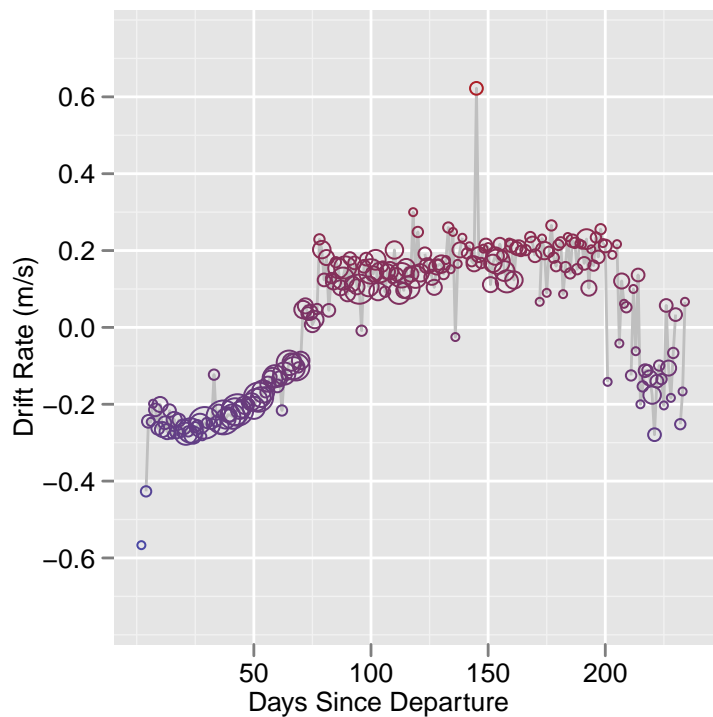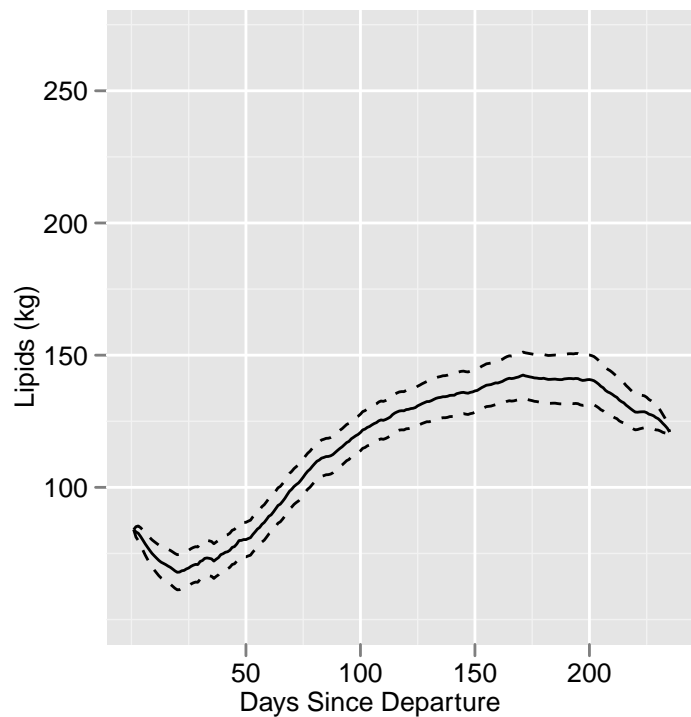

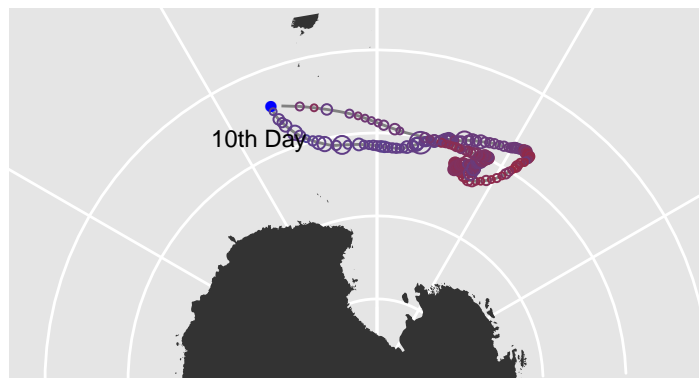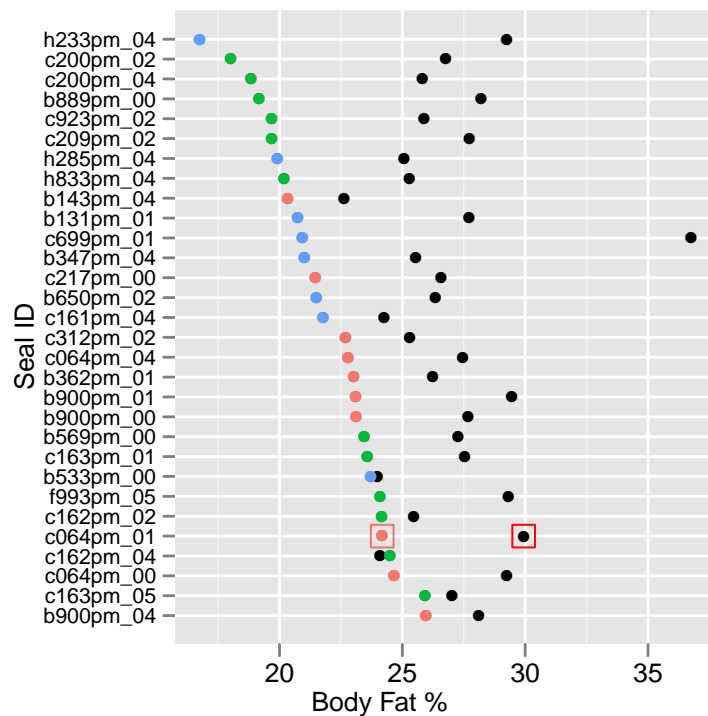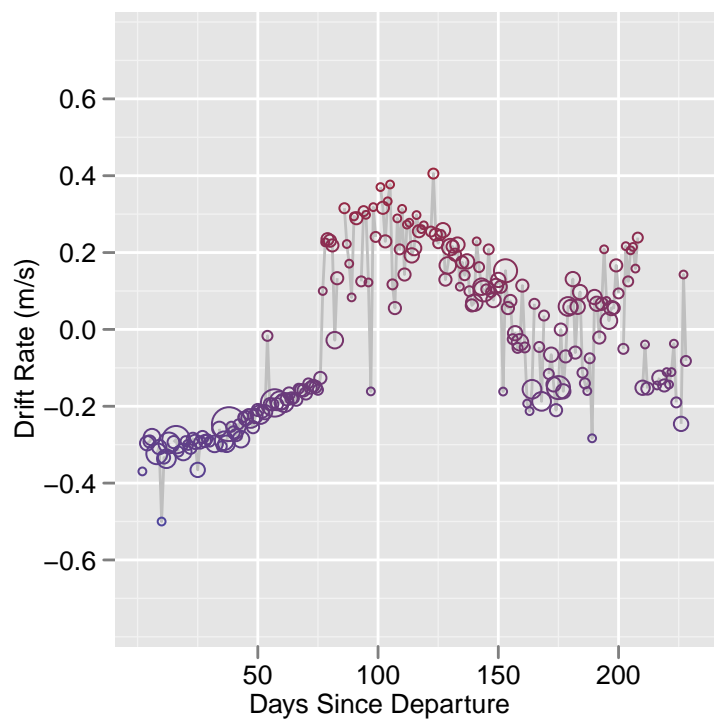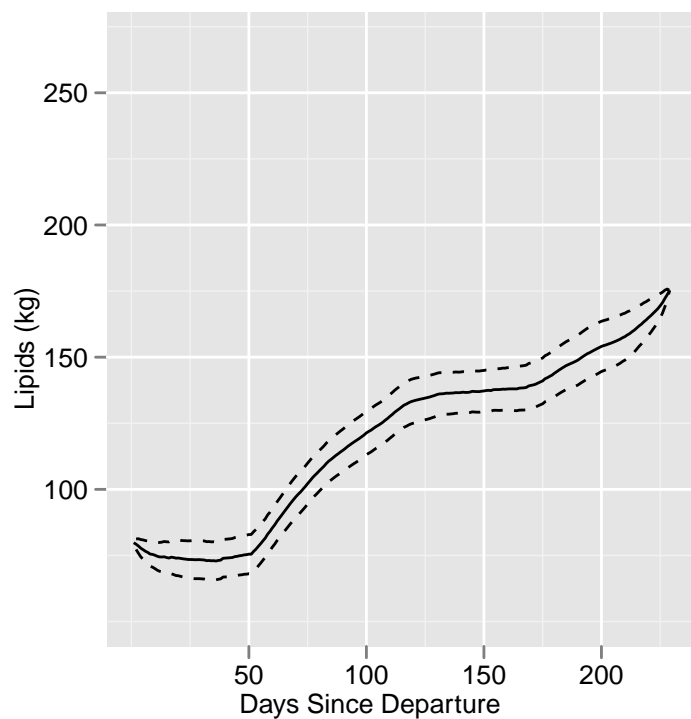

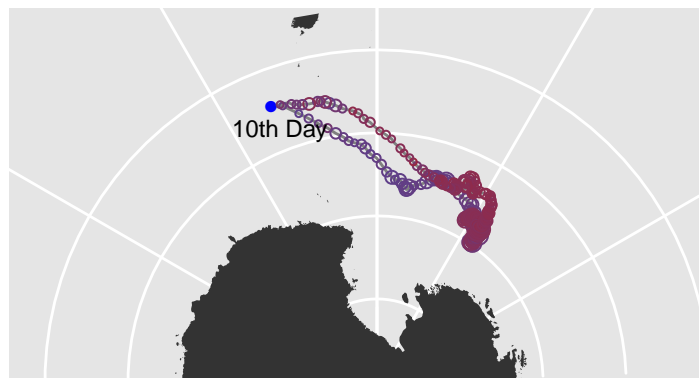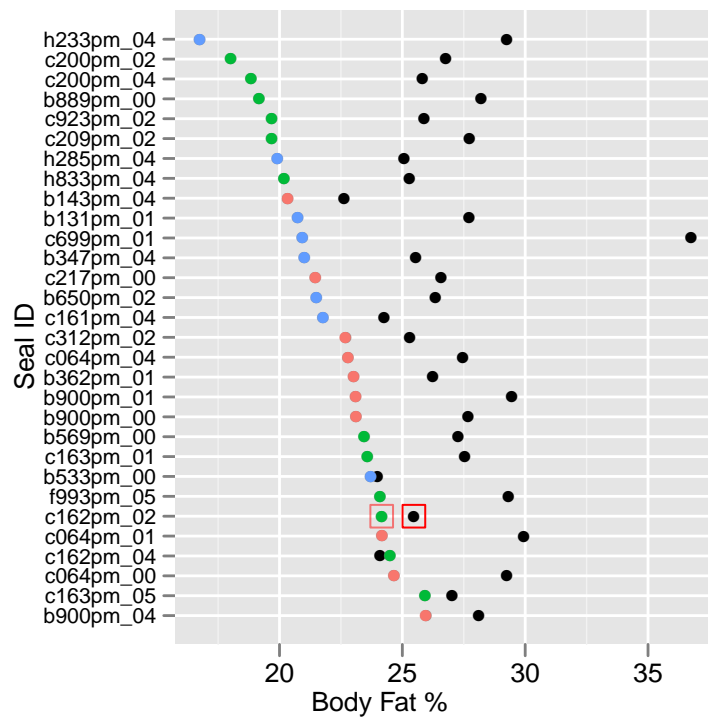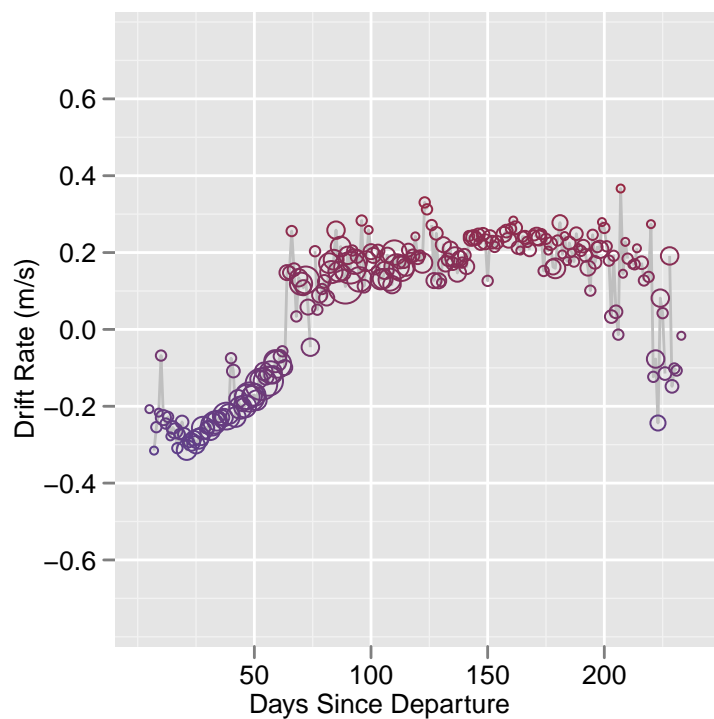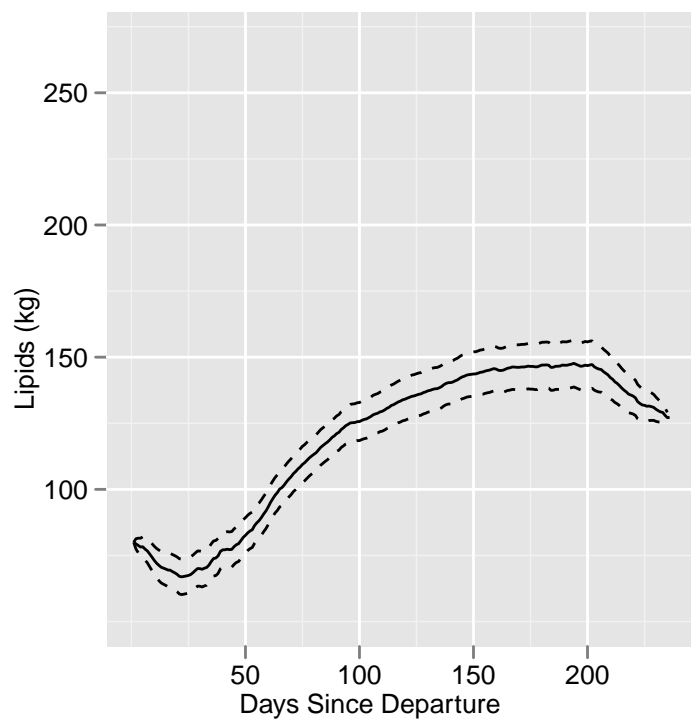

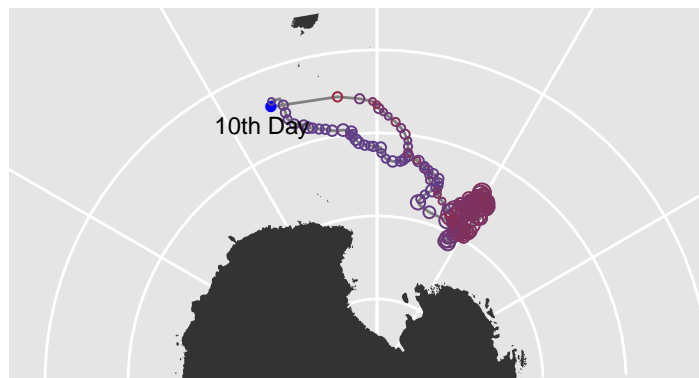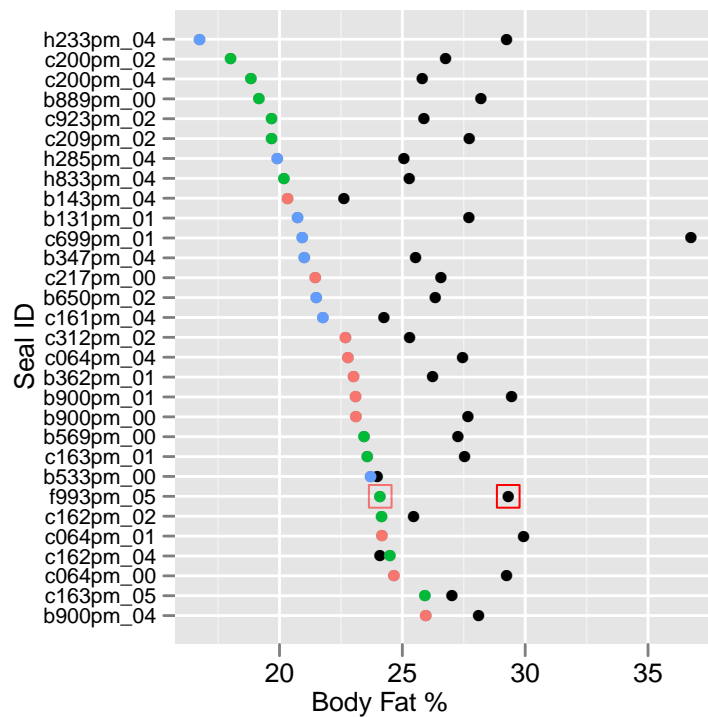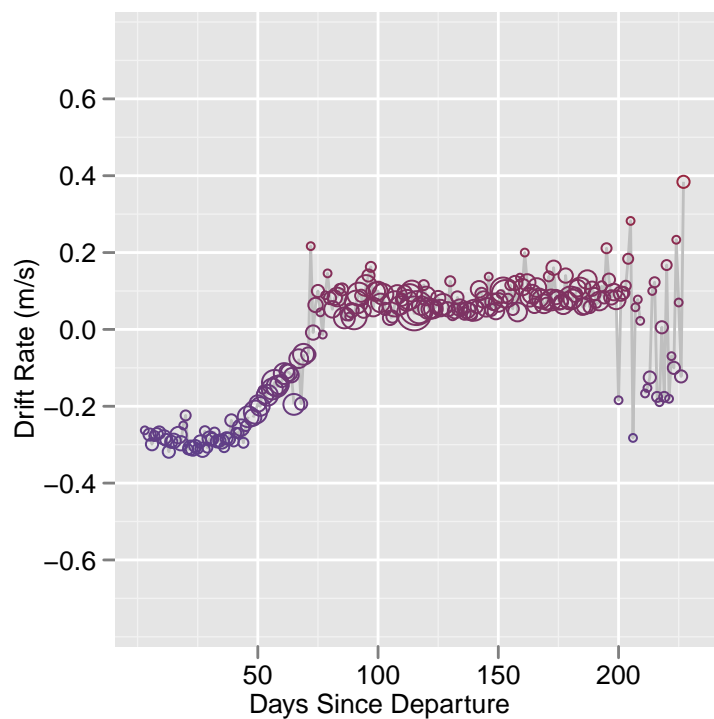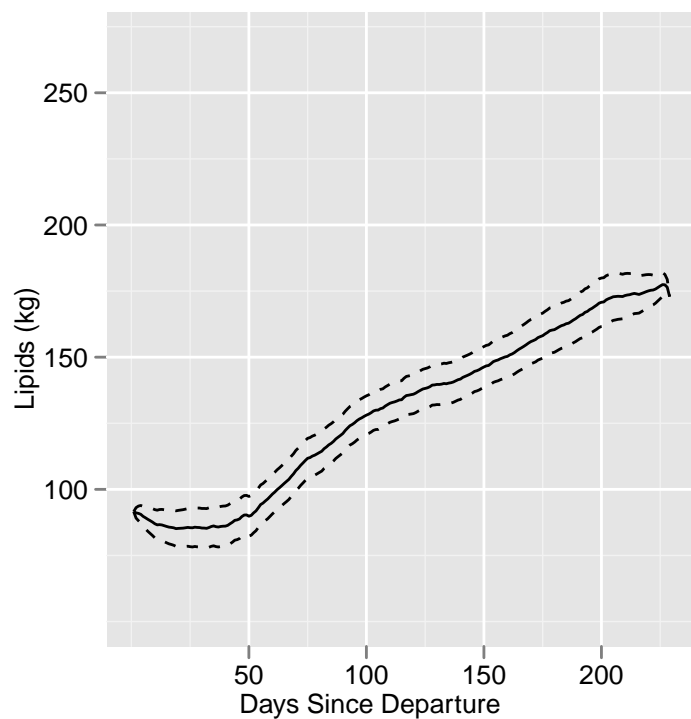

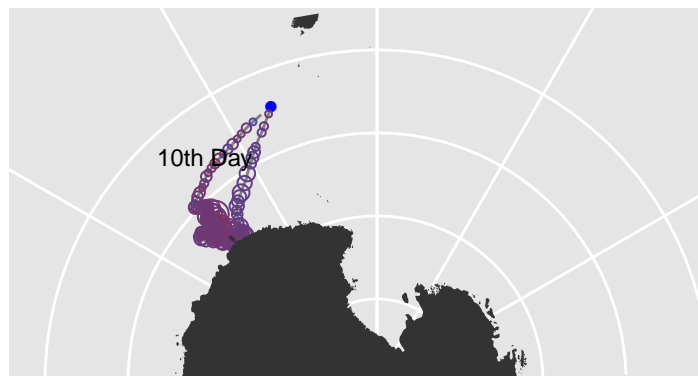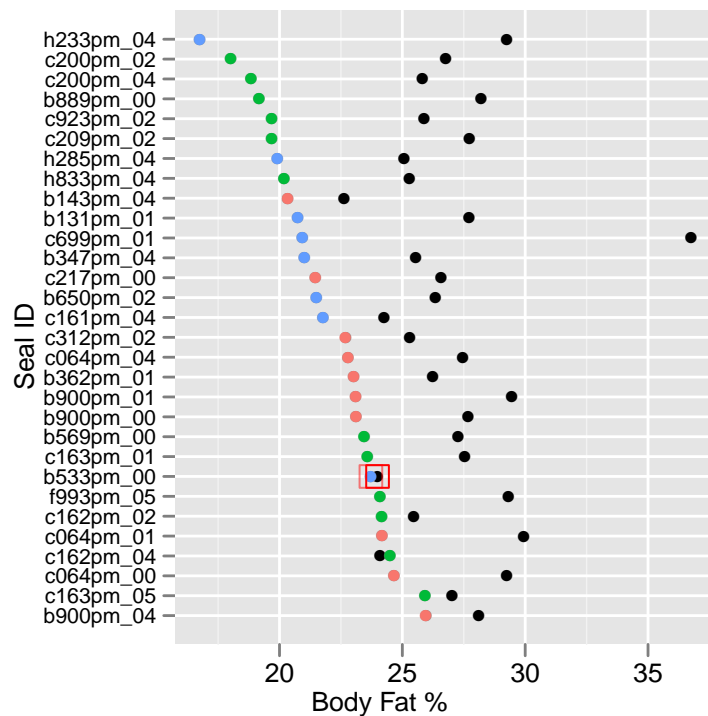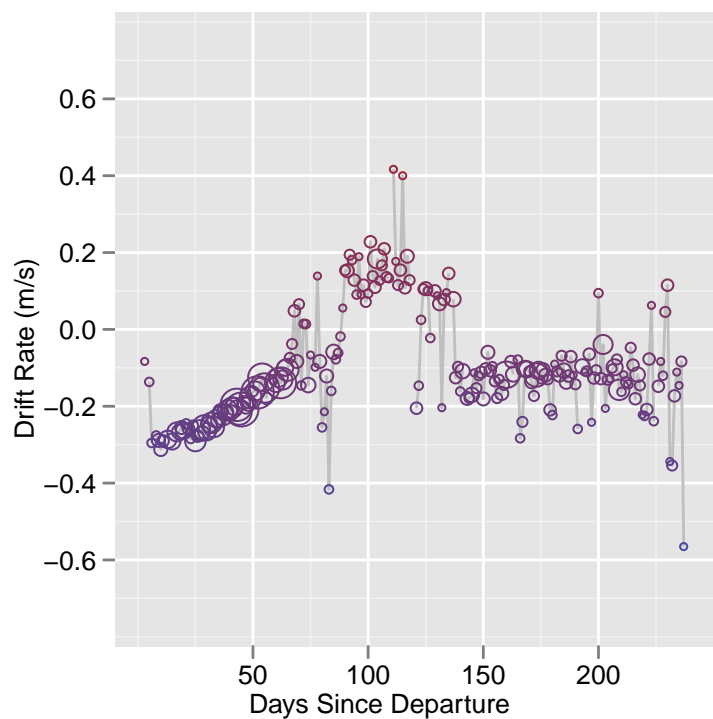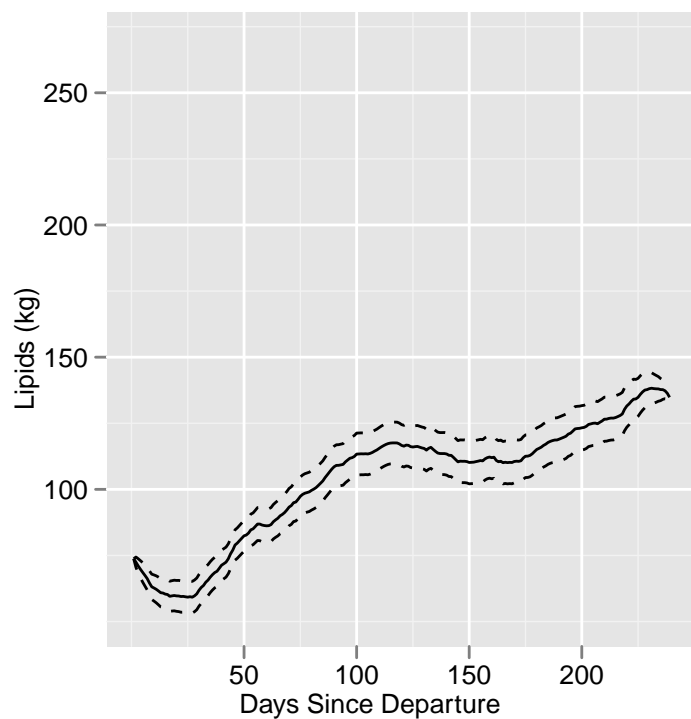

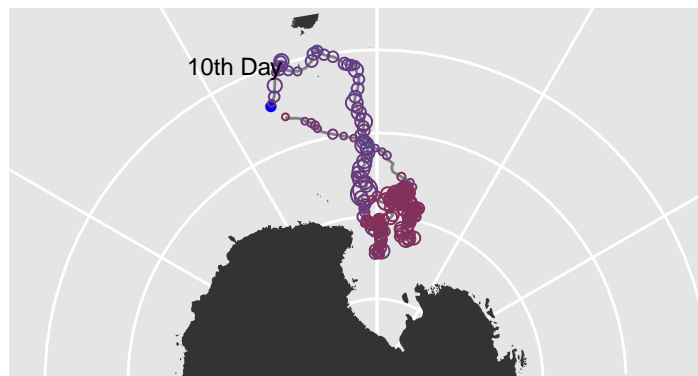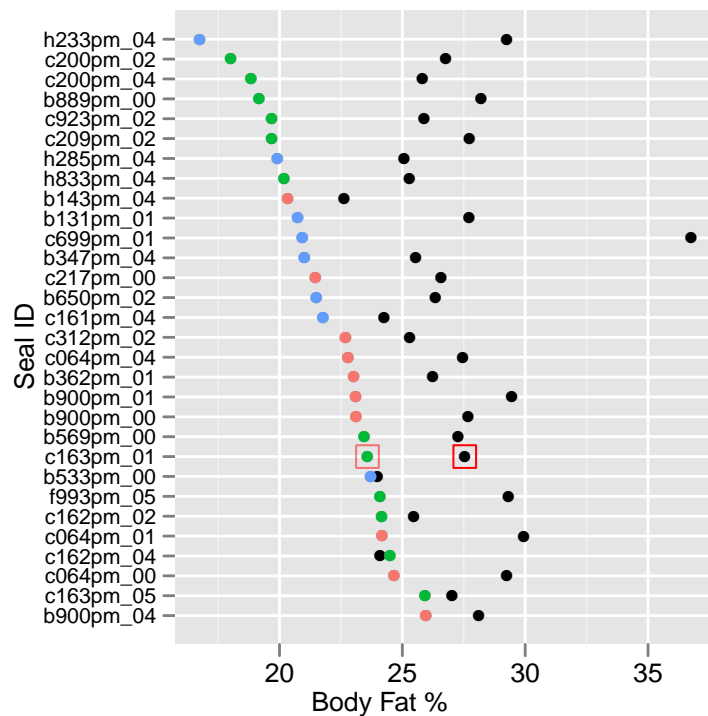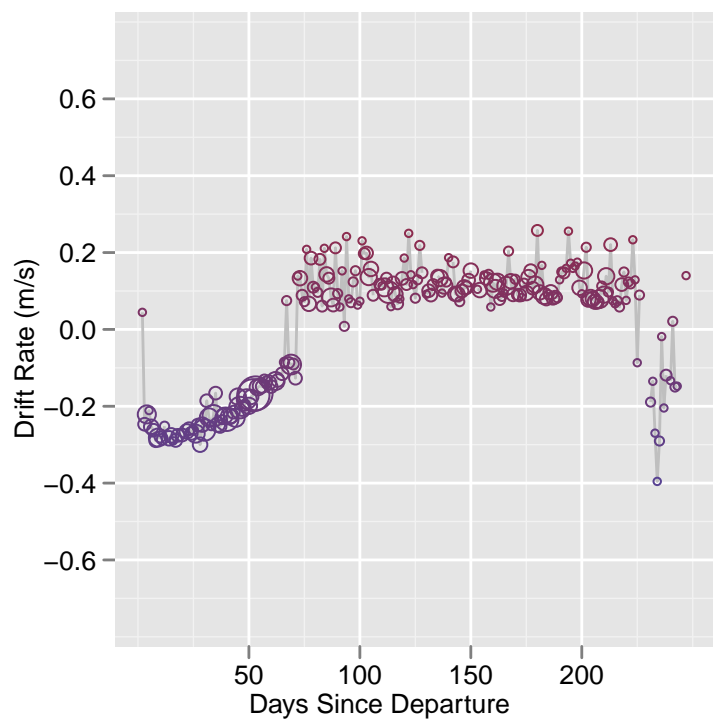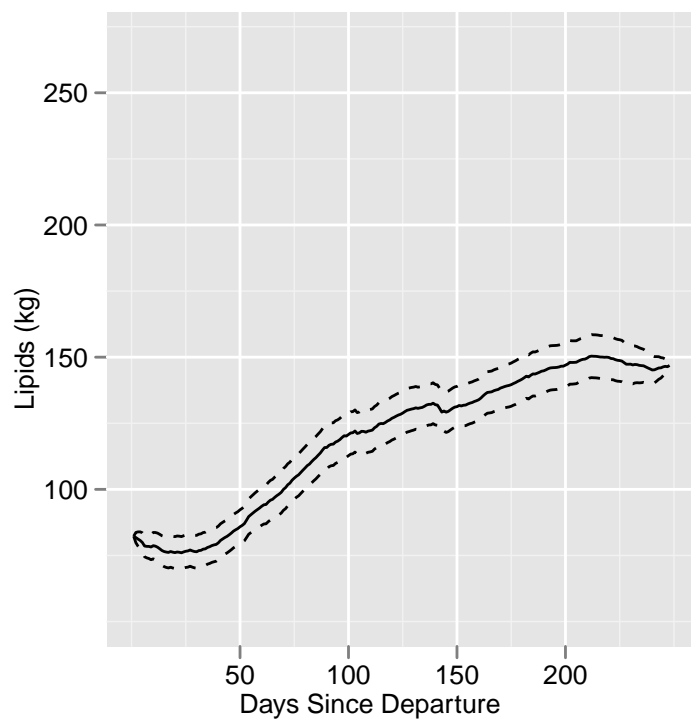

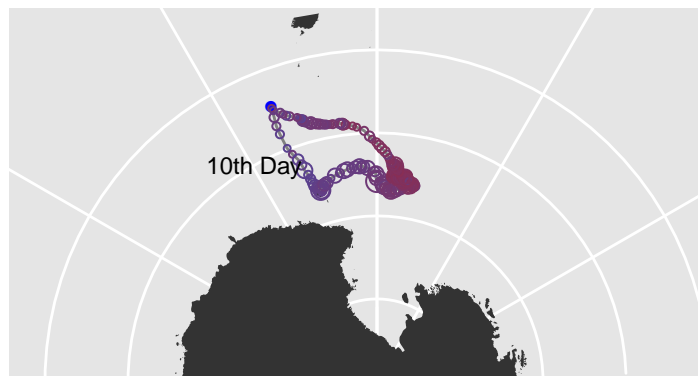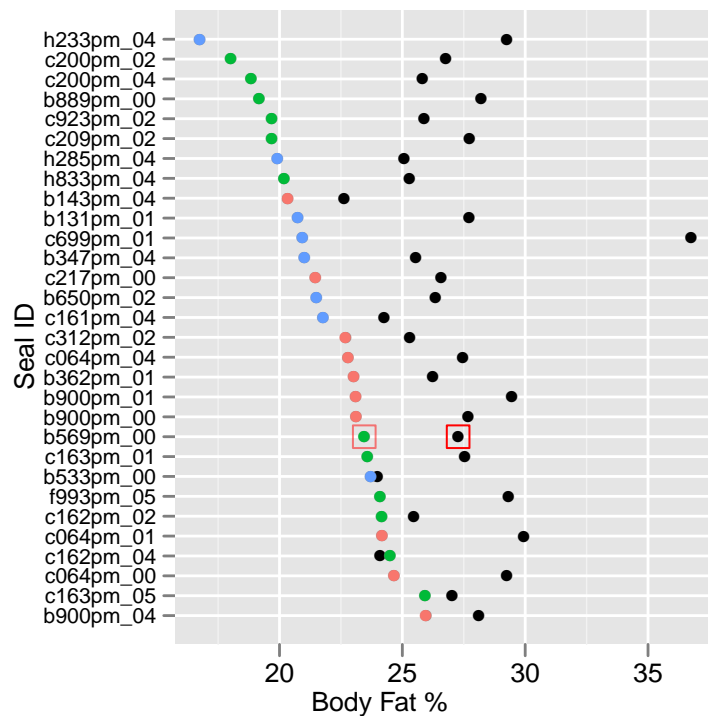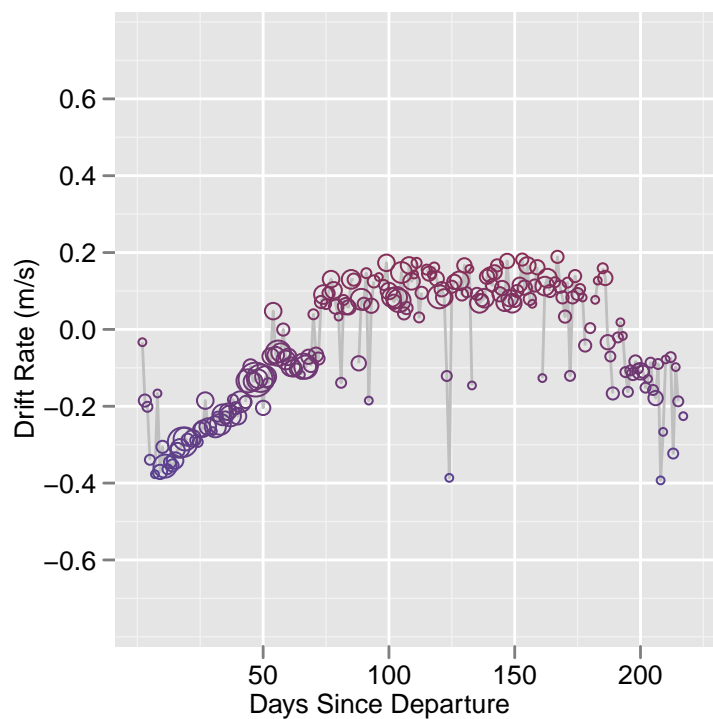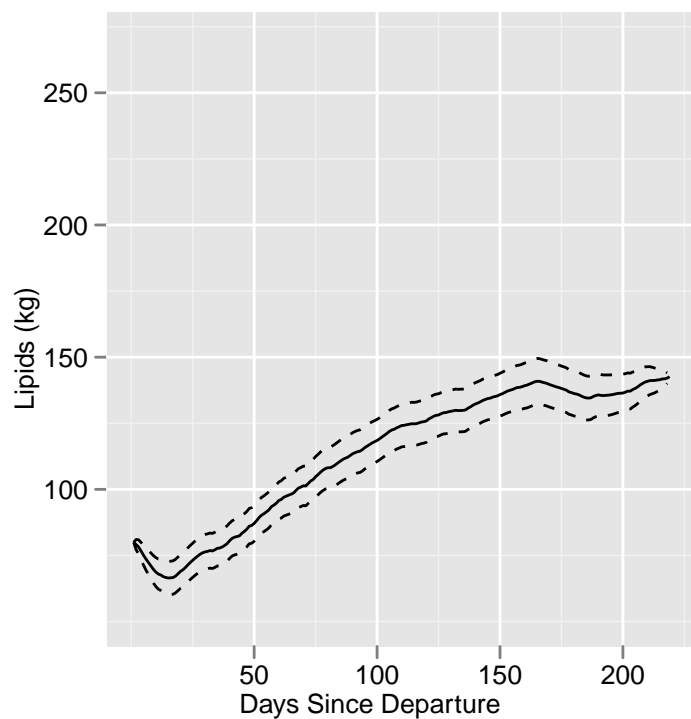

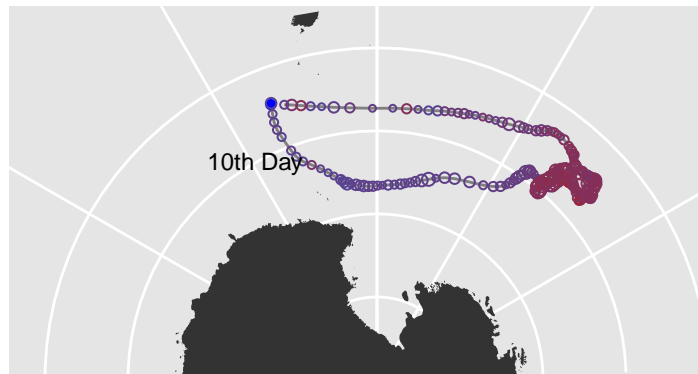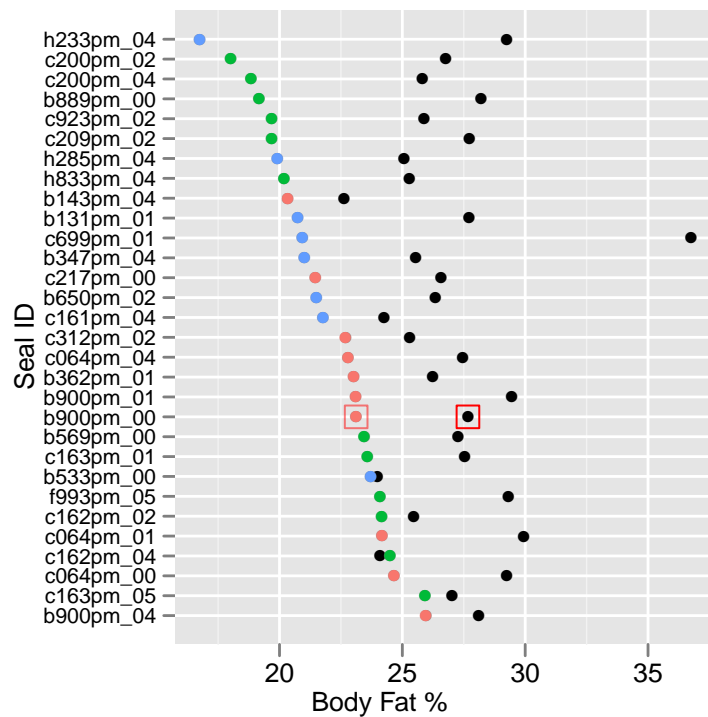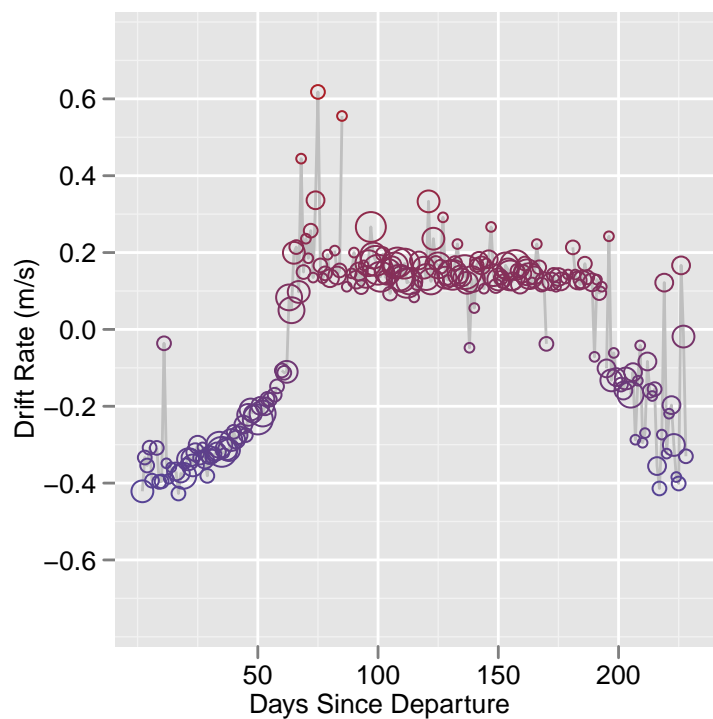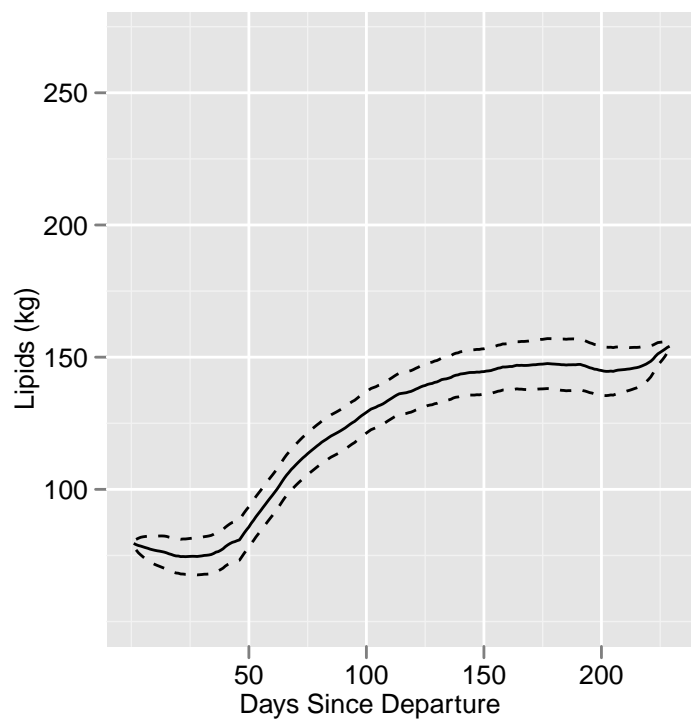

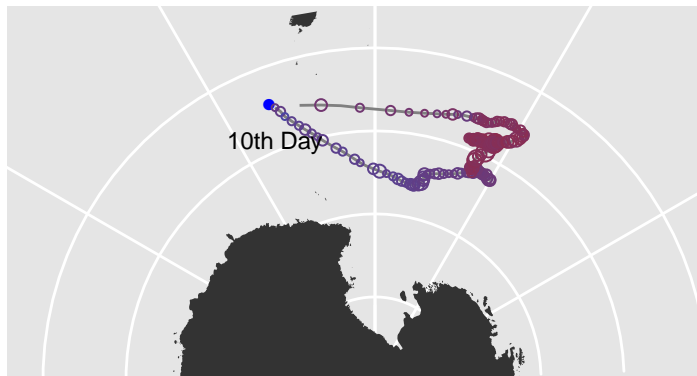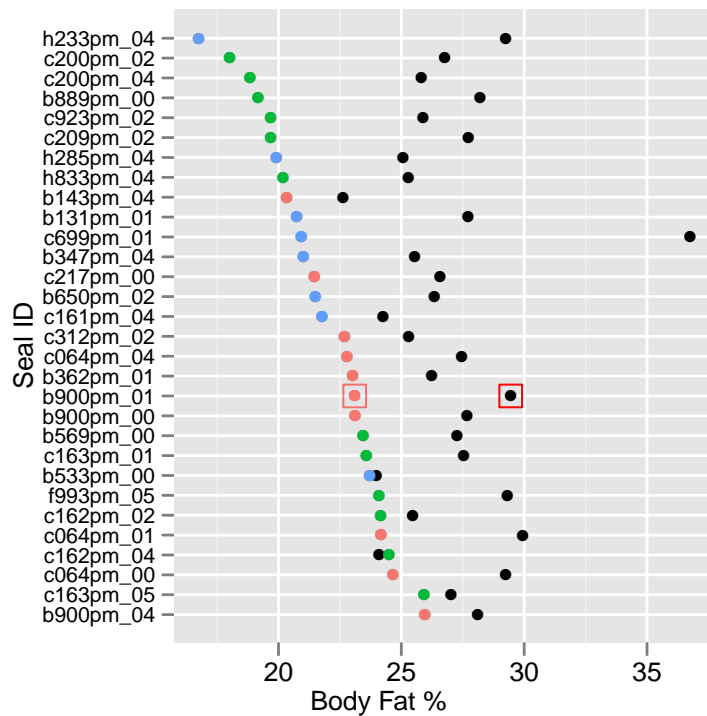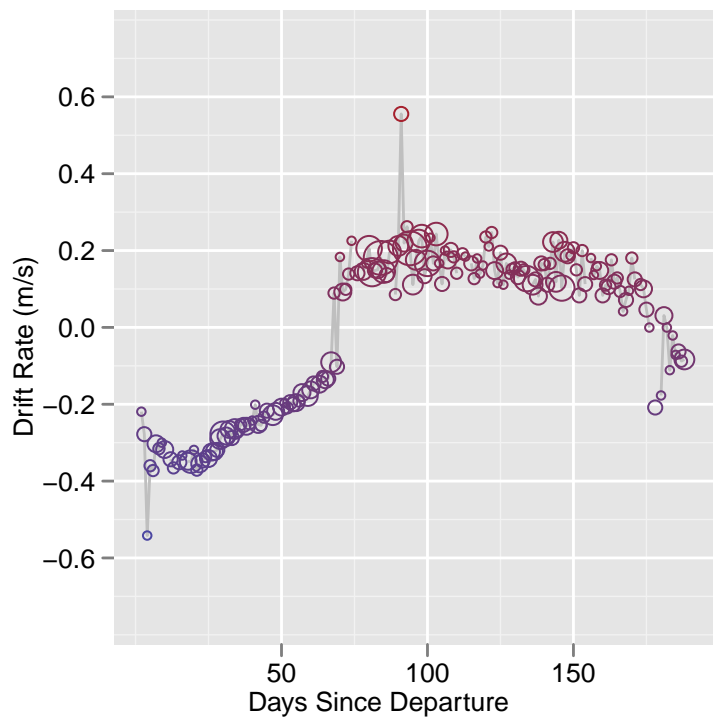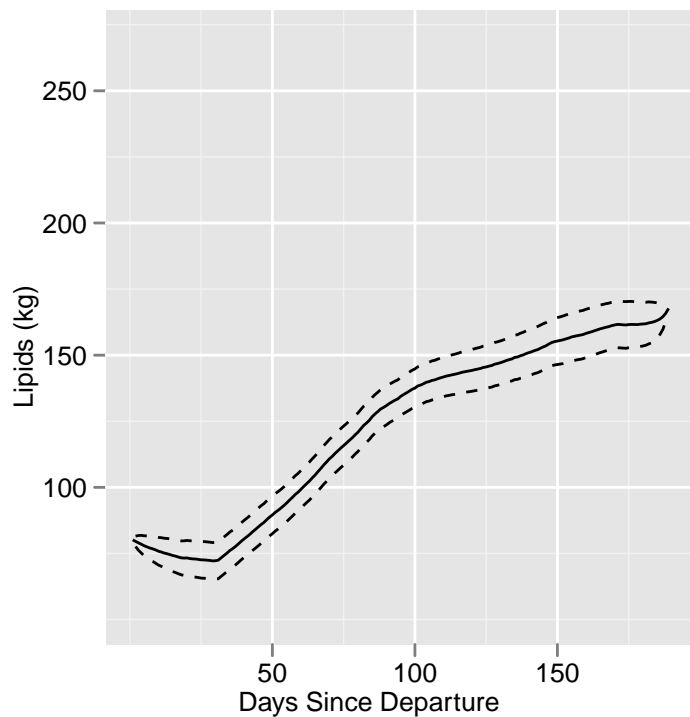

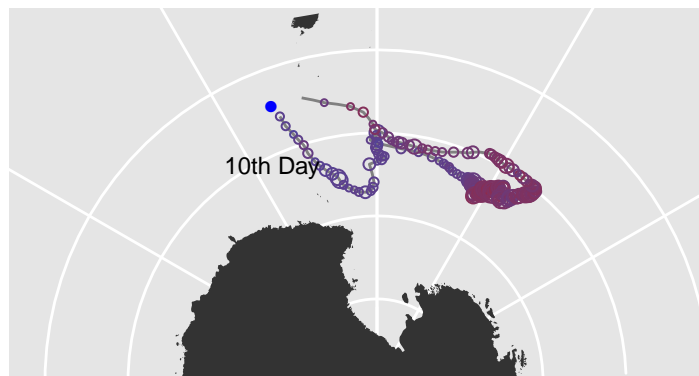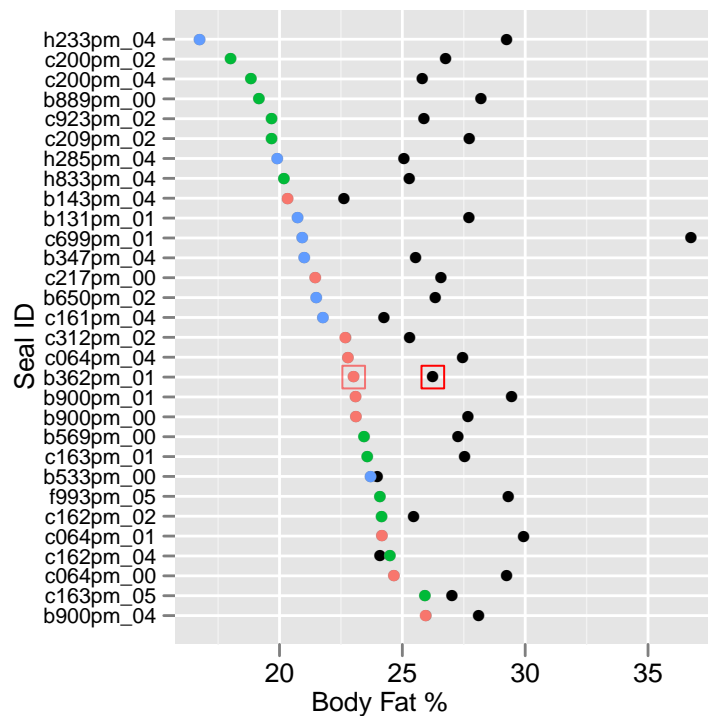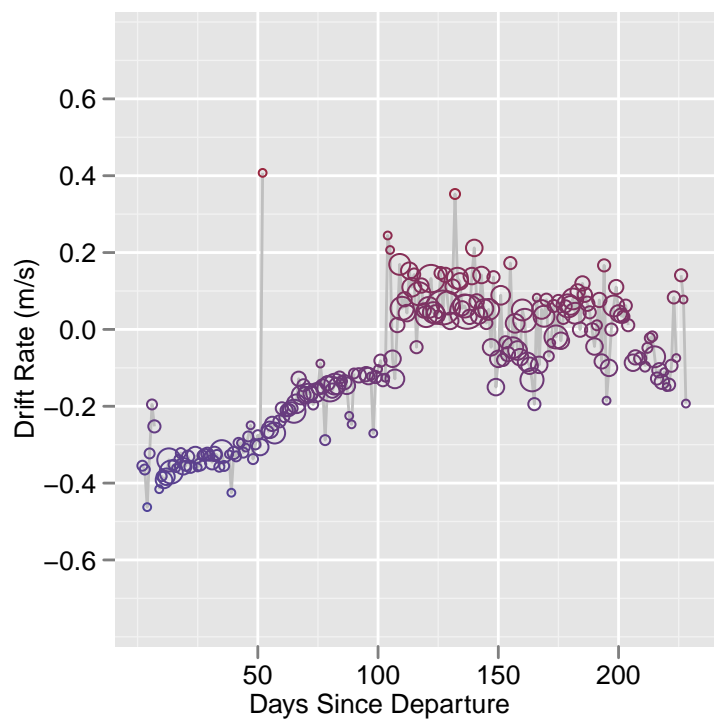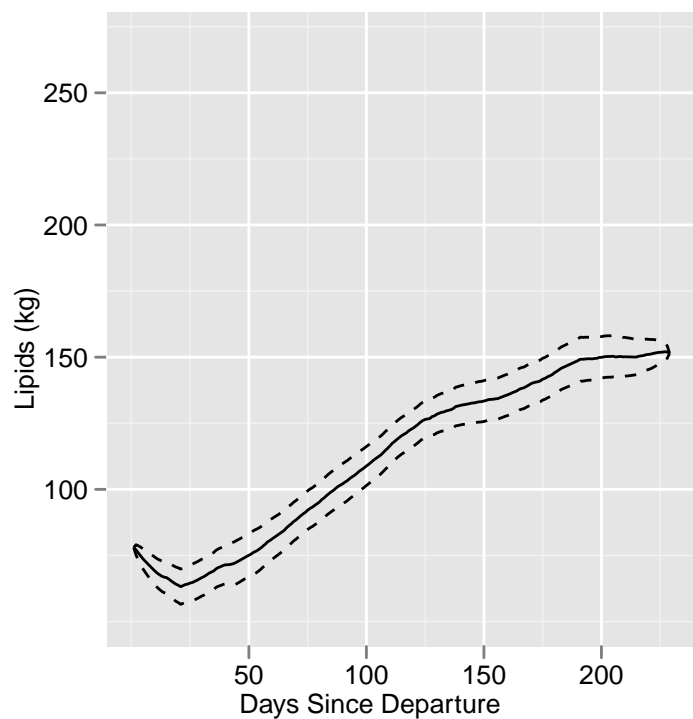

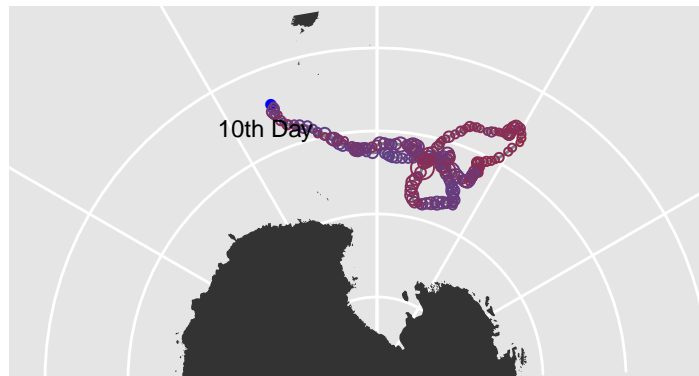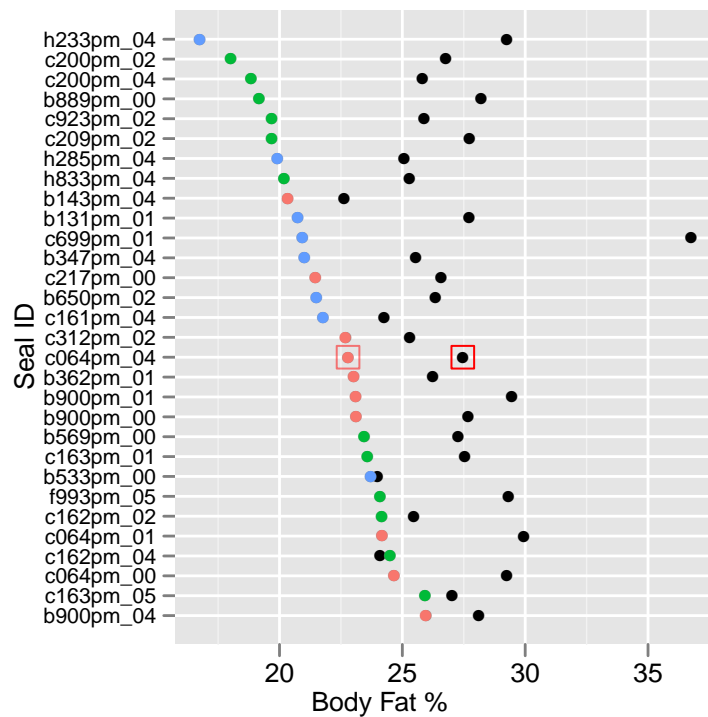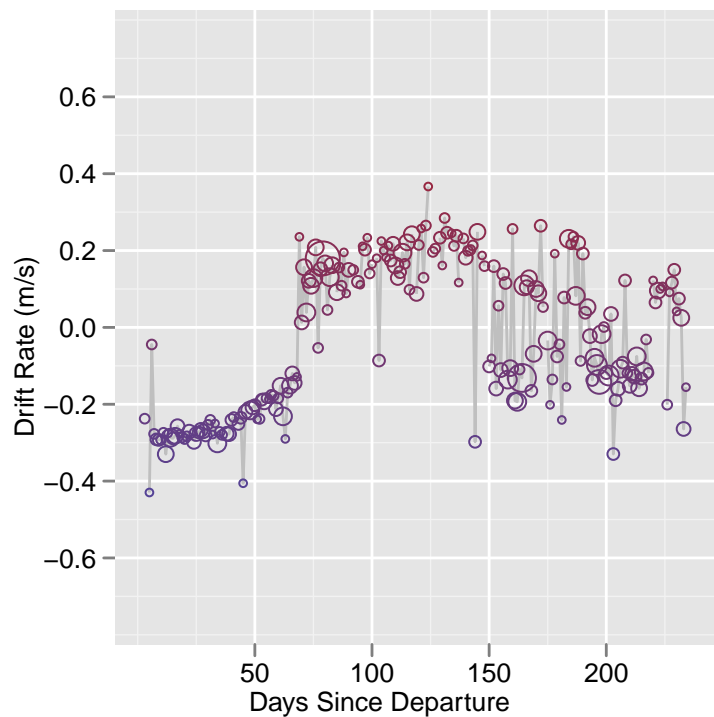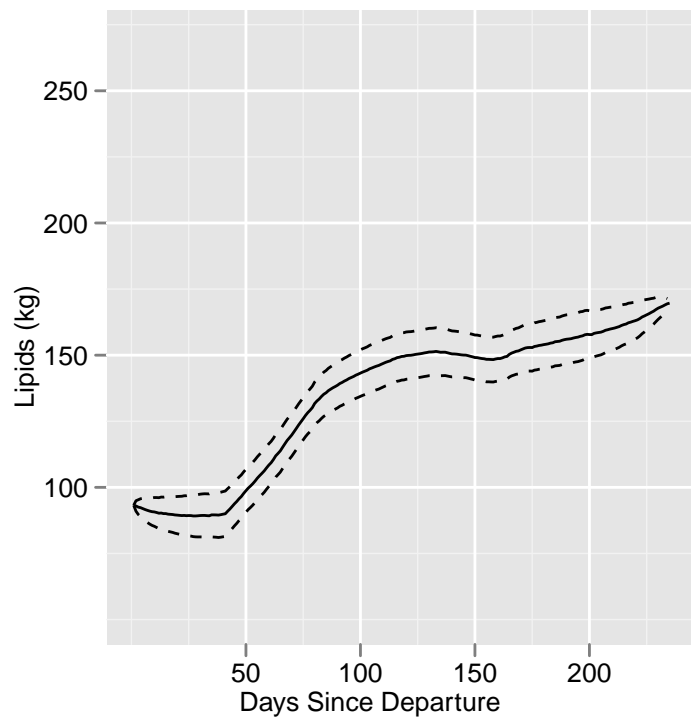

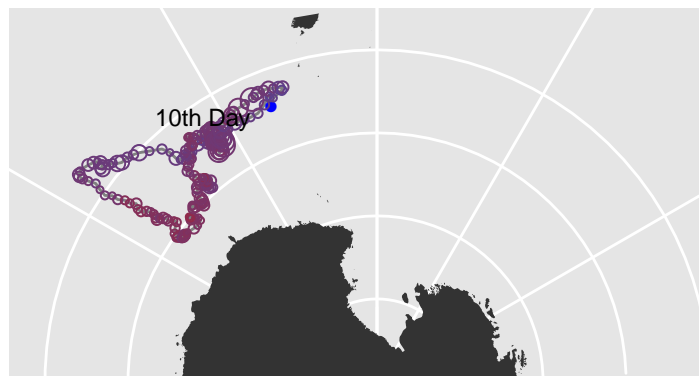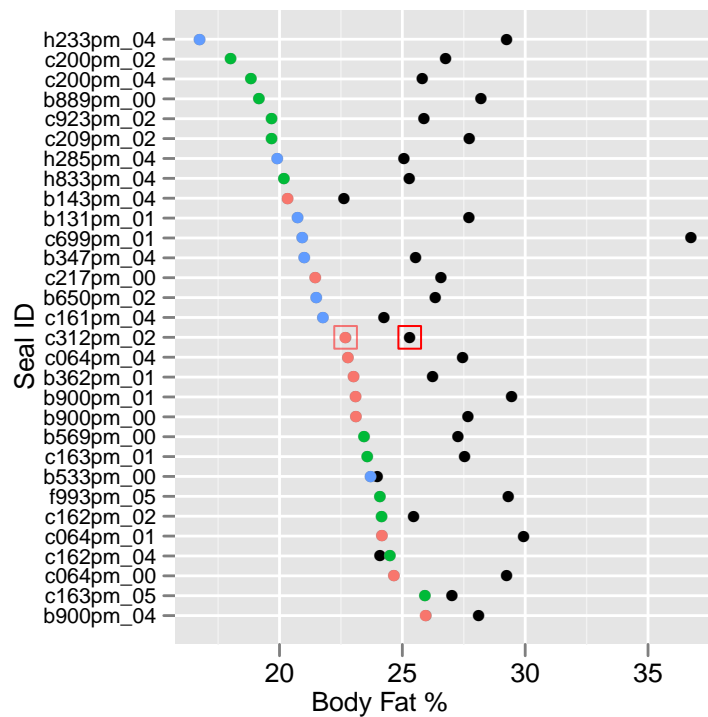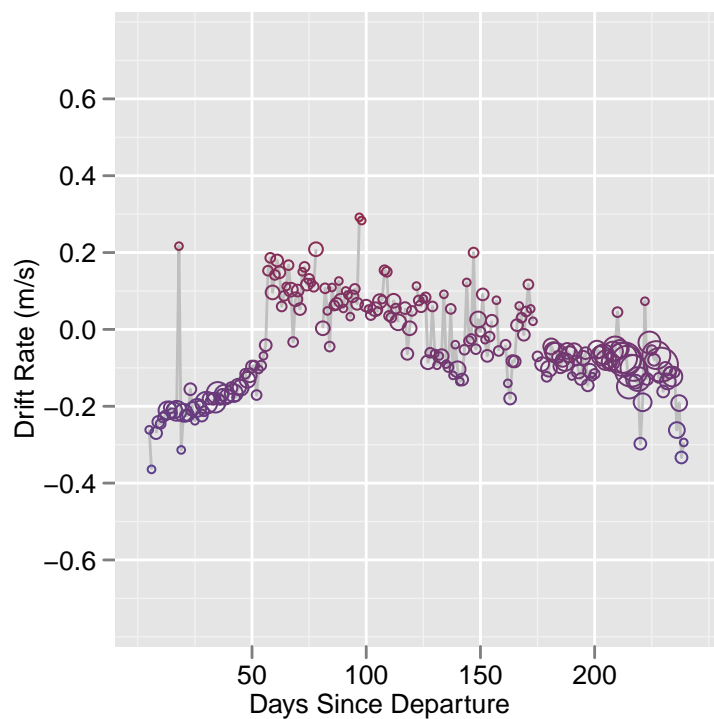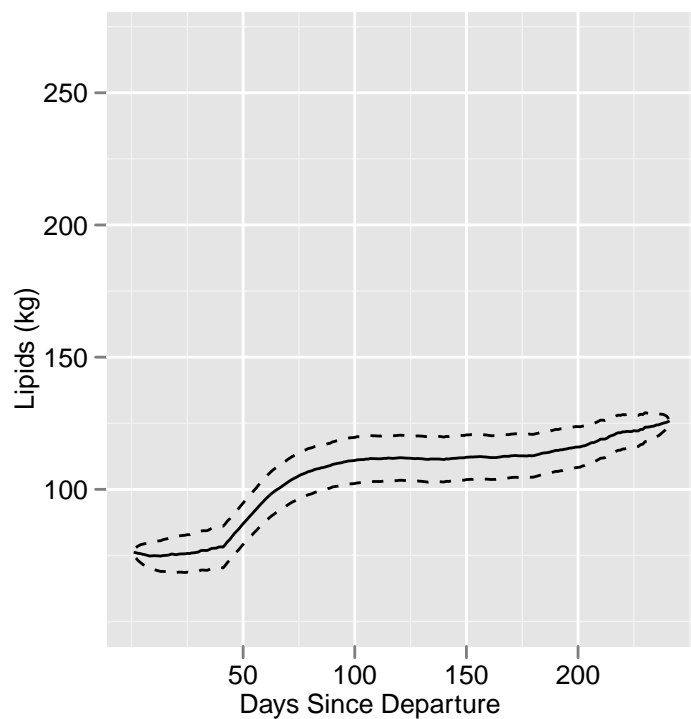

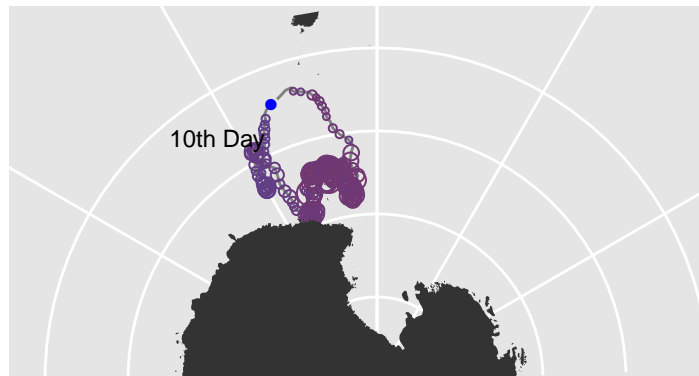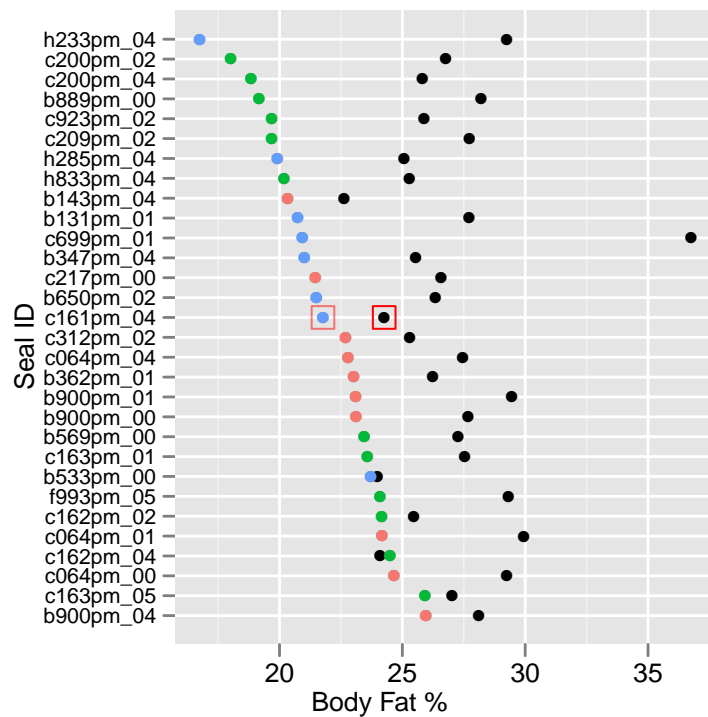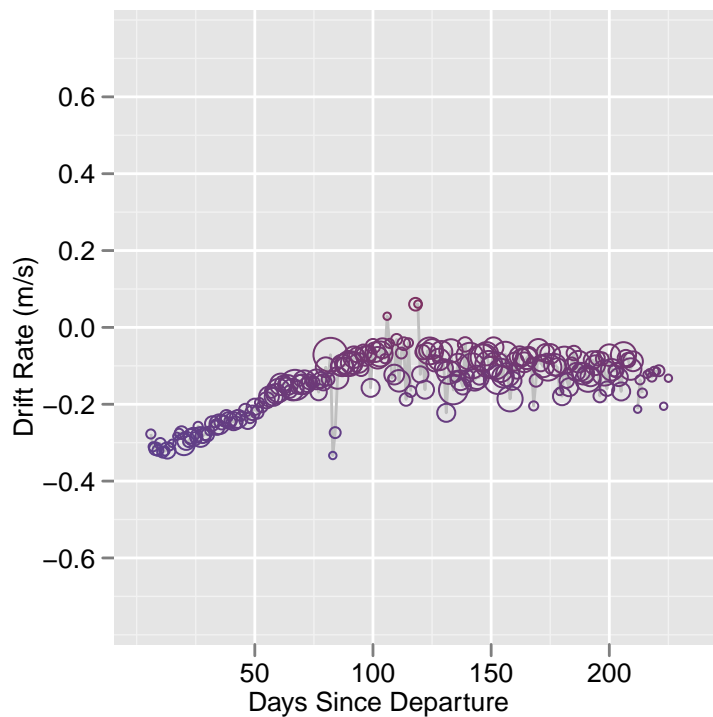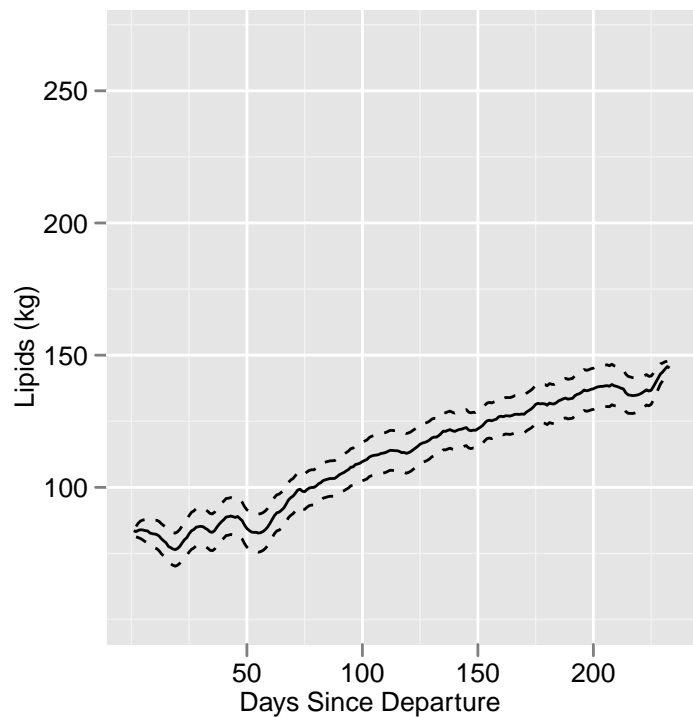

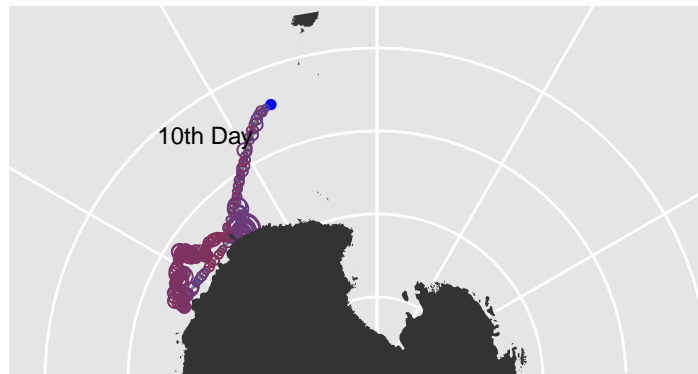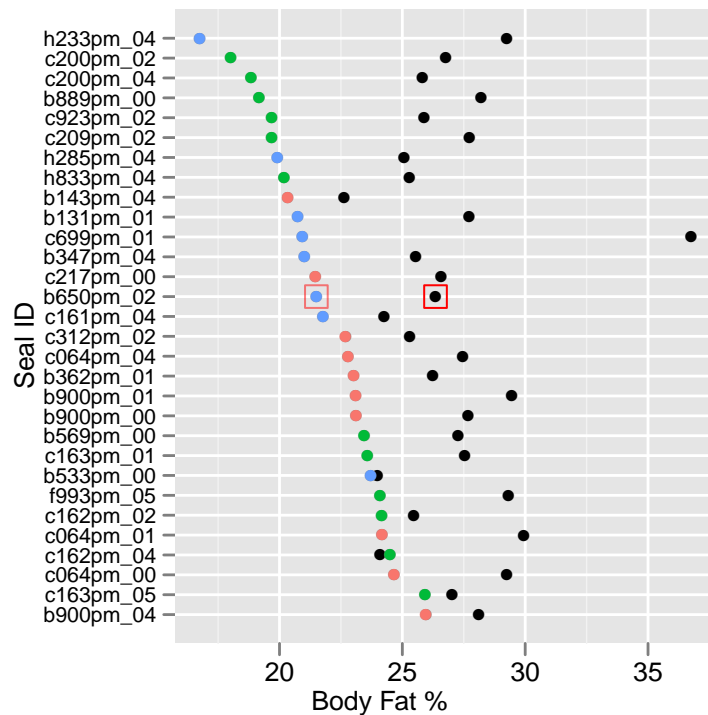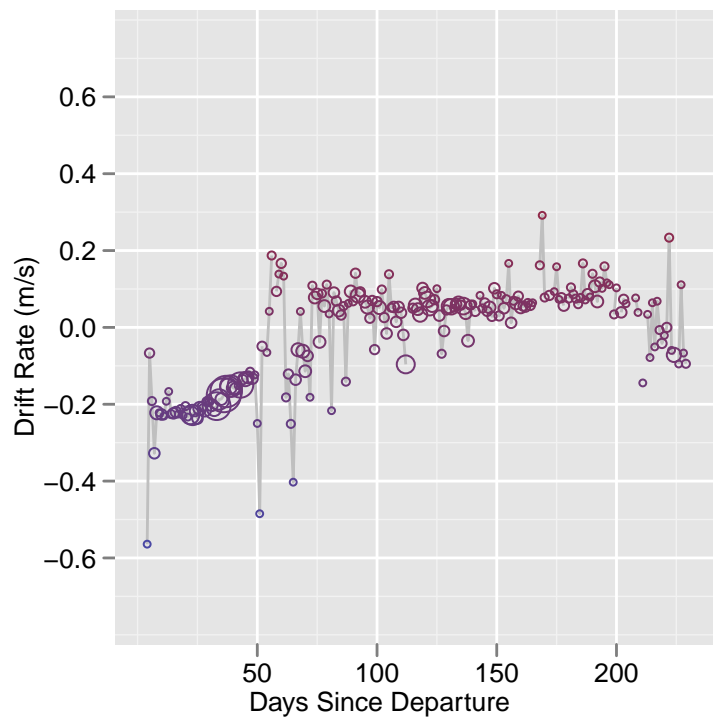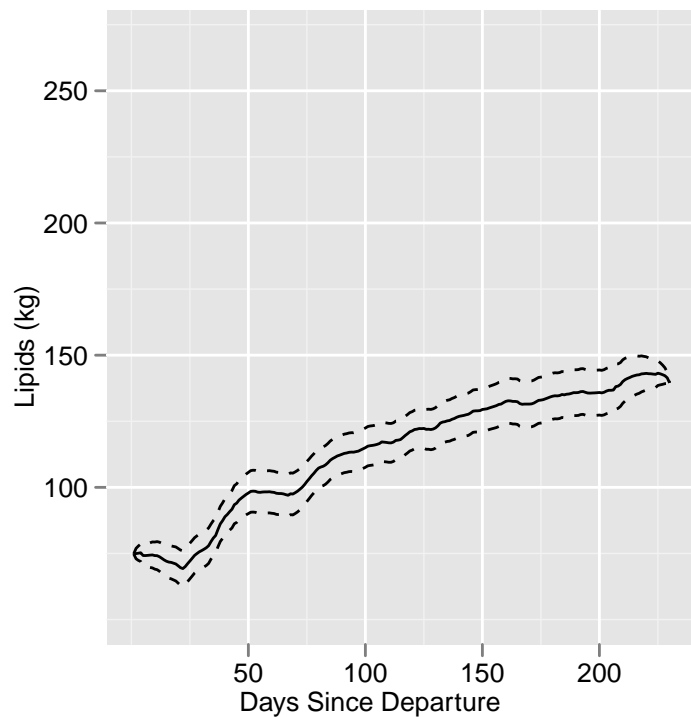

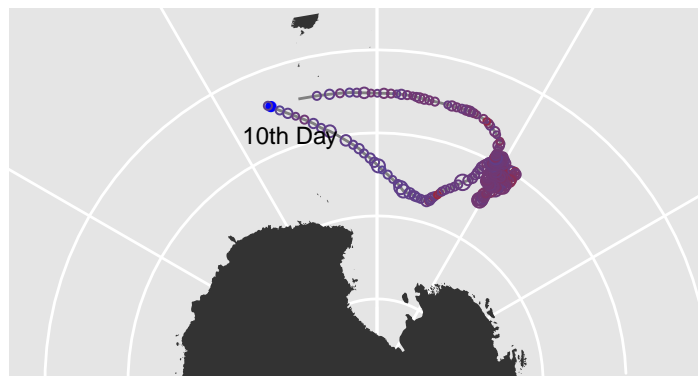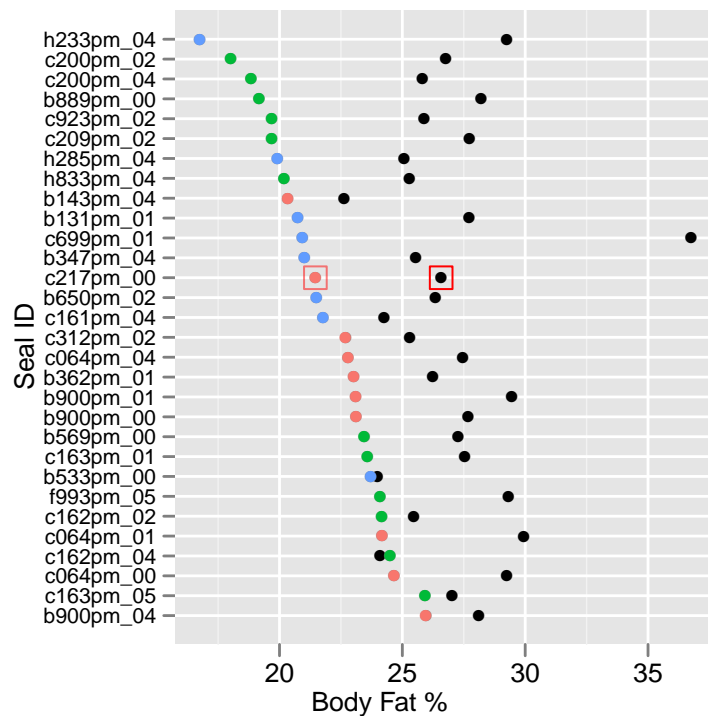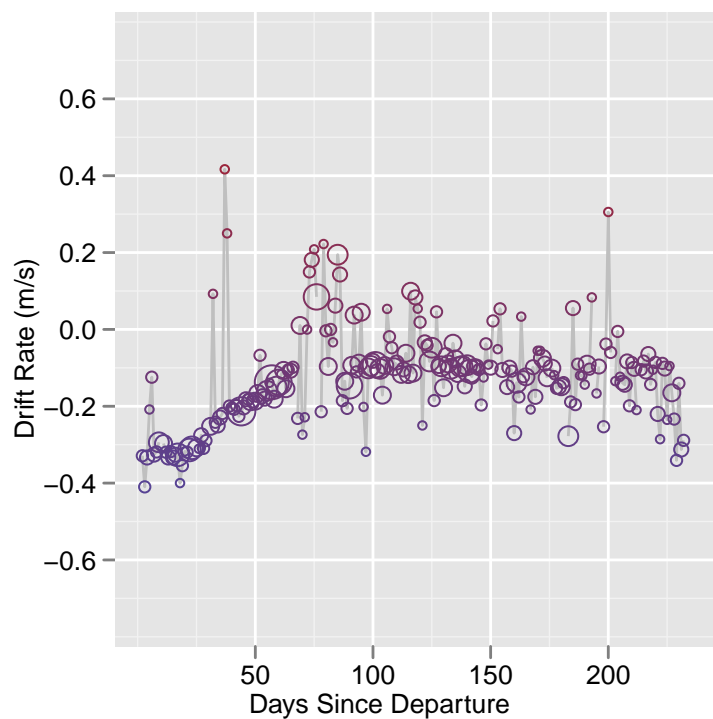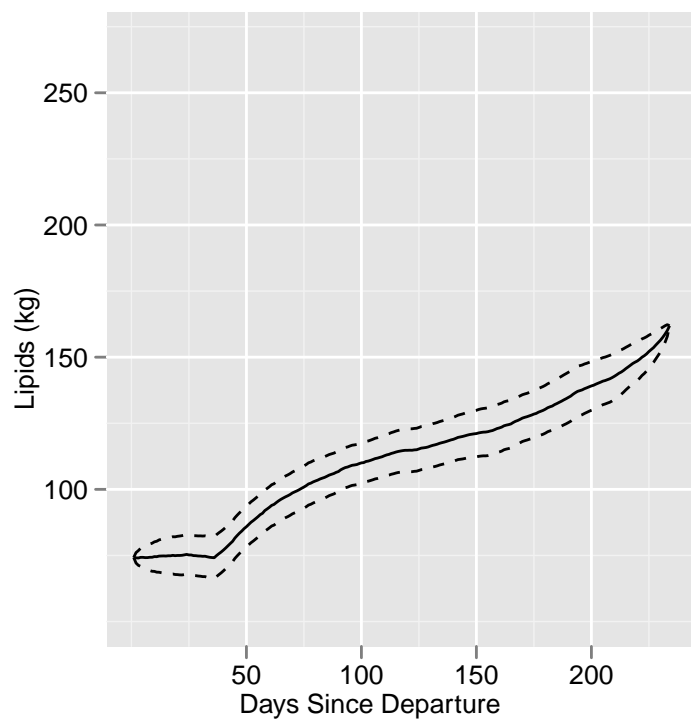

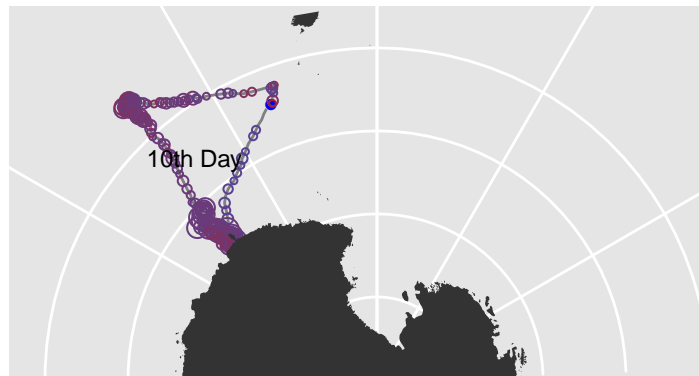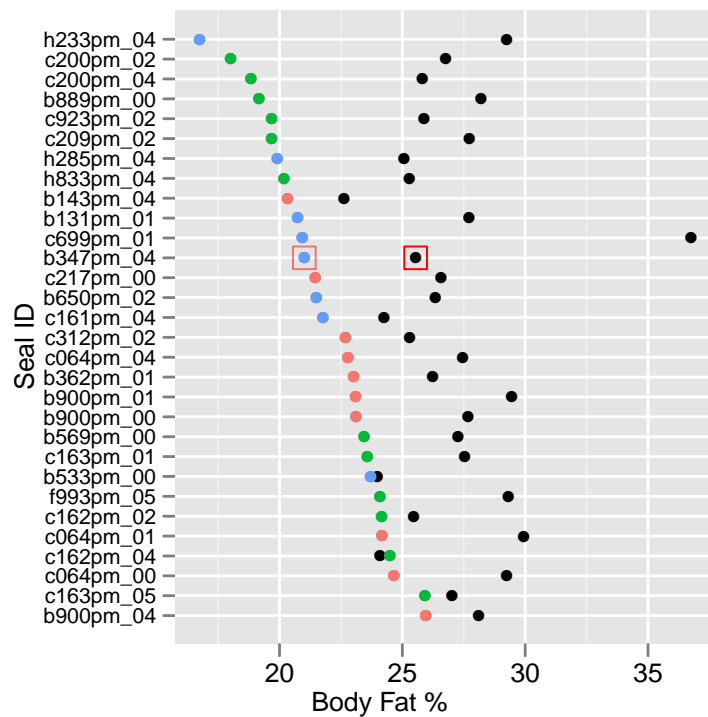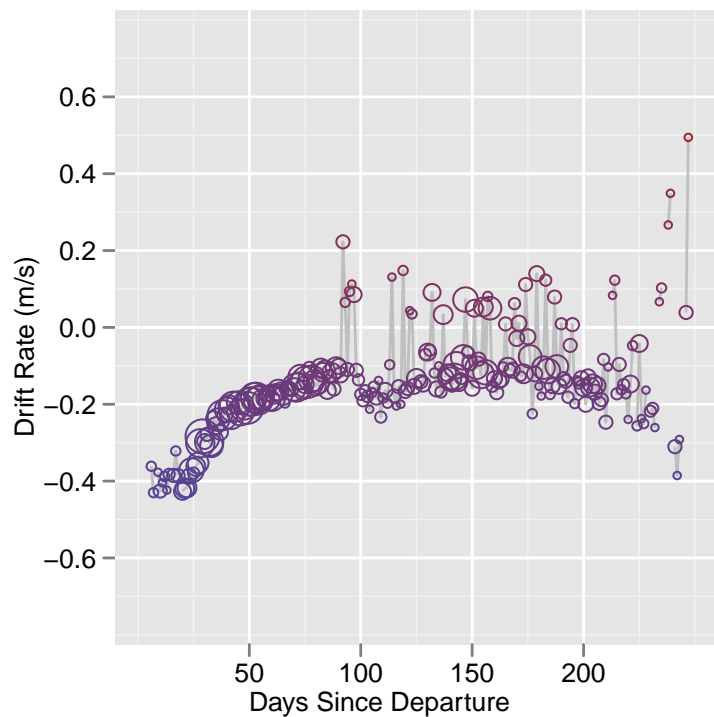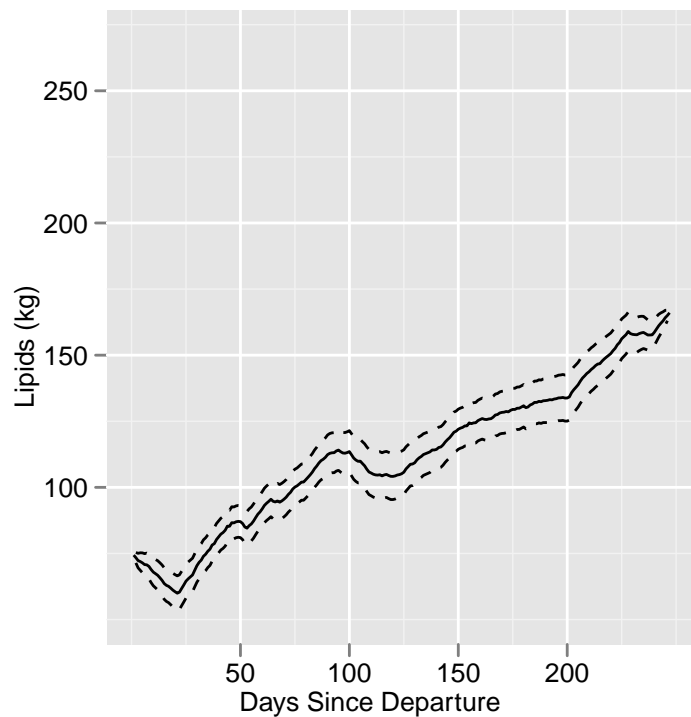

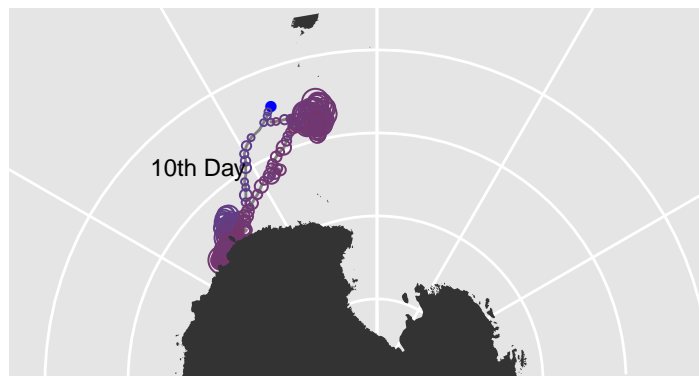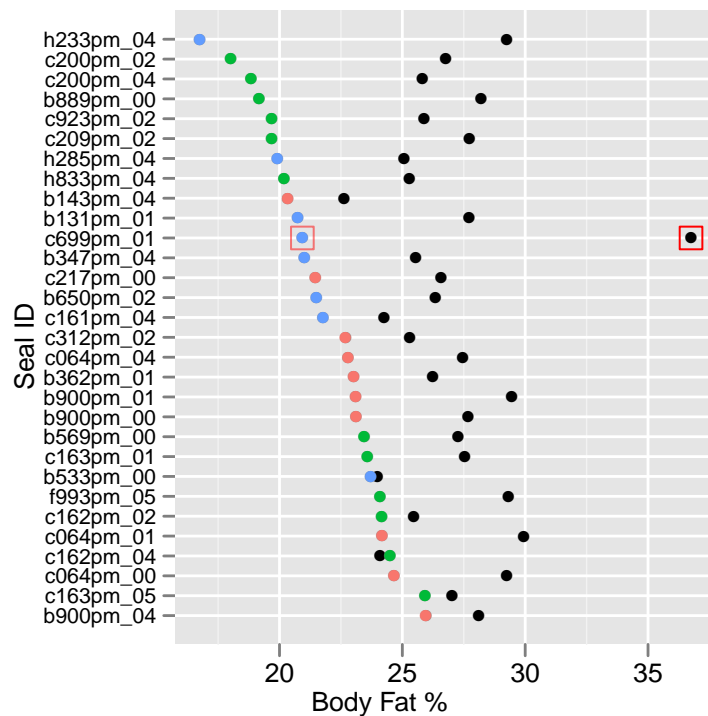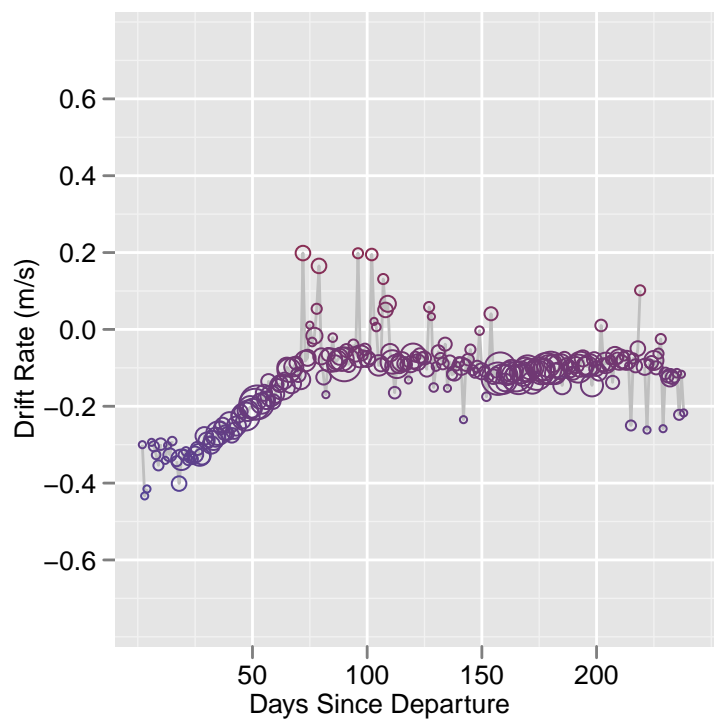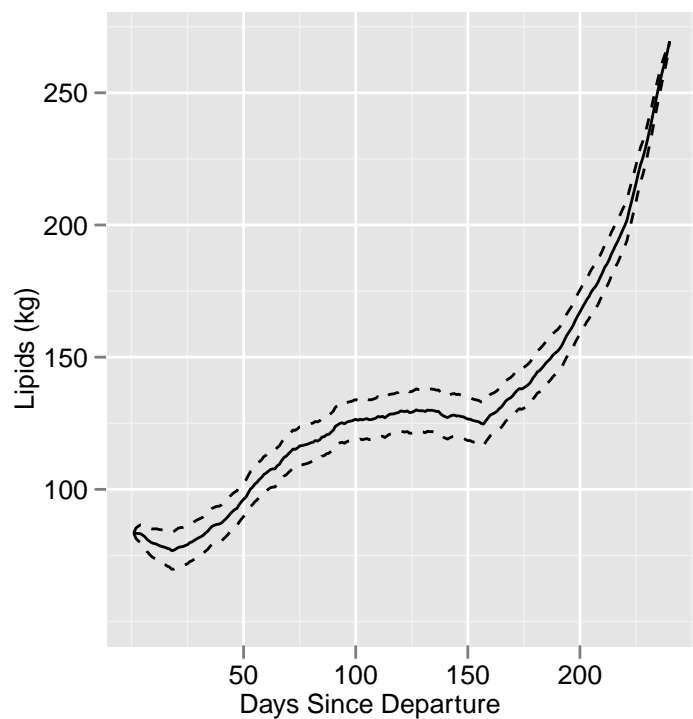

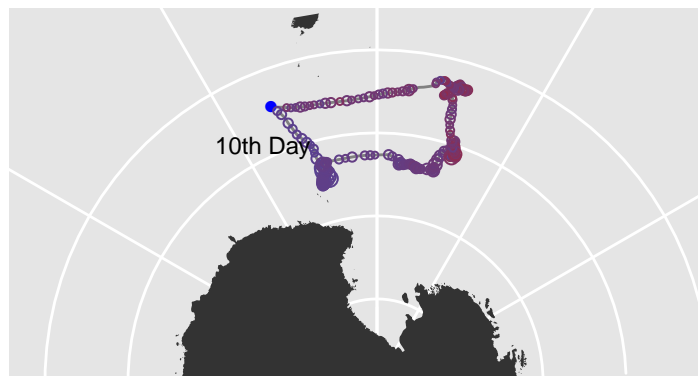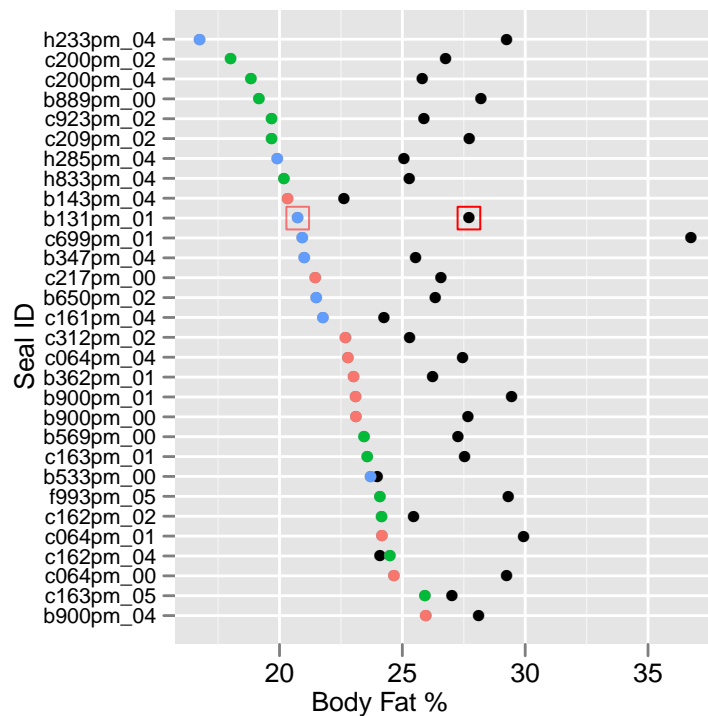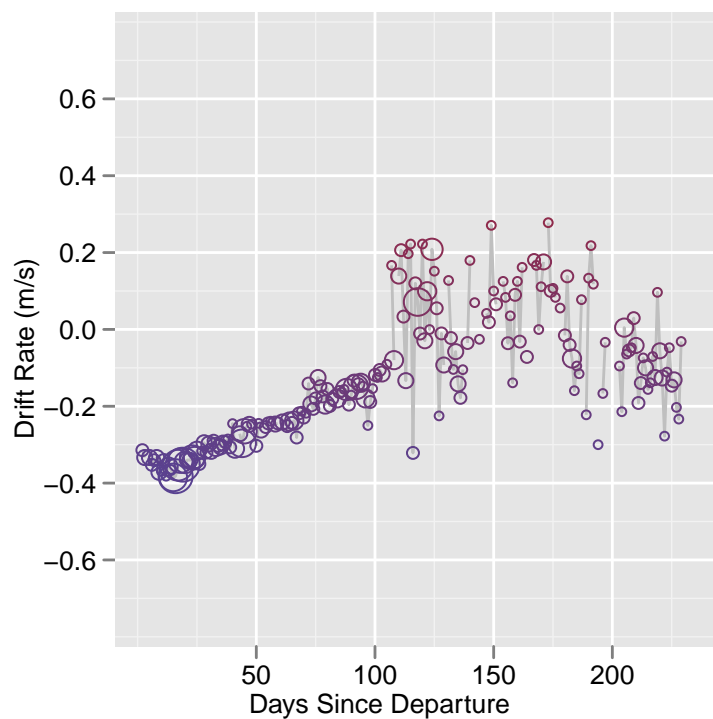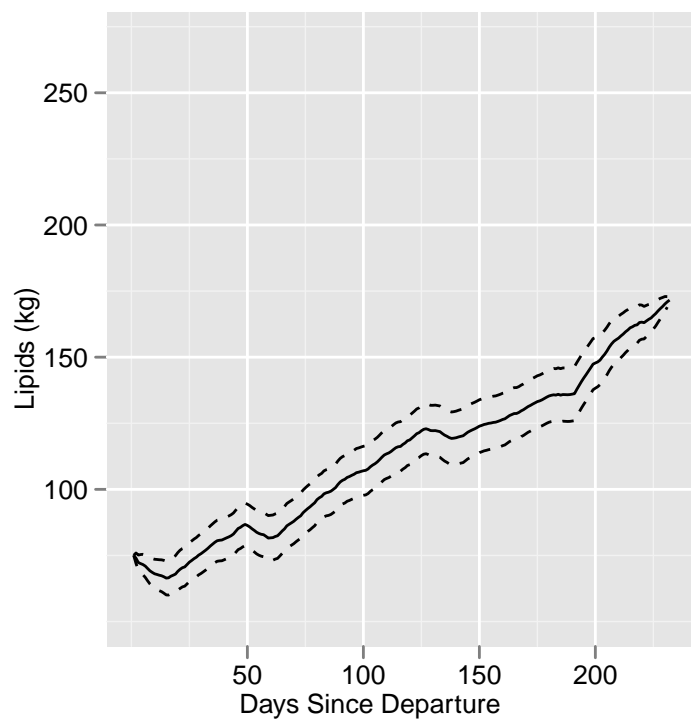

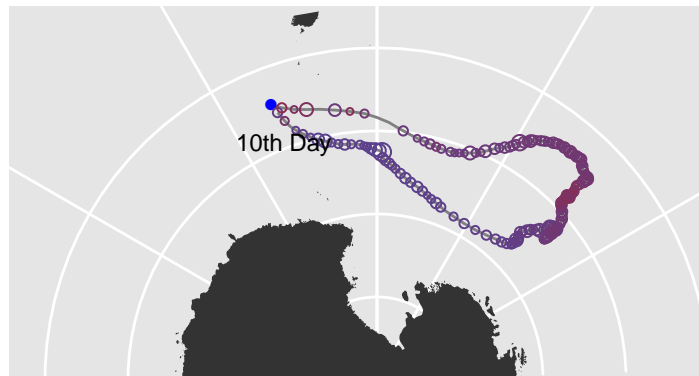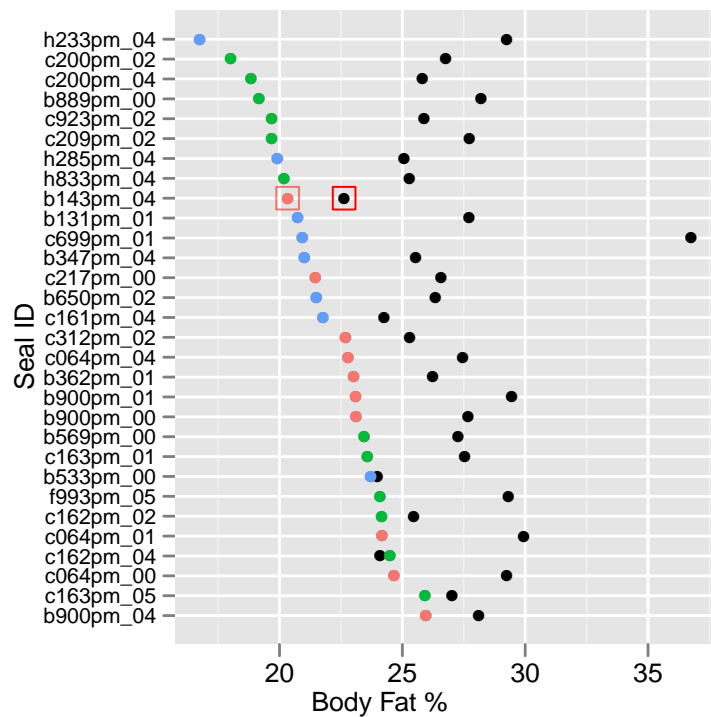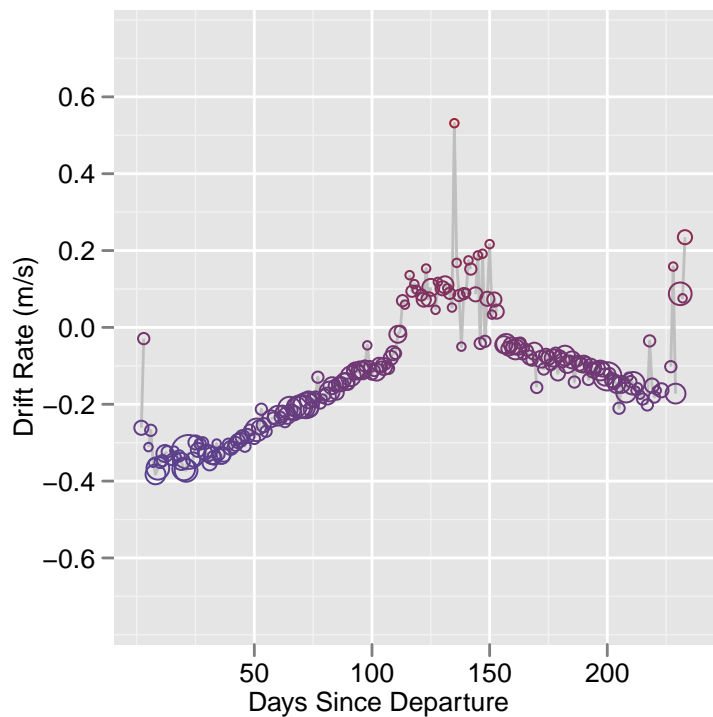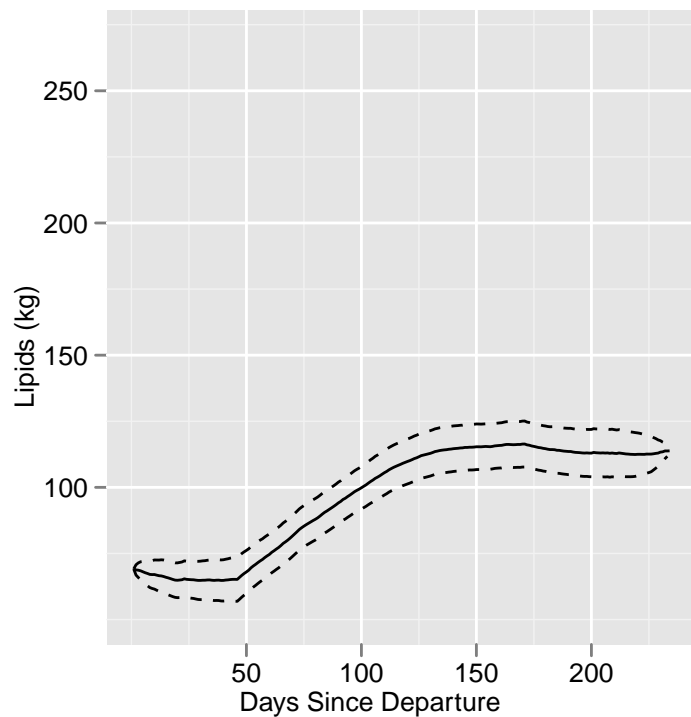

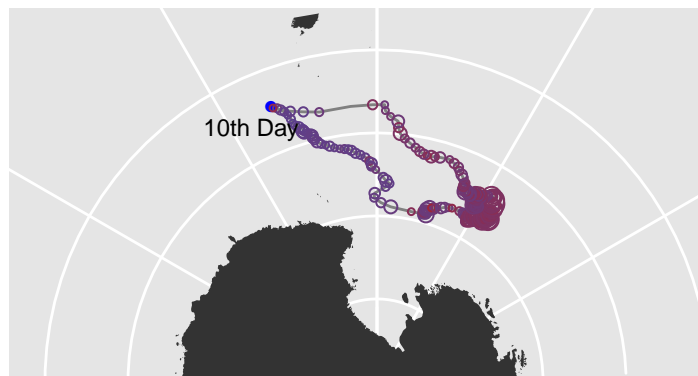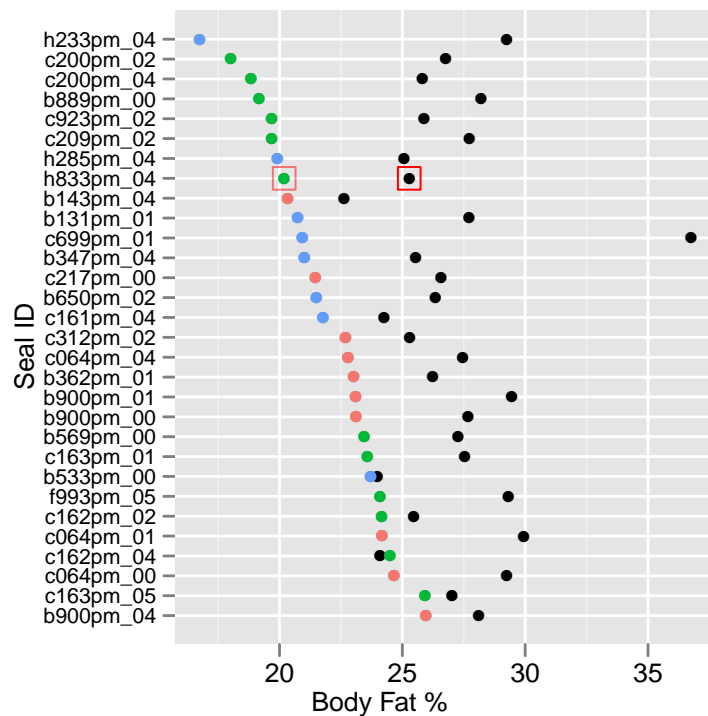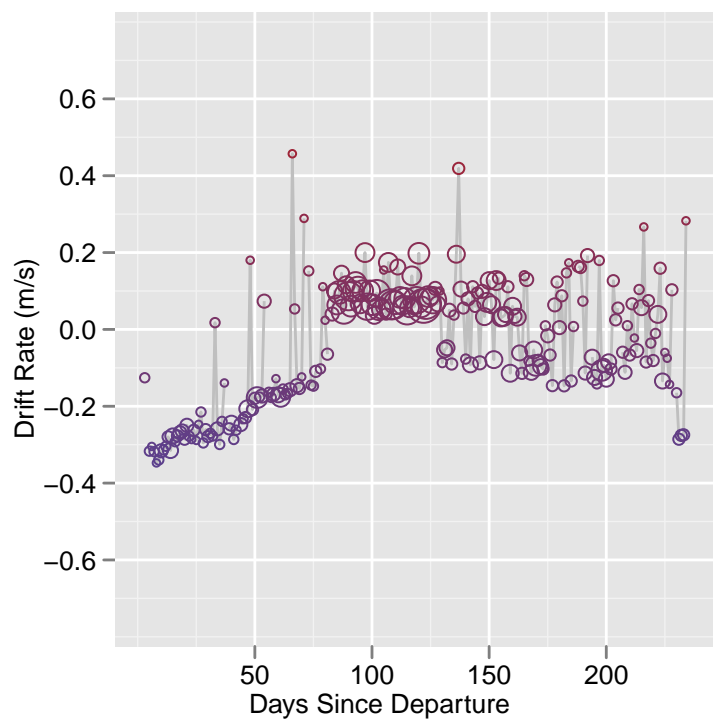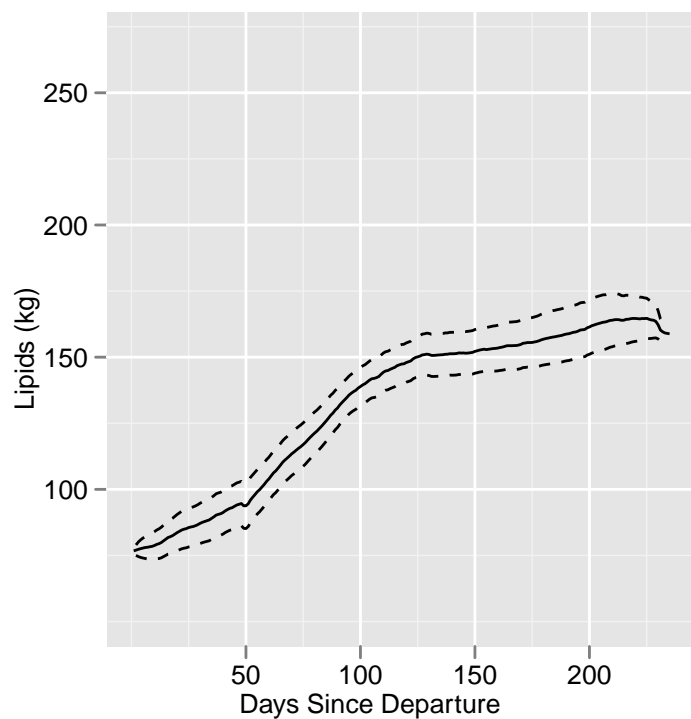

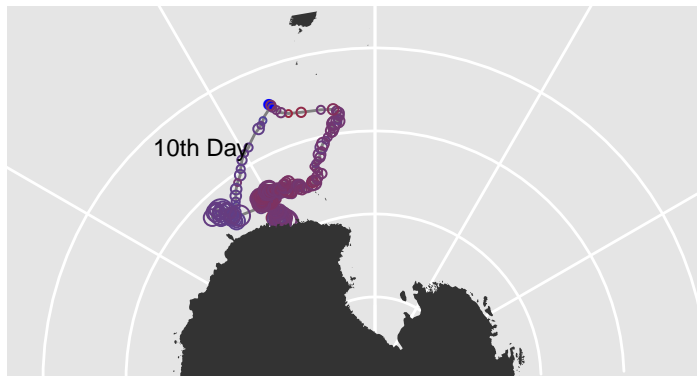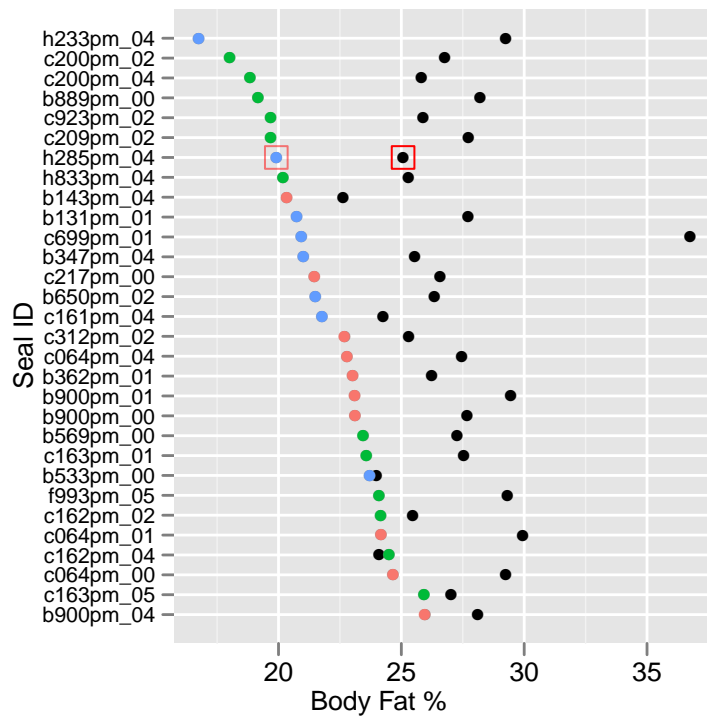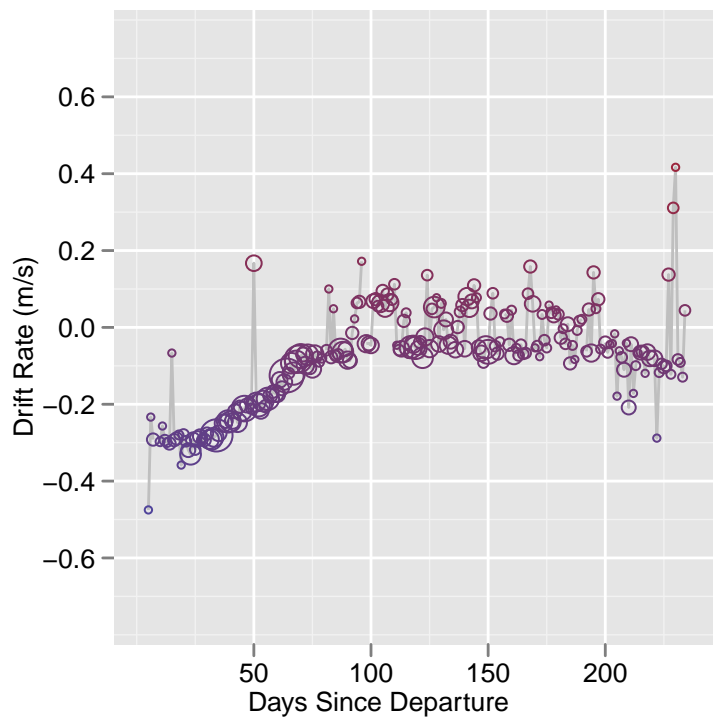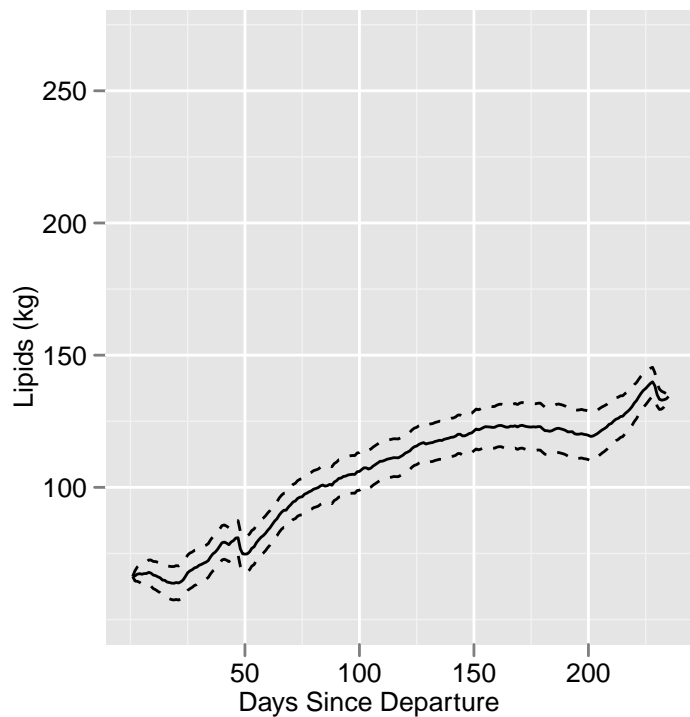

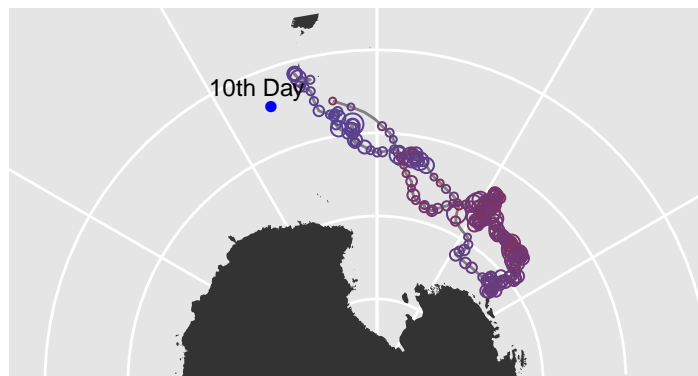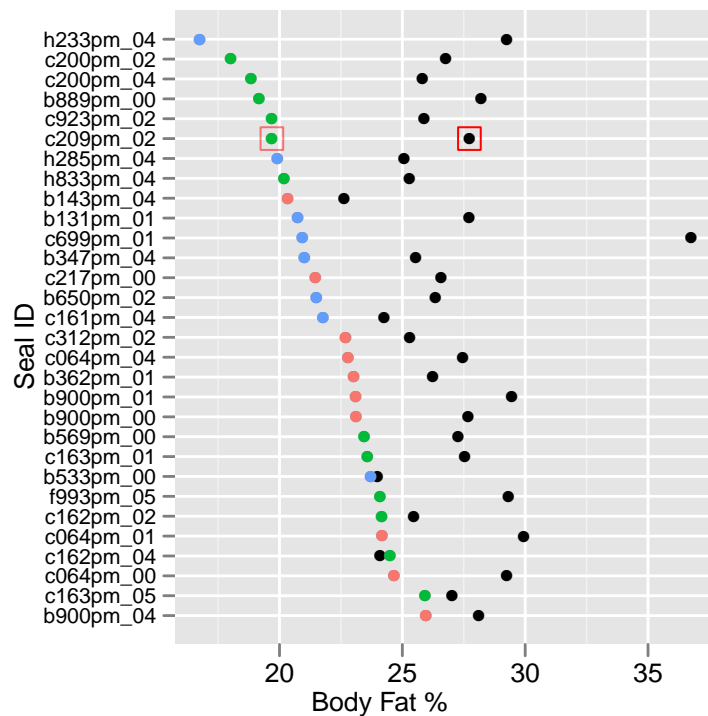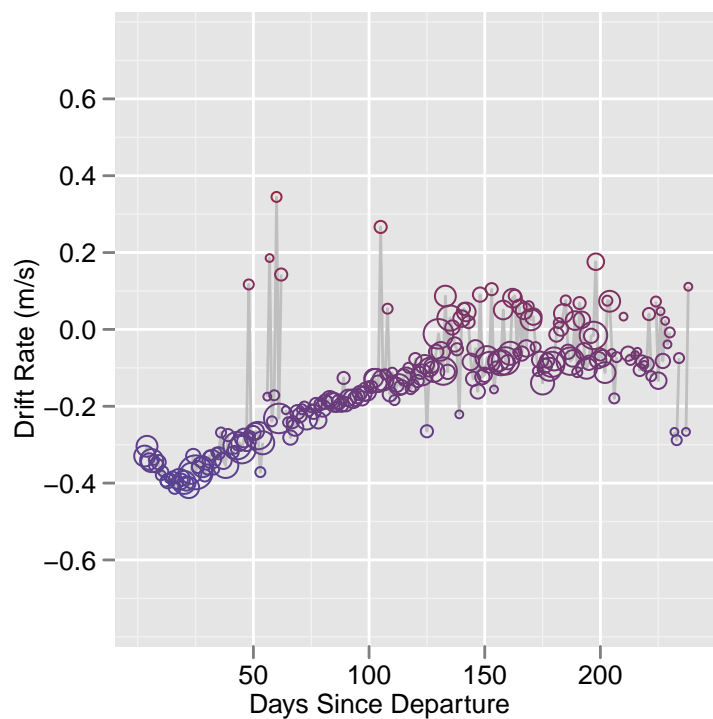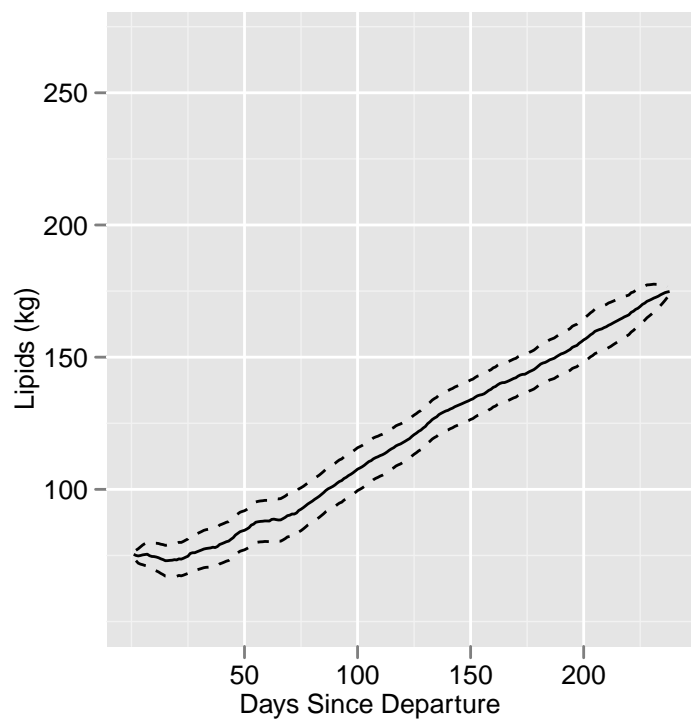

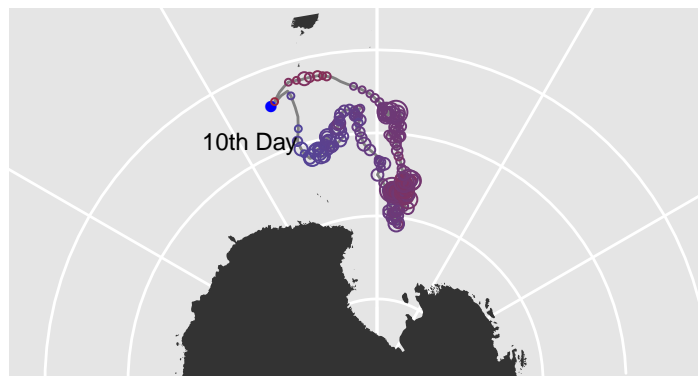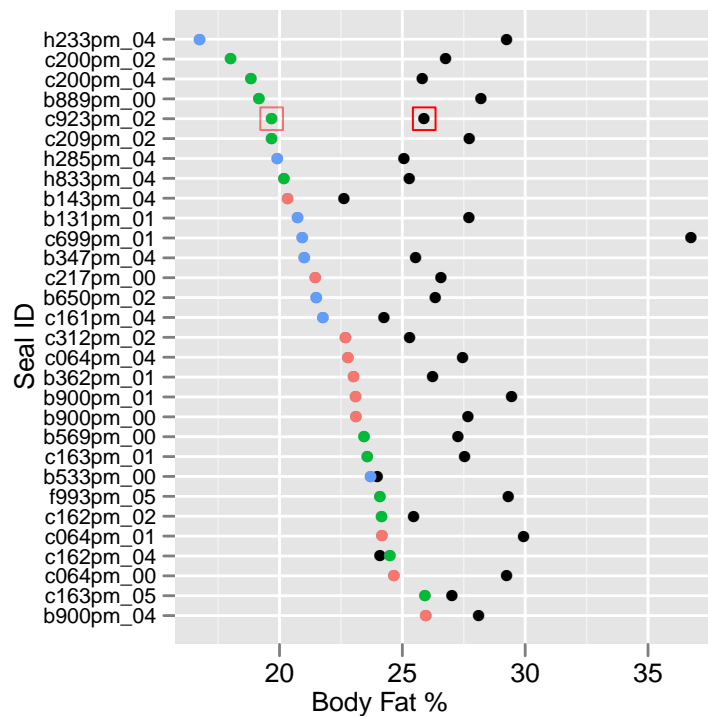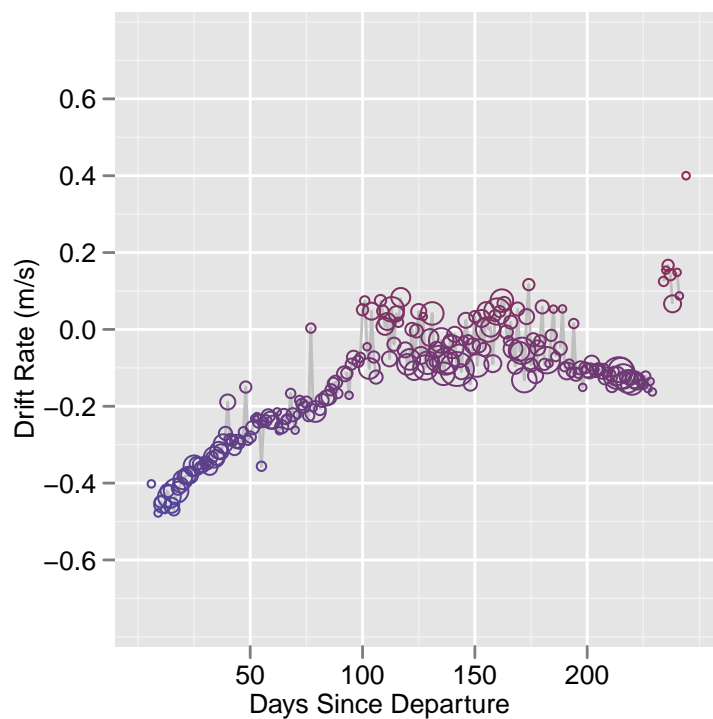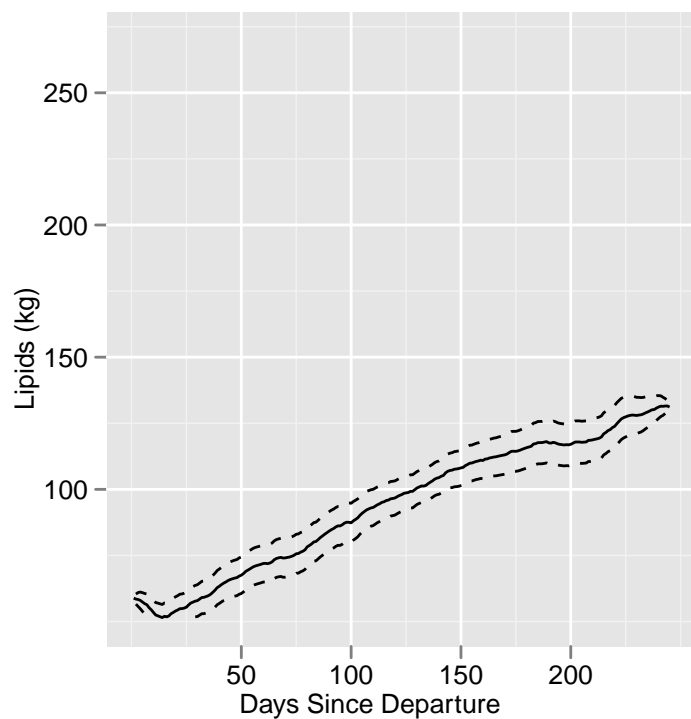

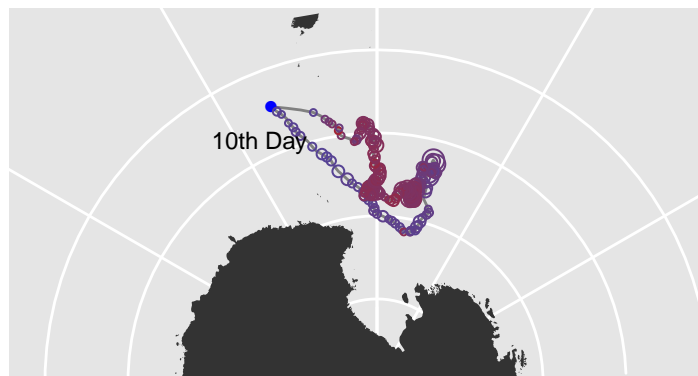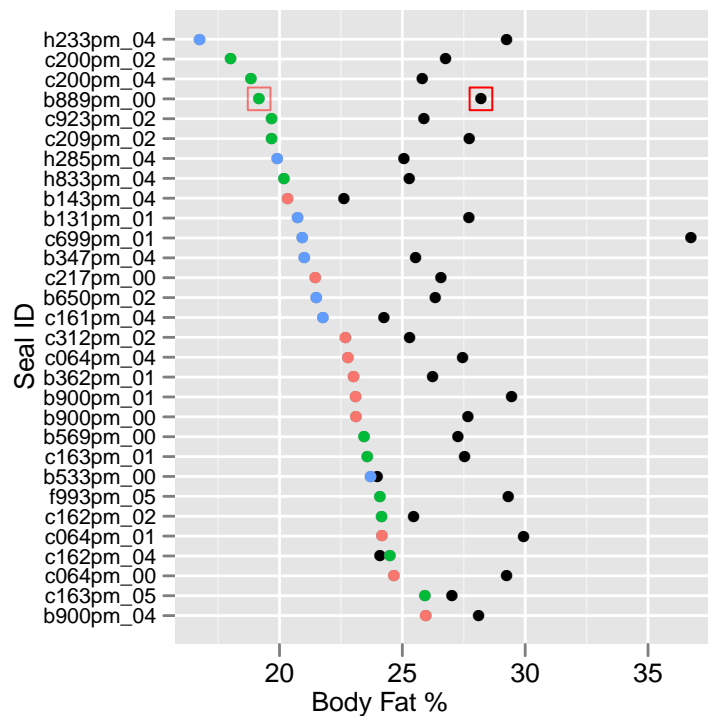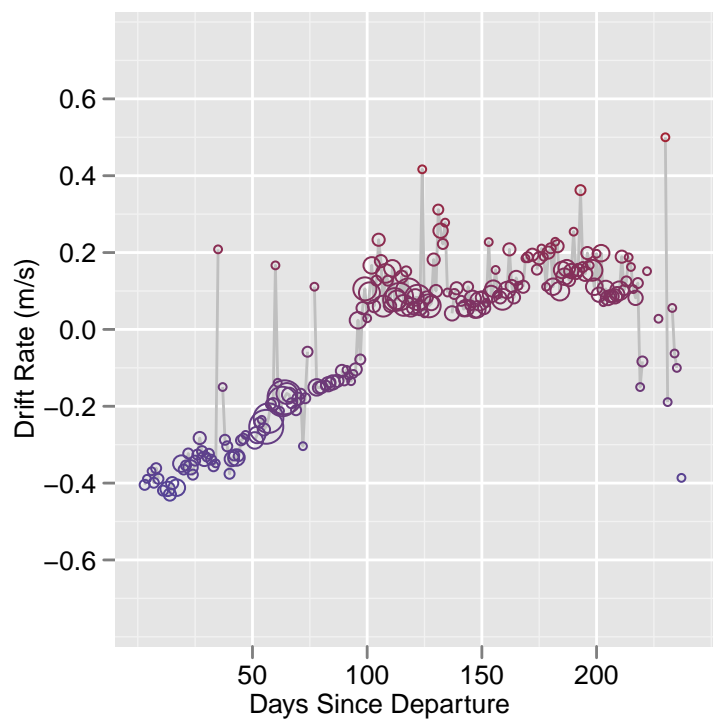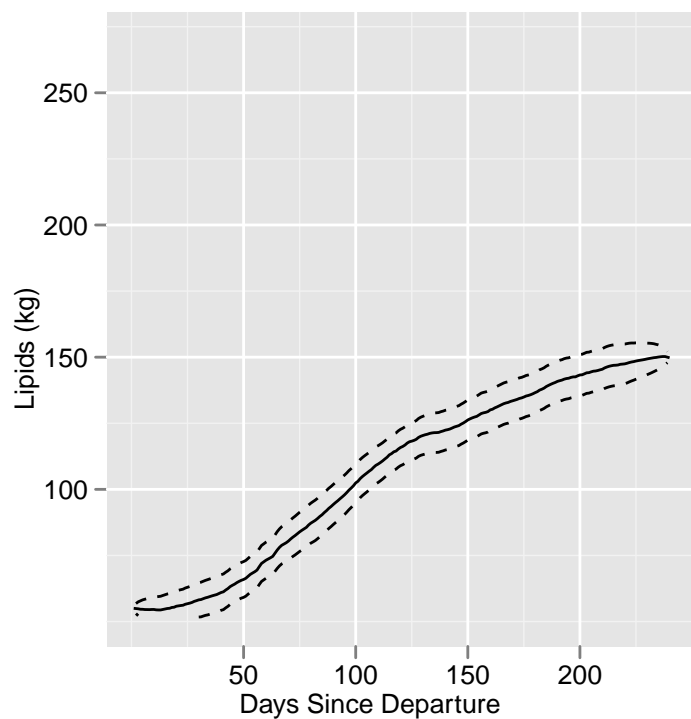

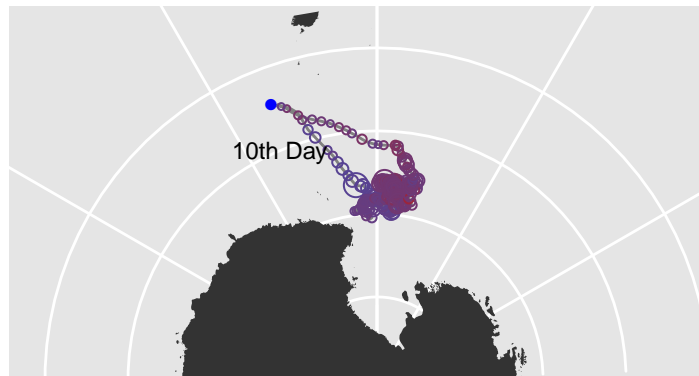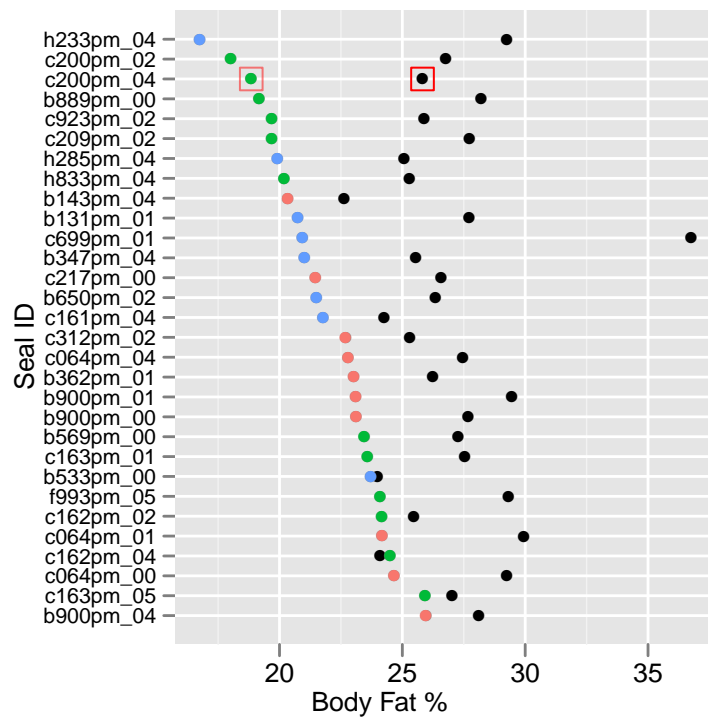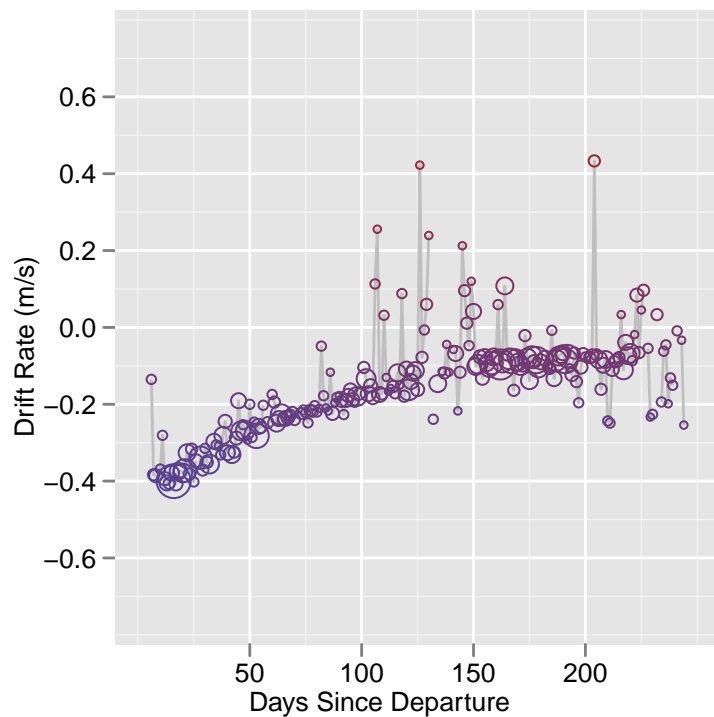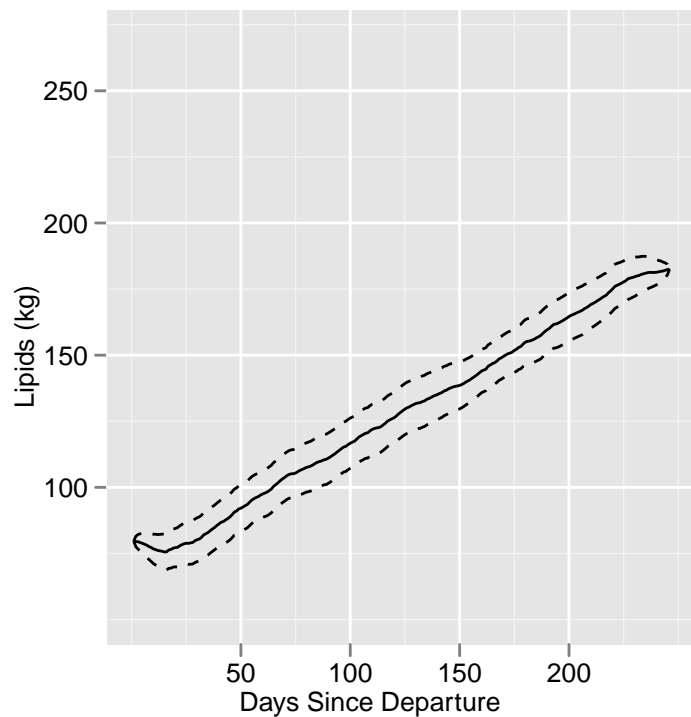

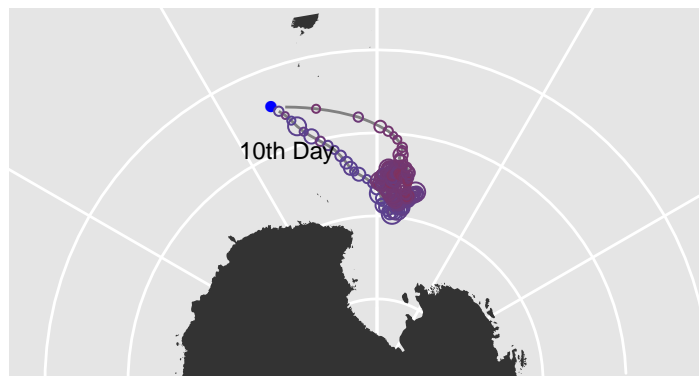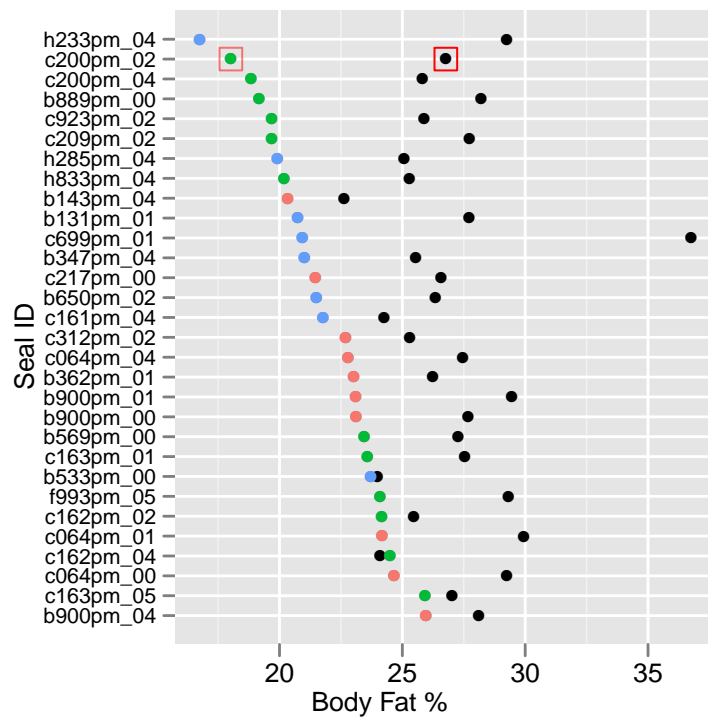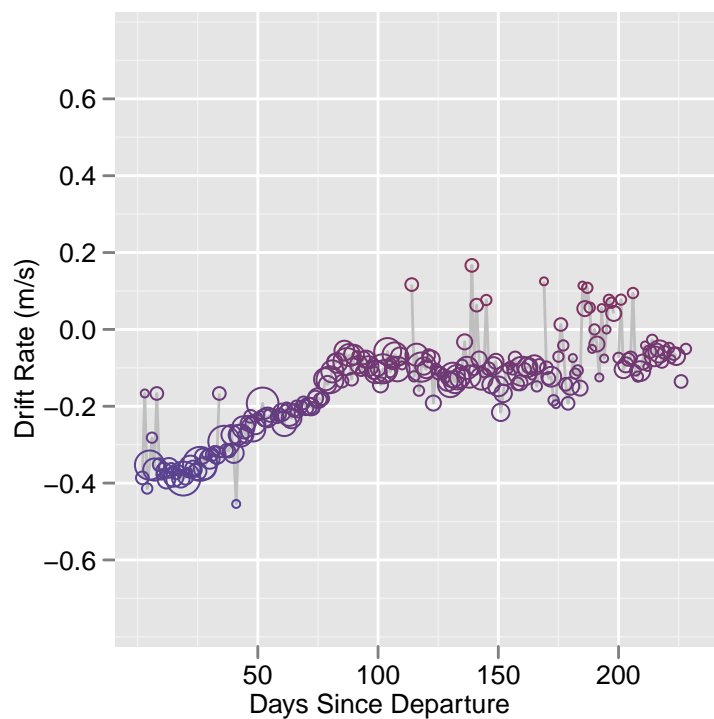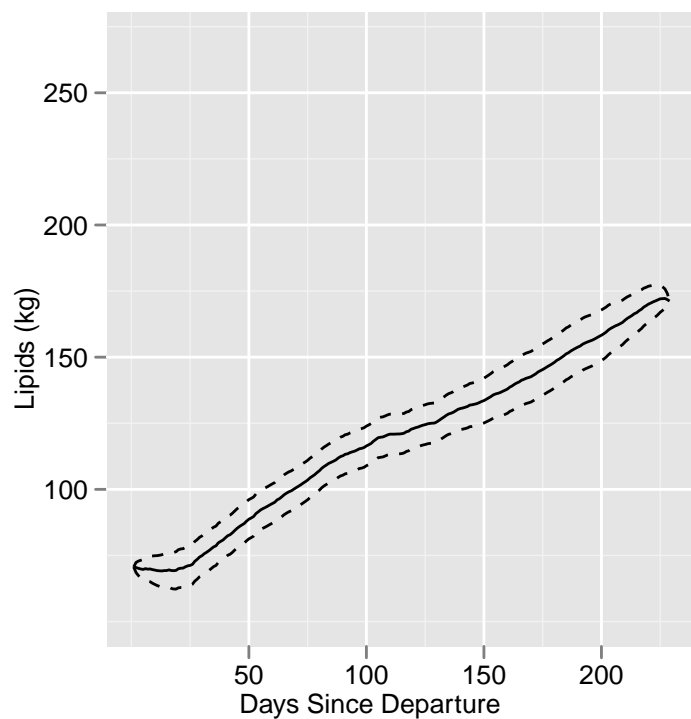

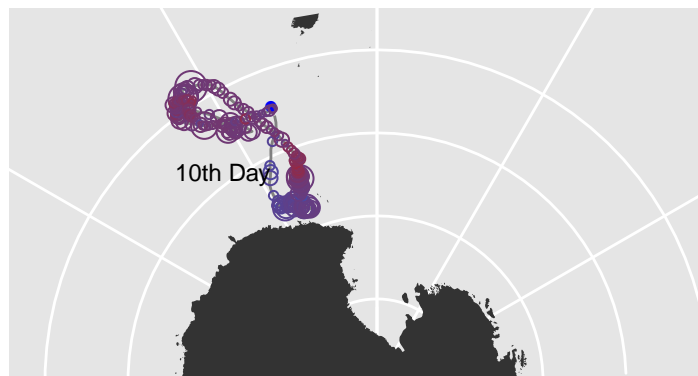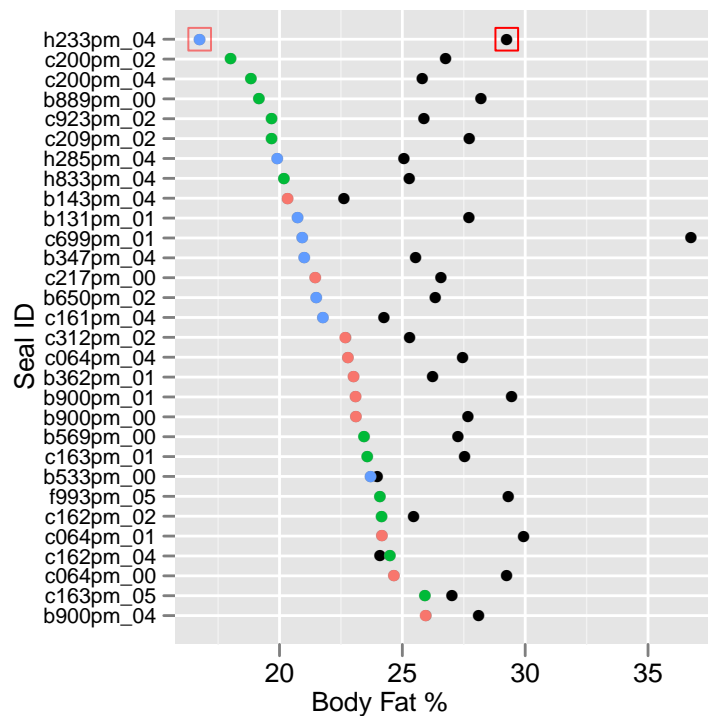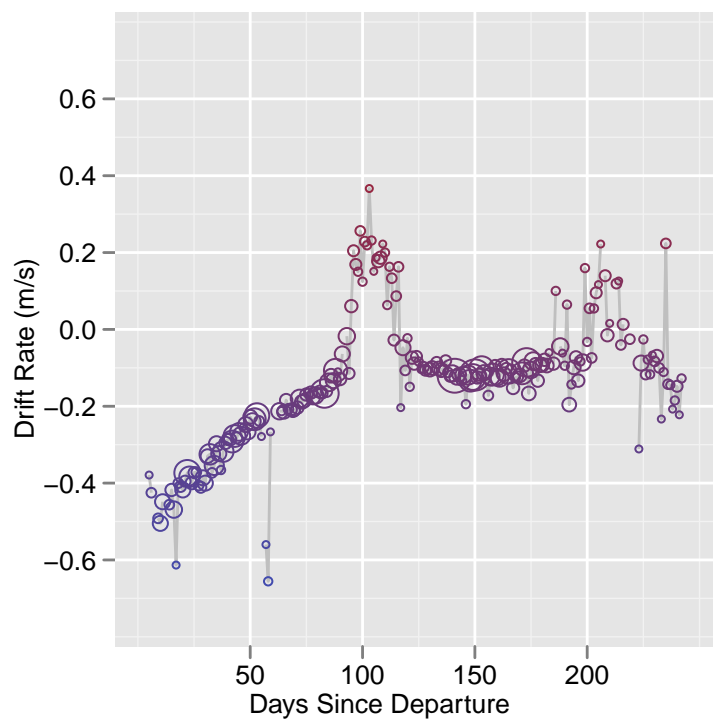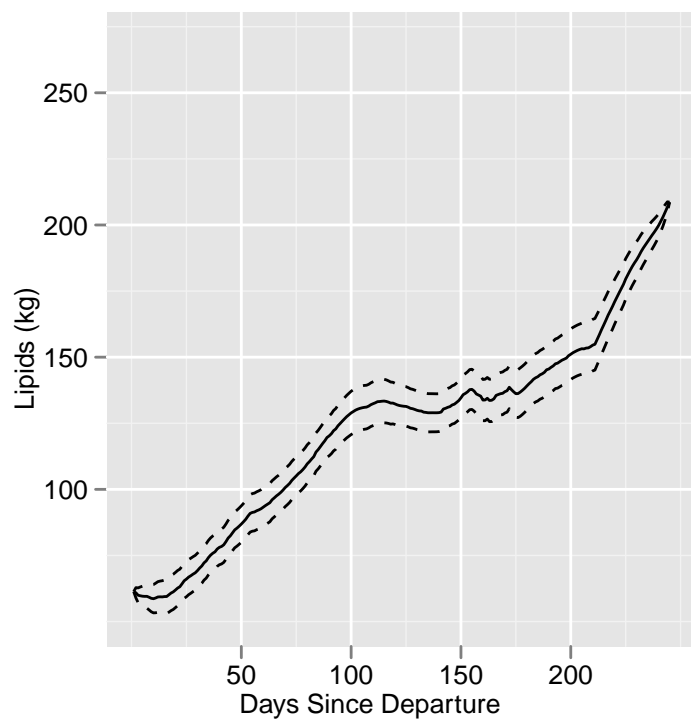

Supplement: Appendix S5 — Complete plots for each individual Southern elephant seal used in the analyses. Four plots per individual show: the overview map, the start and stop lipid percentage, the drift rate time series, and the time series of lipid estimates. [file jane0082-1300-sd5.pdf]
